# Copper-catalyzed aminoalkynylation of alkenes with hypervalent iodine reagents†
†Electronic supplementary information (ESI) available: Characterization data and experimental procedures. CCDC 1567005–1567007. For ESI and crystallographic data in CIF or other electronic format see DOI: 10.1039/c7sc03420b


**DOI:** 10.1039/c7sc03420b

**Published:** 2017-10-16

**Authors:** Kun Shen, Qiu Wang

**Affiliations:** a Department of Chemistry , Duke University , Durham , NC 27708–0346 , USA . Email: qiu.wang@duke.edu

## Abstract

A copper-catalyzed aminoalkynylation of alkenes is achieved with ethynylbenziodoxolone (EBX) reagents under mild conditions with only 1 mol% copper catalyst. This transformation allows for rapid construction of diverse important azahetereocycles and installation of valuable alkyne groups in one step. The developed method features remarkable substrate scope for both terminal and internal alkenes as well as different alkynyl groups, presenting great potential for broad applications in synthesis, bioconjugation, and molecular imaging.

## Abstract 2

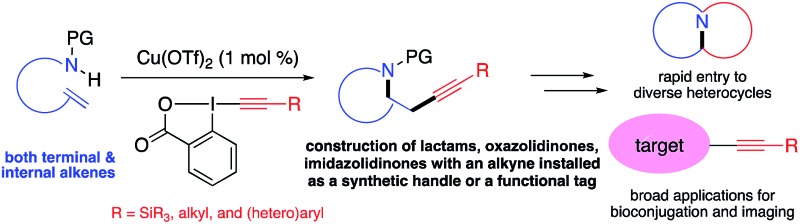
A copper-catalyzed aminoalkynylation reaction of alkenes is developed for construction of diverse azaheterocycles and installation of an alkyne group in one step, presenting broad applications in synthesis, bioconjugation, and molecular imaging.

## Introduction

**Fig. 1:**
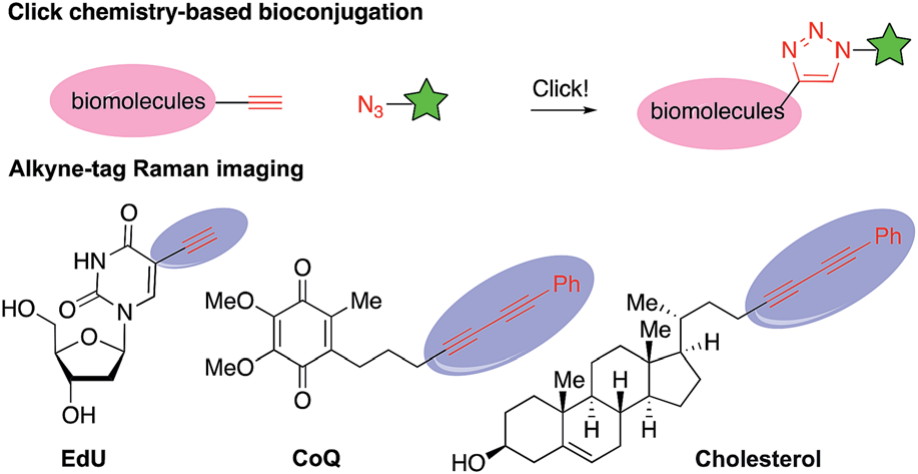
Representative applications of alkynes in bioconjugation and molecular imaging.

The carbon–carbon triple bond is one of the most valuable functional groups in organic chemistry. It features versatile reactivity as a synthetic intermediate in organic synthesis and has broad applications as a functional tag in biochemistry and materials sciences (Fig. 1).^1^ For example, alkynes have been widely used in “alkyne–azide click chemistry” for bioconjugation.^2^ Recently, alkynes have also been demonstrated as a Raman imaging tag because they show distinct, strong Raman scattering at ∼2150 cm^–1^, in a cellular silent region (1800–2800 cm^–1^) where most endogenous molecules show no Raman scattering.^3^ Therefore, developing efficient methods for installation of an alkyne group onto organic molecules is important and has attracted intense interests.

**Scheme 1:**
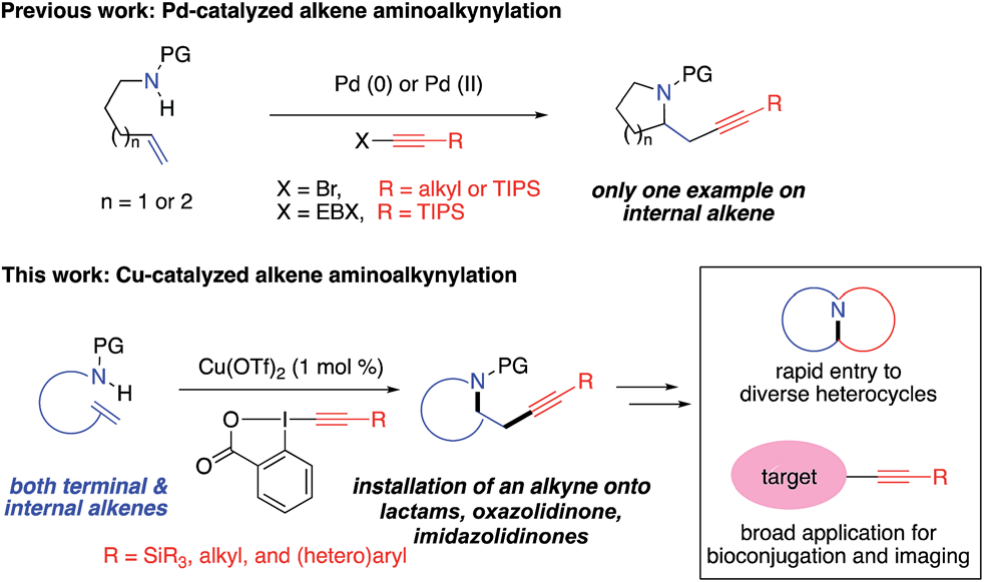
Alkene difunctionalization for installation of an alkyne group onto important azaheterocycles.

Alkene alkynylation represents an attractive and rapid approach to install alkyne groups onto target molecules from readily available alkenes. With azaheterocycles as the most valuable skeletons frequently found in biologically active natural products and pharmaceuticals,^4^ alkene aminoalkynylation is particularly valued for construction of azaheterocycles and installation of an alkyne group in a single step. Significant advances in this area have been reported by the Waser group (Scheme 1), including Pd-catalyzed alkene aminoalkynylation, as well as oxy- and carbo-alkynylation transformations using ethynylbenziodoxolones (EBX)^5^ or aliphatic bromoacetylenes.^6^,^7^ Despite these progress, only one example have been demonstrated on internal alkenes so far, impeding its utility to construct more complex, diverse azaheterocycles. Furthermore, reaction conditions need to be tailored for different alkyne precursors in previous methods, limiting the scope of alkyne groups for broad application in organic synthesis and chemical biology. Motivated by our interest in developing new, efficient methods to access important azaheterocycles^8^ and inspired by recent success in alkene functionalization with hypervalent iodine reagents,^9^ we here report our development of a copper-catalyzed selective aminoalkynylation reaction of both terminal and internal alkenes (Scheme 1). This transformation readily occurs with 1 mol% of copper catalyst under mild conditions and enables rapid access to a wide range of alkyne-labelled fused, and bridged azaheterocyles, which are commonly found in pharmaceutically relevant architectures.^4^ The aminoalkynylation method will enable broad applications of using alkynes as a handle for rapid entry to diverse azaheterocycles and as a functional tag in bioconjugation and Raman imaging.

## Results and discussion

### Reaction optimization

Our investigation of this alkene aminoalkynylation reaction began with unsaturated amide **1a** (Table 1), a model substrate that was demonstrated to undergo effectively copper-catalyzed aminocyclization in our previous studies.8c,9h,^10^ We chose ethynylbenziodoxolones (EBX) as the alkynyl precursors, as they have been successfully used in different alkynylation reactions with nucleophiles,^11^ C–H bonds,^12^ carbon radicals,^13^ and olefins.^5^ Encouragingly, aminoalkynylation product **3aa** was formed in 69% yield in the presence of Cu(OTf)_2_ in CH_3_CN at 80 °C (entry 1). Among the various copper salts examined, cationic copper species generally gave higher yields than neutral copper species, with Cu(OTf)_2_ most effective (entries 1–7). Without a copper catalyst, only trace amounts of **3aa** was observed, indicating the important role of a copper catalyst in this reaction (entry 8). Decreasing the catalyst loading was beneficial for the formation of **3aa** (entries 9 and 10) and elevating the temperature improved the efficiency of reactions (entries 10–12). In comparison to **2a**, other types of alkynyl-based hypervalent iodine reagents such as **2a′** and **2a′′** resulted in much lower yields (entries 13 and 14). Thus entry 11 was chosen as standard conditions for subsequent studies of this aminoalkynylation reaction.^14^

**Table 1:** Reaction condition optimizations^*a*^

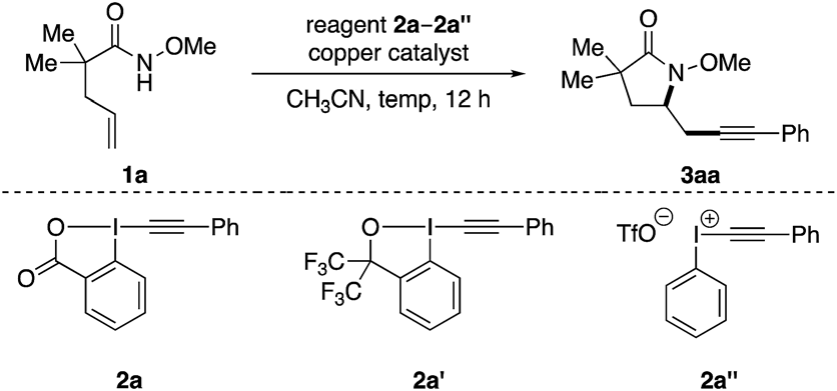				
Entry	Catalyst (mol%)	**2**	*T* (°C)	Yield^*b*^
1	Cu(OTf)_2_ (10)	**2a**	80	69%
2	Cu(OAc)_2_ (10)	**2a**	80	33%
3	Cu(acac)_2_ (10)	**2a**	80	34%
4	CuCl_2_ (10)	**2a**	80	26%
5	CuOTf 1/2PhCH_3_ (10)	**2a**	80	63%
6	Cu(CH_3_CN)_4_PF_6_ (10)	**2a**	80	69%
7	Cu(CH_3_CN)_4_BF_4_ (10)	**2a**	80	67%
8	—	**2a**	80	15%
9	Cu(OTf)_2_ (5)	**2a**	80	73%
10	Cu(OTf)_2_ (1)	**2a**	80	78%
**11**	**Cu(OTf)**_**2**_ **(1)**	**2a**	**100**	**84%**
12	Cu(OTf)_2_ (1)	**2a**	60	37%
13	Cu(OTf)_2_ (1)	**2a′**	100	57%
14	Cu(OTf)_2_ (1)	**2a′′**	100	<5%

^*a*^Reactions conditions: **1** (0.2 mmol, 1 equiv.), **2** (0.24 mmol, 1.2 equiv.), copper catalyst, CH_3_CN (1 mL) unless otherwise noted.

^*b*^Yields were determined by ^1^H NMR with CH_2_Br_2_ as an internal standard.

### Reaction scope

We examined the generality and efficiency of the aminoalkynylation reaction on different unsaturated amide derivatives (Table 2). Monosubstituted alkenes **1a–f** bearing various substitutes on the alkenyl chain all underwent smooth 5-*exo* cyclization and gave γ-lactam products **3aa–fa**. The reactions of substrates bearing substitutes on the backbone were more efficient, likely due to the favorable Thorpe–Ingold effect in the cyclization step. Unsaturated ureas **1g–h** and carbamates **1i–j** were effective in the aminoalkynylation reactions and provided desired imidazolidinones **3ga–ha** and oxazolidinones **3ia–ja**. 1,1-Disubstituted alkene **1k** led to γ-lactam **3ka** bearing a quaternary carbon center. δ-Lactam products **3ma–na** were also readily formed, with heteroarenes such as indoles and pyridines well tolerated in the reactions. Note that the diastereoselectivity of this reaction may be influenced by the substitution on the backbone, as observed in the formation of **3ea**, **3fa** and **3ha**. Remarkably, this aminoalkynylation reaction was applicable to internal alkenes, which are known to be challenging in metal-catalyzed alkene difunctionalization due to competing β-H elimination. The reaction of tri-substituted alkene **1o** gave aminoalkynylation product **3oa** bearing a quaternary carbon in 82% yield. The reactions of (*E*)- and (*Z*)-**1p** both led to the formation of product **3pa** in the same ratio of 2 : 1 diasteroselectivity, suggesting that (*E*)- and (*Z*)-**1p** may share the same intermediate leading to the product **3pa**. The reactions of cyclic internal alkenes well delivered fused-ring products **3qa** and **3ra** bridged-ring products **3sa–ua**. Overall, this aminoalkynylation reaction proved effective on a broad scope of olefin substrates that encompass diverse substitutions on both alkenes and backbones.

**Table 2:** Scope of alkenes and alkynyl groups for the aminoalkynylation reactions^*a*^ ^,^^*b*^

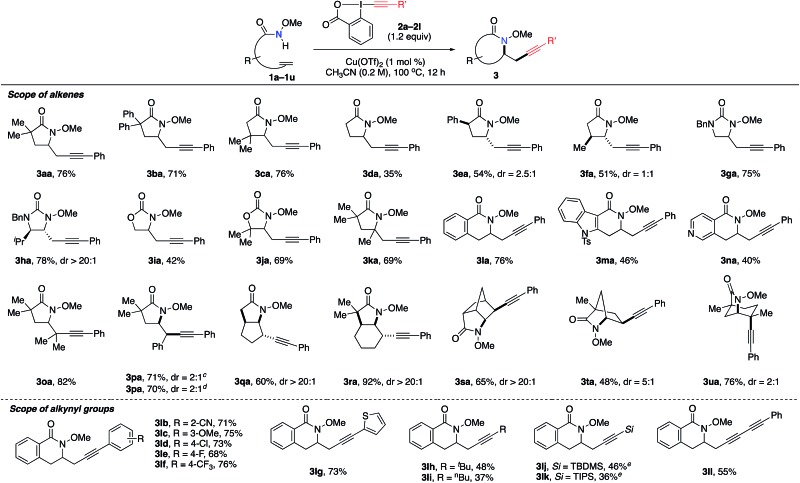

^*a*^Reactions performed with **1** (0.3 mmol, 1 equiv.), **2** (1.2 equiv.), Cu(OTf)_2_ (1 mol%) in CH_3_CN at 100 °C, unless otherwise noted.

^*b*^Isolation yield.

^*c*^From *trans* alkene.

^*d*^From *cis* alkene.

^*e*^Cu(CH_3_CN)_4_BF_4_ (1 mol%) at 60 °C.

Next, the scope of alkynylation reagents **2** was examined in the reactions with alkene **1l** (Table 2). As seen in the formation of **3lb–lf**, both electron-donating and electron-withdrawing groups on the phenyl group were well tolerated. Neither the electronic nor steric properties of the substitutes on the phenyl group significantly influence the reaction efficiency. The thiophene moiety, an electron-rich heterocycles known for direct alkynylation,12*a* is compatible in this reaction, affording product **3lg** in good yield. Alkynylation reagents bearing alkyl substitutes were also applicable (**3lh** and **3li**), albeit in much lower yields compared to aromatic substitutes. TBS-EBX and TIPS-EBX reagents were also feasible in this transformation (**3lj** and **3lk**). Finally, conjugated dialkyne was readily incorporated into the aminoalkynylation product **3ll**. Note that conjugated dialkyne groups are known to offer stronger Raman signal compared to other types of alkynes as potential Raman imagine tags.3b,e

### Mechanism study

**Scheme 2:**
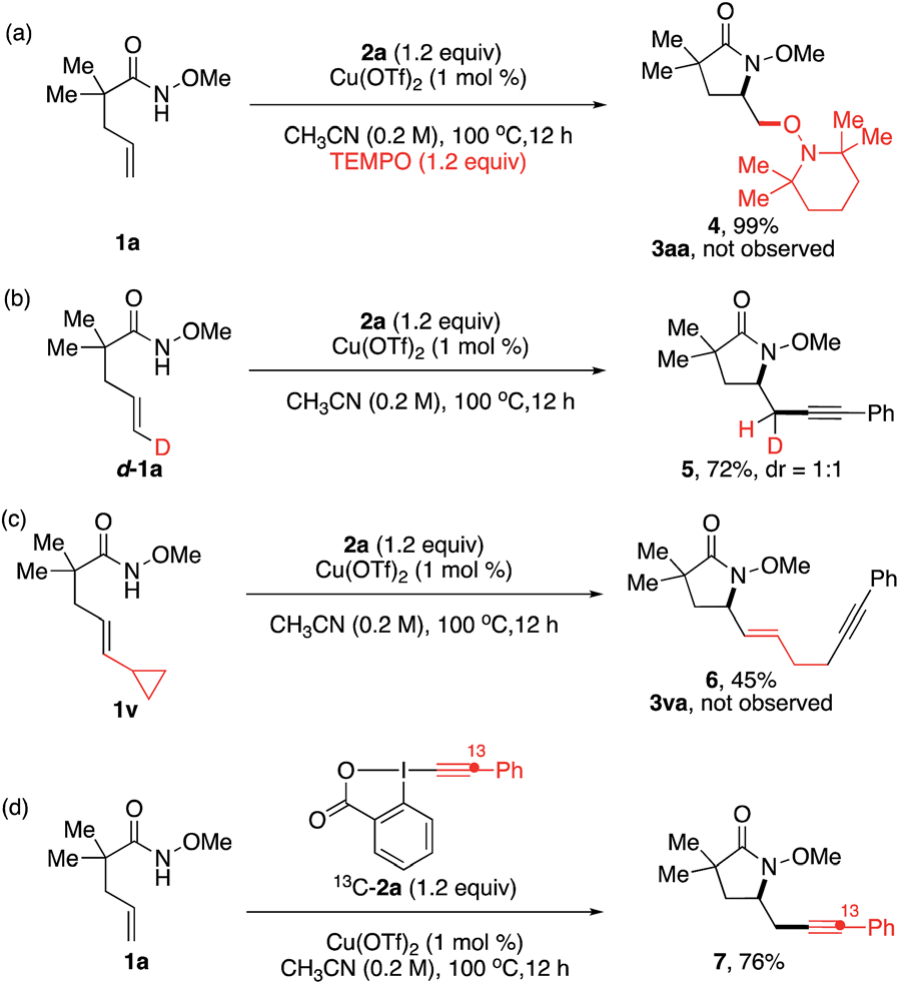
Mechanism investigations.

A series of control experiments were performed to obtain mechanistic insights on this aminoalkynylation reaction (Scheme 2). In the presence of TEMPO as a radical scavenger, the reaction of model substrates **1a** and **2a** provided aminoxygenation product **4** in 99% yield (Scheme 2a). The reaction of the *trans*-d-substituted alkenyl amide **d*-*1a** gave a 1 : 1 mixture of d-substituted diastereomers **5** in 72% yield (Scheme 2b). Both results indicate a radical nature of the intermediate after the intramolecular amino-cupration step, which is consistent with the loss of stereochemistry observed in the formation of **3pa** from (*E*)- and (*Z*)-**1p** in Table 2. To confirm this speculation, we examined the aminoalkynylation reaction using **1v**, an unsaturated amide containing a standard radical clock cyclopropane moiety at the vinyl position (Scheme 2c). The reaction provided ring-opened product **6** in 45% yield with no detection of **3va**, confirming the presence of radical alkyl intermediates after the aminocyclization step. Finally, we used ^13^C labelled Ph–EBX ^13^C-**2a** to probe the alkynylation step, revealing the exclusive presence of ^13^C atom at its original position of **7** (Scheme 2d).

**Scheme 3:**
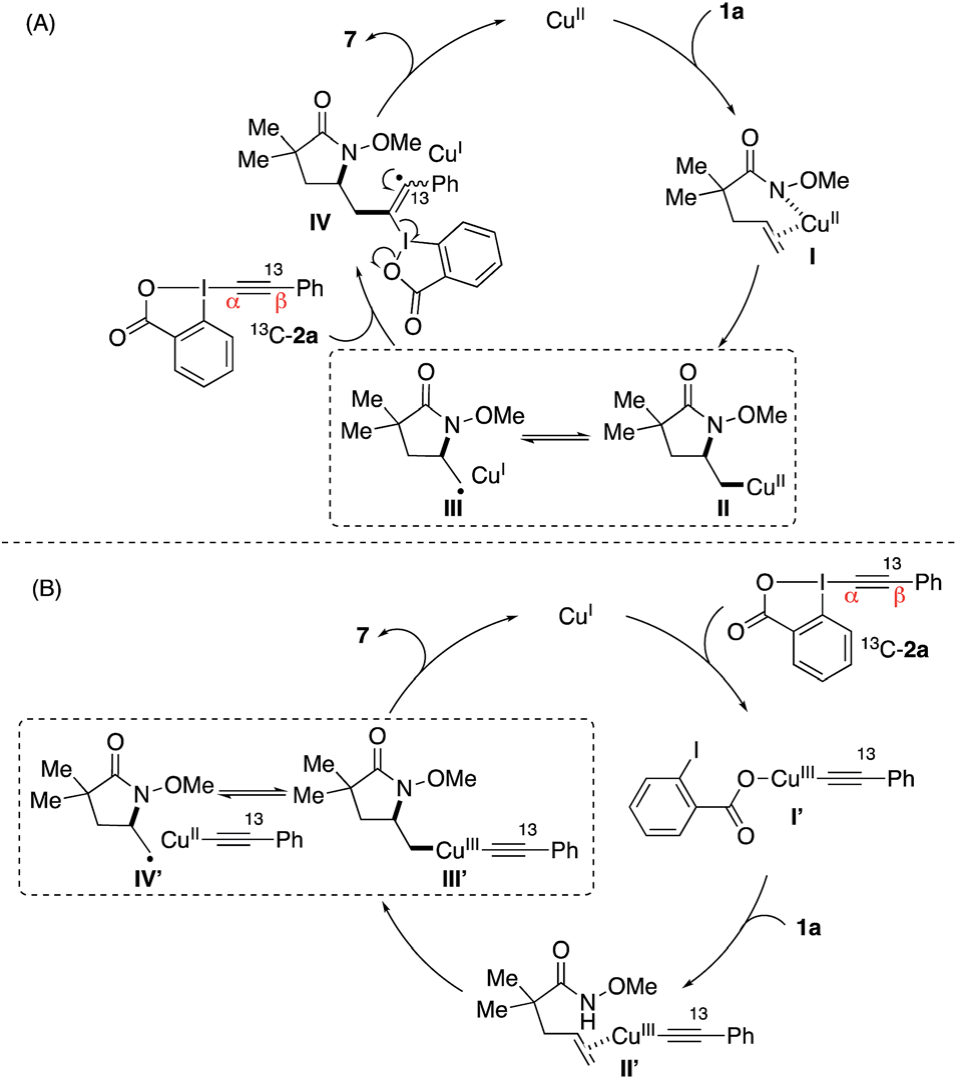
Proposed reaction pathways.

Based on these results, two possible reaction pathways are proposed for this copper-catalyzed aminoalkynylation reaction (Scheme 3).^15^ In pathway A, the reaction may be initiated by the coordination of copper catalyst with alkene **1a** followed by the intramolecular aminocupration.^10^ The resulting alkyl–Cu intermediate **II** may undergo a reversible C–Cu(ii) homolysis to form radical intermediate **III**, which would subsequently attack the α-position of the alkynyliodonium salt **2a**. Finally, the β-elimination of intermediate **IV** would lead to product **3** and regeneration of the copper catalyst. In an alternative pathway (B), the reaction may be initiated by the copper-catalyzed activation of alkynyliodonium salt **2a**. The aminocyclization would occur *via* copper intermediates **I′** and **II′** to form copper–alkyl complex **III′**, which would undergo similar homolysis to form a radical intermediate **IV′** and could lead to the formation of product **3**. Note that the exclusive presence of ^13^C atom at its original position in the reaction of EBX reagent ^13^C-**2a** (Scheme 2d) indicated the involvement of α-addition in the alkynylation step in either pathway (A) or (B), rather than the β-addition of the alkyne followed by the 1,2-shift of the Ph group.11i,l,^16^

### Synthetic applications

**Scheme 4:**
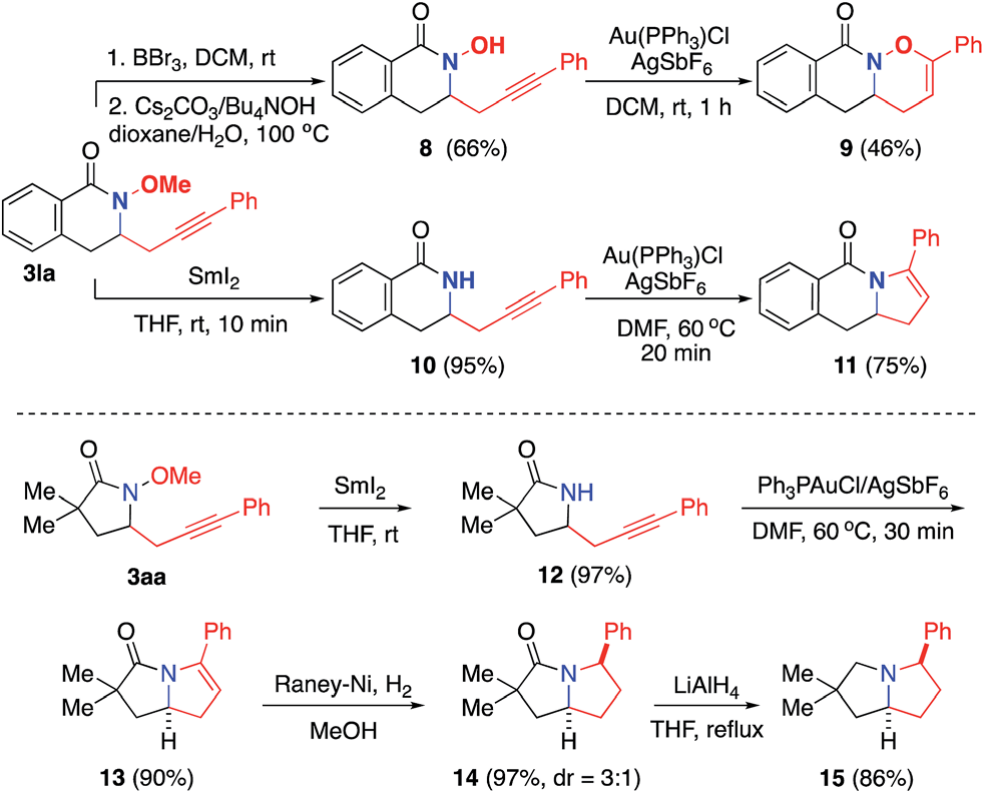
Selective construction of diverse fused heterocycles.

**Scheme 5:**
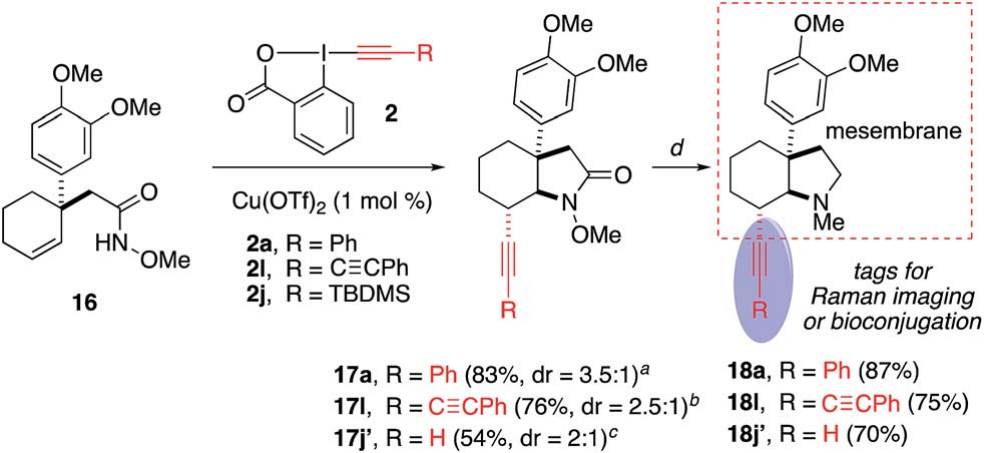
Synthesis of alkyne-labelled derivatives of mesembrane. ^*a*^**2a** (1.2 equiv.), Cu(OTf)_2_ (1 mol%), 100 °C. ^*b*^**2l** (1.2 equiv.), Cu(CH_3_CN)_4_BF_4_ (1 mol%), 100 °C. ^*c*^**2j** (1.2 equiv.), Cu(CH_3_CN)_4_BF_4_ (1 mol%), 60 °C; then TBAF (1.33 equiv.), rt. ^*d*^(i) SmI_2_, THF, rt; (ii) NaH, MeI, THF, rt; (iii) Rh(acac)(cod), PhSiH_3_, THF, 50 °C.

We next explored the synthetic utility of alkynyl group for the construction of various bicyclic heterocycles (Scheme 4).^17^ First, using **3la**, we demonstrate the advantage of the methoxy protecting group of the amide to achieve selective gold-catalyzed oxy- or amino-cyclization onto the alkyne group. For example, upon the removal of methyl group, the resulting hydroxylamide **8** underwent a Au-catalyzed *endo* oxycyclization to form oxazinane **9**. On the other hand, the demethoxylation of **3la** provided amide **10**, which was transformed into dihydropyrole **11** by an analogous Au-catalyzed aminocyclization. Furthermore, we employed the aminoalkynylation product **3aa** to complete the synthesis of a series of important pyrrolizidine derivatives **13–15**.^18^ Finally, this aminoalkynylation method was successfully applied for the preparation of alkyne-labelled derivatives of alkaloid mesembrane (Scheme 5). These readily installed alkynyl groups are expected to serve as valuable labelling tools to facilitate future studies regarding understanding its antidepressant potential and mode of action.^19^

## Conclusions

In summary, we have developed a new copper-catalyzed alkene aminoalkynylation reaction that is effective on an extensive scope of alkene substrates and EBX reagents. This method enables simultaneous construction of valuable azahetereocyclic skeletons and the installation an alkynyl group, which will greatly advance broad applications of using alkynes as a handle for rapid entry to complex, diverse heterocycles and as a functional tag in bioconjugation and Raman imaging.

## Conflicts of interest

There are no conflicts to declare.

## Supplementary Material

Supplementary information

Crystal structure data
