# Supplementary material for: Copper-catalyzed aminoalkynylation of alkenes with hypervalent iodine reagents
Source: Chem Sci. 2017 Oct 16;8(12):8265–70. doi: 10.1039/c7sc03420b (PMC5857935; doi:10.1039/c7sc03420b)
Supplement: Supplementary file 1 [file SC-008-C7SC03420B-s001.pdf]

Electronic Supplementary Material (ESI) for Chemical Science.
This journal is © The Royal Society of Chemistry 2017

Copper-Catalyzed Aminoalkynylation of Alkenes with Hypervalent Iodine Reagents

Kun Shen and Qiu Wang*

Department of Chemistry, Duke University, Durham, North Carolina 27708

Email: qiu.wang@duke.edu

Supporting Information

__Content__

I. General Procedures S1

II. Materials and Instrumentation S1–S2

III. Reaction Condition Optimization for Alkene Aminoalkynylation S2–S4

IV. Synthesis of Hypervalent Iodine Reagent 2l S4–S5

V. General Procedure for Alkene Aminoalkynylation Reaction S5–S17

VI. Mechanism Investigation S17–S19

VII. Synthesis of Bicyclic Heterocycles S19–S23

VIII. Synthesis of Alkyne-Labeled Mesembrane S24–S30

IX. References S30

X. NMR Spectra S31–S173

XI. X-Ray Data S174–S201

I. General Procedures. Glassware and stir bars were dried in an oven at 140 °C for at least 12 h and then cooled in a desiccator cabinet over Drierite prior to use. Optimization and substrate screens were performed in 8-mL microwave vials. Vials were fitted with crimp top septa under a positive pressure of N_2 that had been passed through a column (5 x 20 cm) of Drierite, unless otherwise noted. All other reactions were performed in round-bottom flasks sealed with rubber septa. Plastic syringes or glass pipets were used to transfer liquid reagents. Reactions were stirred magnetically using Teflon-coated, magnetic stir bars. Analytical thin-layer chromatography (TLC) was performed using aluminum plates pre-coated with 0.25 mm of 230–400 mesh silica gel impregnated with a fluorescent indicator (254 nm). TLC plates were visualized by exposure to ultraviolet light and/or exposure to $KMnO_4$ stain. Organic solutions were concentrated under reduced pressure using a rotary evaporator. Flash-column chromatography was performed on silica gel (60 Å, standard grade) or with pre-packed FLASH silica gel columns.

II. Materials and Instrumentation. Nuclear magnetic resonance spectra were recorded at ambient temperature (unless otherwise stated) on 400 MHz or 500 MHz spectrometers. All values for proton chemical shifts are reported in parts per million (δ) and are referenced to the residual protium in $CDCl_3$ (δ 7.26). All values for carbon chemical shifts are reported in parts per million (δ) and are referenced to the carbon resonances in $CDCl_3$ (δ 77.0). NMR data are represented as follows: chemical shift, multiplicity (s = singlet, d = doublet, t = triplet, q = quartet, quin = quintet, m = multiplet, br = broad), coupling constant

(Hz), and integration. Infrared spectroscopic data are reported in wavenumbers (cm^{-1}). High-resolution mass spectra were obtained using a liquid chromatography-electrospray ionization and Time-of-flight mass spectrometer. Hypervalent iodine reagents were synthesized according to literature procedures.[1]

III. Reaction Condition Optimization for Alkene Aminoalkynylation.

a. Screening of Catalyst and Catalyst loading[a]

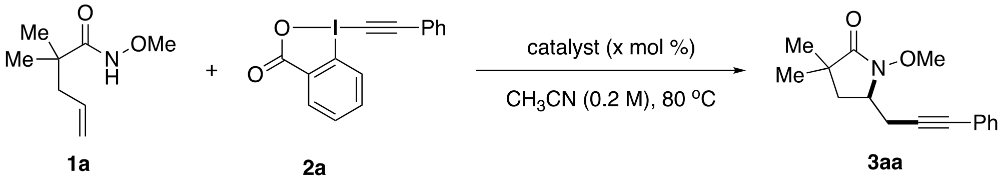

entry	catalyst	catalyst loading	yield[b] (%)
1	$Cu(OTf)_2$	10 mol%	69
2	$Cu(OAc)_2$	10 mol%	33
3	$Cu(acac)_2$	10 mol%	34
4	$CuCl_2$	10 mol%	26
5	CuOTf•1/2PhCH$_3$	10 mol%	63
6	$Cu(CH_3CN)_4PF_6$	10 mol%	69
7	$Cu(CH_3CN)_4BF_4$	10 mol%	67
8	-	-	15
9	$Cu(OTf)_2$	5 mol%	73
10	$Cu(OTf)_2$	2 mol%	76
11	$Cu(OTf)_2$	1 mol%	78

[a]Reaction Conditions: The reactions were performed in a sealed tube with **1a** (0.20 mmol, 1.0 equiv), **2a** (1.2 equiv), catalyst (1–10 mol%) and CH_3CN (1 mL) at 80 °C for 4 h, unless otherwise noted. [b]The yield was determined by ^{1}H NMR with diboromethane as an internal standard.

b. Screening of Temperature[a]

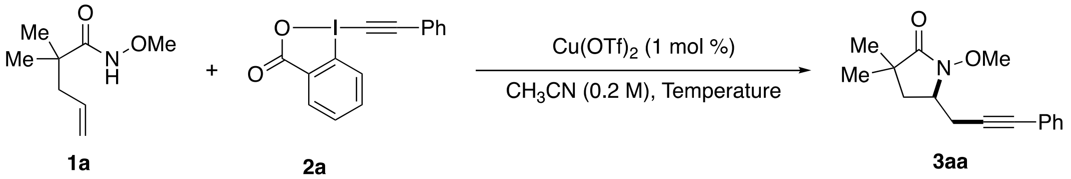

entry	T (°C)	Time	yield[b] (%)
1	rt	12 h	0
2	40	12 h	15
3	60	12 h	37
4	80	4 h	78
5	100	4 h	84

[a]Reaction Conditions: The reactions were performed in a sealed tube with **1a** (0.20 mmol, 1.0 equiv), **2a** (1.2 equiv), $Cu(OTf)_2$ (1 mol%) and CH_3CN (1 mL), unless otherwise noted. [b]The yield was determined by ^{1}H NMR with diboromethane as an internal standard.

c. Screening of Additives[a]

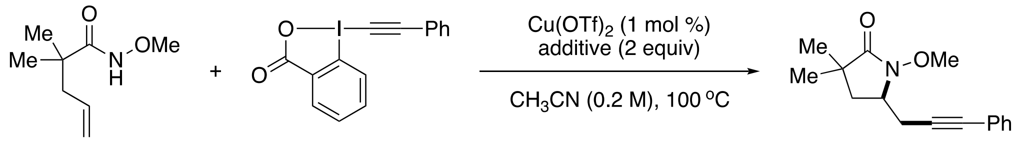

1a 2a 3aa

entry	additives	yield[b] (%)
1	-	84
2[c]	-	0[d]
3	K_2CO_3	28
4	Na_2CO_3	37
5	NEt(^{i}Pr)$_2$	0
6	2,6-lutidine	49
7	2,6-di-*tert*-butyl-4-methylpyridine	67

[a]Reaction Conditions: The reactions were performed in a sealed tube with **1a** (0.20 mmol, 1.0 equiv), **2a** (1.2 equiv), Cu(OTf)$_2$ (1 mol%), additive (2.0 equiv) and CH_3CN (1 mL) at 100 °C for 12 h, unless otherwise noted. [b]The yield was determined by ^{1}H NMR with diboromethane as an internal standard. [c] The reaction was run without **2a**. [d] 55% recovery of **1a** and 32% of hydroamination product.

d. Screening of Ligands[a]

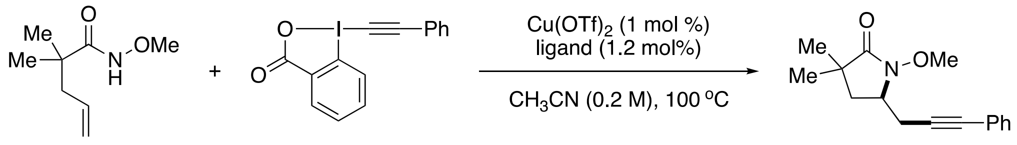

1a 2a 3aa

entry	ligand	yield[b] (%)
1	-	84
2	bpy	44
3	1,10-phenanthroline	36
4	bathophenanthroline	45
5	bathocuproine	44
6	biquinoline	57
7	di(2-pyridyl) ketone	64
8	binap	71
9	dppbz	70
10	dpephos	75
11	xantphos	70

[a]Reaction Conditions: The reactions were performed in a sealed tube with **1a** (0.20 mmol, 1.0 equiv), **2a** (1.2 equiv), Cu(OTf)$_2$ (1 mol%), ligand (1.2 mol%) and CH_3CN (1 mL) at 100 °C for 12 h, unless otherwise noted. [b]The yield was determined by ^{1}H NMR with diboromethane as an internal standard.

e. Screening of Hypervalent Iodine Reagents[a]

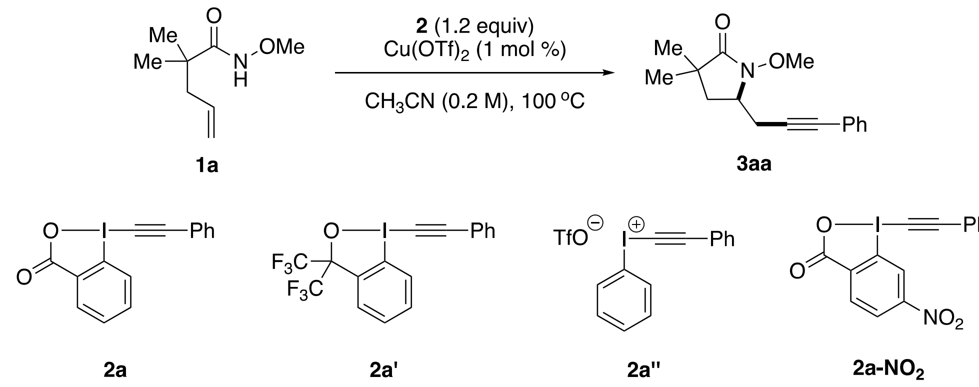

entry	**2**	yield[b] (%)
1	**2a**	84
2	**2a'**	57
3	**2a''**	<5
4	**2a-NO$_2$**	19

[a]Reaction Conditions: The reactions were performed in a sealed tube with **1a** (0.20 mmol, 1.0 equiv), **2** (1.2 equiv), Cu(OTf)$_2$ (1 mol%) and CH_3CN (1 mL) at 100 °C for 5 h, unless otherwise noted. [b]The yield was determined by ^{1}H NMR with diboromethane as an internal standard.

f. Reactions of Amides Bearing Different Protecting Groups[a]

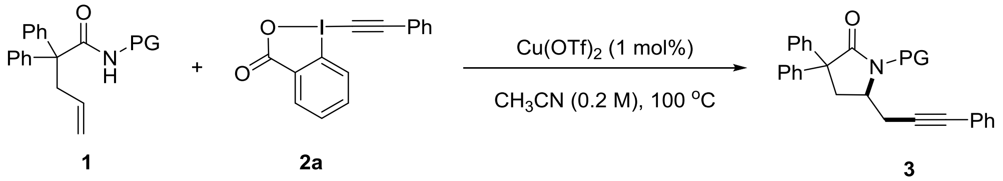

entry	**1**	**3**	yield (%)[b]
1	**1b**, PG = OMe	**3ba**	71
2	**1b-S1**, PG = Ts	**3ba-S1**	0[c] (100)[d]
3	**1b-S2**, PG = H	**3ba-S2**	0[c] (86)[d]
4	**1b-S3**, PG = Ph	**3ba-S3**	0[c] (100)[d]
5	**1b-S4**, PG = Bn	**3ba-S4**	0[c] (100)[d]

[a]Reaction Conditions: The reactions were performed in a sealed tube with **1a** (0.20 mmol, 1.0 equiv), **2a** (1.2 equiv), Cu(OTf)$_2$ (1 mol%) and CH_3CN (1 mL), unless otherwise noted. [b]The yield was determined by ^{1}H NMR with diboromethane as an internal standard. [c]Desired aminoalkynylation product not detected by either GC-MS or ^{1}H NMR. [d]The recovery of starting material **1** was indicated in the parenthesis.

IV. Synthesis of Hypervalent Iodine Reagent 2l

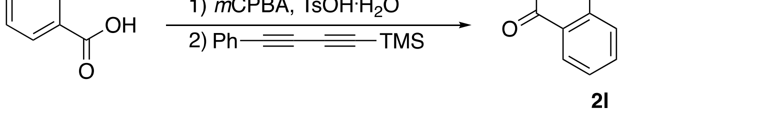

Following a reported procedure with appropriate modifications,[1] 2-iodobenzoic acid (4.96 g, 20.0 mmol, 1.0 equiv), *para*-toluenesulfonic acid monohydrate (3.8 g, 20.0 mmol, 1.0 equiv) and *meta*-chloroperoxybenzoic

acid (≤ 77%, 5.4 g, 22.0 mmol, 1.1 equiv) were dissolved in CH_2Cl_2 (30 mL) and 2,2,2-trifluoroethanol (30 mL). The mixture was stirred at room temperature under N_2 for 1 h, after which trimethyl(phenylbuta-1,3-diyn-1-yl)silane (5.54 g, 28.0 mmol, 1.4 equiv) was added in one portion. The reaction mixture was stirred at room temperature overnight, filtered and concentrated *in vacuo*. The resulting oil was dissolved in CH_2Cl_2 (100 mL) and under vigorous stirring, saturated solution of $NaHCO_3$ (100 mL) was added. The mixture was stirred for 1 h, the two layers were separated and the aqueous layer was extracted with CH_2Cl_2 (3 x 50 mL). The combined organic layers were dried over $NaSO_4$, filtered, and concentrated *in vacuo*. Recrystallization in CH_3CN afford **2l** (2.38 g, 6.4 mmol, 32%) as a yellow solid. R_f = 0.47 (100% EtOAc); ^{1}H NMR (500 MHz, DMSO): δ 8.25 (d, *J* = 8.0 Hz, 1H), 8.12 (d, *J* = 7.0 Hz, 1H), 7.93 (t, *J* = 7.0 Hz, 1H), 7.82 (t, *J* = 7.0 Hz, 1H), 7.69 (d, *J* = 7.0 Hz, 2H), 7.55 (t, *J* = 7.5 Hz, 1H), 7.48 (t, *J* = 7.5 Hz, 2H); ^{13}C NMR (125 MHz, DMSO): δ 166.3, 135.4, 133.2, 131.7, 131.5, 131.4, 131.0, 129.1, 127.6, 119.0, 116.7, 87.2, 80.1, 73.0, 50.8; IR (neat): 3057, 2918, 2098, 1644, 1437, 1285, 1237, 1118, 826, 746, 682 cm^{-1}; HRMS (m/z) Calcd for ($C_{17}H_{10}IO_2$) ($[M+H]^+$): 372.9720; found: 372.9725.

V. General Procedure for Alkene Aminoalkynylation Reaction.

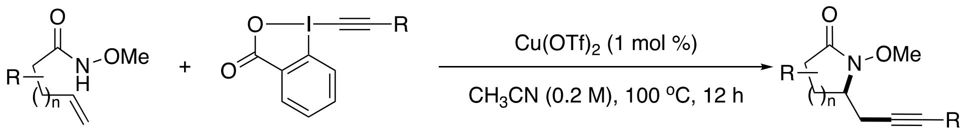

1 **2** **3**

To a reaction tube charged with *N*-methoxyamide **1** (0.3 mmol, 1.0 equiv) and hypervalent iodine reagent **2** (0.36 mmol, 1.2 equiv), was added $Cu(OTf)_2$ (1.09 mg, 0.003 mmol, 1 mol %) and CH_3CN (1.5 mL). The reaction tube was capped and the resulting mixure was allowed to stir at 100 °C for 12 h. After cooling down to room temperature, the reaction mixture was filtered through a pad of basic Al_2O_3 and washed with EtOAc (15 mL). The filtrate was concentrated *in vacuo*. The resulting crude mixture was subject to flash column chromatography to provide the aminoalkynylation product **3**.

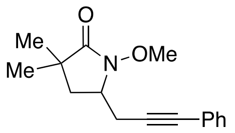

1-Methoxy-3,3-dimethyl-5-(3-phenylprop-2-yn-1-yl)pyrrolidin-2-one (3aa). Purification by column chromatography (20% EtOAc in hexanes) gave **3aa** as a yellow oil (58.4 mg, 76% yield); R_f = 0.30 (33% EtOAc in hexanes); ^{1}H NMR (400 MHz, $CDCl_3$): δ 7.39–7.27 (m, 2H), 7.27–7.16 (m, 3H), 3.82 (s, 3H), 3.82–3.74 (m, 1H), 2.81–2.68 (m, 2H), 1.99 (dd, *J* = 12.8, 7.2 Hz, 1H), 1.81 (dd, *J* = 12.8, 8.4 Hz, 1H), 1.18 (s, 3H), 1.09 (s, 3H); ^{13}C NMR (100 MHz, $CDCl_3$): δ 175.8, 131.1, 127.9, 127.7, 122.7, 84.1, 83.3, 62.5, 53.4, 37.0, 36.7, 25.3, 24.6, 23.2; IR (neat): 2964, 2932, 2869, 1707, 1441, 1365, 1240, 1037, 1014, 926, 755, 729, 691 cm^{-1}; HRMS (m/z) Calcd for ($C_{16}H_{20}NO_2$) ($[M+H]^+$): 258.1489; found: 258.1490.

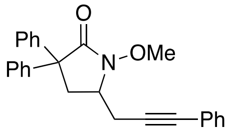

1-Methoxy-3,3-diphenyl-5-(3-phenylprop-2-yn-1-yl)pyrrolidin-2-one (3ba). Purification by column chromatography (10% EtOAc in hexanes) gave **3ba** as a yellow oil (81.6 mg, 71% yield); R_f = 0.65 (33% EtOAc in hexanes); ^{1}H NMR (500 MHz, $CDCl_3$): δ 7.50–7.10 (m, 15H), 3.98 (s, 3H), 3.01 (dd, *J* = 13.0, 6.5 Hz, 1H), 2.95–2.85 (m, 2H), 2.78 (dd, *J* = 13.0, 9.0 Hz, 1H); ^{13}C NMR (125 MHz, $CDCl_3$): δ 172.1, 143.7, 141.1, 131.5, 128.4, 128.26, 128.20, 128.0, 127.7, 127.6, 122.8, 84.14, 84.12, 63.2, 54.5, 54.0, 37.8, 23.1; IR (neat): 2935, 1705, 1489, 1443, 1367, 1238, 1043, 754, 690 cm^{-1}; HRMS (m/z) Calcd for ($C_{26}H_{24}NO_2$) ([M+H]$^+$): 382.1802; found: 382.1802.

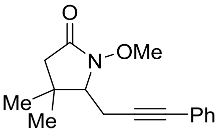

1-Methoxy-4,4-dimethyl-5-(3-phenylprop-2-yn-1-yl)pyrrolidin-2-one (3ca). Purification by column chromatography (33% EtOAc in hexanes) gave **3ca** as a yellow oil (58.4 mg, 76% yield); R_f = 0.18 (33% EtOAc in hexanes); ^{1}H NMR (500 MHz, $CDCl_3$): δ 7.45–7.35 (m, 2H), 7.34–7.24 (m, 3H), 3.86 (s, 3H), 3.58 (dd, *J* = 6.5, 4.0 Hz, 1H), 2.82 (dd, *J* = 17.0, 6.5 Hz, 1H), 2.75 (dd, *J* = 17.0, 4.0 Hz, 1H), 2.42 (d, *J* = 17.0 Hz, 1H), 2.13 (d, *J* = 17.0 Hz, 1H), 1.28 (s, 6H); ^{13}C NMR (125 MHz, $CDCl_3$): δ 170.2, 131.3, 128.2, 128.0, 123.0, 85.1, 83.5, 65.2, 62.5, 43.0, 33.5, 29.8, 23.2, 19.4; IR (neat): 2960, 2931, 1706, 1490, 1441, 1368, 1241, 1064, 756, 691 cm^{-1}; HRMS (m/z) Calcd for ($C_{16}H_{20}NO_2$) ([M+H]$^+$): 258.1489; found: 258.1488.

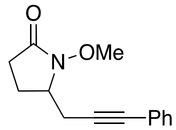

1-Methoxy-5-(3-phenylprop-2-yn-1-yl)pyrrolidin-2-one (3da). Purification by column chromatography (33% EtOAc in hexanes) gave **3da** as a colorless oil (23.7 mg, 35% yield); R_f = 0.10 (33% EtOAc in hexanes); ^{1}H NMR (400 MHz, $CDCl_3$): δ 7.40–7.30 (m, 2H), 7.30–7.20 (m, 3H), 3.95–3.85 (m, 1H), 3.84 (s, 3H), 2.85–2.65 (m, 2H), 2.52–2.38 (m, 1H), 2.35–2.10 (m, 2H), 2.09–1.93 (m, 1H); ^{13}C NMR (100 MHz, $CDCl_3$): δ 171.3, 131.4, 128.2, 128.0, 122.9, 84.2, 83.3, 63.0, 55.8, 26.9, 23.3, 20.7; IR (neat): 2936, 1701, 1489, 1440, 1360, 1277, 1052, 964, 756, 692 cm^{-1}; HRMS (m/z) Calcd for ($C_{14}H_{26}NO_2$) ([M+H]$^+$): 230.1176; found: 230.1177.

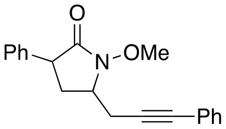

1-Methoxy-3-phenyl-5-(3-phenylprop-2-yn-1-yl)pyrrolidin-2-one (3ea). The crude reaction mixture contains two diastereoisomers with d.r. = 2.5:1 detected by ^{1}H NMR. Purification by column chromatography (25% EtOAc in hexanes) gave **3ea** as a mixture of two separable diastereoisomers (35.2 mg major and 14.1 mg minor, 54% yield; relative stereochemistry was assigned on the basis of ^{1}H NMR.[2]).

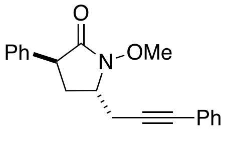

Major diastereomer: Yellow oil; R_f = 0.35 (33% EtOAc in hexanes); ^{1}H NMR (500 MHz, $CDCl_3$): δ 7.50–7.20 (m, 10H), 4.20–4.10 (m, 1H), 3.94 (s, 3H), 3.86 (dd, *J* = 10.0, 8.0 Hz, 1H), 2.94–2.80 (m, 2H), 2.62 (ddd, *J* = 13.0, 10.0, 3.5 Hz, 1H), 2.33 (dd,

J = 13.0, 8.0 Hz, 1H); ^{13}C NMR (125 MHz, $CDCl_3$): δ 170.7, 139.3, 131.5, 128.8, 128.3, 128.1, 127.6, 127.2, 122.9, 84.4, 83.5, 62.9, 54.3, 43.8, 30.9, 23.2; IR (neat): 2935, 1705, 1489, 1441, 1362, 1264, 1044, 959, 756, 691 cm^{-1}; HRMS (m/z) Calcd for ($C_{20}H_{20}NO_2$) ([M+H]$^+$): 306.1489; found: 306.1485.

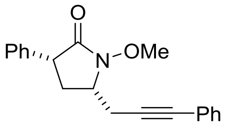

Mnior diastereomer: Yellow oil; R_f = 0.23 (33% EtOAc in hexanes); ^{1}H NMR (500 MHz, $CDCl_3$): δ 7.45–7.38 (m, 2H), 7.37–7.24 (m, 8H), 4.01 (s, 3H), 3.98–3.89 (m, 1H), 3.62 (t, J = 10.0 Hz, 1H), 2.99 (dd, J = 17.0, 6.0 Hz, 1H), 2.87 (dd, J = 17.0, 3.5 Hz, 1H), 2.74–2.64 (m, 1H), 2.26–2.16 (m, 1H); ^{13}C NMR (125 MHz, $CDCl_3$): δ 172.3, 138.9, 131.5, 128.7, 128.3, 128.1, 128.0, 127.2, 122.9, 84.2 (2C), 63.8, 55.2, 45.0, 31.0, 23.5; IR (neat): 2938, 1711, 1490, 1448, 1365, 1267, 1045, 757, 694 cm^{-1}; HRMS (m/z) Calcd for ($C_{20}H_{20}NO_2$) ([M+H]$^+$): 306.1489; found: 306.1487.

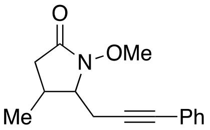

1-Methoxy-4-methyl-5-(3-phenylprop-2-yn-1-yl)pyrrolidin-2-one (3fa). The crude reaction mixture contains two diastereoisomers with d.r. = 1:1 detected by ^{1}H NMR. Purification by column chromatography (25% EtOAc in hexanes) gave **3ea** as a mixture of two separable diastereoisomers (18.9 mg and 18.3 mg, 51% yield; relative stereochemistry was assigned on the basis of ^{1}H NMR,[3] the signals of methyne protons at C-5 in the *trans* isomers were reported to appear at a higher field than those of the *cis* isomers.).

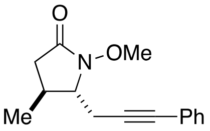

Diastereomer I: Yellow solid; R_f = 0.18 (33% EtOAc in hexanes); ^{1}H NMR (500 MHz, $CDCl_3$): δ 7.43–7.33 (m, 2H), 7.33–7.23 (m, 3H), 3.89 (s, 3H), 3.52–3.42 (m, 1H), 2.88–2.74 (m, 2H), 2.65 (dd, J = 17.0, 9.0 Hz, 1H), 2.45–2.35 (m, 1H), 1.95 (dd, J = 17.0, 7.0 Hz, 1H), 1.21 (d, J = 7.0 Hz, 3H); ^{13}C NMR (125 MHz, $CDCl_3$): δ 170.6, 131.4, 128.2, 128.0, 122.9, 84.4, 83.5, 63.3, 63.0, 35.7, 28.9, 22.2, 19.5; IR (neat): 2961, 2931, 1717, 1489, 1441, 1377, 1290, 1250, 1056, 972, 756, 691 cm^{-1}; HRMS (m/z) Calcd for ($C_{16}H_{18}NO_2$) ([M+H]$^+$): 244.1332; found: 244.1333.

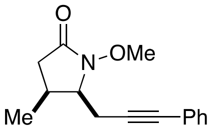

Diastereomer II: Yellow solid; R_f = 0.12 (33% EtOAc in hexanes); ^{1}H NMR (500 MHz, $CDCl_3$): δ 7.43–7.33 (m, 2H), 7.33–7.23 (m, 3H), 4.00–3.90 (m, 1H), 3.85 (s, 3H), 2.83 (dd, J = 17.0, 7.0 Hz, 1H), 2.69 (dd, J = 17.0, 3.0 Hz, 1H), 2.66–2.54 (m, 1H), 2.44 (dd, J = 17.0, 9.0 Hz, 1H), 2.25 (dd, J = 17.0, 8.5 Hz, 1H), 1.27 (d, J = 7.0 Hz, 3H); ^{13}C NMR (125 MHz, $CDCl_3$): δ 171.2, 131.3, 128.2, 128.0, 123.0, 85.0, 83.5, 62.7, 59.0, 35.6, 27.5, 18.7, 14.8; IR (neat): 2967, 2932, 1707, 1489, 1441, 1381, 1261, 1059, 964, 756, 691, 632, 586 cm^{-1}; HRMS (m/z) Calcd for ($C_{16}H_{18}NO_2$) ([M+H]$^+$): 244.1332; found: 244.1334.

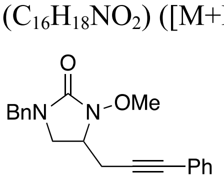

1-Benzyl-3-methoxy-4-(3-phenylprop-2-yn-1-yl)imidazolidin-2-one (3ga). Purification by column chromatography (20% EtOAc in hexanes) gave **3ga** as a yellow oil (72.1 mg, 75% yield); R_f = 0.40 (33% EtOAc in hexanes); ^{1}H NMR (500 MHz, $CDCl_3$): δ 7.45–7.15 (m, 10H), 4.47 (d, J = 15.0 Hz, 1H), 4.37 (d, J = 15.0 Hz, 1H), 3.92 (s, 3H), 3.79–3.69 (m, 1H), 3.31 (t, J = 8.5 Hz, 1H), 3.09 (t, J = 8.5 Hz, 1H), 2.90 (dd,

J = 17.0, 4.0 Hz, 1H), 2.73 (dd, J = 17.0, 7.5 Hz, 1H); ^{13}C NMR (125 MHz, $CDCl_3$): δ 161.9, 135.8, 131.5, 128.7, 128.2, 128.1, 128.0, 127.7, 122.9, 84.2, 83.3, 64.6, 57.4, 47.7, 45.1, 22.1; IR (neat): 2933, 1724, 1489, 1435, 1259, 1030, 754, 691, 631 cm^{-1}; HRMS (m/z) Calcd for ($C_{21}H_{21}N_2O_2$) ($[M+H]^+$): 321.1598; found: 321.1600.

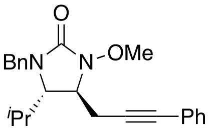

(4*S*,5*S*)-1-Benzyl-5-isopropyl-3-methoxy-4-(3-phenylprop-2-yn-1-yl)imidazolidin-2-one (3ha). Only one diastereomer was observed from the crude ^{1}H NMR. Purification by column chromatography (20% EtOAc in hexanes) gave **3ha** as a yellow oil (84.1 mg, 78% yield); R_f = 0.51 (33% EtOAc in hexanes); ^{1}H NMR (500 MHz, $CDCl_3$): δ 7.40–7.15 (m, 10H), 4.92 (d, J = 15.5 Hz, 1H), 3.96 (d, J = 15.5 Hz, 1H), 3.92 (s, 3H), 3.60 (q, J = 5.0 Hz, 1H), 3.36 (dd, J = 5.0, 3.0 Hz, 1H), 2.72 (d, J = 5.0 Hz, 2H), 2.14–1.98 (m, 1H), 0.93 (d, J = 7.0 Hz, 3H), 0.87 (d, J = 7.0 Hz, 3H); ^{13}C NMR (125 MHz, $CDCl_3$): δ 161.1, 135.9, 131.5, 128.5, 128.1, 128.0, 127.9, 127.4, 123.1, 84.8, 83.5, 64.4, 59.6, 56.2, 45.1, 27.2, 22.7, 18.1, 15.8; IR (neat): 2961, 2933, 2896, 1719, 1490, 1419, 1245, 1029, 755, 691, 631 cm^{-1}; HRMS (m/z) Calcd for ($C_{23}H_{27}N_2O_2$) ($[M+H]^+$): 363.2067; found: 363.2071.

Stereochemistry was assigned on the basis of NOESY.

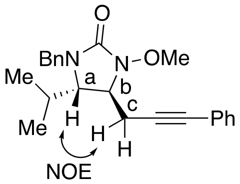

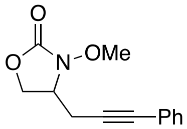

3-Methoxy-4-(3-phenylprop-2-yn-1-yl)oxazolidin-2-one (3ia). Purification by column chromatography (20% EtOAc in hexanes) gave **3ia** as a yellow solid (29.0 mg, 42% yield); R_f = 0.46 (33% EtOAc in hexanes); ^{1}H NMR (500 MHz, $CDCl_3$): δ 7.46–7.36 (m, 2H), 7.36–7.26 (m, 3H), 4.43 (t, J = 8.0 Hz, 1H), 4.25 (t, J = 8.0 Hz, 1H), 4.13–4.05 (m, 1H), 3.89 (s, 3H), 2.89 (dd, J = 17.0, 4.0 Hz, 1H), 2.83 (dd, J = 17.0, 7.0 Hz, 1H); ^{13}C NMR (125 MHz, $CDCl_3$): δ 158.3, 131.5 (2C), 128.33, 128.30 (2C), 122.5, 84.1, 82.5, 65.4, 64.2, 56.8, 21.2; IR (neat): 2974, 2938, 1770, 1661, 1490, 1394, 1209, 1070, 1021, 983, 754, 691 cm^{-1}; HRMS (m/z) Calcd for ($C_{13}H_{14}NO_3$) ($[M+H]^+$): 232.0968; found: 232.0970.

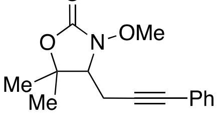

3-Methoxy-5,5-dimethyl-4-(3-phenylprop-2-yn-1-yl)oxazolidin-2-one (3ja). Purification by column chromatography (20% EtOAc in hexanes) gave **3ja** as a yellow solid (53.6 mg, 69% yield); R_f= 0.63 (33% EtOAc in hexanes); ^{1}H NMR (500 MHz, $CDCl_3$): δ 7.43–7.35 (m, 2H), 7.34–7.28 (m, 3H), 3.85 (s, 3H), 3.76–3.68 (m, 1H), 2.95 (dd, J = 17.0, 4.0 Hz, 1H), 2.71 (dd, J = 17.0, 9.5 Hz, 1H), 1.59 (s, 3H), 1.52 (s,

3H); ^{13}C NMR (125 MHz, CDCl$_3$): δ 157.8, 131.4 (2C), 128.3 (3C), 122.6, 83.6, 83.5, 80.6, 65.8, 64.2, 28.0, 21.6, 19.0; IR (neat): 2979, 2937, 1772, 1490, 1441, 1374, 1277, 1072, 755, 691 cm^{-1}; HRMS (m/z) Calcd for ($C_{15}H_{18}NO_3$) ([M+H]$^+$): 260.1281; found: 260.1283.

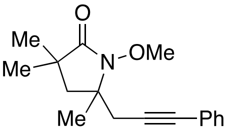

1-Methoxy-3,3,5-trimethyl-5-(3-phenylprop-2-yn-1-yl)pyrrolidin-2-one (3ka). Purification by column chromatography (20% EtOAc in hexanes) gave **3ka** as a yellow oil (56.2 mg, 69% yield); R_f = 0.44 (33% EtOAc in hexanes); ^{1}H NMR (500 MHz, CDCl$_3$): δ 7.45–7.35 (m, 2H), 7.34–7.24 (m, 3H), 3.96 (s, 3H), 2.78 (d, *J* = 17.0 Hz, 1H), 2.65 (d, *J* = 17.0 Hz, 1H), 2.27 (d, *J* = 13.5 Hz, 1H), 1.89 (dd, *J* = 13.5 Hz, 1H), 1.43 (s, 3H), 1.26 (s, 3H); ^{13}C NMR (125 MHz, CDCl$_3$): δ 175.1, 131.4, 128.2, 128.0, 123.0, 85.6, 83.6, 64.0, 60.7, 43.6, 36.4, 30.7, 27.7, 27.0, 25.7; IR (neat): 2968, 2938, 2869, 1703, 1489, 1442, 1385, 1016, 756, 691 cm^{-1}; HRMS (m/z) Calcd for ($C_{17}H_{22}NO_2$) ([M+H]$^+$): 272.1645; found: 272.1642.

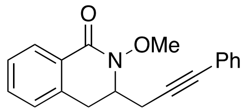

2-Methoxy-3-(3-phenylprop-2-yn-1-yl)-3,4-dihydroisoquinolin-1(2*H*)-one (3la). Purification by column chromatography (20% EtOAc in hexanes) gave **3la** as a yellow oil (66.3 mg, 76% yield); R_f = 0.48 (33% EtOAc in hexanes); ^{1}H NMR (400 MHz, CDCl$_3$): δ 8.12 (d, *J* = 8.0 Hz, 1H), 7.50–7.20 (m, 8H), 4.24–4.10 (m, 1H), 3.96 (s, 3H), 3.52–3.32 (m, 2H), 2.97 (dd, *J* = 16.8, 3.6 Hz, 1H), 2.68 (dd, *J* = 16.8, 9.2 Hz, 1H); ^{13}C NMR (100 MHz, CDCl$_3$): δ 163.4, 135.3, 132.5, 131.5 (2C), 128.2 (2C), 128.1 (2C), 128.0, 127.9, 127.1, 123.0, 85.3, 83.4, 63.1, 57.9, 32.7, 21.8; IR (neat): 3054, 2935, 1669, 1264, 1242, 1021, 998, 730, 689 cm^{-1}; HRMS (m/z) Calcd for ($C_{19}H_{18}NO_2$) ([M+H]$^+$): 292.1332; found: 292.1335.

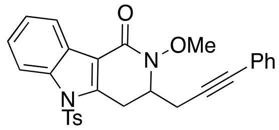

2-Methoxy-3-(3-phenylprop-2-yn-1-yl)-5-tosyl-2,3,4,5-tetrahydro-1*H*-pyrido[4,3-*b*]indol-1-one (3ma). Purification by column chromatography (20% EtOAc in hexanes) gave **3ma** as a yellow oil (66.8 mg, 46% yield); R_f = 0.45 (33% EtOAc in hexanes); ^{1}H NMR (500 MHz, CDCl$_3$): δ 8.28–8.19 (m, 1H), 8.19–8.11 (m, 1H), 7.83 (d, *J* = 8.5 Hz, 2H), 7.45–7.25 (m, 7H), 7.14 (d, *J* = 8.5 Hz, 2H), 4.32–4.22 (m, 1H), 4.12 (dd, *J* = 18.0, 4.0 Hz, 1H), 3.95 (s, 3H), 3.74 (dd, *J* = 18.0, 6.0 Hz, 1H), 3.03 (dd, *J* = 17.0, 3.5 Hz, 1H), 2.76 (dd, *J* = 17.0, 9.5 Hz, 1H), 2.29 (s, 3H); ^{13}C NMR (125 MHz, CDCl$_3$): δ 163.5, 145.7, 140.2, 136.2, 134.9, 131.4, 130.1, 128.2, 128.1, 126.5, 126.1, 125.1, 124.7, 122.8, 121.3, 113.8, 111.8, 85.1, 84.1, 63.4, 58.3, 28.5, 21.5, 21.3; IR (neat): 3054, 2934, 1675, 1449, 1419, 1375, 1264, 1172, 1037, 731, 684, 659, 569, 539 cm^{-1}; HRMS (m/z) Calcd for ($C_{28}H_{25}N_2O_4S$) ([M+H]$^+$): 485.1530; found: 485.1534.

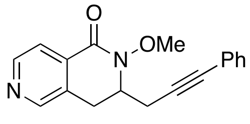

2-Methoxy-3-(3-phenylprop-2-yn-1-yl)-3,4-dihydro-2,6-naphthyridin-1(2*H*)-one (3na). Purification by column chromatography (75% EtOAc in hexanes) gave **3na** as a yellow oil (35.0 mg, 40% yield); R_f = 0.16 (67% EtOAc in hexanes); ^{1}H NMR (500 MHz, $CDCl_3$): δ 8.77–7.50 (m, 2H), 8.04–7.84 (m, 1H), 7.45–7.15 (m, 5H), 4.35–4.20 (m, 1H), 3.97 (s, 3H), 3.55–3.35 (m, 2H), 2.95 (dd, *J* = 17.0, 3.5 Hz, 1H), 2.72 (dd, *J* = 17.0, 9.0 Hz, 1H); ^{13}C NMR (125 MHz, $CDCl_3$): δ 161.2, 149.3, 148.9, 135.2, 131.4 (2C), 129.3, 128.2 (3C), 122.6, 120.5, 84.5, 84.3, 63.1, 57.7, 29.8, 22.3; IR (neat): 2934, 1673, 1568, 1421, 1308, 1244, 1019, 999, 756, 691 cm^{-1}; HRMS (m/z) Calcd for ($C_{18}H_{17}N_2O_2$) ([M+H]$^+$): 293.1285; found: 293.1287.

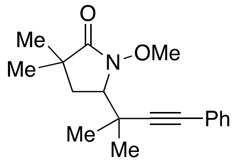

1-Methoxy-3,3-dimethyl-5-(2-methyl-4-phenylbut-3-yn-2-yl)pyrrolidin-2-one (3oa). Purification by column chromatography (25% EtOAc in hexanes) gave **3oa** as a yellow oil (70.5 mg, 82% yield); R_f = 0.45 (33% EtOAc in hexanes); ^{1}H NMR (500 MHz, $CDCl_3$): δ 7.40–7.30 (m, 2H), 7.30–7.20 (m, 3H), 3.84 (s, 3H), 3.61 (t, *J* = 8.5 Hz, 1H), 1.92 (dd, *J* = 13.0, 8.0 Hz, 1H), 1.79 (dd, *J* = 13.0, 9.0 Hz, 1H), 1.43 (s, 3H), 1.28 (s, 3H), 1.19 (s, 3H), 1.11 (s, 3H); ^{13}C NMR (125 MHz, $CDCl_3$): δ 177.0, 131.3, 128.1, 127.7, 123.1, 93.2, 82.6, 62.1, 60.9, 36.5, 35.7, 35.3, 26.3, 26.1, 25.3, 24.9; IR (neat): 2967, 2930, 2868, 1707, 1458, 1442, 1386, 1363, 1271, 1248, 1021, 929, 755, 691 cm^{-1}; HRMS (m/z) Calcd for ($C_{18}H_{24}NO_2$) ([M+H]$^+$): 286.1802; found: 286.1801.

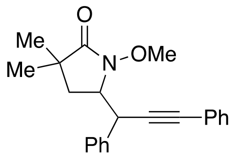

5-(1,3-Diphenylprop-2-yn-1-yl)-1-methoxy-3,3-dimethylpyrrolidin-2-one (3pa). The crude reaction mixture contains two diastereoisomers with d.r. = 2:1 detected by ^{1}H NMR. Purification by column chromatography (33% EtOAc in hexanes) gave **3pa** as a mixture of two separable diastereoisomers (50.0 mg major and 20.5 mg minor from ***(E)*-1p**, 71% yield; 48.0 mg major and 22.2 mg minor from ***(Z)*-1p**, 70% yield). The relative stereochemistry was assigned by X-Ray (See section XI).

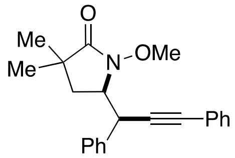

Major diastereomer: Yellow solid; R_f = 0.42 (33% EtOAc in hexanes); ^{1}H NMR (500 MHz, $CDCl_3$): δ 7.51–7.41 (m, 4H), 7.38 (t, *J* = 7.5 Hz, 2H), 7.36–7.27 (m, 4H), 4.64 (d, *J* = 4.0 Hz, 1H), 4.04 (s, 3H), 3.96–3.86 (m, 1H), 2.05 (dd, *J* = 13.0, 8.5 Hz, 1H), 1.55 (dd, *J* = 13.0, 7.0 Hz, 1H), 1.25 (s, 3H), 1.07 (s, 3H); ^{13}C NMR (125 MHz, $CDCl_3$): δ 176.5, 136.5, 131.6, 128.7, 128.3, 128.0, 127.5, 122.7, 87.2, 86.0, 63.5, 60.5, 40.2, 37.3, 33.6, 25.7, 25.2; IR (neat): 3059, 2963, 1710, 1286, 1030, 1017, 755, 691 cm^{-1}; HRMS (m/z) Calcd for ($C_{22}H_{24}NO_2$) ([M+H]$^+$): 334.1802; found: 334.1800.

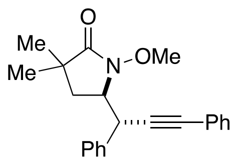

Minor diastereomer: Yellow solid; R_f = 0.26 (33% EtOAc in hexanes); ^{1}H NMR (500 MHz, $CDCl_3$): δ 7.55–7.42 (m, 4H), 7.42–7.28 (m, 6H), 4.23 (d, *J* = 3.5 Hz, 1H), 4.40–4.30 (m, 1H), 3.85 (s, 3H), 1.95 (dd, *J* = 13.5, 8.5 Hz, 1H), 1.71 (dd, *J* = 13.5, 6.0 Hz, 1H), 1.10 (s, 3H), 0.55 (s, 3H); ^{13}C NMR (125 MHz, $CDCl_3$): δ 174.7, 135.1,

131.6, 129.1, 128.4, 128.3, 128.2, 127.6, 122.9, 87.3, 85.1, 61.2, 57.3, 39.4, 36.5, 33.5, 26.7, 24.6; IR (neat): 3059, 2965, 1694, 1449, 1228, 1028, 1014, 757, 698 cm^{-1}; HRMS (m/z) Calcd for ($C_{22}H_{24}NO_2$) ($[M+H]^+$): 334.1802; found: 334.1808.

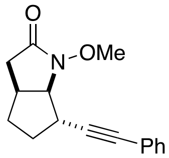

1-Methoxy-6-(phenylethynyl)hexahydrocyclopenta[*b*]pyrrol-2(1*H*)-one (3qa). Only one diastereomer was observed from the crude ^{1}H NMR. Purification by column chromatography (20% EtOAc in hexanes) gave **3qa** as a yellow oil (45.3 mg, 60% yield); R_f = 0.13 (33% EtOAc in hexanes); ^{1}H NMR (400 MHz, $CDCl_3$): δ 7.40–7.30 (m, 2H), 7.30–7.20 (m, 3H), 4.23 (d, *J* = 7.6 Hz, 1H), 3.78 (s, 3H), 3.24–3.14 (m, 1H), 2.89–2.74 (m, 1H), 2.57 (dd, *J* = 17.6, 10.4 Hz, 1H), 2.30–2.10 (m, 1H), 1.98 (dd, *J* = 17.6, 7.2 Hz, 1H), 1.95–1.77 (m, 2H), 1.57–1.41 (m, 1H); ^{13}C NMR (100 MHz, $CDCl_3$): δ 168.8, 131.3, 128.0, 127.8, 122.9, 89.8, 82.2, 66.9, 61.8, 35.1, 34.4, 32.9, 31.7, 30.4; IR (neat): 2925, 2868, 1700, 1489, 1442, 1395, 1267, 1057, 961, 756, 691 cm^{-1}; HRMS (m/z) Calcd for ($C_{16}H_{18}NO_2$) ($[M+H]^+$): 256.1332; found: 256.1333.

The relative stereochemistry was assigned on the basis of NOESY.

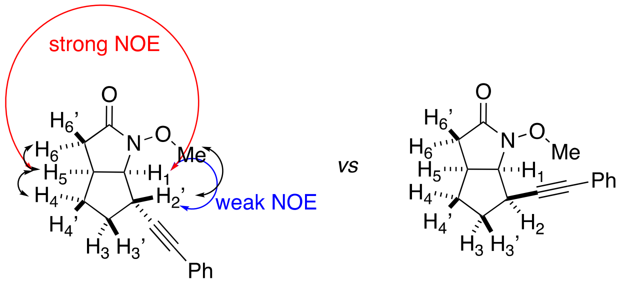

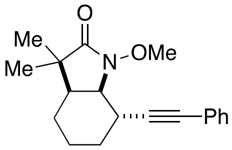

1-Methoxy-3,3-dimethyl-7-(phenylethynyl)octahydro-2*H*-indol-2-one (3ra). Only one diastereomer was observed from the crude ^{1}H NMR. Purification by column chromatography (25% EtOAc in hexanes) gave **3ra** as a yellow oil (82.1 mg, 92% yield); R_f = 0.56 (33% EtOAc in hexanes); ^{1}H NMR (500 MHz, $CDCl_3$): δ 7.50–7.35 (m, 2H), 7.35–7.20 (m, 3H), 3.93–3.86 (m, 1H), 3.82 (s, 3H), 3.42–3.32 (m, 1H), 2.14–2.02 (m, 1H), 1.90–1.50 (m, 5H), 1.35–1.20 (m, 1H), 1.12 (s, 3H), 1.10 (s, 3H); ^{13}C NMR (125 MHz, $CDCl_3$): δ 177.8, 131.5, 128.1, 127.8, 123.2, 90.0, 82.4, 62.3, 58.0, 42.5, 38.3, 27.8, 25.6, 24.5, 23.4, 19.7, 18.6; IR (neat): 2934, 2861, 1714, 1489, 1442, 1386, 1233, 1023, 756, 691 cm^{-1}; HRMS (m/z) Calcd for ($C_{19}H_{24}NO_2$) ($[M+H]^+$): 298.1802; found: 298.1803.

The relative stereochemistry was assigned on the basis of NOESY.

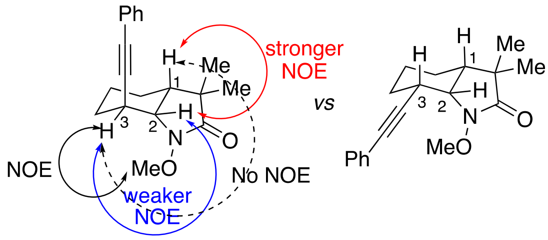

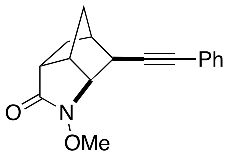

1-Methoxy-6-(phenylethynyl)hexahydro-3,5-methanocyclopenta[*b*]pyrrol-2(1*H*)-one (3sa). Only one diastereomer was observed from the crude ^{1}H NMR. Purification by column chromatography (33% EtOAc in hexanes) gave **3sa** as a yellow oil (51.9 mg, 65% yield); R_f = 0.22 (33% EtOAc in hexanes); ^{1}H NMR (500 MHz, $CDCl_3$): δ 7.42–7.32 (m, 2H), 7.32–7.20 (m, 3H), 3.93 (d, *J* = 4.5 Hz, 1H), 3.78 (s, 3H), 2.96–2.86 (m, 1H), 2.67 (s, 1H), 2.57 (s, 1H), 2.41 (dd, *J* = 11.0, 4.0 Hz, 1H), 2.09 (d, *J* = 11.0 Hz, 1H), 2.00–1.90 (m, 1H), 1.65 (d, *J* = 13.0 Hz, 1H), 1.55 (d, *J* = 10.5 Hz, 1H); ^{13}C NMR (125 MHz, $CDCl_3$): δ 176.3, 131.3, 128.1, 127.8, 123.0, 90.1, 82.7, 65.0, 62.7, 43.9, 40.4, 38.9, 37.3, 35.0, 34.7; IR (neat): 2965, 2874, 1720, 1489, 1441, 1227, 1058, 1002, 756, 691 cm^{-1}; HRMS (m/z) Calcd for ($C_{17}H_{18}NO_2$) ($[M+H]^+$): 268.1332; found: 268.1329.

The relative stereochemistry was assigned on the basis of NOESY.

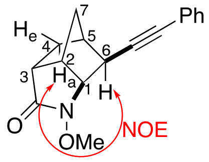

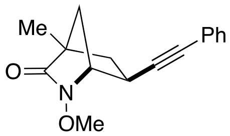

2-Methoxy-4-methyl-6-(phenylethynyl)-2-azabicyclo[2.2.1]heptan-3-one (3ta). The crude reaction mixture contains two diastereoisomers with d.r. = 5:1 detected by ^{1}H NMR. Purification by column chromatography (33% EtOAc in hexanes) gave **3ta** as a mixture of two separable diastereoisomers (29.8 mg major and 6.8 mg minor, 48% yield).

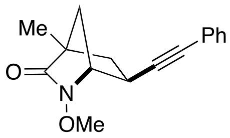

Major diastereomer: Yellow solid; R_f = 0.56 (33% EtOAc in hexanes); ^{1}H NMR (500 MHz, $CDCl_3$): δ 7.45–7.35 (m, 2H), 7.35–7.25 (m, 3H), 4.05 (s, 1H), 3.81 (s, 3H), 3.26–3.16 (m, 1H), 2.08–1.97 (m, 1H), 1.93 (d, *J* = 10.0 Hz, 1H), 1.82 (dd, *J* = 12.5, 5.0 Hz, 1H), 1.70 (d, *J* = 10.0 Hz, 1H), 1.33 (s, 3H); ^{13}C NMR (125 MHz, $CDCl_3$): δ 179.3, 131.5, 128.2, 128.0, 123.0, 90.2, 81.9, 64.2, 63.4, 50.0, 41.8, 38.2, 30.5, 14.1; IR (neat): 2963, 2929, 1727, 1490, 1440, 1241, 1212, 1026, 763, 752, 694 cm^{-1}; HRMS (m/z) Calcd for ($C_{16}H_{18}NO_2$) ($[M+H]^+$): 256.1332; found: 256.1328.

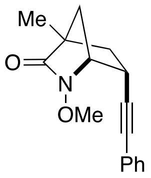

Minor diastereomer: Yellow solid; R_f = 0.28 (33% EtOAc in hexanes); ^{1}H NMR (500 MHz, $CDCl_3$): δ 7.43–7.33 (m, 2H), 7.33–7.22 (m, 3H), 4.20 (s, 1H), 3.84 (s, 3H), 3.35–3.25 (m, 1H), 2.18–2.07 (m, 1H), 1.81–1.71 (m, 1H), 1.71 (d, *J* = 9.5 Hz, 1H), 1.52 (d, *J* = 9.5 Hz, 1H), 1.33 (s, 3H); ^{13}C NMR (125 MHz, $CDCl_3$): δ 175.6, 131.5, 128.0, 127.7, 123.4, 89.2, 82.4, 63.6, 62.9, 50.0, 42.8, 40.4, 33.7, 14.7; IR (neat): 2958, 2945, 2868, 1719, 1490, 1441, 1363, 1254, 1021, 755, 748, 690 cm^{-1}; HRMS (m/z) Calcd for ($C_{16}H_{18}NO_2$) ($[M+H]^+$): 256.1332; found: 256.1336.

Stereochemistry was assigned on the basis of NOESY.

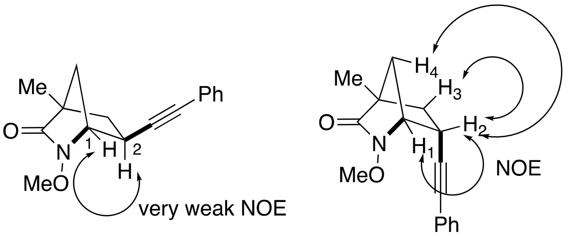

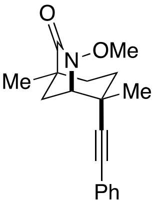

6-Methoxy-1,4-dimethyl-4-(phenylethynyl)-6-azabicyclo[3.2.1]octan-7-one (3ua). The crude reaction mixture contains two diastereoisomers with d.r. = 2:1 detected by ^{1}H NMR. Purification by column chromatography (33% EtOAc in hexanes) gave **3ua** as a mixture of two separable diastereoisomers (43.4 mg major and 21.2 mg minor, 76% yield).

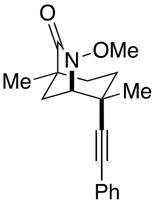

Major diastereomer: Yellow oil; R_f = 0.49 (33% EtOAc in hexanes); ^{1}H NMR (500 MHz, $CDCl_3$): δ 7.44–7.34 (m, 2H), 7.34–7.23 (m, 3H), 3.79 (d, J = 5.5 Hz, 1H), 3.77 (s, 3H), 2.39 (d, J = 11.0 Hz, 1H), 1.97–1.87 (m, 1H), 1.87–1.76 (m, 1H), 1.71-1.53 (m, 3H), 1.46 (s, 3H), 1.11 (s, 3H); ^{13}C NMR (125 MHz, $CDCl_3$): δ 174.3, 131.3, 128.1, 127.8, 123.0, 93.9, 82.7, 62.6, 61.2, 40.8, 38.8, 34.7, 33.7, 31.8, 27.1, 20.1; IR (neat): 2933, 2868, 1716, 1489, 1453, 1377, 1021, 754, 691 cm^{-1}; HRMS (m/z) Calcd for ($C_{18}H_{22}NO_2$) ($[M+H]^+$): 284.1645; found: 284.1648.

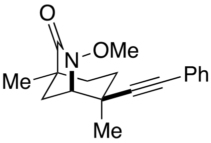

Minor diastereomer: Yellow oil; R_f = 0.32 (33% EtOAc in hexanes); ^{1}H NMR (500 MHz, $CDCl_3$): δ 7.42–7.32 (m, 2H), 7.31–7.20 (m, 3H), 3.90 (d, J = 5.0 Hz, 1H), 3.83 (s, 3H), 1.97 (td, J = 14.0, 6.0 Hz, 1H), 1.91 (d, J = 11.5 Hz, 1H), 1.79 (dd, J = 15.0, 5.5 Hz, 1H), 1.77–1.69 (m, 1H), 1.62 (dd, J = 13.0, 5.5 Hz, 1H), 1.46 (dd, J = 13.0, 5.5 Hz, 1H), 1.42 (s, 3H), 1.12 (s, 3H); ^{13}C NMR (125 MHz, $CDCl_3$): δ 174.5, 131.3, 128.0, 127.6, 123.6, 95.3, 81.1, 62.0, 61.8, 40.8, 35.2, 32.0, 31.3, 30.5, 24.4, 20.3; IR (neat): 2930, 2870, 1714, 1489, 1442, 1378, 1258, 1229, 1026, 756, 691 cm^{-1}; HRMS (m/z) Calcd for ($C_{18}H_{22}NO_2$) ($[M+H]^+$): 284.1645; found: 284.1646.

Stereochemistry was assigned on the basis of NOESY.

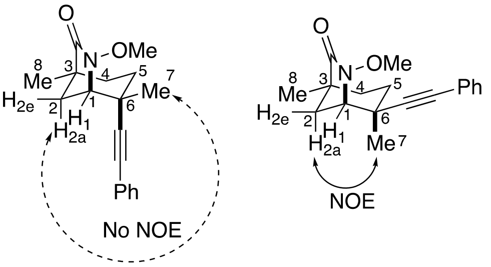

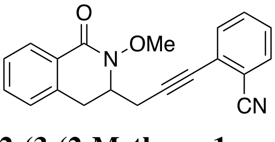

2-(3-(2-Methoxy-1-oxo-1,2,3,4-tetrahydroisoquinolin-3-yl)prop-1-yn-1-yl)benzonitrile (3lb). Purification by column chromatography (20% EtOAc in hexanes) gave **3lb** as a yellow oil (67.3 mg, 71% yield); R_f = 0.32 (33% EtOAc in hexanes); ^{1}H NMR (400 MHz, $CDCl_3$): δ 8.09 (d, J = 7.5 Hz, 1H), 7.65–

7.57 (m, 1H), 7.54–7.40 (m, 3H), 7.40–7.25 (m, 3H), 4.27–4.25 (m, 1H), 3.93 (s, 3H), 3.57–3.40 (m, 2H), 3.05 (dd, J = 16.8, 3.2 Hz, 1H), 2.68 (dd, J = 16.8, 9.6 Hz, 1H); ^{13}C NMR (100 MHz, $CDCl_3$): δ 163.3, 135.2, 132.6, 132.4, 132.3, 132.1, 128.2, 128.1, 127.99, 127.95, 127.1, 127.0, 117.7, 115.3, 92.7, 79.7, 63.0, 57.7, 32.4, 21.7; IR (neat): 2933, 2226, 1668, 1481, 1459, 1424, 1278, 1241, 1020, 996, 762, 740, 723, 685 cm^{-1}; HRMS (m/z) Calcd for ($C_{20}H_{17}N_2O_2$) ($[M+H]^+$): 317.1285; found: 317.1291.

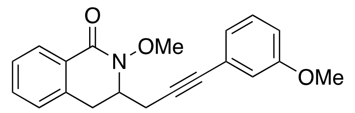

2-Methoxy-3-(3-(3-methoxyphenyl)prop-2-yn-1-yl)-3,4-dihydroisoquinolin-1(2*H*)-one (3lc). Purification by column chromatography (20% EtOAc in hexanes) gave **3lc** as a yellow oil (72.2 mg, 75% yield); R_f = 0.40 (33% EtOAc in hexanes); ^{1}H NMR (400 MHz, $CDCl_3$): δ 7.95 (d, J = 8.0 Hz, 1H), 7.29 (t, J = 7.5 Hz, 1H), 7.17 (t, J = 7.5 Hz, 1H), 7.06 (d, J = 7.5 Hz, 1H), 7.01 (t, J = 7.5 Hz, 1H), 6.78 (d, J = 7.5 Hz, 1H), 6.73–6.63 (m, 2H), 4.07–3.97 (m, 1H), 3.78 (s, 3H), 3.60 (s, 3H), 3.35–3.15 (m, 2H), 2.80 (d, J = 16.8 Hz, 1H), 2.50 (dd, J = 16.8, 9.6 Hz, 1H); ^{13}C NMR (100 MHz, $CDCl_3$): δ 163.3, 159.1, 135.2, 132.5, 129.2, 128.0, 127.9, 127.1, 124.0, 116.4, 114.4, 85.1, 83.3, 63.0, 57.9, 55.1, 32.6, 21.8; IR (neat): 2934, 2833, 1670, 1603, 1573, 1480, 1459, 1423, 1286, 1250, 1201, 1163, 1040, 1022, 999, 854, 782, 740, 685 cm^{-1}; HRMS (m/z) Calcd for ($C_{20}H_{20}NO_3$) ($[M+H]^+$): 322.1438; found: 322.1438.

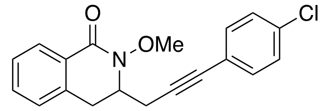

3-(3-(4-Chlorophenyl)prop-2-yn-1-yl)-2-methoxy-3,4-dihydroisoquinolin-1(2*H*)-one (3ld). Purification by column chromatography (20% EtOAc in hexanes) gave **3ld** as a yellow oil (71.2 mg, 73% yield); R_f = 0.40 (33% EtOAc in hexanes); ^{1}H NMR (400 MHz, $CDCl_3$): δ 8.10 (d, J = 8.0 Hz, 1H), 7.45 (t, J = 7.5 Hz, 1H), 7.33 (t, J = 7.5 Hz, 1H), 7.30–7.15 (m, 5H), 4.23–4.07 (m, 1H), 3.93 (s, 3H), 3.43 (dd, J = 16.4, 5.6 Hz, 1H), 3.33 (dd, J = 16.4, 4.4 Hz, 1H), 2.94 (dd, J = 16.8, 3.2 Hz, 1H), 2.68 (dd, J = 16.8, 9.2 Hz, 1H); ^{13}C NMR (100 MHz, $CDCl_3$): δ 163.4, 135.2, 134.0, 132.7 (2C), 132.5, 128.5 (2C), 128.1 (2C), 127.9, 127.1, 121.5, 86.4, 82.4, 63.0, 57.8, 32.8, 21.9; IR (neat): 2933, 1667, 1604, 1488, 1459, 1423, 1396, 1276, 1242, 1088, 1013, 826, 737, 720, 688 cm^{-1}; HRMS (m/z) Calcd for ($C_{19}H_{17}ClNO_2$) ($[M+H]^+$): 326.0942; found: 326.0941.

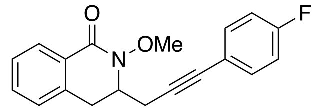

3-(3-(4-Fluorophenyl)prop-2-yn-1-yl)-2-methoxy-3,4-dihydroisoquinolin-1(2*H*)-one (3le). Purification by column chromatography (25% EtOAc in hexanes) gave **3le** as a yellow oil (63.0 mg, 68% yield); R_f = 0.40 (33% EtOAc in hexanes); ^{1}H NMR (500 MHz, $CDCl_3$): δ 8.12 (d, J = 7.5 Hz, 1H), 7.47 (t, J = 7.5 Hz, 1H), 7.35 (t, J = 7.5 Hz, 1H), 7.32 (d, J = 8.0 Hz, 1H), 7.31 (d, J = 8.0 Hz, 1H), 7.24 (d, J = 7.5 Hz, 1H), 6.97 (t, J = 8.5 Hz, 2H), 4.23–4.13 (m, 1H), 3.96 (s, 3H), 3.45 (dd, J = 16.0, 6.0 Hz, 1H), 3.37 (dd, J = 16.0, 4.0 Hz, 1H), 2.94 (dd, J = 17.0, 3.0 Hz, 1H), 2.69 (dd, J = 17.0, 9.0 Hz, 1H); ^{13}C NMR (125 MHz, $CDCl_3$): δ 163.4, 162.2 (d, J = 246.2 Hz, 1C), 135.3, 133.3 (d, J = 8.8 Hz, 2C), 132.5, 128.1, 128.0, 127.9, 127.1, 119.1 (d, J = 2.5 Hz, 1C), 115.4 (d, J = 22.5 Hz, 2C), 85.0, 82.4, 63.0, 57.9, 32.7, 21.8; IR (neat): 2928, 1670,

1600, 1505, 1459, 1425, 1276, 1218, 1154, 1092, 1021, 997, 834, 725, 689 cm^{-1}; HRMS (m/z) Calcd for ($C_{19}H_{17}FNO_2$) ($[M+H]^+$): 310.1238; found: 310.1236.

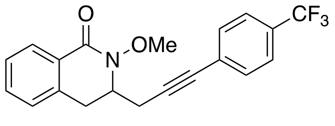

2-Methoxy-3-(3-(4-(trifluoromethyl)phenyl)prop-2-yn-1-yl)-3,4-dihydroisoquinolin-1(2*H*)-one (3lf). Purification by column chromatography (25% EtOAc in hexanes) gave **3lf** as a yellow oil (81.6 mg, 76% yield); R_f= 0.40 (33% EtOAc in hexanes); ^{1}H NMR (500 MHz, $CDCl_3$): δ 8.13 (d, *J* = 8.0 Hz, 1H), 7.54 (d, *J* = 8.0 Hz, 2H), 7.48 (t, *J* = 7.0 Hz, 1H), 7.43 (d, *J* = 8.0 Hz, 2H), 7.36 (t, *J* = 7.5 Hz, 1H), 7.25 (d, *J* = 7.5 Hz, 1H), 4.25–4.17 (m, 1H), 3.97 (s, 3H), 3.48 (dd, *J* = 16.5, 6.0 Hz, 1H), 3.36 (dd, *J* = 16.5, 4.0 Hz, 1H), 2.98 (dd, *J* = 17.0, 3.5 Hz, 1H), 2.74 (dd, *J* = 17.0, 9.5 Hz, 1H); ^{13}C NMR (125 MHz, $CDCl_3$, one carbon missing): δ 163.4, 135.2, 132.6, 131.7 (2C), 129.7 (q, *J* = 32.5 Hz, 1C), 128.1, 127.9, 127.2, 126.8, 125.1 (q, *J* = 2.5 Hz, 2C), 123.8 (q, *J* = 270.0 Hz, 1C), 88.1, 82.3, 63.0, 57.7, 32.8, 22.0; IR (neat): 2935, 1672, 1613, 1459, 1403, 1320, 1163, 1119, 1104, 1065, 1016, 840, 730, 688 cm^{-1}; HRMS (m/z) Calcd for ($C_{20}H_{17}F_3NO_2$) ($[M+H]^+$): 360.1206; found: 360.1201.

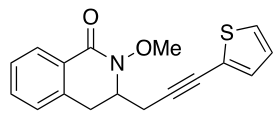

2-Methoxy-3-(3-(thiophen-2-yl)prop-2-yn-1-yl)-3,4-dihydroisoquinolin-1(2*H*)-one (3lg). Purification by column chromatography (20% EtOAc in hexanes) gave **3lg** as a yellow oil (65.4 mg, 73% yield); R_f= 0.46 (33% EtOAc in hexanes); ^{1}H NMR (500 MHz, $CDCl_3$): δ 8.14 (d, *J* = 8.0 Hz, 1H), 7.48 (t, *J* = 7.5 Hz, 1H), 7.37 (t, *J* = 7.5 Hz, 1H), 7.26 (d, *J* = 8.0 Hz, 1H), 7.21 (d, *J* = 4.5 Hz, 1H), 7.15–7.10 (m, 1H), 7.00–6.90 (m, 1H), 4.25–4.15 (m, 1H), 3.96 (s, 3H), 3.45 (dd, *J* = 16.5, 5.5 Hz, 1H), 3.37 (dd, *J* = 16.5, 4.5 Hz, 1H), 3.00 (d, *J* = 16.5, 1H), 2.70 (dd, *J* = 16.5, 9.5 Hz, 1H); ^{13}C NMR (125 MHz, $CDCl_3$): δ 163.4, 135.2, 132.6, 131.6, 128.1, 128.09, 128.02, 127.2, 126.8, 126.5, 123.0, 89.4, 76.6, 63.1, 57.9, 32.7, 22.1; IR (neat): 2932, 2897, 1668, 1605, 1459, 1424, 1298, 1238, 1017, 997, 738, 690 cm^{-1}; HRMS (m/z) Calcd for ($C_{17}H_{16}NO_2S$) ($[M+H]^+$): 298.0896; found: 298.0898.

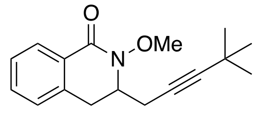

3-(4,4-Dimethylpent-2-yn-1-yl)-2-methoxy-3,4-dihydroisoquinolin-1(2*H*)-one (3lh). Purification by column chromatography (25% EtOAc in hexanes) gave **3lh** as a yellow oil (38.8 mg, 48% yield); R_f= 0.60 (33% EtOAc in hexanes); ^{1}H NMR (500 MHz, $CDCl_3$): δ 8.07 (d, *J* = 7.5 Hz, 1H), 7.42 (t, *J* = 7.5 Hz, 1H), 7.31 (t, *J* = 7.5 Hz, 1H), 7.18 (d, *J* = 7.5 Hz, 1H), 4.05–3.95 (m, 1H), 3.89 (s, 3H), 3.32 (dd, *J* = 16.5, 6.0 Hz, 1H), 3.27 (dd, *J* = 16.5, 4.5 Hz, 1H), 2.65 (dd, *J* = 16.5, 3.0 Hz, 1H), 2.40 (dd, *J* = 16.5, 9.0 Hz, 1H), 1.12 (s, 9H); ^{13}C NMR (125 MHz, $CDCl_3$): δ 163.3, 135.5, 132.3, 128.1, 127.9, 127.8, 126.9, 92.1, 73.7, 62.9, 58.0, 32.4, 30.9, 27.2, 21.1; IR (neat): 2966, 1672, 1606, 1459, 1427, 1263, 1241, 1012, 740, 726, 690 cm^{-1}; HRMS (m/z) Calcd for ($C_{17}H_{22}NO_2$) ($[M+H]^+$): 272.1645; found: 272.1647.

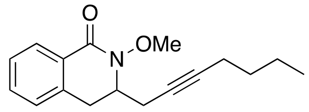

3-(Hept-2-yn-1-yl)-2-methoxy-3,4-dihydroisoquinolin-1(2*H*)-one (3li). Purification by column chromatography (25% EtOAc in hexanes) gave **3li** as a yellow oil (30.3 mg, 37% yield); R_f = 0.60 (33% EtOAc in hexanes); ^{1}H NMR (500 MHz, $CDCl_3$): δ 8.10 (d, *J* = 8.0 Hz, 1H), 7.45 (t, *J* = 8.0 Hz, 1H), 7.34 (t, *J* = 7.5 Hz, 1H), 7.21 (d, *J* = 7.5 Hz, 1H), 4.07–3.97 (m, 1H), 3.91 (s, 3H), 3.41–3.25 (m, 2H), 2.71 (d, *J* = 16.5 Hz, 1H), 2.39 (dd, *J* = 16.5, 9.5 Hz, 1H), 2.11 (t, *J* = 7.0 Hz, 2H), 1.50–1.30 (m, 4H), 0.90 (t, *J* = 7.0 Hz, 3H); ^{13}C NMR (125 MHz, $CDCl_3$): δ 163.4, 135.5, 132.4, 128.1, 128.0, 127.9, 127.0, 83.6, 75.4, 63.0, 58.2, 32.6, 30.9, 21.9, 21.2, 18.3, 13.5; IR (neat): 2930, 2870, 1672, 1605, 1459, 1428, 1273, 1241, 1014, 998, 737, 688 cm^{-1}; HRMS (m/z) Calcd for ($C_{17}H_{22}NO_2$) ([M+H]$^+$): 272.1645; found: 272.1646.

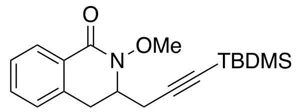

3-(3-(*Tert*-butyldimethylsilyl)prop-2-yn-1-yl)-2-methoxy-3,4-dihydroisoquinolin-1(2*H*)-one (3lj). Purification by column chromatography (10% EtOAc in hexanes) gave **3lj** as a yellow oil (45.8 mg, 46% yield); R_f = 0.68 (33% EtOAc in hexanes); ^{1}H NMR (500 MHz, $CDCl_3$): δ 8.10 (d, *J* = 7.5 Hz, 1H), 7.45 (t, *J* = 7.5 Hz, 1H), 7.34 (t, *J* = 7.5 Hz, 1H), 7.20 (d, *J* = 7.5 Hz, 1H), 4.16–4.02 (m, 1H), 3.91 (s, 3H), 3.49–3.28 (m, 2H), 2.79 (dd, *J* = 17.0, 3.0 Hz, 1H), 2.45 (dd, *J* = 17.0, 10.0 Hz, 1H), 0.91 (s, 9H), 0.06 (s, 6H); ^{13}C NMR (125 MHz, $CDCl_3$): δ 163.4, 135.2, 132.5, 128.1, 128.0, 127.9, 127.1, 102.7, 86.2, 63.0, 57.9, 32.4, 26.0, 22.1, 16.4, 4.6; IR (neat): 2927, 2855, 2174, 1678, 1606, 1460, 1426, 1248, 1015, 823, 773, 680 cm^{-1}; HRMS (m/z) Calcd for ($C_{19}H_{28}NO_2Si$) ([M+H]$^+$): 330.1884; found: 330.1886.

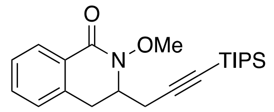

2-Methoxy-3-(3-(triisopropylsilyl)prop-2-yn-1-yl)-3,4-dihydroisoquinolin-1(2*H*)-one (3lk). Purification by column chromatography (10% EtOAc in hexanes) gave **3lk** as a white solid (40.1 mg, 36% yield); R_f = 0.48 (25% EtOAc in hexanes); ^{1}H NMR (500 MHz, $CDCl_3$): δ 8.11 (d, *J* = 7.5 Hz, 1H), 7.45 (t, *J* = 7.5 Hz, 1H), 7.35 (t, *J* = 7.5 Hz, 1H), 7.19 (d, *J* = 7.5 Hz, 1H), 4.16–4.02 (m, 1H), 3.92 (s, 3H), 3.47–3.31 (m, 2H), 2.84 (d, *J* = 17.0 Hz, 1H), 2.45 (dd, *J* = 17.0, 10.0 Hz, 1H), 1.05 (s, 21H); ^{13}C NMR (125 MHz, $CDCl_3$): δ 163.4, 135.3, 132.5, 128.18, 128.11, 127.9, 127.1, 103.8, 84.1, 63.1, 58.2, 32.4, 22.2, 18.6, 11.2; IR (neat): 2940, 2862, 2171, 1681, 1459, 1240, 1015, 995, 881, 676, 660 cm^{-1}; HRMS (m/z) Calcd for ($C_{22}H_{34}NO_2Si$) ([M+H]$^+$): 372.2353; found: 372.2359.

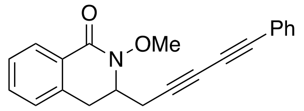

2-Methoxy-3-(5-phenylpenta-2,4-diyn-1-yl)-3,4-dihydroisoquinolin-1(2*H*)-one (3ll). Purification by column chromatography (20% EtOAc in hexanes) gave **3ll** as a yellow oil (52.0 mg, 55% yield); R_f = 0.48 (33% EtOAc in hexanes); ^{1}H NMR (400 MHz, $CDCl_3$): δ 8.09 (d, *J* = 8.0 Hz, 1H), 7.55–7.38 (m, 3H), 7.38–7.17 (m, 5H), 4.20–4.05 (m, 1H), 3.91 (s, 3H), 3.39 (dd, *J* = 16.4, 5.6 Hz, 1H), 3.32 (dd, *J* = 16.4, 4.4 Hz,

1H), 2.91 (dd, J = 17.2, 3.6 Hz, 1H), 2.57 (dd, J = 17.2, 5.6 Hz, 1H); ^{13}C NMR (100 MHz, $CDCl_3$): δ 163.4, 135.0, 132.6, 132.4, 129.1, 128.3, 128.1, 128.0, 127.8, 127.2, 121.4, 79.5, 75.7, 73.6, 67.8, 63.1, 57.7, 32.6, 21.8; IR (neat): 2934, 1670, 1604, 1459, 1422, 1290, 1241, 1015, 996, 739, 687 cm^{-1}; HRMS (m/z) Calcd for ($C_{21}H_{18}NO_2$) ($[M+H]^+$): 316.1332; found: 316.1333.

VI. Mechanism Investigation.

1. Radical Trapping Experiments.

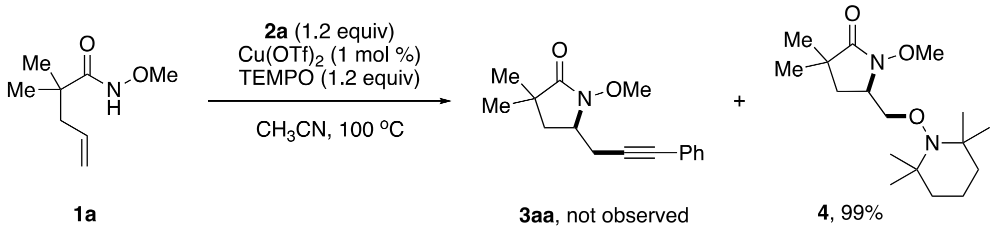

To a reaction tube charged with N-methoxy-2,2-diphenylpent-4-enamide **1a** (31.4 mg, 0.2 mmol, 1.0 equiv) and hypervalent iodine reagent **2a** (83.6 mg, 0.24 mmol, 1.2 equiv), was added $Cu(OTf)_2$ (0.72 mg, 0.002 mmol, 1 mol%), TEMPO (37.5 mg, 0.24 mmol, 1.2 equiv) and CH_3CN (2 mL). The reaction tube was capped and the resulting mixure was stirred at 100 °C for 12 h. After cooling down to room temperature, the reaction mixture was filtered through a pad of Al_2O_3 and washed with EtOAc (15 mL). The filtrate was concentrated *in vacuo*. Aminoalkynylation product **3a** was not observed from the crude ^{1}H NMR. The resulting crude mixture was subject to flash column chromatography to provide aminooxygenation product **4** as a colorless oil (61.9 mg, 99% yield); R_f= 0.25 (12.5% EtOAc in hexanes); ^{1}H NMR (400 MHz, $CDCl_3$): δ 3.95–3.83 (m, 2H), 3.82–3.75 (m, 1H), 3.76 (s, 3H), 1.95 (dd, J = 12.8, 7.2 Hz, 1H), 1.73 (dd, J = 12.8, 7.6 Hz, 1H), 1.59–1.33 (m, 5H), 1.33–1.22 (m, 1H), 1.18 (s, 3H), 1.13 (s, br, 6H), 1.10 (s, 3H), 1.06 (s, 3H), 1.05 (s, 3H); ^{13}C NMR (100 MHz, $CDCl_3$): δ 175.7, 76.5, 62.0, 59.9, 53.1, 39.6, 37.2, 35.4, 32.9, 25.5, 25.0, 20.1, 20.0, 16.9; IR (neat): 2969, 2929, 2869, 1711, 1453, 1373, 1359, 1261, 1243, 1132, 1039, 1024, 929, 794, 745, 556 cm^{-1}; HRMS (m/z) Calcd for ($C_{17}H_{33}N_2O_3$) ($[M+H]^+$): 313.2489; found: 313.2485.

2. Deuterium-Labeling Experiments.

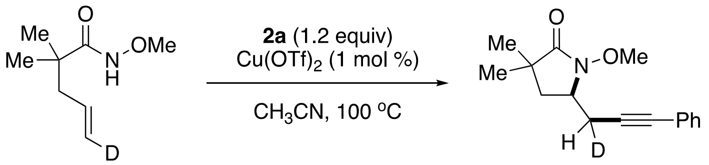

To a reaction tube charged with (E)-N-methoxy-2,2-dimethylpent-4-en-5-d-amide ***D*-1a** (47.4 mg, 0.3 mmol, 1.0 equiv) and hypervalent iodine reagent **2a** (125.3 mg, 0.36 mmol, 1.2 equiv), was added $Cu(OTf)_2$ (1.08 mg, 0.003 mmol, 1 mol%) and CH_3CN (1.5 mL). The reaction tube was capped and the resulting mixure was stirred at 100 °C for 12 h. After cooling down to room temperature, the reaction mixture was filtered through a pad of Al_2O_3 and washed with EtOAc (15 mL). The filtrate was concentrated *in vacuo*. The resulting crude mixture was subject to flash column chromatography (33% EtOAc in hexanes) to provide the aminoalkynylation product **6** as a yellow oil (55.4 mg, 72% yield, inseparable diastereoisomer, dr = 1:1); R_f = 0.30 (33% EtOAc in hexanes); ^{1}H NMR (500 MHz, $CDCl_3$): δ 7.50–7.35 (m, 2H), 7.35–7.22 (m, 3H),

3.90 (s, 3H), 3.92–2.82 (m, 1H), 2.81 (s, br, 0.5 H), 2.78 (d, J = 4.5 Hz, 0.5H), 2.06 (dd, J = 12.5, 7.5 Hz, 1H), 1.93–1.80 (m, 1H), 1.25 (s, 3H), 1.16 (s, 3H); ^{13}C NMR (125 MHz, $CDCl_3$): δ 176.2, 131.4, 128.2, 128.0, 123.0, 84.3, 83.6, 63.0, 53.8, 37.4, 37.2, 25.7, 25.0, 23.4 (t, J = 18.9 Hz); IR (neat): 2963, 2931, 2868, 1706, 1441, 1361, 1259, 1015, 755, 691 cm^{-1}; HRMS (m/z) Calcd for ($C_{16}H_{29}DNO_2$) ($[M+H]^+$): 259.1551; found: 259.1551.

3. Radical Clock Experiments.

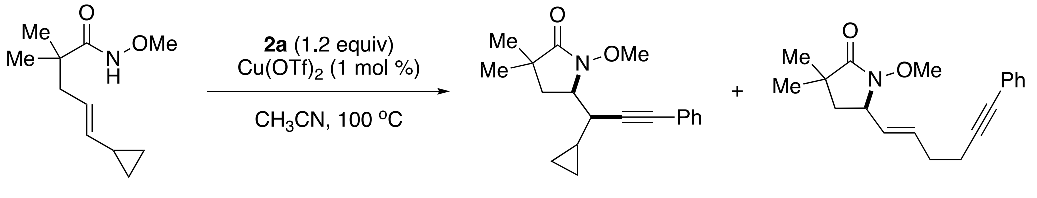

1v **3va** (not observed) **6** (45%)

To a reaction tube charged with *N*-methoxy-2,2-diphenylpent-4-enamide **1v** (59.1 mg, 0.3 mmol, 1.0 equiv) and hypervalent iodine reagent **2a** (125.3 mg, 0.36 mmol, 1.2 equiv), was added $Cu(OTf)_2$ (1.08 mg, 0.003 mmol, 1 mol%) and CH_3CN (1.5 mL). The reaction tube was capped and the resulting mixure was stirred at 100 °C for 5 h. After cooling down to room temperature, the reaction mixture was filtered through a pad of Al_2O_3 and washed with EtOAc (15 mL). The filtrate was concentrated *in vacuo*. Aminoalkynylation product **3va** was not observed from the crude ^{1}H NMR. The resulting crude mixture was subject to flash column chromatography to provide cyclopropane ring opening product **5** as a yellow oil (40.1 mg, 45% yield); R_f= 0.14 (25% EtOAc in hexanes); ^{1}H NMR (500 MHz, $CDCl_3$): δ 7.50–7.35 (m, 2H), 7.35–7.22 (m, 3H), 5.91 (dt, J = 15.5, 7.0 Hz, 1H), 5.52 (dd, J = 15.5, 8.5 Hz, 1H), 4.11 (q, J = 8.0 Hz, 1H), 3.74 (s, 3H), 2.53 (t, J = 7.0 Hz, 2H), 2.47–2.33 (m, 2H), 2.01 (dd, J = 12.5, 7.0 Hz, 1H), 1.62 (dd, J = 12.5, 8.5 Hz, 1H), 1.20 (s, 3H), 1.13 (s, 3H); ^{13}C NMR (125 MHz, $CDCl_3$): δ 176.1, 133.8, 131.4, 130.2, 128.1, 127.6, 123.6, 89.0, 81.3, 63.2, 58.1, 39.6, 37.5, 31.3, 25.5, 24.4, 19.2; IR (neat): 2964, 2931, 2868, 1709, 1441, 1241, 1033, 1014, 967, 756, 692 cm^{-1}; HRMS (m/z) Calcd for ($C_{19}H_{24}NO_2$) ($[M+H]^+$): 298.1802; found: 298.1799.

4. ^{13}C-Labeling Experiments.

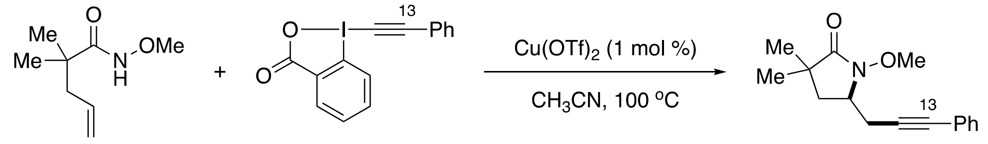

1a **^{13}C-2a** **7**, 76%

To a reaction tube charged with *N*-methoxy-2,2-dimethylpent-4-en-5-*d*-amide **1a** (23.6 mg, 0.15 mmol, 1.0 equiv) and hypervalent iodine reagent **^{13}C-2a**[4] (62.9 mg, 0.18 mmol, 1.2 equiv), was added $Cu(OTf)_2$ (0.54 mg, 0.0015 mmol, 1 mol%) and CH_3CN (1 mL). The reaction tube was capped and the resulting mixure was stirred at 100 °C for 2 h. After cooling down to room temperature, the reaction mixture was filtered through a pad of Al_2O_3 and washed with EtOAc (15 mL). The filtrate was concentrated *in vacuo*. The resulting crude mixture was subject to flash column chromatography (33% EtOAc in hexanes) to provide the aminoalkynylation product **7** as a yellow oil (29.7 mg, 77% yield); R_f= 0.30 (33% EtOAc in hexanes); ^{1}H NMR (500 MHz, $CDCl_3$): δ 7.45–7.33 (m, 2H), 7.33–7.23 (m, 3H), 3.89 (s, 3H), 3.92–3.82 (m, 1H), 2.90–2.70 (m, 2H), 2.06 (dd, J = 12.5, 7.5 Hz, 1H), 1.87 (dd, J = 12.5, 8.0 Hz, 1H), 1.24 (s, 3H), 1.16 (s, 3H); ^{13}C

NMR (125 MHz, $CDCl_3$): δ 176.3, 131.5, 128.2 (d, J = 5.0 Hz), 128.0, 123.0 (d, J = 90.8 Hz), 84.0, 83.7 (^{13}C enriched), 63.0, 53.8, 37.5, 37.2, 25.7, 25.0, 23.6; IR (neat): 2964, 2930, 2868, 1706, 1441, 1362, 1264, 1035, 1014, 928, 755, 691 cm^{-1}; HRMS (m/z) Calcd for ($C_{15}{}^{13}CH_{20}NO_2$) ($[M+H]^+$): 259.1522; found: 259.1525.

VII. Synthesis of Bicyclic Heterocycles.

1. Selective Deprotection and Gold-Catalyzed Cyclization

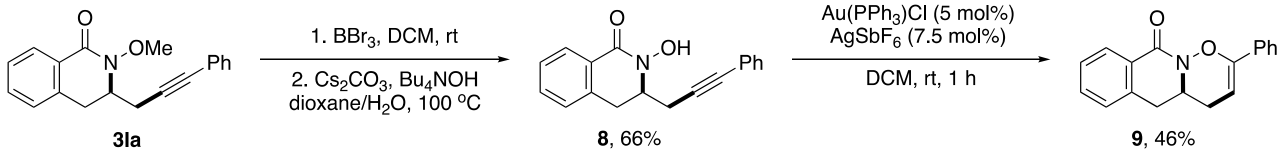

To a 25 mL flask was added a solution of **3la** (146 mg, 0.5 mmol, 1.0 equiv) in DCM (3.5 mL). Then BBr_3 (1.0 M in DCM, 1.5 mL, 1.5 mmol, 3.0 equiv) was added dropwise. The resulting mixture was allowed to stir at room temperature for 1 h before quenching with a saturated aqueous solution of $NaHCO_3$ (10 mL). The organic layer was separated and the aqueous layer was extracted with DCM (10 mL). The combined organic solvent was dried over Na_2SO_4 and filtered off. The filtrate was concentrated under reduced pressure. The residue was dissolved in dioxane (3 mL). Then Bu_4NOH (1.5 M in H_2O, 1.5 mL, 2.25 mmol, 4.5 equiv), Cs_2CO_3 (353 mg, 1 mmol, 2.0 equiv) and H_2O (1.5 mL) were added sequencially. The resulting mixture was allowed to stir at 100 °C for 1 h.[5] The reaction mixture was diluted with EtOAc (20 mL) and washed with a saturated aqueous solution of NH_4Cl (10 mL). The organic phase was dried over anhydrous Na_2SO_4, filtered and concentrated. Purification by column chromatography (33% EtOAc in hexanes) gave 2-hydroxy-3-(3-phenylprop-2-yn-1-yl)-3,4-dihydroisoquinolin-1(2*H*)-one **8** as a yellow oil (90.8 mg, 66% yield); R_f= 0.38 (33% EtOAc in hexanes); ^1H NMR (400 MHz, $CDCl_3$): δ 9.50–8.50 (br, 1H), 8.01 (d, J = 7.6 Hz, 1H), 7.60–7.10 (m, 8H), 4.40–4.20 (m, 1H), 3.50 (dd, J = 16.4, 6.4 Hz, 1H), 3.33 (dd, J = 16.4, 4.0 Hz, 1H), 3.05 (dd, J = 16.8, 2.8 Hz, 1H), 2.69 (dd, J = 16.8, 9.2 Hz, 1H); ^{13}C NMR (125 MHz, $CDCl_3$): δ 161.6, 134.9, 132.4, 131.5, 128.2, 128.1, 128.0, 127.4, 127.2, 126.5, 123.0, 84.9, 83.5, 57.8, 31.7, 22.0; IR (neat): 3060, 2858, 1641, 1463, 729, 689 cm^{-1}; HRMS (m/z) Calcd for ($C_{18}H_{16}NO_2$) ($[M+H]^+$): 278.1175; found: 278.1176.

To a 4 mL vial was added Ph_3PAuCl (2.47 mg, 0.005 mmol, 5 mol%), $AgSbF_6$ (2.58 mg, 0.0075 mmol, 7.5 mo%) and DCM (1 mL). The resulting mixture was allowed to stir at room temperature for 15 min before adding a solution of 2-hydroxy-3-(3-phenylprop-2-yn-1-yl)-3,4-dihydroisoquinolin-1(2*H*)-one **8** (27.7 mg, 0.1 mmol, 1.0 equiv) in DCM (1 mL). The resulting mixture was allowed to stir at room temperature for 1 h. Purification by column chromatography (20% EtOAc in hexanes) gave 2-phenyl-4*a*,5-dihydro-[1,2]oxazino[2,3-*b*]isoquinolin-10(4*H*)-one **9** as a colorless oil (12.8 mg, 46% yield); R_f= 0.62 (33% EtOAc in hexanes); ^1H NMR (400 MHz, $CDCl_3$): δ 8.15 (d, J = 8.0 Hz, 1H), 7.73 (d, J = 7.6 Hz, 2H), 7.60–7.40 (m, 2H), 7.40–7.30 (m, 3H), 7.21 (d, J = 7.2 Hz, 1H), 5.32 (dd, J = 5.2, 2.8 Hz, 1H), 4.55–4.40 (m, 1H), 3.75 (dd, J = 16.4, 6.0 Hz, 1H), 3.02 (dd, J = 16.4, 2.4 Hz, 1H), 2.48 (ddd, J = 17.6, 11.2, 2.8 Hz, 1H), 2.19 (dt, J =

17.2, 5.2 Hz, 1H); ^{13}C NMR (100 MHz, $CDCl_3$): δ 164.1, 155.4, 135.4, 133.1, 132.9, 129.0, 128.7, 128.3, 128.1, 127.4, 126.9, 125.6, 97.1, 55.5, 33.0, 25.8; IR (neat): 3058, 2928, 2837, 1685, 1662, 1245, 926, 757 cm^{-1}; HRMS (m/z) Calcd for ($C_{18}H_{16}NO_2$) ([M+H]$^+$): 278.1175; found: 278.1177.

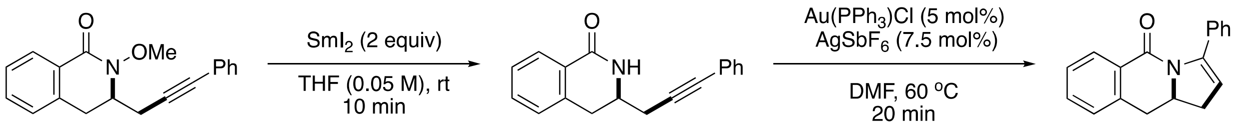

3la **10**, 95% **11**, 75%

A 25 mL round-bottomed flask was charged with **3la** (232.8 mg, 0.8 mmol, 1.0 equiv). The flask was degassed and refilled with N_2 for three times. Then SmI_2 (0.1 M in THF, 16 mL, 1.6 mmol, 2.0 equiv) was added dropwise under N_2 atmosphere. The resulting mixture was stirred at room temperature for 10 min and then the reaction was quenched by the addition of a saturated aqueous solution of $Na_2S_2O_3$. The organic layer was separated and the aqueous layer was extracted with EtOAc. The combined organic solvent was dried over Na_2SO_4 and filtered off. The filtrate was concentrated under reduced pressure. Purification by column chromatography (50% EtOAc in hexanes) gave 3-(3-phenylprop-2-yn-1-yl)-3,4-dihydroisoquinolin-1(2*H*)-one **10** as a yellow solid (198.4 mg, 95% yield); R_f= 0.21 (33% EtOAc in hexanes); ^{1}H NMR (400 MHz, $CDCl_3$): δ 8.07 (d, *J* = 7.6 Hz, 1H), 7.45 (t, *J* = 7.6 Hz, 1H), 7.42–7.32 (m, 3H), 7.32–7.25 (m, 3H), 7.22 (d, *J* = 7.6 Hz, 1H), 6.26 (s, br, 1H), 4.00–3.90 (m, 1H), 3.10 (dd, *J* = 15.6, 4.8 Hz, 1H), 3.00 (dd, *J* = 15.6, 9.6 Hz, 1H), 2.80–2.60 (m, 2H); ^{13}C NMR (125 MHz, $CDCl_3$, one carbon missing): δ 165.8, 137.1, 132.4, 131.6, 128.3, 128.2, 128.0, 127.5, 127.2, 122.7, 84.4, 83.9, 50.3, 33.7, 26.3; IR (neat): 3188, 3053, 2924, 2896, 1660, 1335, 743, 687 cm^{-1}; HRMS (m/z) Calcd for ($C_{18}H_{16}NO$) ([M+H]$^+$): 262.1226; found: 262.1224.

To a 4 mL vial was added Ph_3PAuCl (2.47 mg, 0.005 mmol, 5 mol%), $AgSbF_6$ (2.58 mg, 0.0075 mmol, 7.5 mol%) and DMF (0.5 mL). The resulting mixture was allowed to stir at room temperature for 15 min before adding a solution of 3-(3-phenylprop-2-yn-1-yl)-3,4-dihydroisoquinolin-1(2*H*)-one **10** (26.1 mg, 0.1 mmol, 1.0 equiv) in DMF (0.5 mL). The resulting mixture was allowed to stir at 60 °C for 20 min. The reaction was quenched by the addition of H_2O and Et_2O. The organic layer was separated and the aqueous layer was extracted with Et_2O. The combined organic solvent was dried over Na_2SO_4 and filtered off. The filtrate was concentrated under reduced pressure. Purification by column chromatography (20% EtOAc in hexanes) gave 3-phenyl-10,10*a*-dihydropyrrolo[1,2-*b*]isoquinolin-5(1*H*)-one **11** as a colorless oil (19.6 mg, 75% yield); R_f = 0.59 (33% EtOAc in hexanes); ^{1}H NMR (400 MHz, CD_3OD): δ 7.86 (d, *J* = 8.0 Hz, 1H), 7.62–7.22 (m, 8H), 5.60–5.50 (m, 1H), 4.50–4.30 (m, 1H), 3.40–3.20 (m, 1H), 3.20–3.00 (m, 1H), 3.00–2.90 (m, 1H), 2.70–2.50 (m, 1H); ^{13}C NMR (125 MHz, CD_3OD): δ 162.8, 145.8, 139.1, 134.7, 131.6, 129.1, 128.8, 128.5, 128.4, 128.2, 116.6, 60.6, 36.9, 35.7; IR (neat): 3055, 2918, 2849, 1655, 1400, 1352, 743, 695 cm^{-1}; HRMS (m/z) Calcd for ($C_{18}H_{16}NO$) ([M+H]$^+$): 262.1226; found: 262.1226.

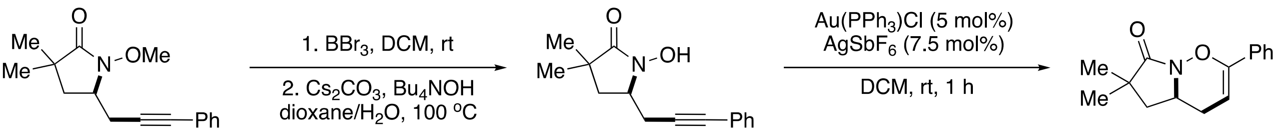

3aa **3aa-S1**, 79% **3aa-S2**, 38%

To a 100 mL flask was added a solution of **3aa** (1.03 g, 4 mmol, 1.0 equiv) in DCM (10 mL). Then BBr_3 (1.0 M in DCM, 12 mL, 12 mmol, 3.0 equiv) was added dropwise. The resulting mixture was allowed to stir at room temperature for 1 h before quenching with a saturated aqueous solution of $NaHCO_3$ (50 mL). The organic layer was separated and the aqueous layer was extracted with DCM (50 mL). The combined organic solvent was dried over Na_2SO_4 and filtered off. The filtrate was concentrated under reduced pressure. The residue was dissolved in dioxane (20 mL). Then Bu_4NOH (1.5 M in H_2O, 13.3 mL, 20 mmol, 5.0 equiv), Cs_2CO_3 (2.83 g, 8 mmol, 2.0 equiv) and H_2O (8 mL) were added sequencially. The resulting mixture was allowed to stir at 100 °C for 1 h.[5] The reaction mixture was diluted with EtOAc (100 mL) and washed with a saturated aqueous solution of NH_4Cl (50 mL). The organic phase was dried over anhydrous Na_2SO_4, filtered and concentrated. Purification by column chromatography (50% EtOAc in hexanes) gave 1-hydroxy-3,3-dimethyl-5-(3-phenylprop-2-yn-1-yl)pyrrolidin-2-one **3aa-S1** as a yellow oil (767.8 mg, 79% yield); R_f = 0.11 (33% EtOAc in hexanes); ^{1}H NMR (400 MHz, $CDCl_3$): δ 7.45–7.33 (m, 2H), 7.33–7.15 (m, 3H), 4.00–3.80 (m, 1H), 2.97 (dd, *J* = 16.8, 3.6 Hz, 1H), 2.69 (dd, *J* = 16.8, 7.6 Hz, 1H), 2.10 (dd, *J* = 16.8, 7.6 Hz, 1H), 1.82 (dd, *J* = 12.8, 7.2 Hz, 1H), 1.16 (s, 3H), 1.10 (s, 3H); ^{13}C NMR (100 MHz, $CDCl_3$): δ 175.4, 131.5, 128.0, 127.8, 123.1, 84.5, 83.0, 55.6, 37.9, 37.4, 25.7, 25.2, 23.7; IR (neat): 3060, 2962, 2867, 1679, 1489, 1362, 1301, 1268, 1029, 1005, 755, 736, 690 cm^{-1}; HRMS (m/z) Calcd for ($C_{15}H_{18}NO_2$) ($[M+H]^+$): 244.1332; found: 244.1335.

To a 4 mL vial was added Ph_3PAuCl (4.94 mg, 0.01 mmol, 5 mol%), $AgSbF_6$ (5.16 mg, 0.015 mmol, 7.5 mo%) and DCM (1 mL). The resulting mixture was allowed to stir at room temperature for 15 min before adding a solution of 1-hydroxy-3,3-dimethyl-5-(3-phenylprop-2-yn-1-yl)pyrrolidin-2-one **3aa-S1** (48.6 mg, 0.2 mmol, 1.0 equiv) in DCM (1 mL). The resulting mixture was allowed to stir at room temperature for 1 h. Purification by column chromatography (20% EtOAc in hexanes) gave 6,6-dimethyl-2-phenyl-4,4a,5,6-tetrahydro-7*H*-pyrrolo[1,2-*b*][1,2]oxazin-7-one **3aa-S2** as a yellow oil (18.5 mg, 38% yield); R_f= 0.42 (33% EtOAc in hexanes); ^{1}H NMR (400 MHz, $CDCl_3$): δ 7.70–7.50 (m, 2H), 7.45–7.25 (m, 3H), 5.36 (dd, *J* = 6.4, 2.4 Hz, 1H), 3.90–3.75 (m, 1H), 2.54 (dt, *J* = 17.2, 5.2 Hz, 1H), 2.29 (dd, *J* = 12.8, 6.8 Hz, 1H), 2.27 (ddd, *J* = 17.2, 9.6, 2.4 Hz, 1H), 1.61 (dd, *J* = 12.8, 7.2 Hz, 1H), 1.26 (s, 3H), 1.23 (s, 3H); ^{13}C NMR (100 MHz, $CDCl_3$): δ 171.7, 152.0, 132.4, 128.8, 128.2, 125.0, 94.7, 48.9, 39.9, 37.9, 29.6, 25.9, 25.3; IR (neat): 2962, 2927, 2868, 1714, 1658, 1446, 1328, 1307, 1276, 1239, 1076, 1052, 1004, 982, 756, 691 cm^{-1}; HRMS (m/z) Calcd for ($C_{15}H_{18}NO_2$) ($[M+H]^+$): 244.1332; found: 244.1338.

2. Synthesis of Pyrrolizidine Derivatives

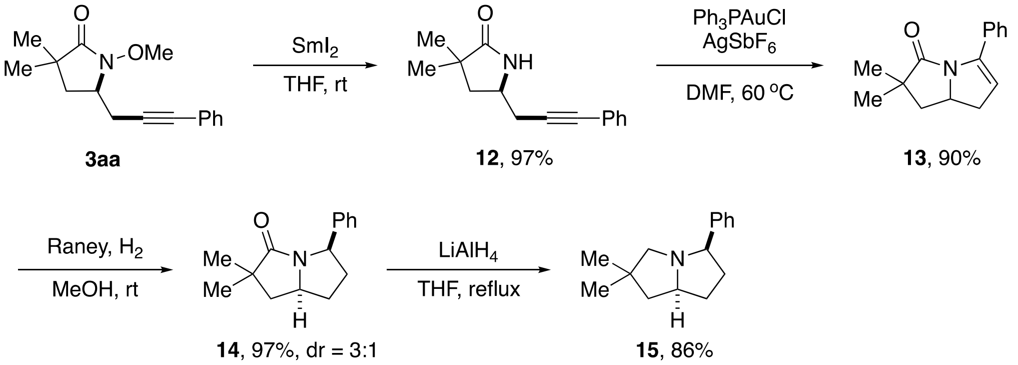

A 25 mL round-bottomed flask was charged with **3aa** (257 mg, 1 mmol, 1.0 equiv). The flask was degassed and refilled with N_2 for three times. Then SmI_2 (0.1 M in THF, 20 mL, 2.0 mmol, 2.0 equiv) was added dropwise under N_2 atmosphere. The resulting mixture was stirred at room temperature for 30 min and then the reaction was quenched by the addition of a saturated aqueous solution of $Na_2S_2O_3$. The organic layer was separated and the aqueous layer was extracted with EtOAc. The combined organic solvent was dried over Na_2SO_4 and filtered off. The filtrate was concentrated under reduced pressure. Purification by column chromatography (50% EtOAc in hexanes) gave 3,3-dimethyl-5-(3-phenylprop-2-yn-1-yl)pyrrolidin-2-one **12** as a white solid (220.2 mg, 97% yield); R_f= 0.21 (50% EtOAc in hexanes); ^{1}H NMR (500 MHz, $CDCl_3$): δ 7.45–7.35 (m, 2H), 7.32–7.20 (m, 4H), 3.84–3.74 (m, 1H), 2.57 (d, *J* = 6.0 Hz, 1H), 2.13 (dd, *J* = 13.0, 7.0 Hz, 1H), 1.71 (dd, *J* = 13.0, 7.5 Hz, 1H), 1.19 (s, 3H), 1.15 (s, 3H); ^{13}C NMR (125 MHz, $CDCl_3$): δ 182.7, 131.4, 128.0, 127.7, 123.0, 85.2, 82.7, 49.9, 42.0, 40.6, 27.0, 25.3, 25.0; IR (neat): 3170, 3078, 2956, 2867, 1682, 1270, 755, 689 cm^{-1}; HRMS (m/z) Calcd for ($C_{15}H_{18}NO$) ([M+H]$^+$): 228.1383; found: 228.1384.

To a 4 mL vial was added Ph_3PAuCl (4.94 mg, 0.01 mmol, 5 mol%), $AgSbF_6$ (5.15 mg, 0.015 mmol, 7.5 mol%) and DMF (1 mL). The resulting mixture was allowed to stir at room temperature for 15 min before adding a solution of 3,3-dimethyl-5-(3-phenylprop-2-yn-1-yl)pyrrolidin-2-one **12** (45.4 mg, 0.2 mmol, 1.0 equiv) in DMF (1 mL). The resulting mixture was allowed to stir at 60 °C for 30 min. The reaction was quenched by the addition of H_2O and Et_2O. The organic layer was separated and the aqueous layer was extracted with Et_2O. The combined organic solvent was dried over Na_2SO_4 and filtered off. The filtrate was concentrated under reduced pressure. Purification by column chromatography (25% EtOAc in hexanes) gave 2,2-dimethyl-5-phenyl-1,2,7,7a-tetrahydro-3*H*-pyrrolizin-3-one **13** as a colorless oil (40.6 mg, 90% yield); R_f= 0.56 (33% EtOAc in hexanes); ^{1}H NMR (500 MHz, CD_3OD): δ 7.47 (d, *J* = 8.0 Hz, 2H), 7.35–7.25 (m, 3H), 5.79–5.74 (m, 1H), 4.70–4.55 (m, 1H), 2.72 (ddd, *J* = 16.5, 9.0, 3.5 Hz, 1H), 2.40 (dd, *J* = 16.5, 10.5 Hz, 1H), 2.30 (dd, *J* = 12.0, 6.5 Hz, 1H), 1.84 (dd, *J* = 12.0, 10.0 Hz, 1H), 1.33 (s, 3H), 1.16 (s, 3H); ^{13}C NMR (125 MHz, CD_3OD): δ 179.4, 142.7, 132.1, 129.5, 128.9, 128.2, 116.7, 62.2, 48.4, 44.5, 37.0, 25.3, 24.7; IR (neat): 2961, 2928, 2867, 1695, 1447, 1388, 1287, 1243, 751, 696 cm^{-1}; HRMS (m/z) Calcd for ($C_{15}H_{18}NO$) ([M+H]$^+$): 228.1383; found: 228.1384.

To a 15 mL round-bottomed flask was added Raney Ni (40 mg), 2,2-dimethyl-5-phenyl-1,2,7,7a-tetrahydro-3*H*-pyrrolizin-3-one **13** (45.4 mg, 0.2 mmol) and MeOH (8 mL) under N_2 atmosphere. An atmosphere of

hydrogen was introduced by briefly evacuating the flask, then flushing with pure hydrogen (1 atm, hydrogen balloon). The mixture was stirred at room temperature overnight under H_2 atmosphere. The hydrogen was then removed under vacuum, and the flask was refilled with N_2. The mixture was filtered through a pale of Celite and the filtrate was concentrated under reduced pressure. The crude reaction mixture contains two diastereoisomers with d.r. = 3:1 detected by ^{1}H NMR. Purification by column chromatography (33% EtOAc in hexanes) gave 2,2-dimethyl-5-phenylhexahydro-3*H*-pyrrolizin-3-one **14** as a mixture of separable diastereomers (34.2 mg major and 10.4 mg minor, 97% yield, relative stereochemistry was determined by the NOESY of both diasteromers).

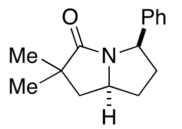

Major diastereomer: colorless oil; R_f = 0.20 (33% EtOAc in hexanes); ^{1}H NMR (500 MHz, $CDCl_3$): δ 7.45–7.05 (m, 5H), 4.67 (d, *J* = 9.0 Hz, 1H), 4.05–3.95 (m, 1H), 2.70–2.50 (m, 1H), 2.25–2.10 (m, 1H), 2.10–1.90 (m, 2H), 1.90–1.75 (m, 1H), 1.75–1.55 (m, 1H), 1.23 (s, 3H), 1.21 (s, 3H); ^{13}C NMR (125 MHz, $CDCl_3$): δ 175.7, 141.4, 128.2, 126.8, 125.7, 59.6, 56.4, 47.3, 43.0, 38.7, 28.8, 25.5, 23.1; IR (neat): 2951, 2867, 1677, 1412, 748, 695 cm^{-1}; HRMS (m/z) Calcd for ($C_{15}H_{20}NO$) ([M+H]$^+$): 230.1539; found: 230.1546.

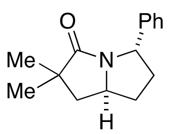

Minor diastereomer: colorless oil; R_f= 0.37 (33% EtOAc in hexanes); ^{1}H NMR (500 MHz, $CDCl_3$): δ 7.60–7.10 (m, 5H), 4.95 (t, *J* = 8.0 Hz, 1H), 4.20–4.00 (m, 1H), 2.75–2.60 (m, 1H), 2.35–2.10 (m, 2H), 2.10–1.95 (m, 1H), 1.75–1.60 (m, 1H), 1.52–1.38 (m, 1H), 1.33 (s, 3H), 1.21 (s, 3H); ^{13}C NMR (125 MHz, $CDCl_3$): δ 179.5, 142.7, 128.4, 126.7, 125.3, 58.2, 56.8, 45.7, 43.7, 37.0, 33.2, 25.5, 25.1; IR (neat): 2959, 2866, 1687, 1400, 1132, 752, 696 cm^{-1}; HRMS (m/z) Calcd for ($C_{15}H_{20}NO$) ([M+H]$^+$): 230.1539; found: 230.1545.

Relative stereochemistry was assigned on the basis of NOESY.

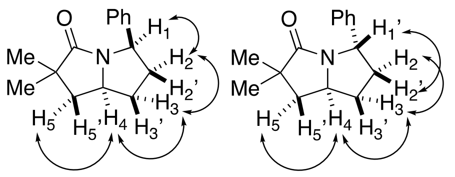

To a 25 mL round-bottomed flask charged with $LiAlH_4$ (19 mg, 0.5 mmol, 5.0 equiv), was added a solution of 2,2-dimethyl-5-phenylhexahydro-3*H*-pyrrolizin-3-one **14-major** (22.9 mg, 0.1 mmol, 1.0 equiv) in THF (4 mL) at room temperature. The resulting mixture was refluxed for 3 h. The reaction was quenched with the addition of an aqueous solution of NaOH (2 M, 1 mL). The mixture was filtered through a pale of Celite. The solvent was removed under reduced pressure to give 2,2-dimethyl-5-phenylhexahydro-1*H*-pyrrolizine **15** as a colorless oil (18.5 mg, 86% yield); R_f= 0.19 (5% MeOH in DCM); ^{1}H NMR (500 MHz, $CDCl_3$): δ 7.43 (d, *J* = 7.5 Hz, 2H), 7.33 (t, *J* = 7.5 Hz, 2H), 7.26 (t, *J* = 7.5 Hz, 1H), 4.22–4.10 (m, 1H), 3.82–3.67 (m, 1H), 2.20–1.80 (m, 6H), 1.70–1.60 (m, 1H), 1.26–1.16 (m, 1H), 1.10 (s, 3H), 0.97 (s, 3H); ^{13}C NMR (125 MHz, $CDCl_3$): δ 140.2, 128.4, 127.9, 126.8, 65.8, 64.2, 62.1, 48.0, 39.6, 31.6, 27.5, 26.9, 26.8; IR (neat): 2949, 2863, 1449, 1364, 1120, 1056, 744, 697 cm^{-1}; HRMS (m/z) Calcd for ($C_{15}H_{22}N$) ([M+H]$^+$): 216.1747; found: 216.1751.

VIII. Synthesis of Alkyne-Labeled Mesembrane.

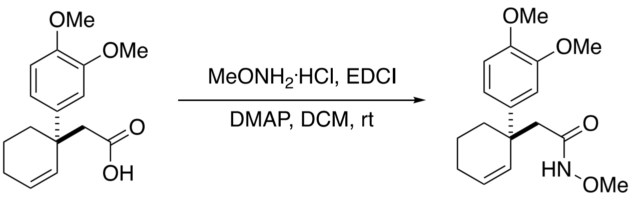

16, 59%

To a solution of 2-(3',4'-dimethoxy-3,4-dihydro-[1,1'-biphenyl]-1(2*H*)-yl)acetic acid (3.3 g, 12 mmol, 1.0 equiv) in CH_2Cl_2 (60 mL) was added $MeONH_2$·HCl (1.5 g, 18 mmol, 1.5 equiv), EDCI (4.62 g, 24 mmol, 2.0 equiv) and DMAP (2.94 g, 24 mmol, 2.0 equiv) successively. The resulting mixture was allowed to stire at room temperature overnight, and then an aqueous solution of HCl (2 M) was added. The organic layer was separated and the aqueous layer was extracted with CH_2Cl_2 (10 mL). The combined extracts were washed with brine, dried over Na_2SO_4, and filtrated. The filtrate was concentrated. Purification by column chromatography (50% EtOAc in hexanes) gave **16** as a yellow oil (2.16 g, 59% yield); R_f = 0.12 (50% EtOAc in hexanes); ^{1}H NMR (500 MHz, $CDCl_3$): δ 8.25 (s, 1H), 6.90–6.82 (m, 2H), 6.82–6.74 (m, 1H), 6.00 (d, J = 10.0 Hz, 1H), 5.92 (d, J = 10.0 Hz, 1H), 3.82 (s, 3H), 3.81 (s, 3H), 3.48 (s, 3H), 2.60–2.25 (m, 2H), 2.07–1.97 (m, 3H), 1.85 (t, J = 12.0 Hz, 1H), 1.62–1.45 (m, 1H), 1.39–1.20 (m, 1H); ^{13}C NMR (125 MHz, $CDCl_3$): δ 168.5, 148.5, 147.2, 139.0, 131.4, 129.1, 119.0, 110.8, 110.3, 63.9, 55.8, 55.7, 46.7, 41.6, 36.4, 25.0, 18.5; IR (neat): 3190, 2931, 2833, 1651, 1511, 1442, 1252, 1135, 1023, 756, 699 cm^{-1}; HRMS (m/z) Calcd for ($C_{17}H_{24}NO_4$) ($[M+H]^+$): 306.1700; found: 306.1697.

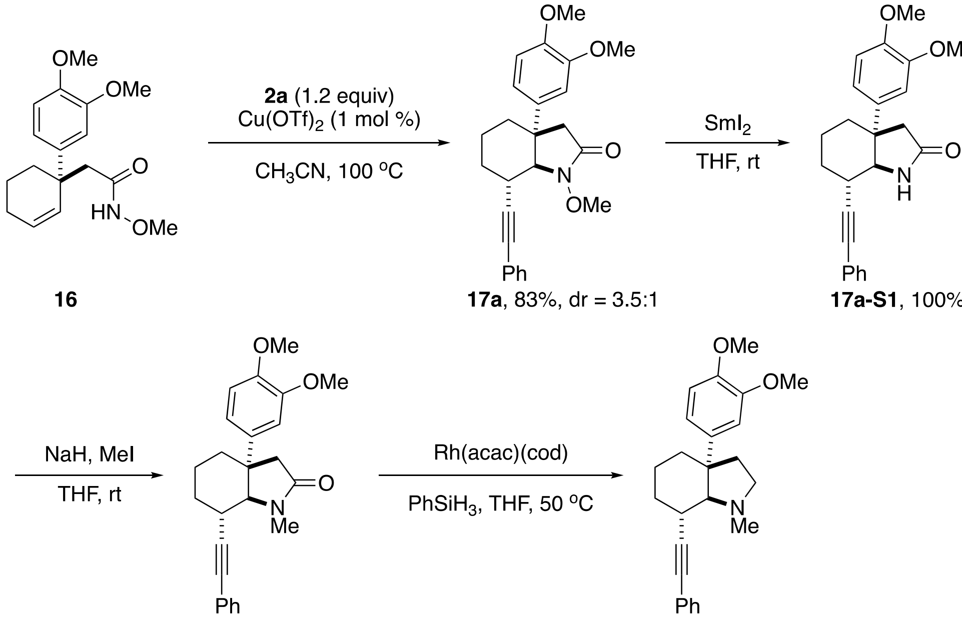

16 **17a**, 83%, dr = 3.5:1 **17a-S1**, 100%

17a-S2, 93% **18a**, 94%

To a reaction tube charged with **16** (91.5 mg, 0.3 mmol, 1.0 equiv) and hypervalent iodine reagent **2a** (125.3 mg, 0.36 mmol, 1.2 equiv), was added $Cu(OTf)_2$ (1.08 mg, 0.003 mmol, 1 mol %) and CH_3CN (1.5 mL). The reaction tube was capped and the resulting mixure was allowed to stir at 100 °C for 12 h. After cooling down to room temperature, the reaction mixture was filtered through a pad of Al_2O_3 and washed with EtOAc (15 mL). The filtrate was concentrated *in vacuo*. The resulting crude mixture was subject to flash column

chromatography to provide the aminoalkynylation product **17a** as a mixture of separable diastereomers (100.7 mg, 83% yield, d.r. = 3.5:1 detected by crude ^{1}H NMR, major diastereomer shown); R_f = 0.35 (50% EtOAc in hexanes); ^{1}H NMR (500 MHz, $CDCl_3$): δ 7.25–7.15 (m, 3H), 7.07 (d, *J* = 8.5 Hz, 1H), 7.05–6.95 (m, 3H), 6.78 (d, *J* = 8.5 Hz, 1H), 4.30 (d, *J* = 3.5 Hz, 1H), 3.88 (s, 3H), 3.82 (s, 3H), 3.81 (s, 3H), 3.27 (q, *J* = 3.5 Hz, 1H), 2.59 (d, *J* = 17.0 Hz, 1H), 2.43 (d, *J* = 17.0 Hz, 1H), 2.36–2.26 (m, 1H), 2.07–1.95 (m, 1H), 1.95–1.85 (m, 1H), 1.85–1.73 (m, 2H), 1.67–1.57 (m, 1H); ^{13}C NMR (125 MHz, $CDCl_3$): δ 170.6, 148.7, 147.7, 136.7, 131.2, 127.8, 127.7, 122.9, 118.8, 110.8, 110.3, 89.7, 83.0, 65.6, 62.7, 56.0, 55.7, 45.2, 39.8, 32.8, 28.1, 25.8, 18.6; IR (neat): 2940, 2855, 1714, 1520, 1445, 1241, 1145, 1020, 756 cm^{-1}; HRMS (m/z) Calcd for ($C_{25}H_{28}NO_4$) ($[M+H]^+$): 406.2013; found: 406.2011.

A 25 mL round-bottomed flask was charged with **17a-major** (202.5 mg, 0.5 mmol, 1.0 equiv). The flask was degassed and refilled with N_2 for three times. Then SmI_2 (0.1 M in THF, 10 mL, 1.0 mmol, 2.0 equiv) was added dropwise under N_2 atmosphere. The resulting mixture was stirred at room temperature for 5 min and then the reaction was quenched by the addition of a saturated aqueous solution of $Na_2S_2O_3$. The organic layer was separated and the aqueous layer was extracted with EtOAc. The combined organic solvent was dried over Na_2SO_4 and filtered off. The filtrate was concentrated under reduced pressure to give **17a-S1** as a yellow solid (190.2 mg, 100% yield); R_f = 0.12 (50% EtOAc in hexanes); ^{1}H NMR (500 MHz, $CDCl_3$): δ 7.26 (s, 5H), 7.01 (d, *J* = 8.5 Hz, 1H), 6.95 (s, 1H), 6.80 (d, *J* = 8.5 Hz, 1H), 6.55 (s, br, 1H), 3.94 (d, *J* = 8.0 Hz, 1H), 3.87 (s, 3H), 3.84 (s, 3H), 2.80–2.70 (m, 1H), 2.60 (s, 2H), 2.05–1.90 (m, 3H), 1.90–1.80 (m, 1H), 1.55–1.50 (m, 2H); ^{13}C NMR (125 MHz, $CDCl_3$): δ 176.4, 148.7, 147.7, 138.2, 131.4, 128.1, 127.9, 122.8, 118.2, 110.8, 109.9, 90.1, 82.8, 63.3, 56.0, 55.8, 45.5, 42.4, 34.5, 34.0, 27.8, 20.6; IR (neat): 2936, 1697, 1519, 1442, 1253, 1149, 1025, 758, 693 cm^{-1}; HRMS (m/z) Calcd for ($C_{24}H_{26}NO_3$) ($[M+H]^+$): 376.1907; found: 376.1908.

To a solution of **17a-S1** (75 mg, 0.2 mmol, 1.0 equiv) in THF (2 mL) was added NaH (24 mg, 60% dispersion in mineral oil, 0.6 mmol, 3.0 equiv). The resulting mixture was allowed to stir at room temperature for 30 min and then methyl iodide (85.2 mg, 0.6 mmol, 3.0 equiv) was added. The resulting mixture was allowed to stir at room temperature for 90 min. The reaction was quenched by the addition of a saturated aqueous solution of NH_4Cl. The organic layer was separated and the aqueous layer was extracted with EtOAc. The combined organic solvent was dried over Na_2SO_4 and filtered off. The filtrate was concentrated under reduced pressure. Purification by column chromatography (50% EtOAc in hexanes) gave **17a-S2** as a yellow oil (72.1 mg, 93% yield); R_f = 0.19 (50% EtOAc in hexanes); ^{1}H NMR (500 MHz, $CDCl_3$): δ 7.36–7.16 (m, 5H), 6.97 (d, *J* = 8.5 Hz, 1H), 6.92 (s, 1H), 6.81 (d, *J* = 8.5 Hz, 1H), 3.88 (s, 3H), 3.84 (s, 3H), 3.87–3.82 (m, 1H), 3.02 (s, 3H), 2.90–2.80 (m, 1H), 2.66 (d, *J* = 16.5 Hz, 1H), 2.60 (d, *J* = 16.5 Hz, 1H), 2.08–1.86 (m, 4H), 1.70–1.52 (m, 2H); ^{13}C NMR (125 MHz, $CDCl_3$): δ 173.4, 148.8, 147.7, 138.5, 131.3, 128.1, 127.9, 123.0, 118.1, 110.9, 109.9, 90.9, 83.2, 69.0, 56.0, 55.8, 44.2, 42.8, 33.9, 32.7, 29.5, 28.5, 20.4; IR (neat): 2933, 2859, 1688, 1518, 1442, 1393, 1251, 1148, 1024, 756, 691 cm^{-1}; HRMS (m/z) Calcd for ($C_{25}H_{28}NO_3$) ($[M+H]^+$): 390.2064; found: 390.2063.

To a solution of **17a-S2** (77.8 mg, 0.2 mmol, 1.0 equiv) in THF (1 mL) was added Rh(acac)(cod) (1.24 mg, 0.004 mmol, 2 mol %). Then $PhSiH_3$ (0.1 mL, 0.8 mmol, 4.0 equiv) was added dropwise. The resulting solution was allowed to stir at 50 °C for 0.5 h. The solvent was removed under reduced pressure. Purification by column chromatography (33% EtOAc in hexanes containing 5% TEA) gave **18a** as a yellow oil (70.6 mg, 94% yield); R_f= 0.42 (50% EtOAc in hexanes containing 5% TEA); ^{1}H NMR (500 MHz, $CDCl_3$): δ 7.23–7.11 (m, 3H), 7.10–7.02 (m, 2H), 7.02–6.96 (m, 2H), 6.75 (d, *J* = 8.0 Hz, 1H), 3.84 (s, 3H), 3.78 (s, 3H), 3.28–3.18 (m, 1H), 3.02–2.94 (m, 2H), 2.52–2.45 (m, 1H), 2.44 (s, 3H), 2.16–2.08 (m, 1H), 2.01–1.81 (m, 5H), 1.65–1.53 (m, 2H); ^{13}C NMR (125 MHz, $CDCl_3$): δ 148.3, 146.9, 139.9, 131.3, 127.7, 127.2, 123.6, 119.1, 111.1, 110.5, 92.4, 82.2, 73.4, 56.0, 55.6, 54.1, 47.6, 41.38, 41.31, 33.3, 28.9, 26.2, 19.6; IR (neat): 2932, 2832, 1736, 1517, 1452, 1246, 1145, 1026, 755, 691 cm^{-1}; HRMS (m/z) Calcd for ($C_{25}H_{30}NO_2$) ([M+H]$^+$): 376.2271; found: 376.2272.

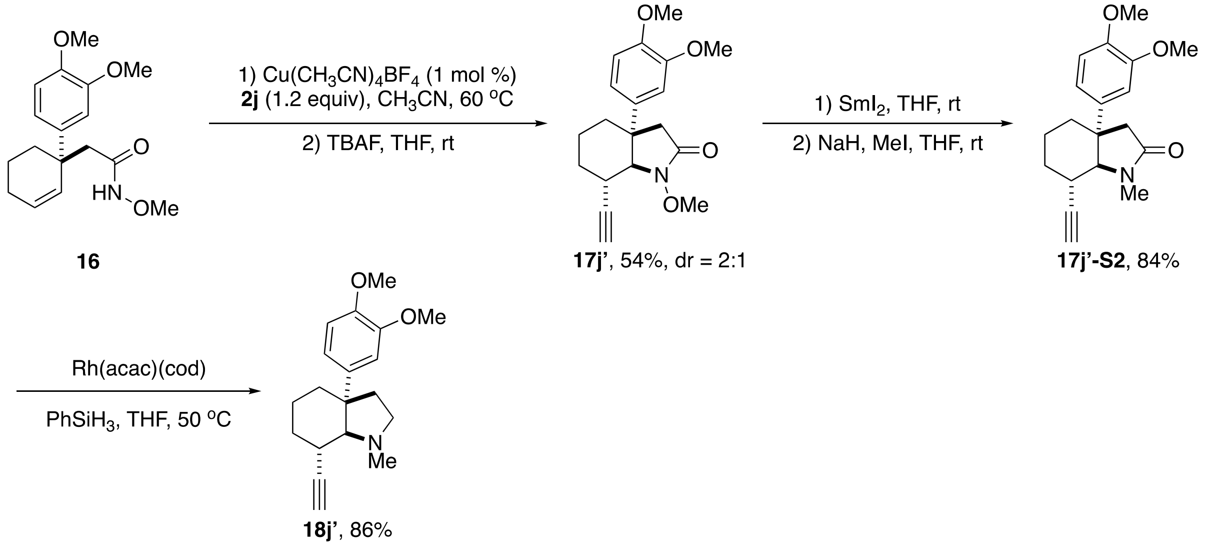

To a reaction tube charged with **16** (91.5 mg, 0.3 mmol, 1.0 equiv) and hypervalent iodine reagent **2j** (139 mg, 0.36 mmol, 1.2 equiv), was added $Cu(CH_3CN)_4BF_4$ (0.95 mg, 0.003 mmol, 1 mol %) and CH_3CN (1.5 mL). The reaction tube was capped and the resulting mixure was allowed to stir at 60 °C for 12 h. After cooling down to room temperature, the reaction mixture was filtered through a pad of Al_2O_3 and washed with EtOAc (15 mL). The filtrate was concentrated *in vacuo*. The residue was dissolved in THF (3 mL). Then TBAF (1 M in THF, 0.4 mL, 0.4 mmol) was added dropwise. The resulting mixure was allowed to stir at room temperature overnight. The reaction was quenched by the addition of a saturated aqueous solution of NH_4Cl. The organic layer was separated and the aqueous layer was extracted with EtOAc. The combined organic solvent was dried over Na_2SO_4 and filtered off. The filtrate was concentrated under reduced pressure. Purification by column chromatography (50% EtOAc in hexanes) gave **17j'** as a mixture of inseparable diastereomers as a white solid (53.46 mg, 54% yield, d.r. = 2:1 detected by crude ^{1}H NMR, major diastereomer shown); R_f= 0.33 (67% EtOAc in hexanes); ^{1}H NMR (400 MHz, $CDCl_3$, signals for the minor diastereoisomer are reported in *italics*): δ 7.00–6.75 (m, 3H, both), 4.20–4.10 (m, 1H, both), 3.90–3.30 (m, 9H, both), 3.05–2.90 (m, 1H, both), *2.56* (d, *J* = 16.8 Hz, 1H), 2.52 (d, *J* = 16.8 Hz, 1H), *2.43* (d, *J* = 16.8

Hz, 1H), 2.41 (d, *J* = 16.8 Hz, 1H), 2.25–2.10 (m, 1H, both), 2.05–1.40 (m, 6H, both); ^{13}C NMR (125 MHz, $CDCl_3$, signals for the minor diastereoisomer are reported in *italics*): δ *172.2*, 170.1, *148.9*, 148.5, *147.8*, 147.6, 136.9, *136.2*, 118.5, *118.1*, *110.9*, 110.6, 110.0, *109.5*, 84.4, *83.9*, *70.7*, 70.6, 65.1, *63.2*, *62.6*, 62.4, 55.9 (both), 55.7 (both), *44.1*, 43.8, *40.6*, 40.0, *33.7*, 33.1, *28.6*, 27.9, *26.5*, 26.0, *20.4*, 18.5; IR (neat): 3247, 2935, 1712, 1516, 1444, 1244, 1146, 1022 cm^{-1}; HRMS (m/z) Calcd for ($C_{19}H_{23}NO_4$) ($[M+H]^+$): 330.1700; found: 330.1702.

A 25 mL round-bottomed flask was charged with **17j'** (131.6 mg, 0.4 mmol, 1.0 equiv). The flask was degassed and refilled with N_2 for three times. Then SmI_2 (0.1 M in THF, 8 mL, 0.8 mmol, 2.0 equiv) was added dropwise under N_2 atmosphere. The resulting mixture was stirred at room temperature for 5 min and then the reaction was quenched by the addition of a saturated aqueous solution of $Na_2S_2O_3$. The organic layer was separated and the aqueous layer was extracted with EtOAc. The combined organic solvent was dried over Na_2SO_4 and filtered off. The filtrate was concentrated under reduced pressure. The residue was dissolved in THF (4 mL) Then NaH (48 mg, 60% dispersion in mineral oil, 1.2 mmol, 3.0 equiv) was added. The resulting mixture was allowed to stir at room temperature for 30 min and then methyl iodide (170.4 mg, 1.2 mmol, 3.0 equiv) was added. The resulting mixture was allowed to stir at room temperature overnight. The reaction was quenched by the addition of a saturated aqueous solution of NH_4Cl. The organic layer was separated and the aqueous layer was extracted with EtOAc. The combined organic solvent was dried over Na_2SO_4 and filtered off. The filtrate was concentrated under reduced pressure. Purification by column chromatography (50% EtOAc in hexanes) gave **17j'-S2** as a colorless oil (105.2 mg, 84% yield); R_f= 0.20 (67% EtOAc in hexanes); ^1H NMR (400 MHz, $CDCl_3$, signals for the minor diastereoisomer are reported in *italics*): δ 6.95–6.75 (m, 3H, both), 3.86 (s, 3H, both), *3.84* (s, 3H), 3.83 (s, 3H), 3.69 (d, *J* = 8.0 Hz, 1H, both), *2.98* (s, 3H), 2.94 (s, 3H), *2.63* (d, *J* = 16.4 Hz, 1H), 2.61 (d, *J* = 16.4 Hz, 1H), 2.51 (d, *J* = 16.4 Hz, 1H), *2.49* (d, *J* = 16.4 Hz, 1H), *2.18* (d, *J* = 2.4 Hz, 1H), 2.11 (d, *J* = 2.4 Hz, 1H), 2.00–1.40 (m, 6H, both); ^{13}C NMR (125 MHz, $CDCl_3$, signals for the minor diastereoisomer are reported in *italics*): δ *174.5*, 173.2, *148.9*, 148.7, *147.70*, 147.66, 138.6, *137.1*, *118.2*, 117.8, *110.9*, 110.8, *109.8*, 109.6, 85.8, *84.4*, *72.0*, 71.1, 68.9, *65.4*, 55.9 (both), 55.8 (both), *45.9*, 44.4, *43.5*, 41.4, 34.2, *33.2*, 32.6, 29.8, *29.0*, 28.9, *28.8*, *26.5*, 20.5, *20.1*; IR (neat): 2934, 1683, 1518, 1450, 1252, 1150, 1024 cm^{-1}; HRMS (m/z) Calcd for ($C_{19}H_{23}NO_3$) ($[M+H]^+$): 314.1751; found: 314.1755.

To a solution of **17j'-S2** (46.95 mg, 0.15 mmol, 1.0 equiv) in THF (1 mL) was added Rh(acac)(cod) (0.93 mg, 0.003 mmol, 2 mol %). Then $PhSiH_3$ (0.075 mL, 0.6 mmol, 4.0 equiv) was added dropwise. The resulting solution was allowed to stir at 50 ^oC for 0.5 h. The solvent was removed under reduced pressure. Purification by column chromatography (33% EtOAc in hexanes containing 5% TEA) gave **18j'** as a colorless oil (38.9 mg, 86% yield); R_f= 0.42 (33% EtOAc in hexanes containing 5% TEA); ^1H NMR (400 MHz, $CDCl_3$, signals for the minor diastereoisomer are reported in *italics*): δ 7.05–6.75 (m, 3H, both), 3.88 (s, 3H), *3.87* (s, 3H), 3.85 (s, 3H, both), *3.35–3.25* (m, 1H), 3.20–3.10 (m, 1H), 2.95–2.85 (m, 1H, both), 2.75–2.55 (m, 2H, both), 2.50–2.30 (m, 3H, both), 2.10–1.40 (m, 9H, both); ^{13}C NMR (125 MHz, $CDCl_3$,

signals for the minor diastereoisomer are reported in *italics*): δ *148.8*, 148.3, *147.1*, 147.0, 140.0, *138.1*, 118.9, *118.7*, 110.9 (both), 110.4 (both), *87.8*, 87.0, 73.1, *71.0*, *69.9*, 69.5, 56.0 (both), 55.8 (both), *55.0*, 53.9, *49.1*, 48.0, *43.3*, 41.8, *40.6*, 40.1, 33.9, *33.2*, 29.1, *28.7*, 26.6 (both), *22.8*, 19.6; IR (neat): 3284, 2933, 2832, 2781, 1517, 1452, 1249, 1146, 1026, 765, 641 cm^{-1}; HRMS (m/z) Calcd for ($C_{19}H_{23}NO_2$) ($[M+H]^+$): 300.1958; found: 300.1962.

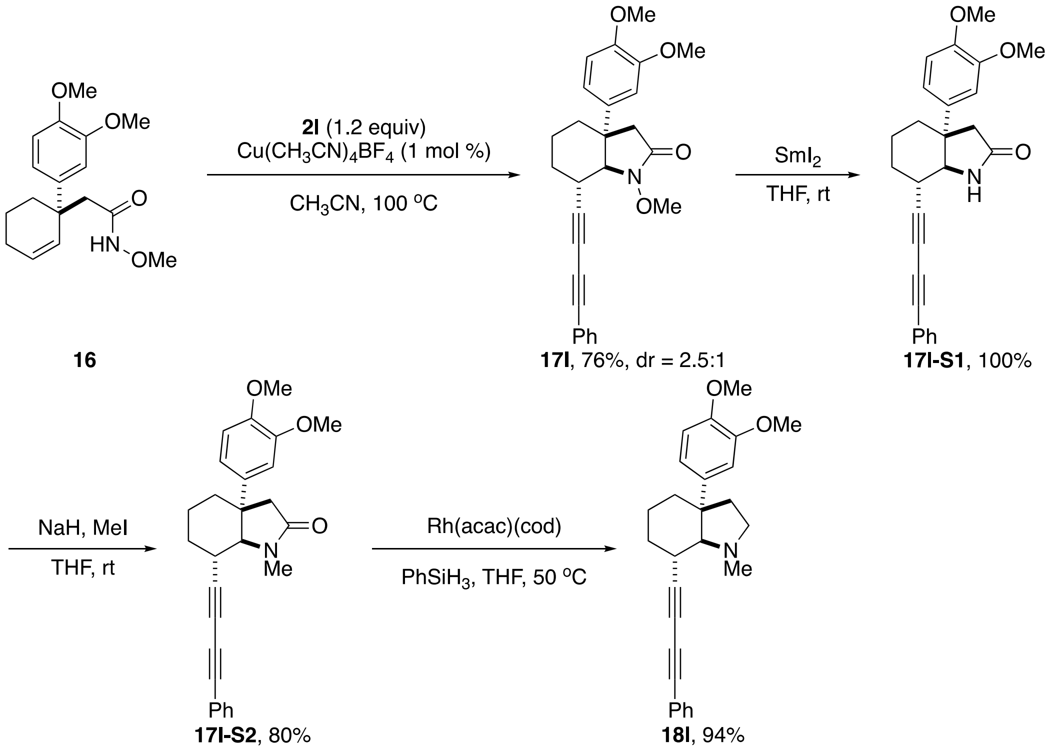

To a reaction tube charged with **16** (91.5 mg, 0.3 mmol, 1.0 equiv) and hypervalent iodine reagent **2l** (133.9 mg, 0.36 mmol, 1.2 equiv), was added $Cu(CH_3CN)_4BF_4$ (0.95 mg, 0.003 mmol, 1 mol %) and CH_3CN (1.5 mL). The reaction tube was capped and the resulting mixure was allowed to stir at 100 °C for 12 h. After cooling down to room temperature, the reaction mixture was filtered through a pad of Al_2O_3 and washed with EtOAc (15 mL). The filtrate was concentrated *in vacuo*. The resulting crude mixture was subject to flash column chromatography to provide the aminoalkynylation product **17l** as a mixture of separable diastereomers (90.5 mg, 76% yield, d.r. = 2.5:1 detected by crude ^1H NMR, relative stereochemistry of **17l** was determined by the X-ray structure of both diasteromers).

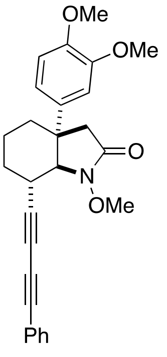

17l-major: brown solid; R_f = 0.35 (50% EtOAc in hexanes); ^1H NMR (500 MHz, $CDCl_3$): δ 7.39 (d, J = 7.5 Hz, 2H), 7.33–7.20 (m, 3H), 7.03–6.93 (m, 2H), 6.82 (d, J = 9.0 Hz, 1H), 4.20 (d, J = 3.5 Hz, 1H), 3.90 (s, 3H), 3.80 (s, 3H), 3.77 (s, 3H), 3.11 (q, J = 3.5 Hz, 1H), 2.54 (d, J = 17.0 Hz, 1H), 2.43 (d, J = 17.0 Hz, 1H), 2.25–2.15 (m, 1H), 1.94–1.80 (m, 2H), 1.80–1.64 (m, 2H), 1.63–1.49 (m, 1H); ^{13}C NMR (125 MHz, $CDCl_3$): δ 170.1, 148.7, 147.7, 136.5, 132.2, 128.8, 128.1, 121.3, 118.4, 110.8, 110.0, 83.7, 76.1, 73.3, 67.5, 64.7, 62.4, 55.9, 55.6, 43.7, 39.9, 33.0, 28.8, 25.6, 18.6; IR (neat): 2939, 1715, 1515, 1442, 1243, 1229, 1144, 1022, 750, 686 cm^{-1}; HRMS (m/z) Calcd for ($C_{27}H_{28}NO_4$) ($[M+H]^+$): 430.2013; found: 430.2008.

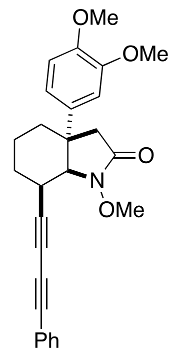

17l-minor: brown solid; R_f = 0.40 (50% EtOAc in hexanes); ^1H NMR (500 MHz, $CDCl_3$): δ 7.34–7.27 (m, 2H), 7.22–7.07 (m, 3H), 6.76–6.60 (m, 3H), 4.16–4.10 (m, 1H), 3.76 (s, 3H), 3.72 (s, 3H), 3.70 (s, 3H), 2.96–2.86 (m, 1H), 2.36 (d, J = 17.0 Hz, 1H), 2.30 (d, J = 17.0 Hz, 1H), 1.97–1.90 (m, 1H), 1.81–1.55 (m, 4H), 1.36–1.24 (m, 1H); ^{13}C NMR (125 MHz, $CDCl_3$): δ 172.2, 149.1, 147.9, 135.4, 132.4, 128.9, 128.2, 121.6, 118.2, 111.0, 109.6, 83.5, 76.2, 73.7, 66.9, 63.5, 62.8, 56.0, 55.8, 45.1, 40.6, 33.4, 29.5, 26.4, 21.0; IR (neat): 2937, 1719, 1517, 1441, 1258, 1232, 1148, 1021, 758, 693 cm^{-1}; HRMS (m/z) Calcd for ($C_{27}H_{28}NO_4$) ($[M+H]^+$): 430.2013; found: 430.2008.

A 25 mL round-bottomed flask was charged with **17l-major** (82.6 mg, 0.2 mmol, 1.0 equiv). The flask was degassed and refilled with N_2 for three times. Then SmI_2 (0.1 M in THF, 4 mL, 0.4 mmol, 2.0 equiv) was added dropwise under N_2 atmosphere. The resulting mixture was stirred at room temperature for 5 min and then the reaction was quenched by the addition of a saturated aqueous solution of $Na_2S_2O_3$. The organic layer was separated and the aqueous layer was extracted with EtOAc. The combined organic solvent was dried over Na_2SO_4 and filtered off. The filtrate was concentrated under reduced pressure to give **17l-S1** as a yellow oil (80.4 mg, 100% yield); R_f = 0.14 (50% EtOAc in hexanes); ^1H NMR (500 MHz, $CDCl_3$): δ 7.44 (d, J = 7.0 Hz, 2H), 7.38–7.24 (m, 3H), 6.96 (d, J = 8.5 Hz, 1H), 6.93 (s, 1H), 6.82 (d, J = 8.5 Hz, 1H), 6.74 (s, br, 1H), 3.94–3.86 (s, 4H), 3.82 (s, 3H), 2.74–2.64 (m, 1H), 2.64–2.52 (m, 2H), 2.01–1.85 (m, 3H), 1.85–1.72 (m, 1H), 1.60–1.45 (m, 2H); ^{13}C NMR (125 MHz, $CDCl_3$): δ 176.1, 148.7, 147.7, 138.0, 132.3, 129.0, 128.2, 121.4, 118.0, 110.8, 109.7, 84.3, 76.3, 73.5, 67.3, 62.5, 55.9, 55.7, 45.6, 41.7, 35.0, 34.1, 27.6, 20.5; IR (neat): 2933, 1693, 1517, 1441, 1250, 1229, 1147, 1022, 753, 687 cm^{-1}; HRMS (m/z) Calcd for ($C_{26}H_{26}NO_3$) ($[M+H]^+$): 400.1907; found: 400.1910.

To a solution of **17l-S1** (60 mg, 0.15 mmol, 1.0 equiv) in THF (1.5 mL) was added NaH (18 mg, 60% dispersion in mineral oil, 0.45 mmol, 3.0 equiv). The resulting mixture was allowed to stir at room temperature for 30 min and then methyl iodide (63.9 mg, 0.45 mmol, 3.0 equiv) was added. The resulting mixture was allowed to stir at room temperature overnight. The reaction was quenched by the addition of a saturated aqueous solution of NH_4Cl. The organic layer was separated and the aqueous layer was extracted with EtOAc. The combined organic solvent was dried over Na_2SO_4 and filtered off. The filtrate was concentrated under reduced pressure. Purification by column chromatography (50% EtOAc in hexanes) gave **17l-S2** as a yellow oil (49.5 mg, 80% yield); R_f = 0.09 (33% EtOAc in hexanes); ^1H NMR (500 MHz, $CDCl_3$): δ 7.46 (d, J = 7.5 Hz, 2H), 7.39–7.27 (m, 3H), 6.92 (d, J = 8.5 Hz, 1H), 6.88 (s, 1H), 6.82 (d, J = 8.5 Hz, 1H), 3.90 (s, 3H), 3.83 (s, 3H), 3.77 (d, J = 8.0 Hz, 1H), 2.98 (s, 3H), 2.75–2.69 (m, 1H), 2.64 (d, J = 16.5 Hz, 1H), 2.57 (d, J = 16.5 Hz, 1H), 2.00–1.76 (m, 4H), 1.65–1.49 (m, 2H); ^{13}C NMR (125 MHz, $CDCl_3$, one carbon missing): δ 173.2, 148.8, 147.8, 138.4, 132.4, 129.1, 128.3, 121.5, 117.9, 110.9, 109.7, 84.7, 73.4, 68.7, 68.0, 56.0, 55.8, 44.3, 41.6, 34.1, 33.5, 29.6, 28.5, 20.5; IR (neat): 2932, 1689, 1517, 1441,

1392, 1250, 1149, 1025, 756, 729, 690 cm^{-1}; HRMS (m/z) Calcd for ($C_{27}H_{28}NO_3$) ($[M+H]^+$): 414.2064; found: 414.2066.

To a solution of **17l-S2** (41.3 mg, 0.1 mmol, 1.0 equiv) in THF (1 mL) was added Rh(acac)(cod) (0.62 mg, 0.002 mmol, 2 mol %). Then $PhSiH_3$ (0.05 mL, 0.4 mmol, 4.0 equiv) was added dropwise. The resulting solution was allowed to stir at 50 °C for 0.5 h. The solvent was removed under reduced pressure. Purification by column chromatography (33% EtOAc in hexanes containing 5% TEA) gave **18l** as a yellow oil (37.6 mg, 94% yield); R_f= 0.42 (50% EtOAc in hexanes containing 5% TEA); ^{1}H NMR (500 MHz, $CDCl_3$): δ 7.43 (d, *J* = 7.0 Hz, 2H), 7.37–7.23 (m, 3H), 7.10–7.00 (m, 2H), 6.83 (d, *J* = 8.0 Hz, 1H), 3.95 (s, 3H), 3.80 (s, 3H), 3.20 (td, *J* = 9.0, 3.5 Hz, 1H), 2.99 (d, *J* = 4.5 Hz, 1H), 2.86 (q, *J* = 4.5 Hz, 1H), 2.47 (q, *J* = 9.0 Hz, 1H), 2.42 (s, 3H), 2.12–1.97 (m, 2H), 1.97–1.77 (m, 4H), 1.62–1.48 (m, 2H); ^{13}C NMR (125 MHz, $CDCl_3$): δ 148.6, 147.2, 139.5, 132.4, 128.6, 128.2, 122.1, 119.0, 111.2, 110.6, 86.7, 75.4, 74.0, 72.9, 66.7, 56.2, 55.7, 53.8, 47.9, 41.5, 40.1, 33.5, 29.8, 25.9, 19.7; IR (neat): 2933, 1736, 1517, 1441, 1237, 1146, 1027, 755, 729, 689 cm^{-1}; HRMS (m/z) Calcd for ($C_{27}H_{30}NO_2$) ($[M+H]^+$): 400.2271; found: 400.2277.

X. References

1. R. Frei, M. D. Wodrich, D. P. Hari, P.-A. Borin, C. Chauvier and J. Waser, *J. Am. Chem. Soc.,* 2014, **136**, 16563–16573.
2. H. Takahata, T. Takamatsu, Y. Chen, N. Ohkubo, T. Yamazaki and T. Momose, *J. Org. Chem.,* 1990, **55**, 3792–3797.
3. H. Takahata, T. Takamatsu and T. Yamazaki, *J. Org. Chem.,* 1989, **54**, 4812–4822.
4. B. Lu, J. Wu and N. Yoshikai, *J. Am. Chem. Soc.,* 2014, **136**, 11598–11601.
5. G. Chelucci, F. Capitta and S. Baldino, *Tetrahedron,* 2008, **64**, 10250–10257.

X. NMR Spectra.

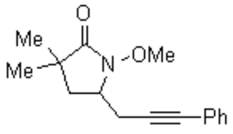

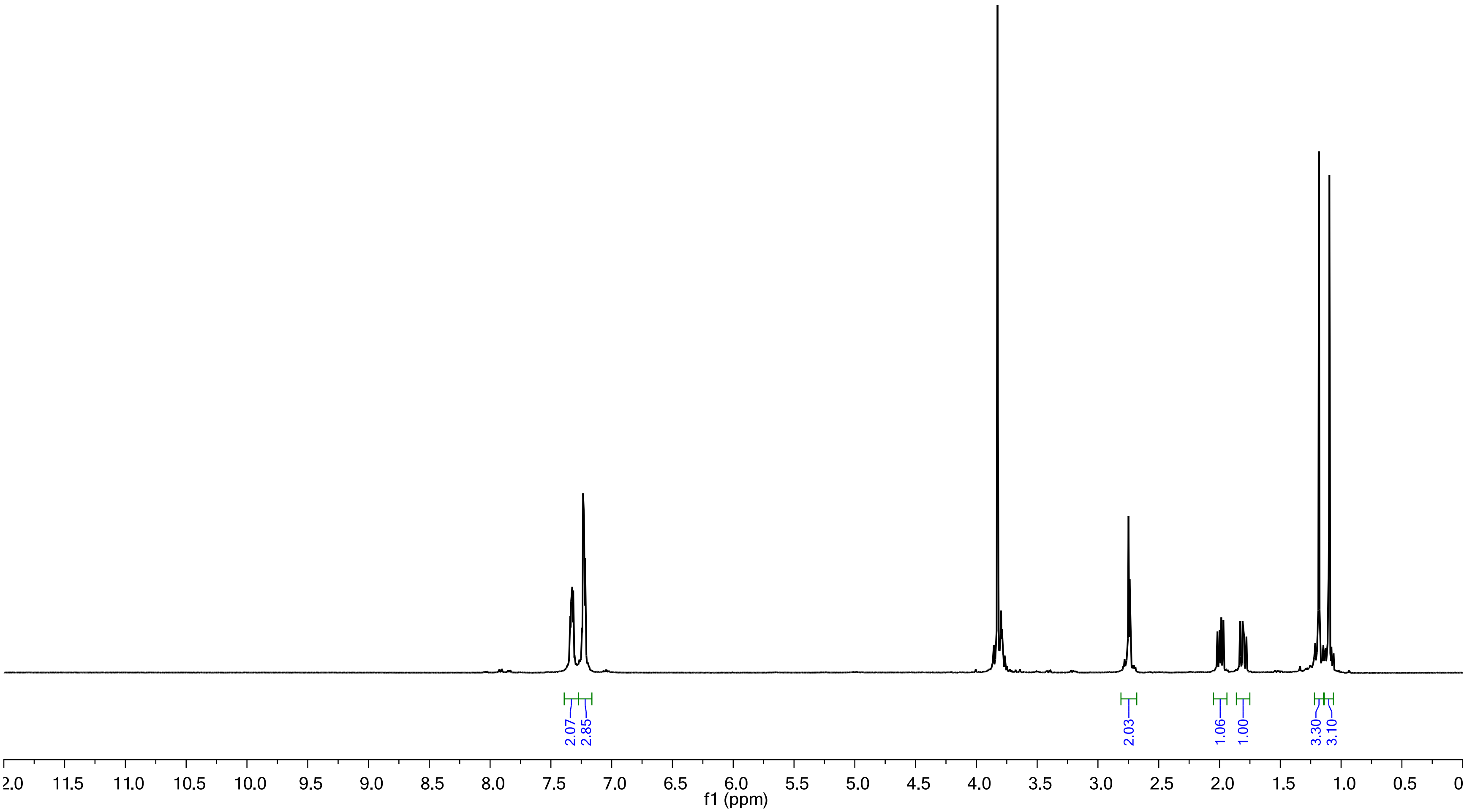

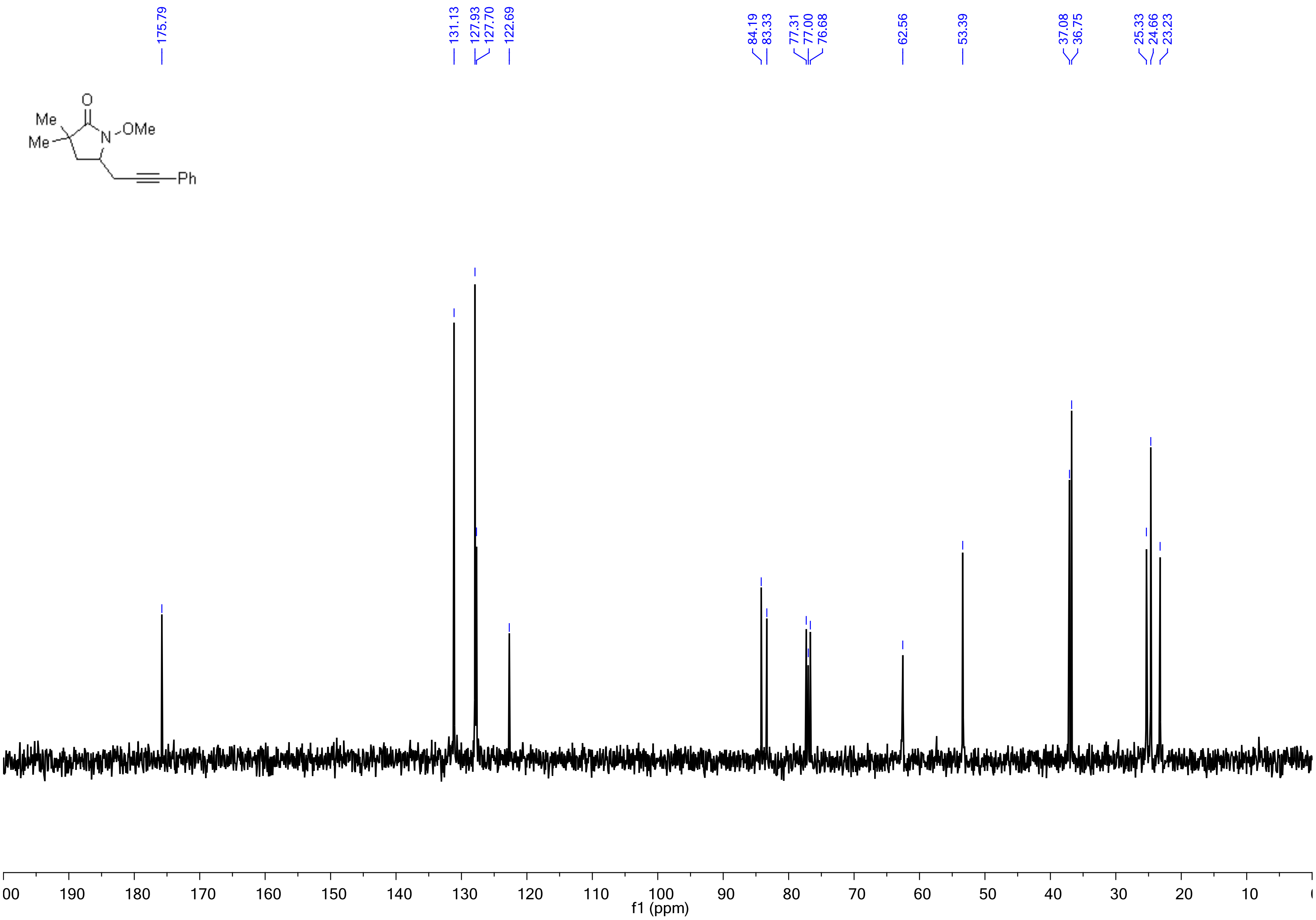
175.79
131.13
127.93
127.70
122.69
84.19
83.33
77.31
77.00
76.68
62.56
53.39
37.08
36.75
25.33
24.66
23.23
O
Me
Me
N
OMe
Ph
f1 (ppm)

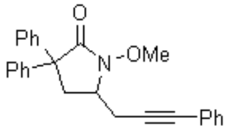
O
Ph
Ph
N
-OMe
Ph

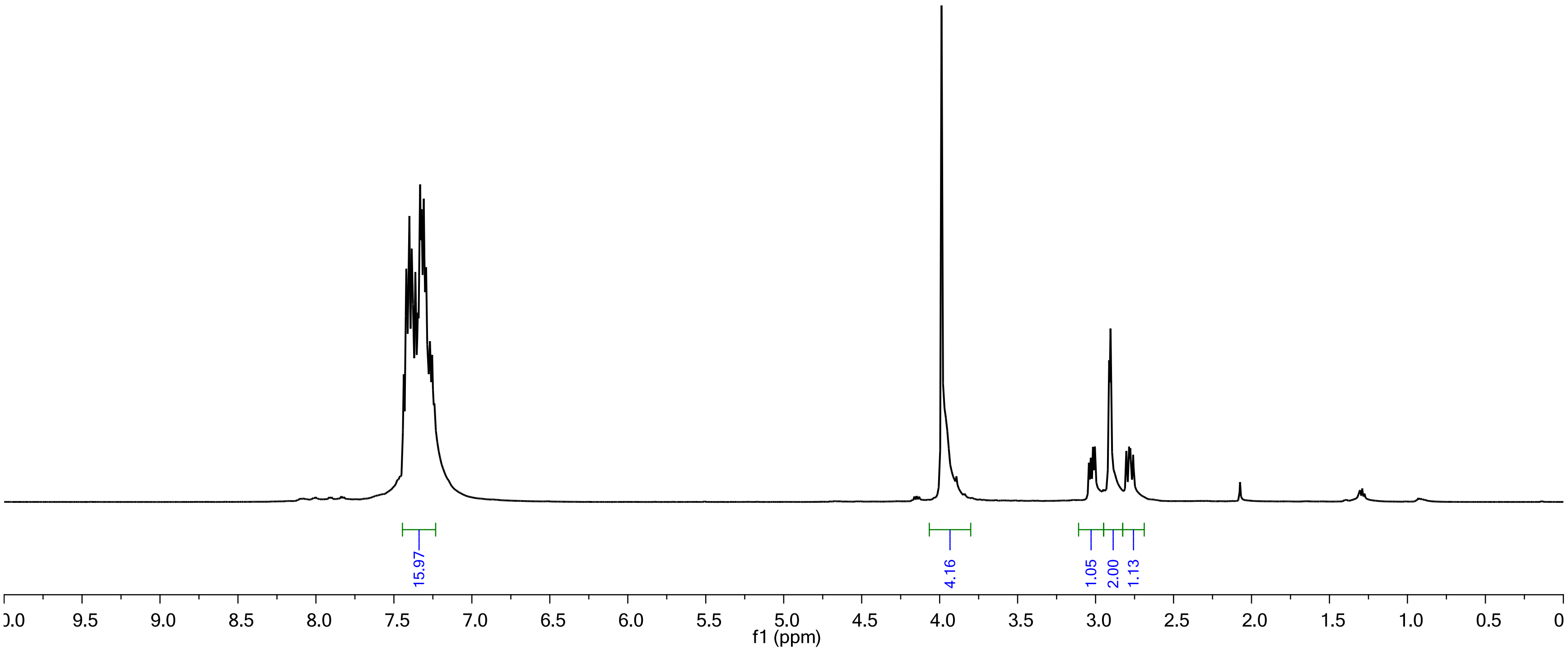
15.97
4.16
1.05
2.00
1.13
f1 (ppm)

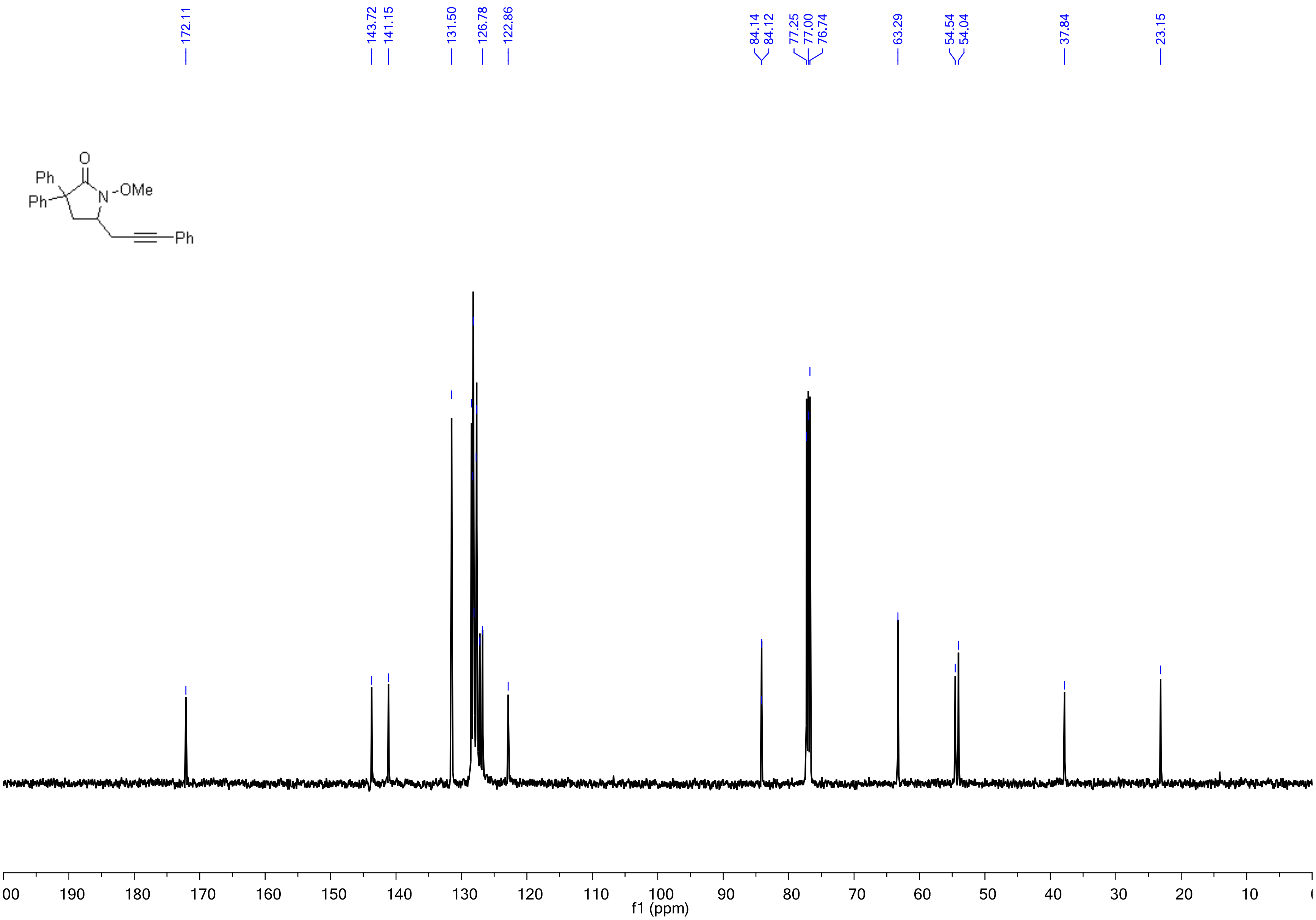

172.11
143.72
141.15
131.50
126.78
122.86
84.14
84.12
77.25
77.00
76.74
63.29
54.54
54.04
37.84
23.15
O
Ph
Ph
N
OMe
Ph
f1 (ppm)

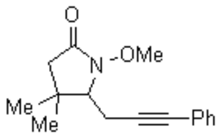
O
N
OMe
Me
Me
Ph

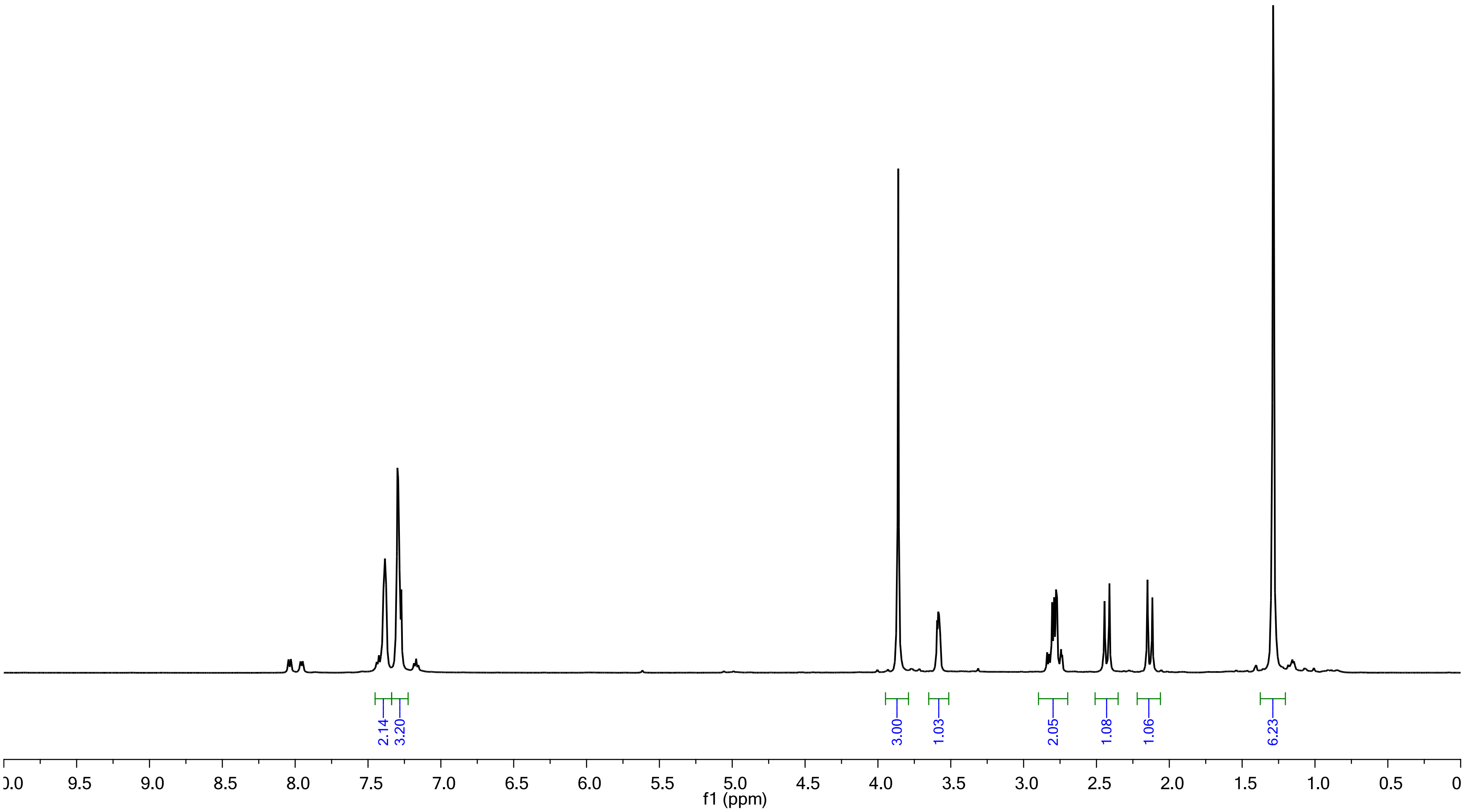
2.14
3.20
3.00
1.03
2.05
1.08
1.06
6.23
0.0
9.5
9.0
8.5
8.0
7.5
7.0
6.5
6.0
5.5
5.0
4.5
4.0
3.5
3.0
2.5
2.0
1.5
1.0
0.5
0
f1 (ppm)

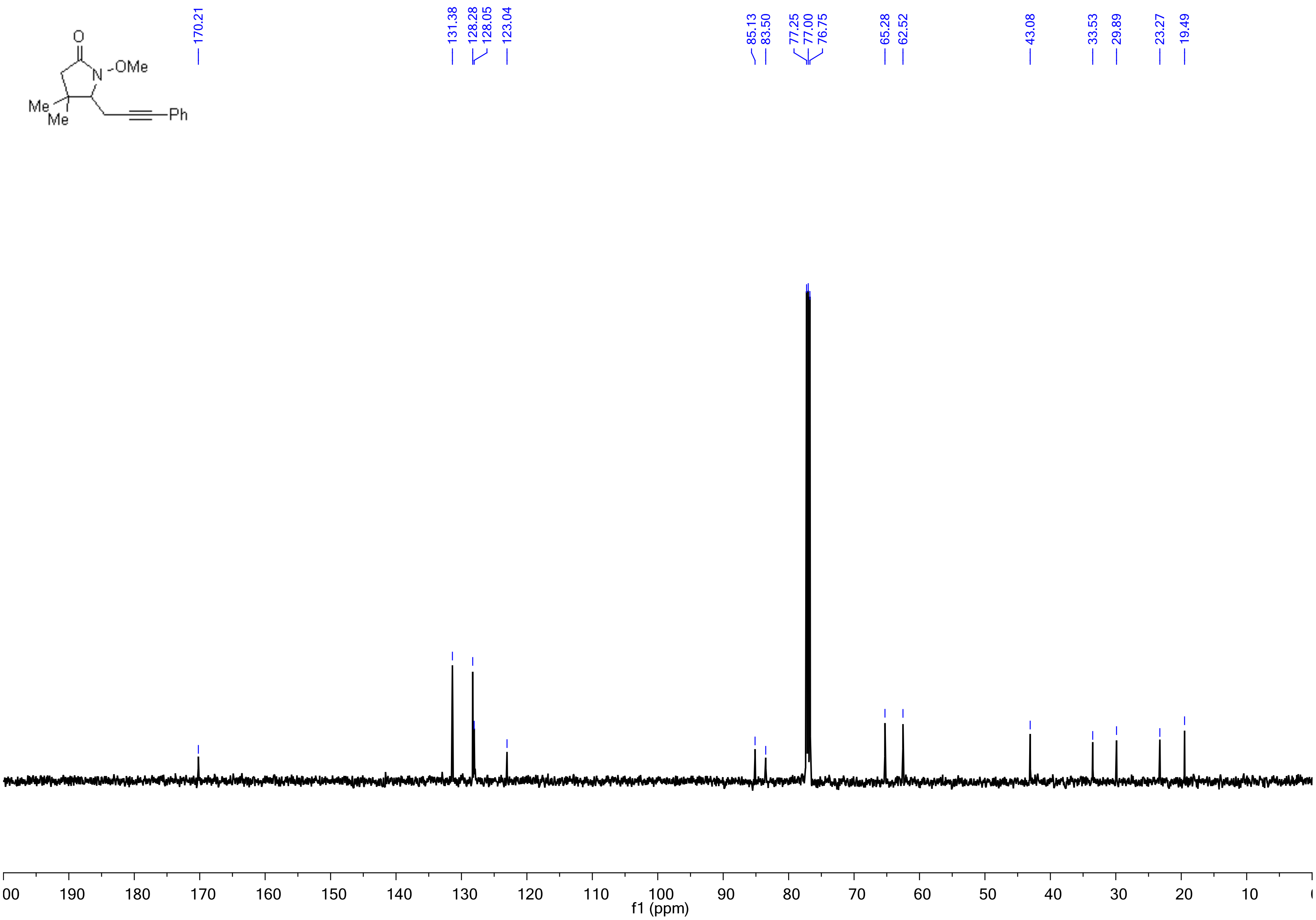
O
N
OMe
Me
Me
Ph
170.21
131.38
128.28
128.05
123.04
85.13
83.50
77.25
77.00
76.75
65.28
62.52
43.08
33.53
29.89
23.27
19.49
f1 (ppm)

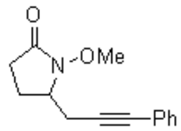
O
N
OMe
Ph

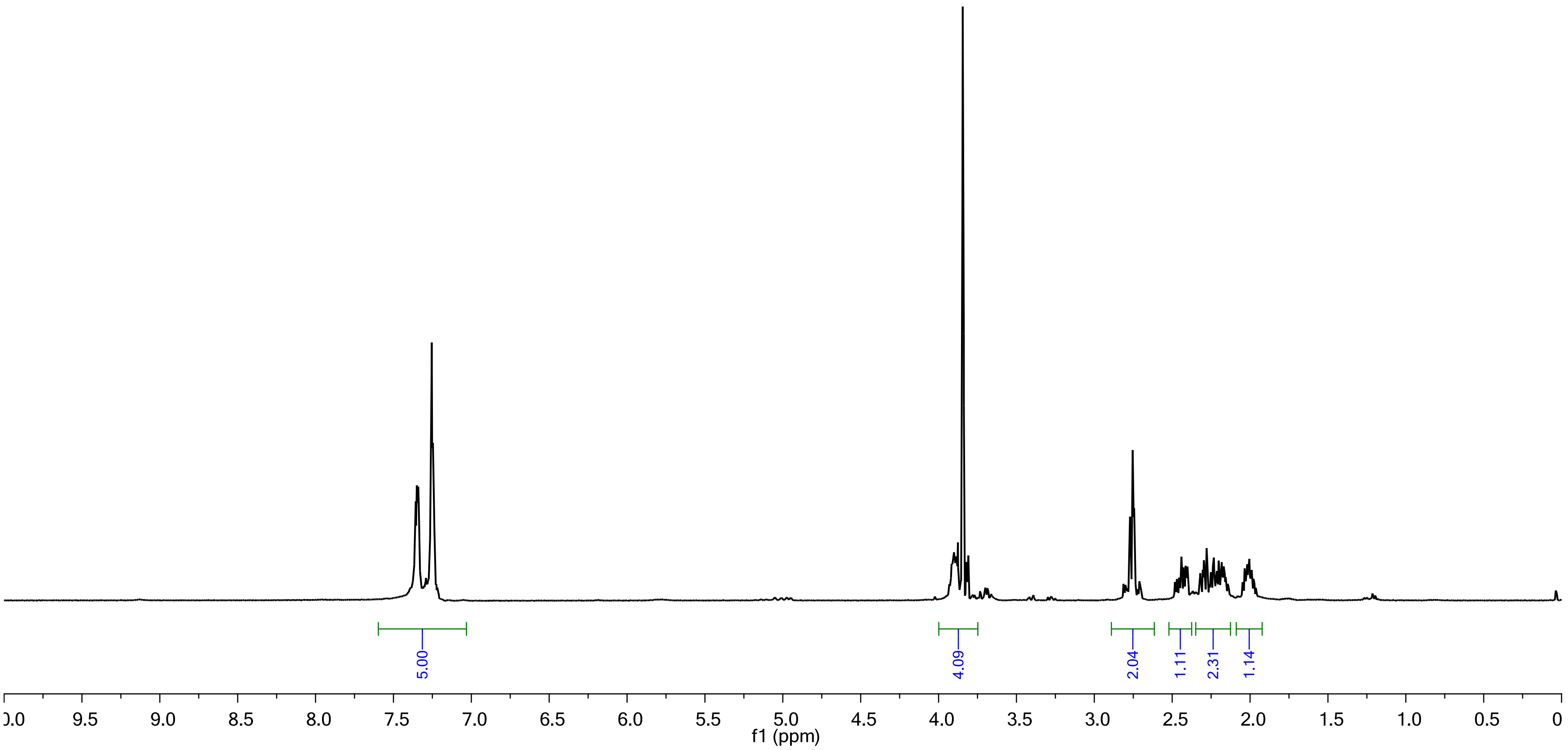
5.00
4.09
2.04
1.11
2.31
1.14
9.5
9.0
8.5
8.0
7.5
7.0
6.5
6.0
5.5
5.0
4.5
4.0
3.5
3.0
2.5
2.0
1.5
1.0
0.5
0
f1 (ppm)

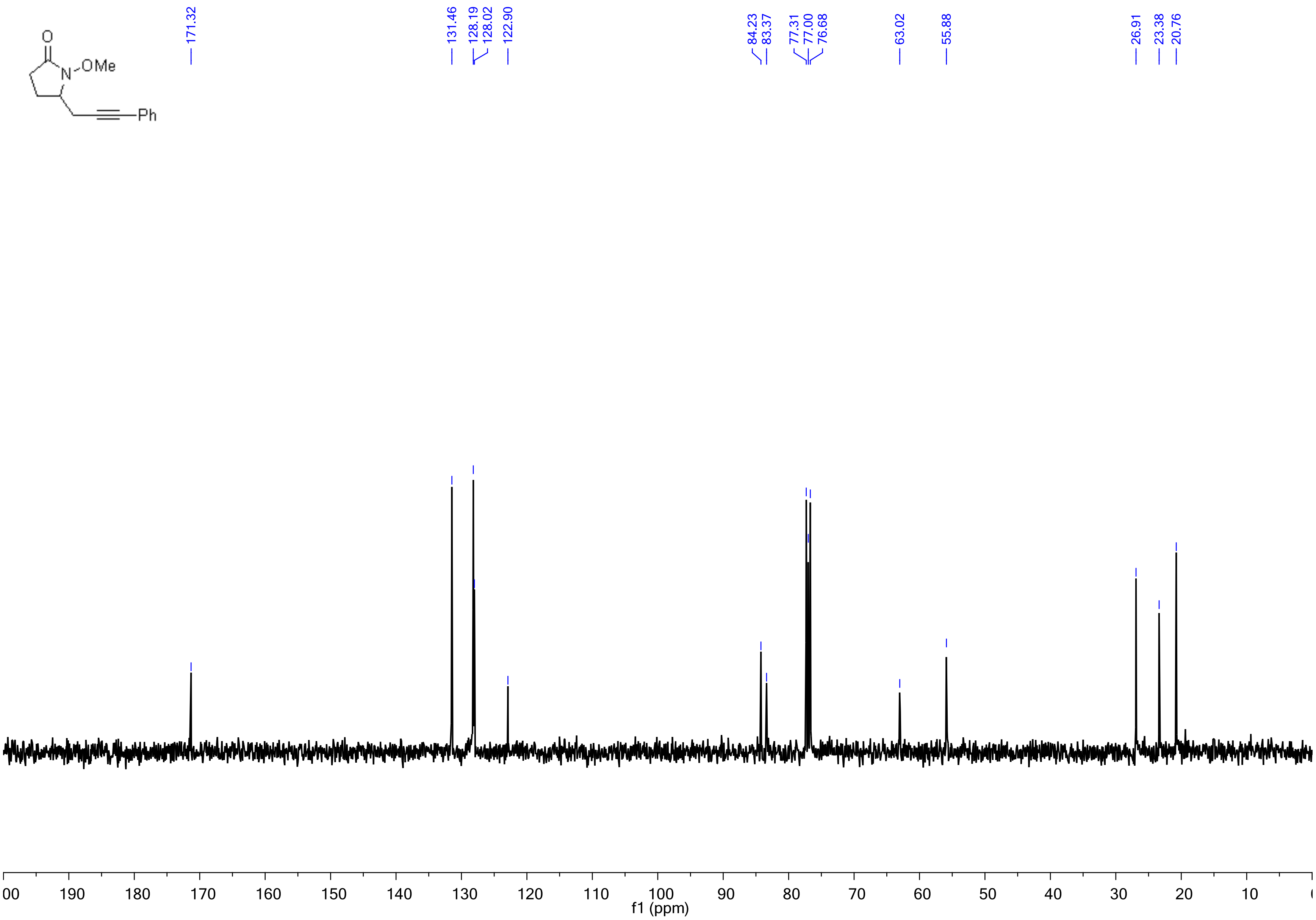

O
N
OMe
Ph
171.32
131.46
128.19
128.02
122.90
84.23
83.37
77.31
77.00
76.68
63.02
55.88
26.91
23.38
20.76
00
190
180
170
160
150
140
130
120
110
100
90
80
70
60
50
40
30
20
10
f1 (ppm)

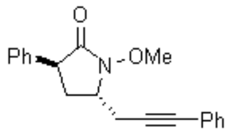
O
Ph
N
OMe
Ph

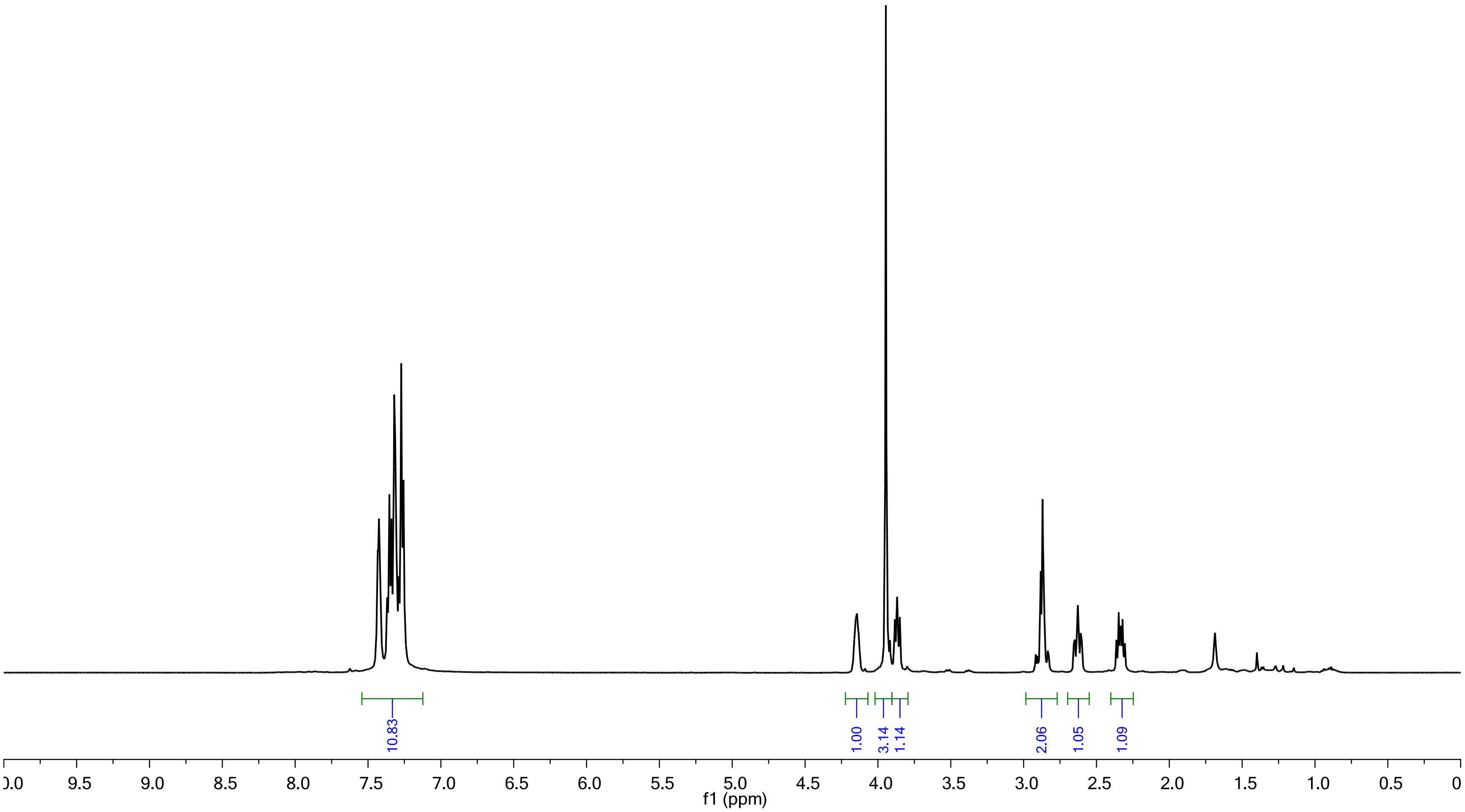
10.83
1.00
3.14
1.14
2.06
1.05
1.09
f1 (ppm)

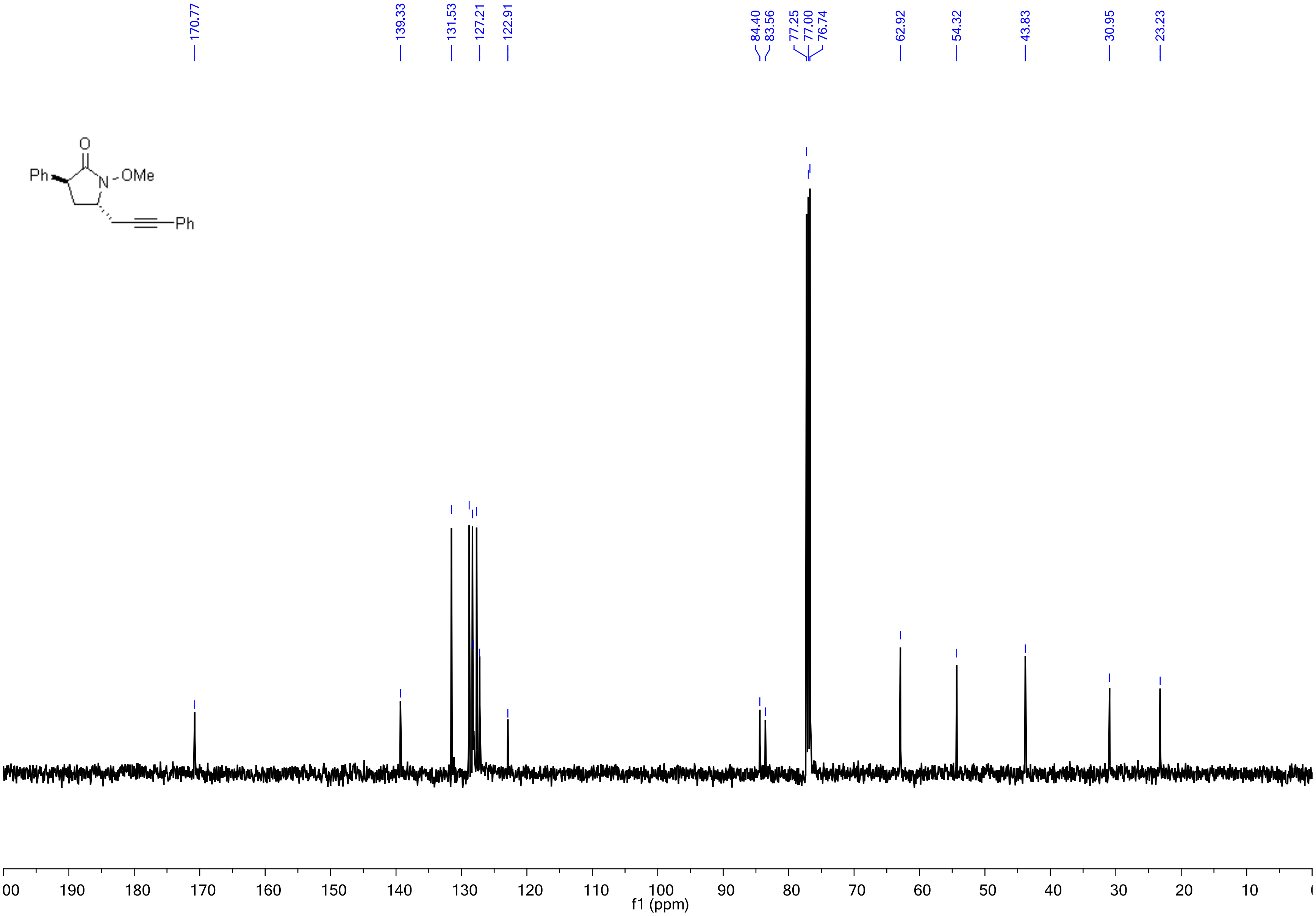

170.77
139.33
131.53
127.21
122.91
84.40
83.56
77.25
77.00
76.74
62.92
54.32
43.83
30.95
23.23
O
Ph
N
OMe
Ph
f1 (ppm)

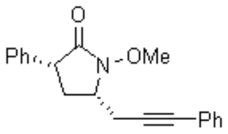
Ph
N
OMe
O
Ph

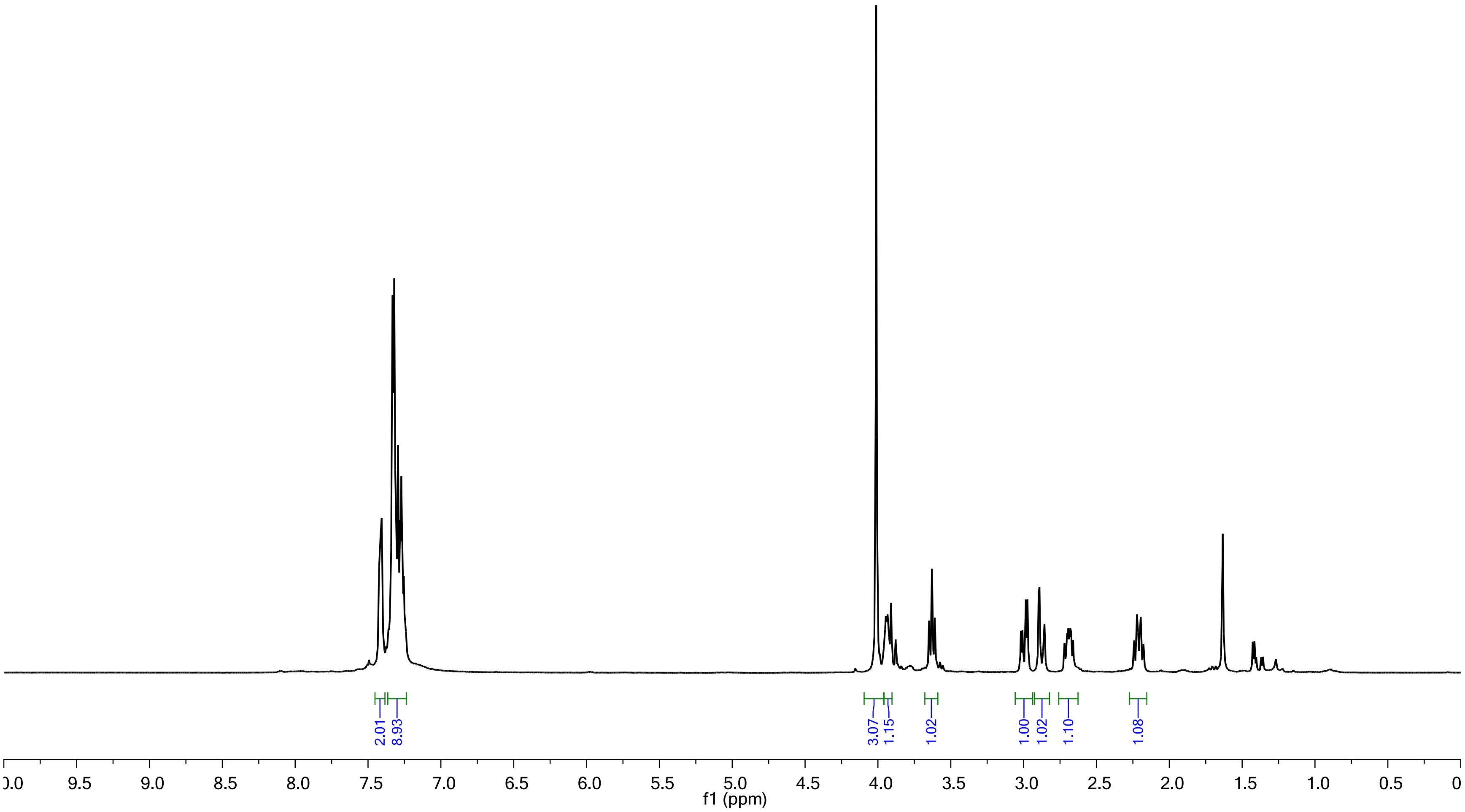
2.01
8.93
3.07
1.15
1.02
1.00
1.02
1.10
1.08
9.5
9.0
8.5
8.0
7.5
7.0
6.5
6.0
5.5
5.0
4.5
4.0
3.5
3.0
2.5
2.0
1.5
1.0
0.5
f1 (ppm)

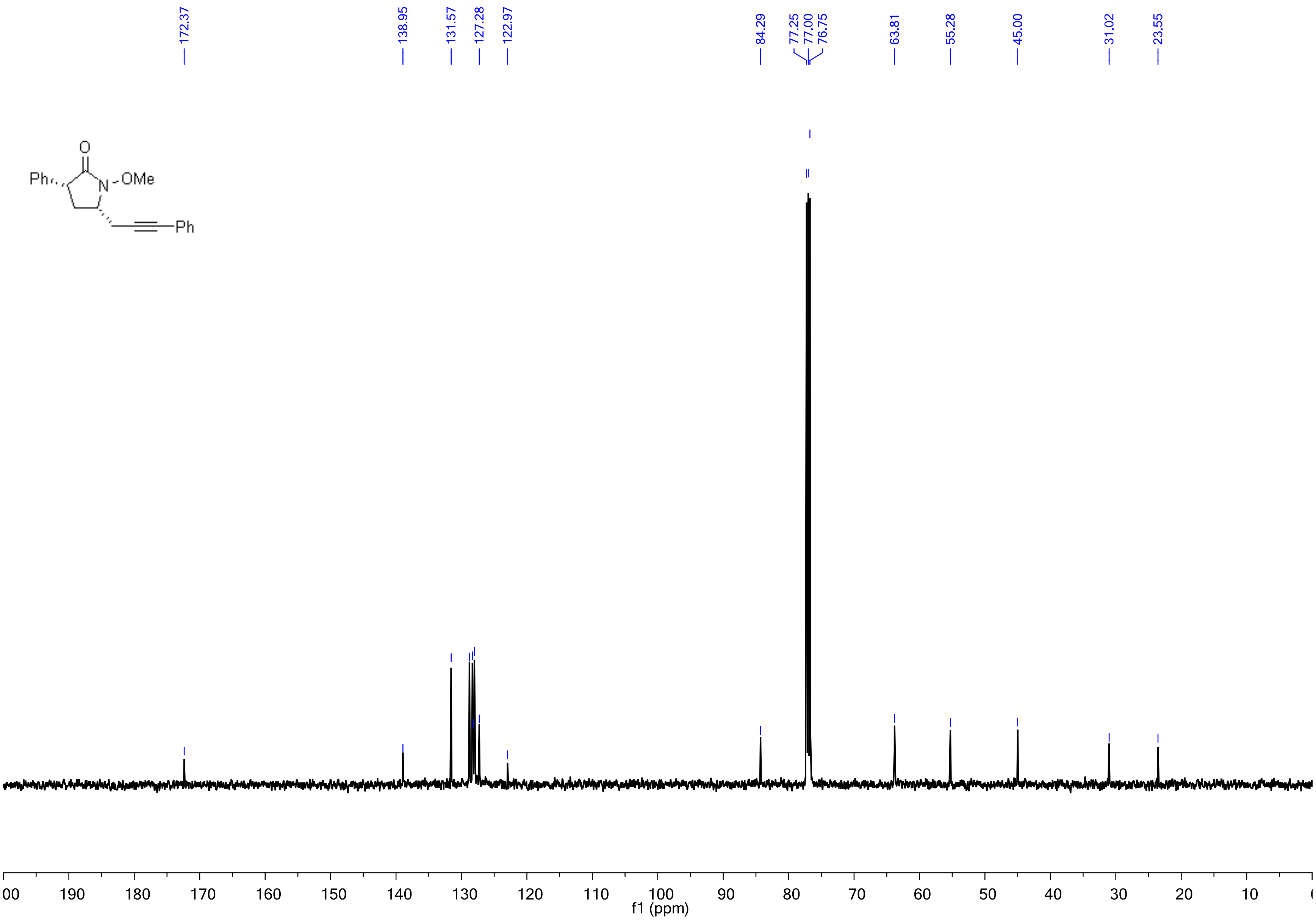
172.37
138.95
131.57
127.28
122.97
84.29
77.25
77.00
76.75
63.81
55.28
45.00
31.02
23.55
O
Ph
N
OMe
Ph
00
190
180
170
160
150
140
130
120
110
100
90
80
70
60
50
40
30
20
10
f1 (ppm)

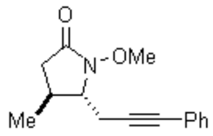

O
N
OMe
Me
Ph

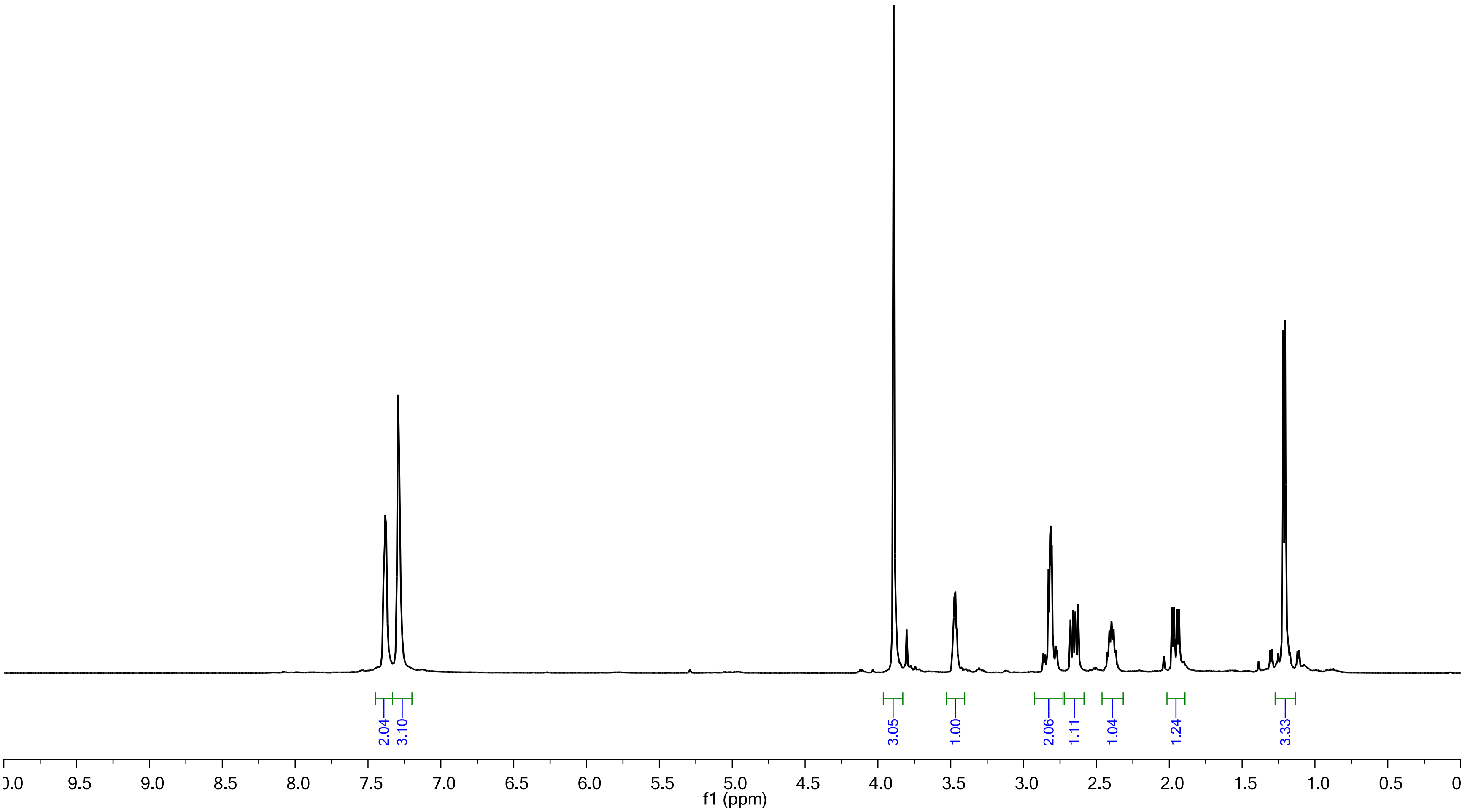

2.04
3.10
3.05
1.00
2.06
1.11
1.04
1.24
3.33
10.0
9.5
9.0
8.5
8.0
7.5
7.0
6.5
6.0
5.5
5.0
4.5
4.0
3.5
3.0
2.5
2.0
1.5
1.0
0.5
0
f1 (ppm)

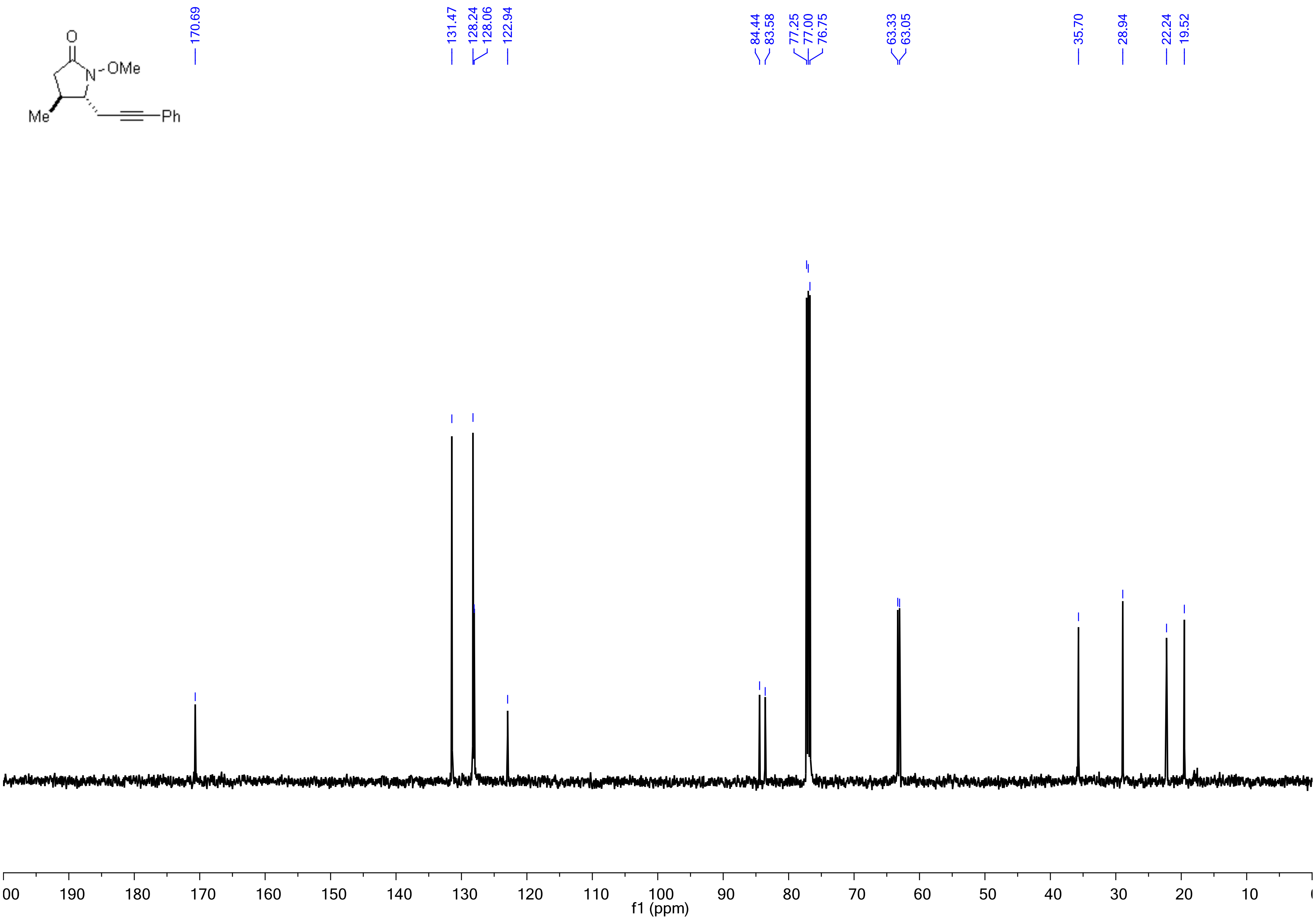
O
N
OMe
Me
Ph
170.69
131.47
128.24
128.06
122.94
84.44
83.58
77.25
77.00
76.75
63.33
63.05
35.70
28.94
22.24
19.52
00
190
180
170
160
150
140
130
120
110
100
90
80
70
60
50
40
30
20
10
f1 (ppm)

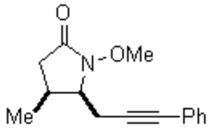

O
N
OMe
Me
Ph

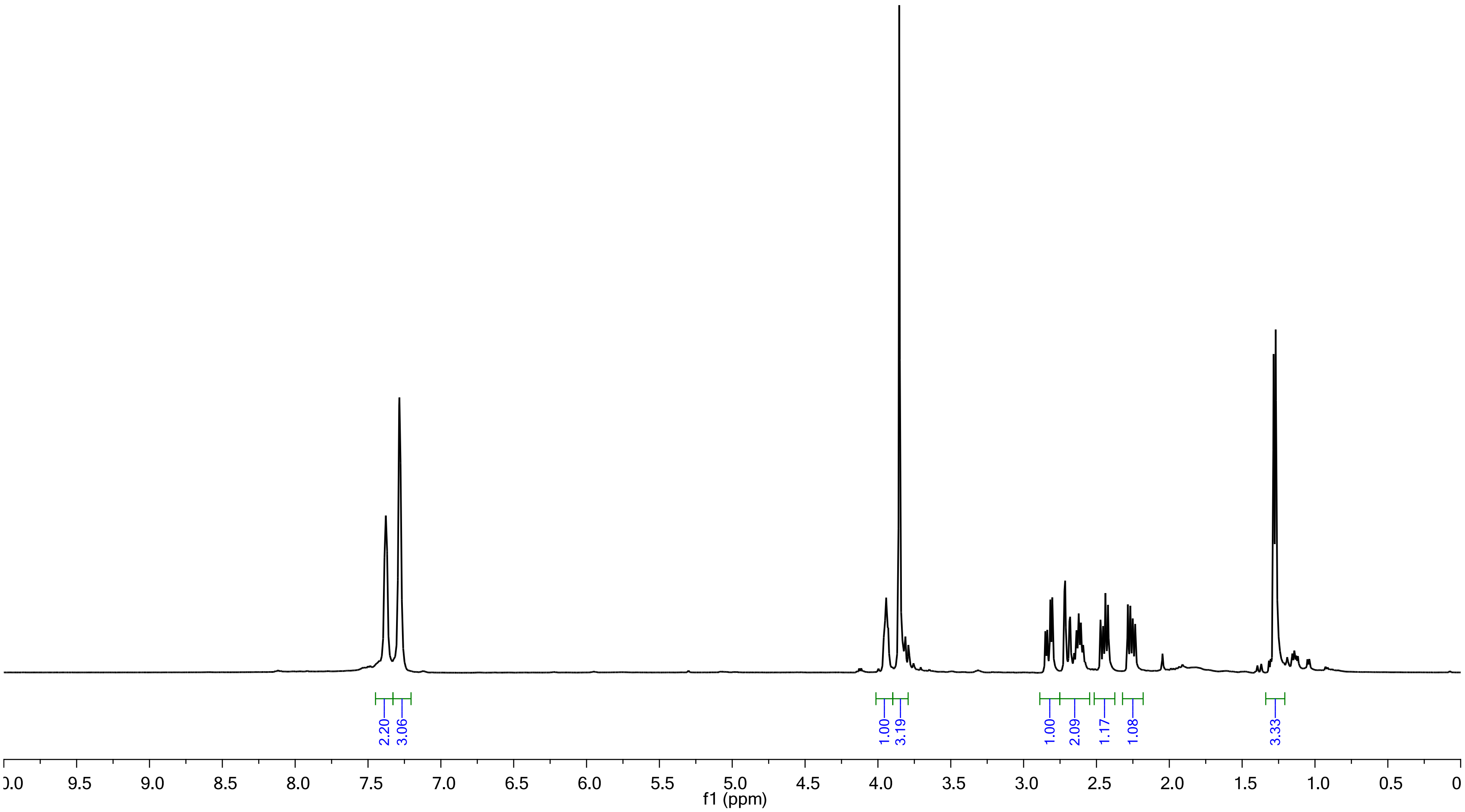

2.20
3.06
1.00
3.19
1.00
2.09
1.17
1.08
3.33
10.0
9.5
9.0
8.5
8.0
7.5
7.0
6.5
6.0
5.5
5.0
4.5
4.0
3.5
3.0
2.5
2.0
1.5
1.0
0.5
0
f1 (ppm)

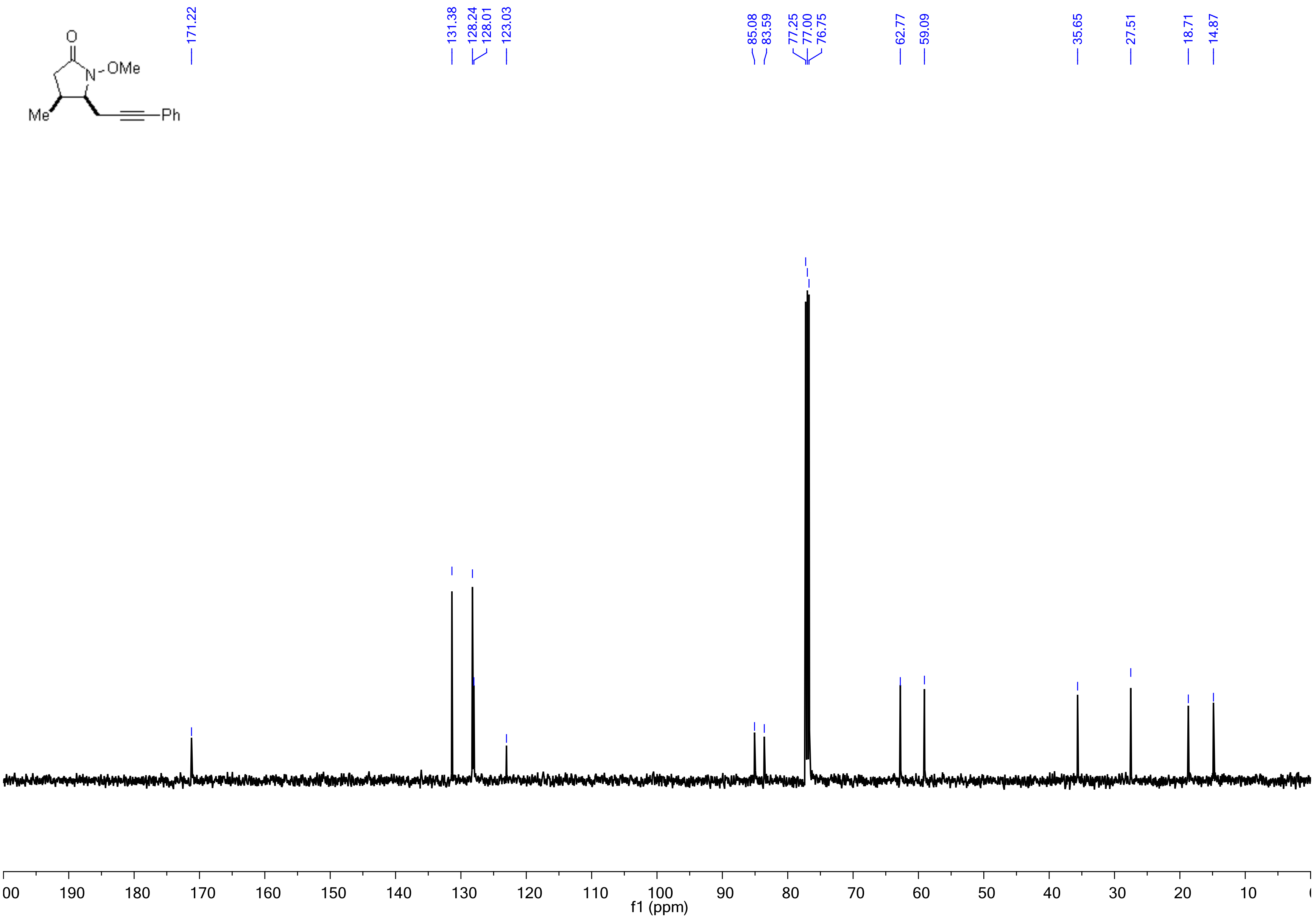
O
N
OMe
Me
Ph
171.22
131.38
128.24
128.01
123.03
85.08
83.59
77.25
77.00
76.75
62.77
59.09
35.65
27.51
18.71
14.87
f1 (ppm)

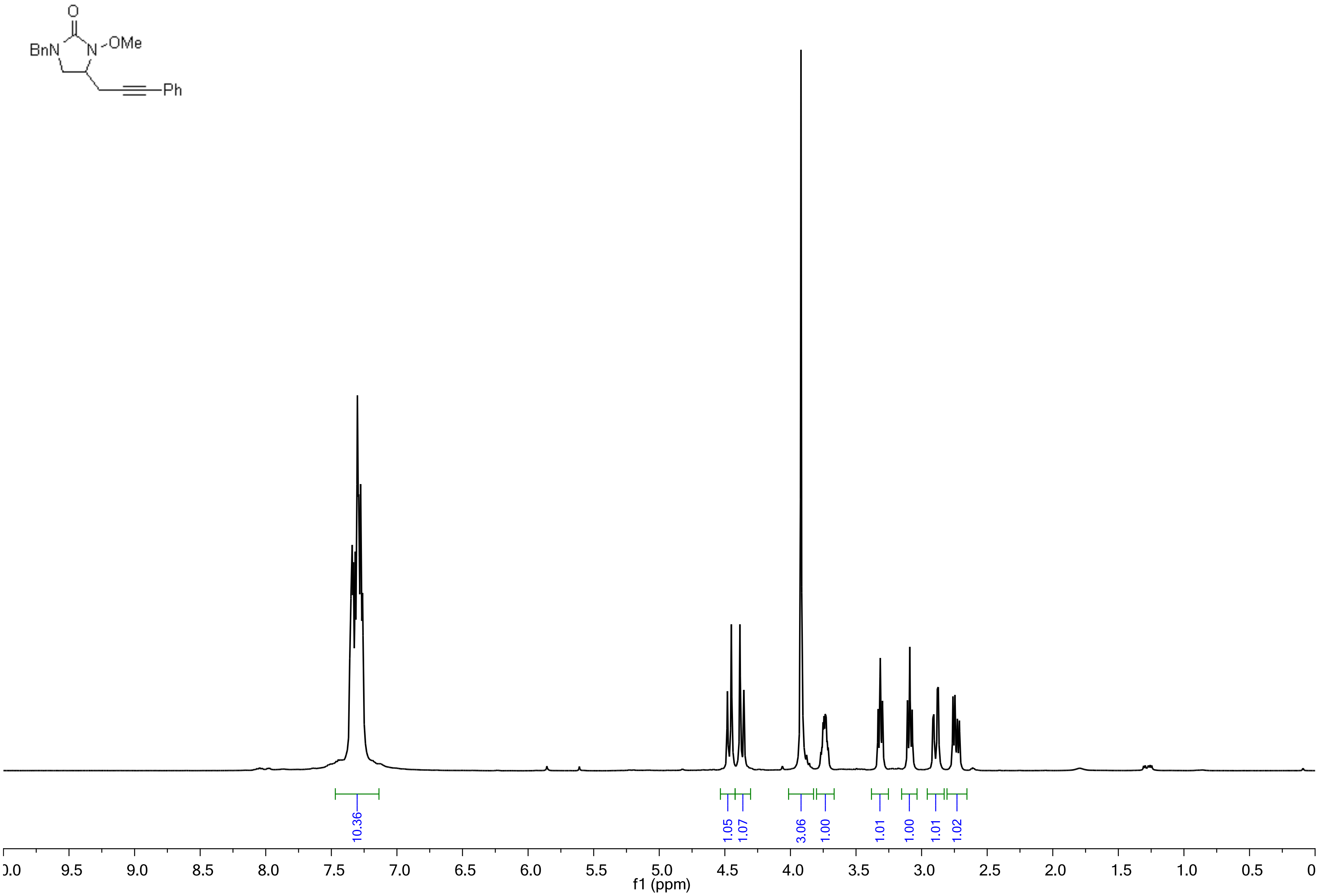
O
BnN
N
OMe
Ph
10.36
1.05
1.07
3.06
1.00
1.01
1.00
1.01
1.02
9.5
9.0
8.5
8.0
7.5
7.0
6.5
6.0
5.5
5.0
4.5
4.0
3.5
3.0
2.5
2.0
1.5
1.0
0.5
0
f1 (ppm)

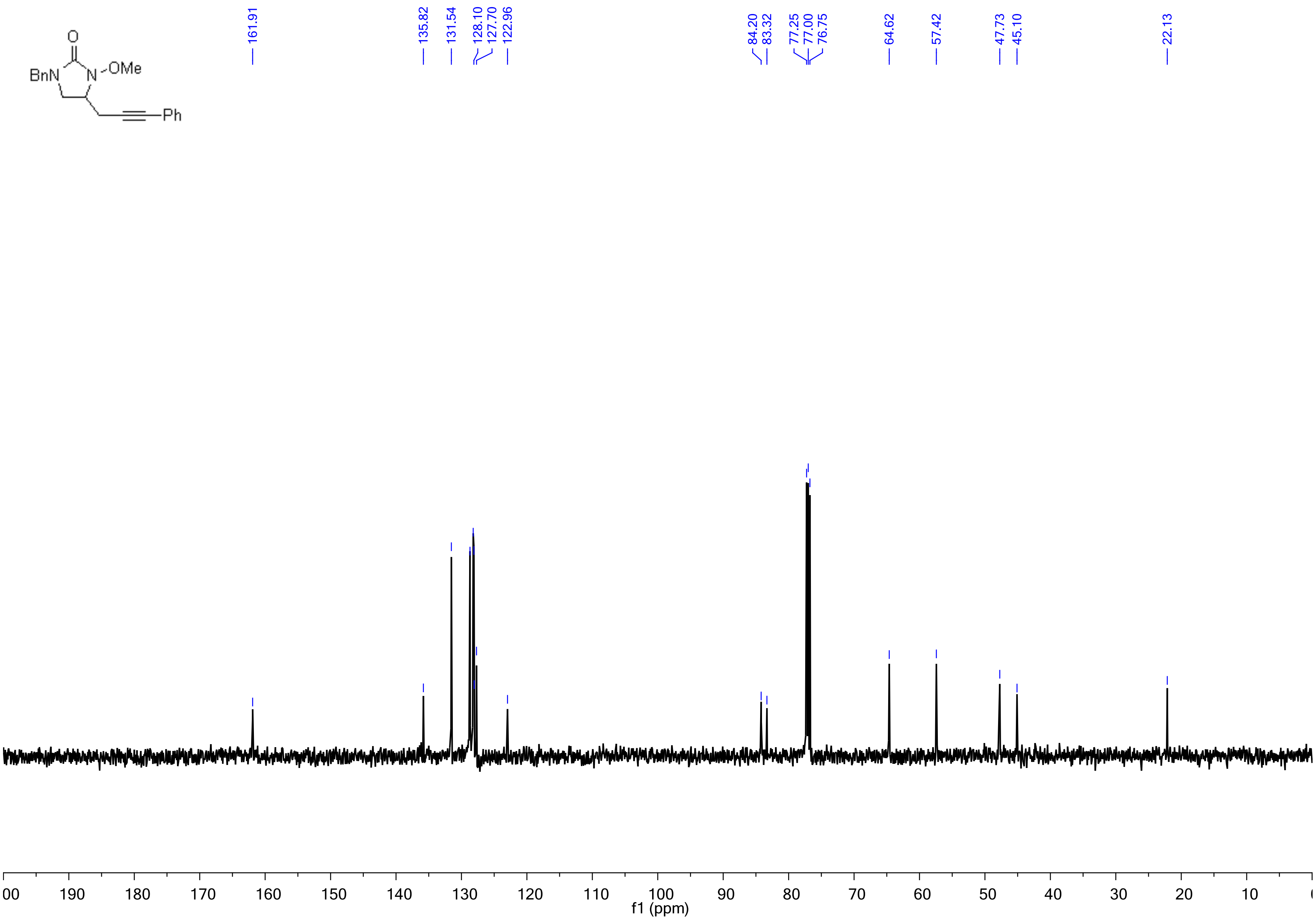

O
BnN
N
OMe
Ph
161.91
135.82
131.54
128.10
127.70
122.96
84.20
83.32
77.25
77.00
76.75
64.62
57.42
47.73
45.10
22.13
f1 (ppm)

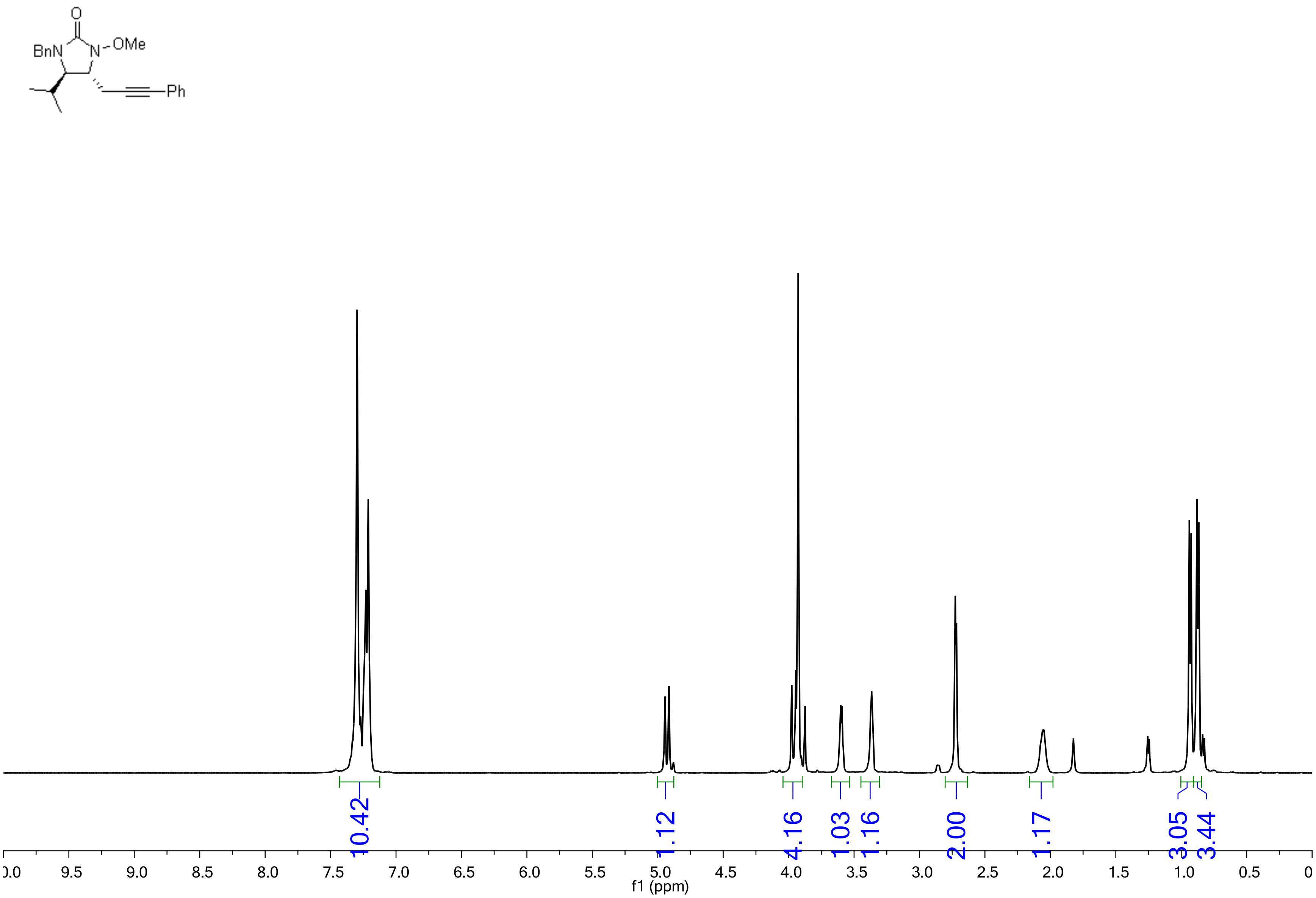

O
BnN
N
OMe
Ph
10.42
1.12
4.16
1.03
1.16
2.00
1.17
3.05
3.44
0.0
9.5
9.0
8.5
8.0
7.5
7.0
6.5
6.0
5.5
5.0
4.5
4.0
3.5
3.0
2.5
2.0
1.5
1.0
0.5
0
f1 (ppm)

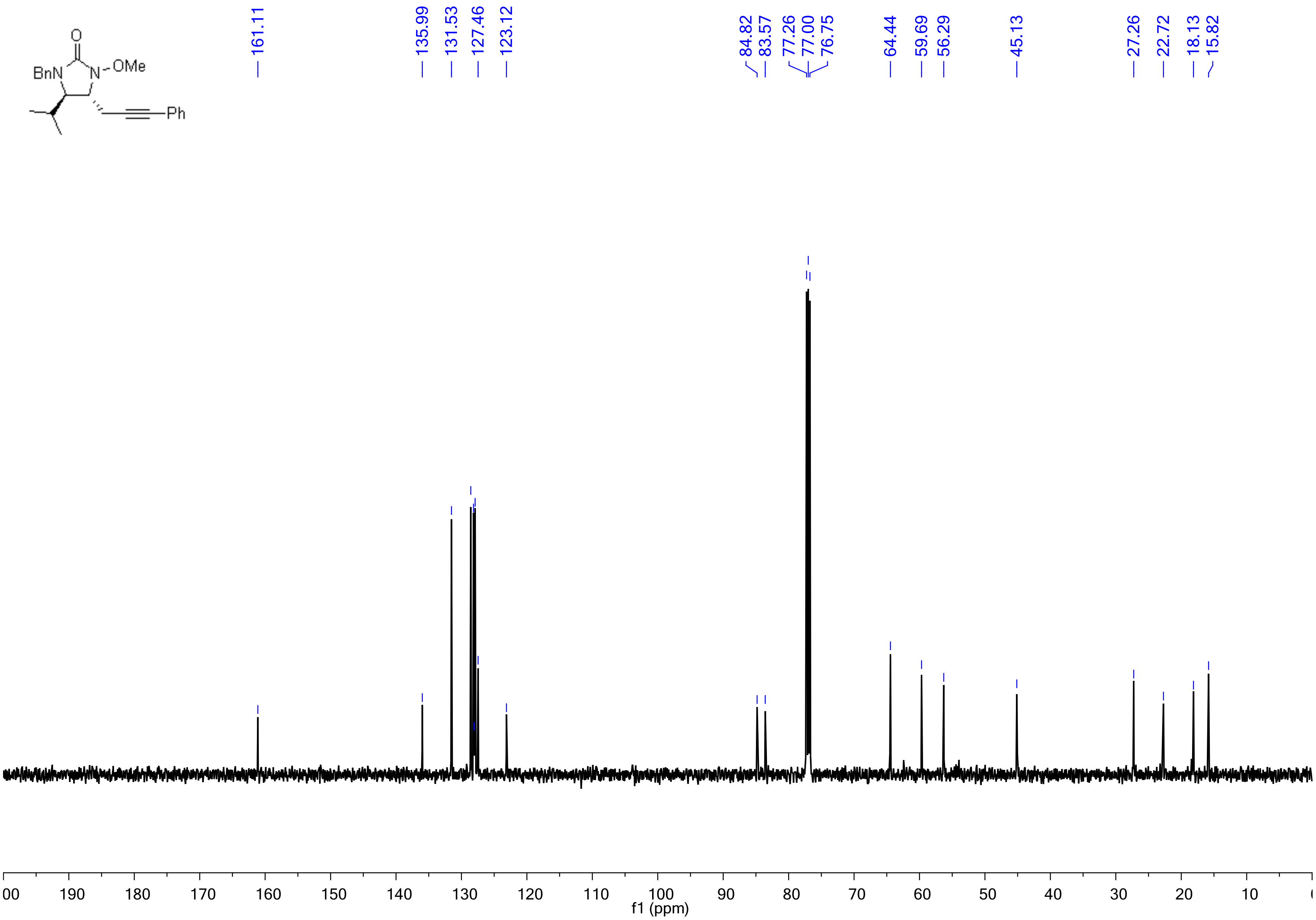
BnN
O
N-OMe
Ph
161.11
135.99
131.53
127.46
123.12
84.82
83.57
77.26
77.00
76.75
64.44
59.69
56.29
45.13
27.26
22.72
18.13
15.82
f1 (ppm)

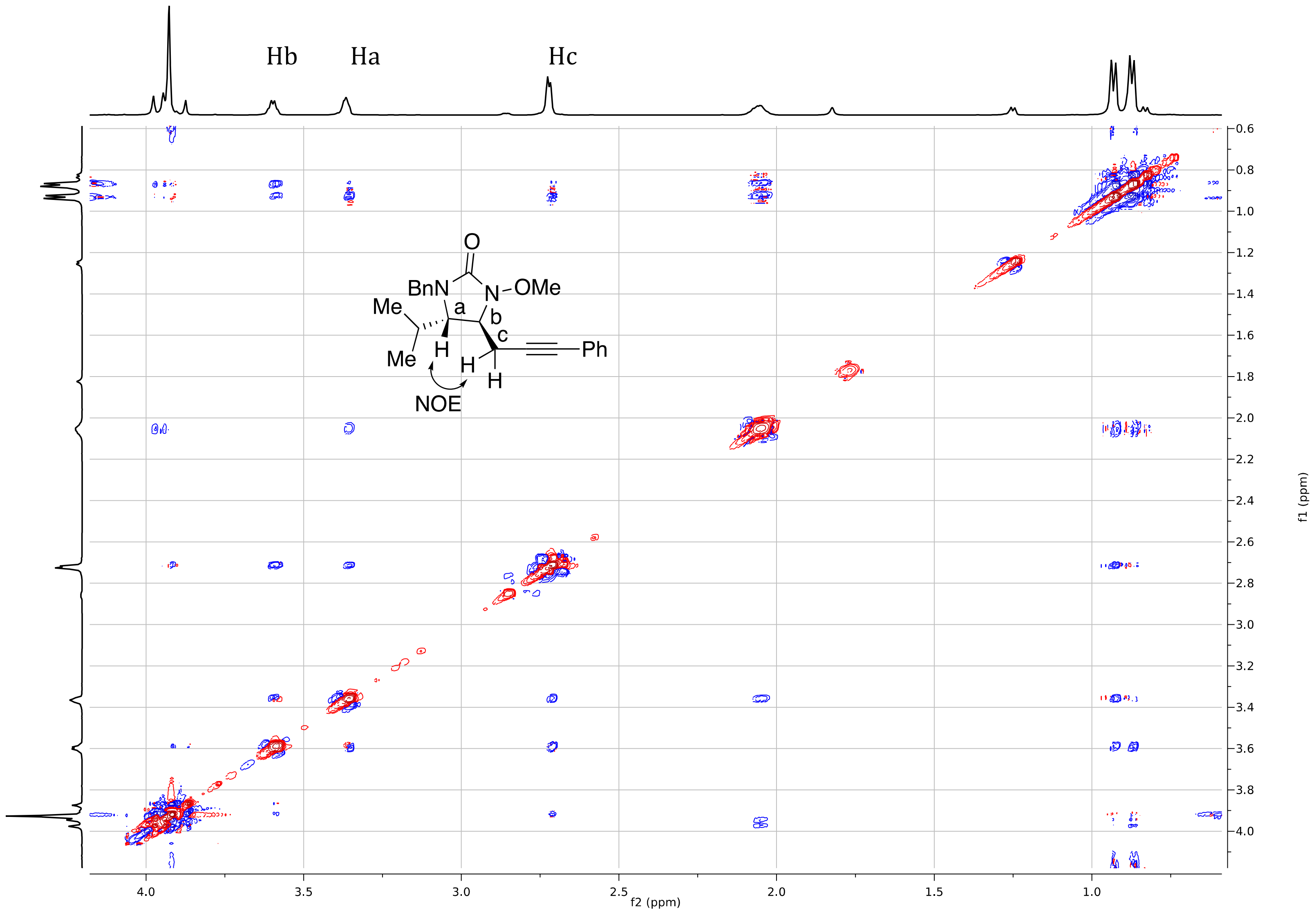
Hb
Ha
Hc
O
BnN
N
OMe
Me
a
b
c
Ph
Me
H
H
H
NOE
f2 (ppm)
f1 (ppm)

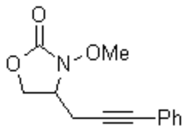

O
N
OMe
O
Ph

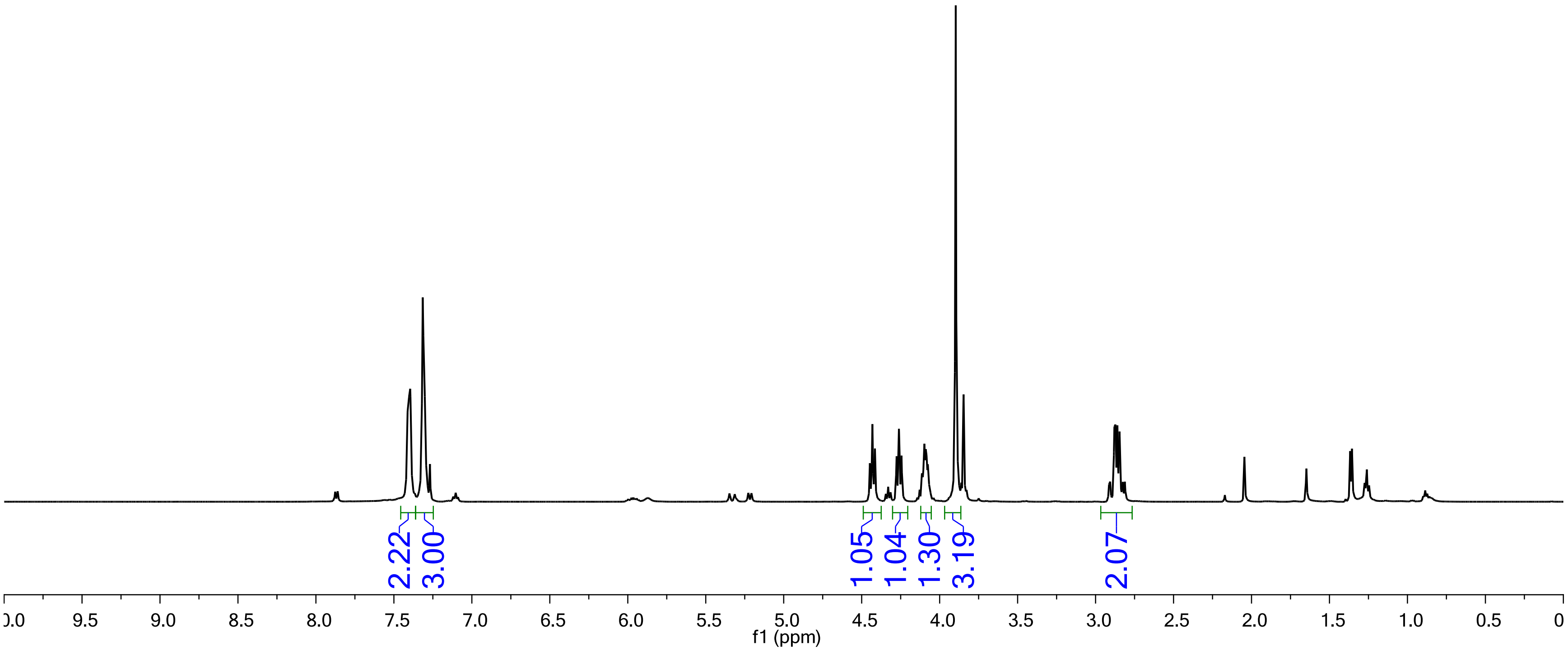

2.22
3.00
1.05
1.04
1.30
3.19
2.07
10.0
9.5
9.0
8.5
8.0
7.5
7.0
6.5
6.0
5.5
5.0
4.5
4.0
3.5
3.0
2.5
2.0
1.5
1.0
0.5
0
f1 (ppm)

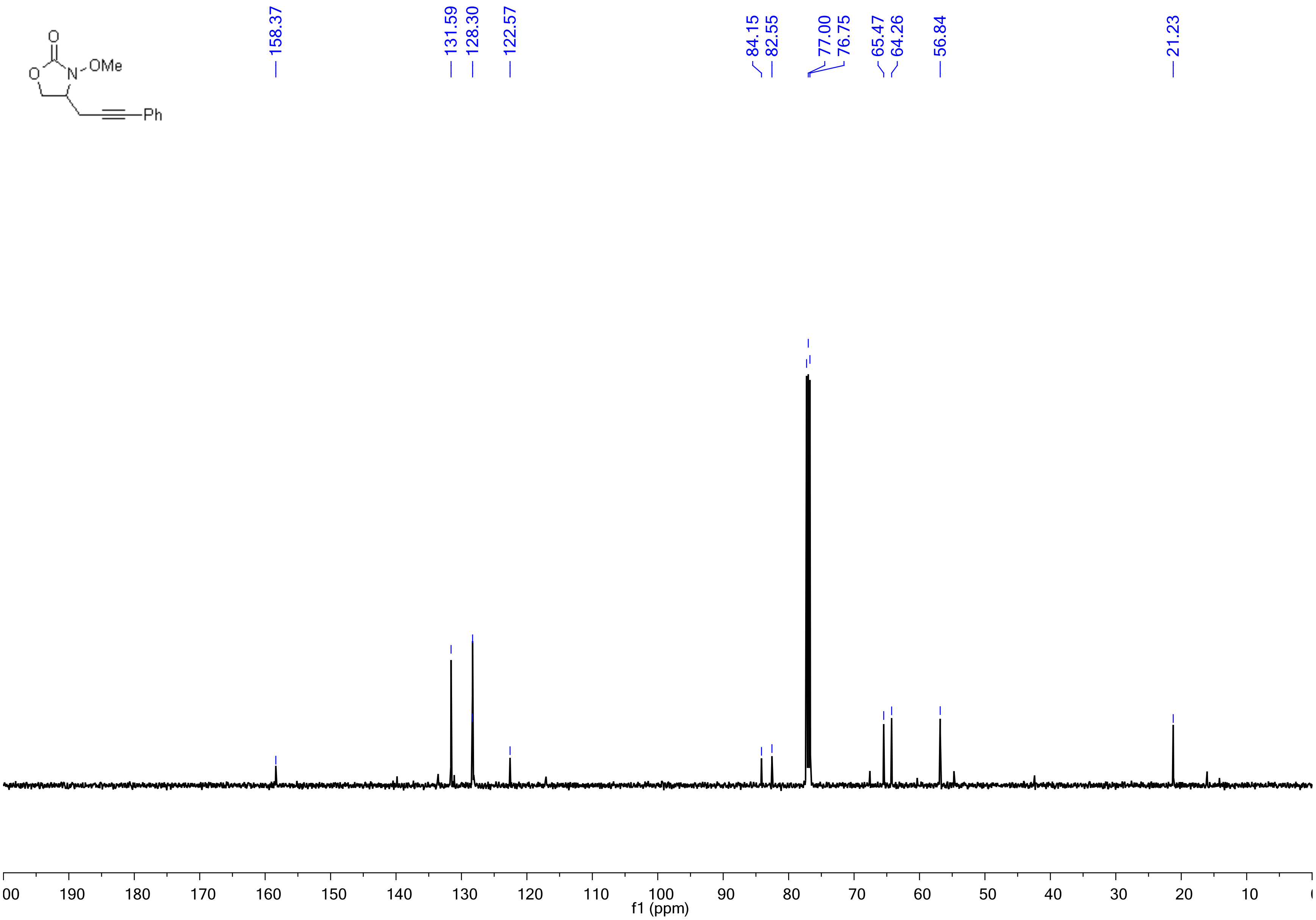

OMe
Ph
158.37
131.59
128.30
122.57
84.15
82.55
77.00
76.75
65.47
64.26
56.84
21.23
f1 (ppm)

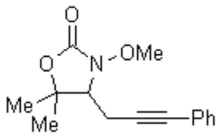
O
O
N
OMe
Me
Me
Ph

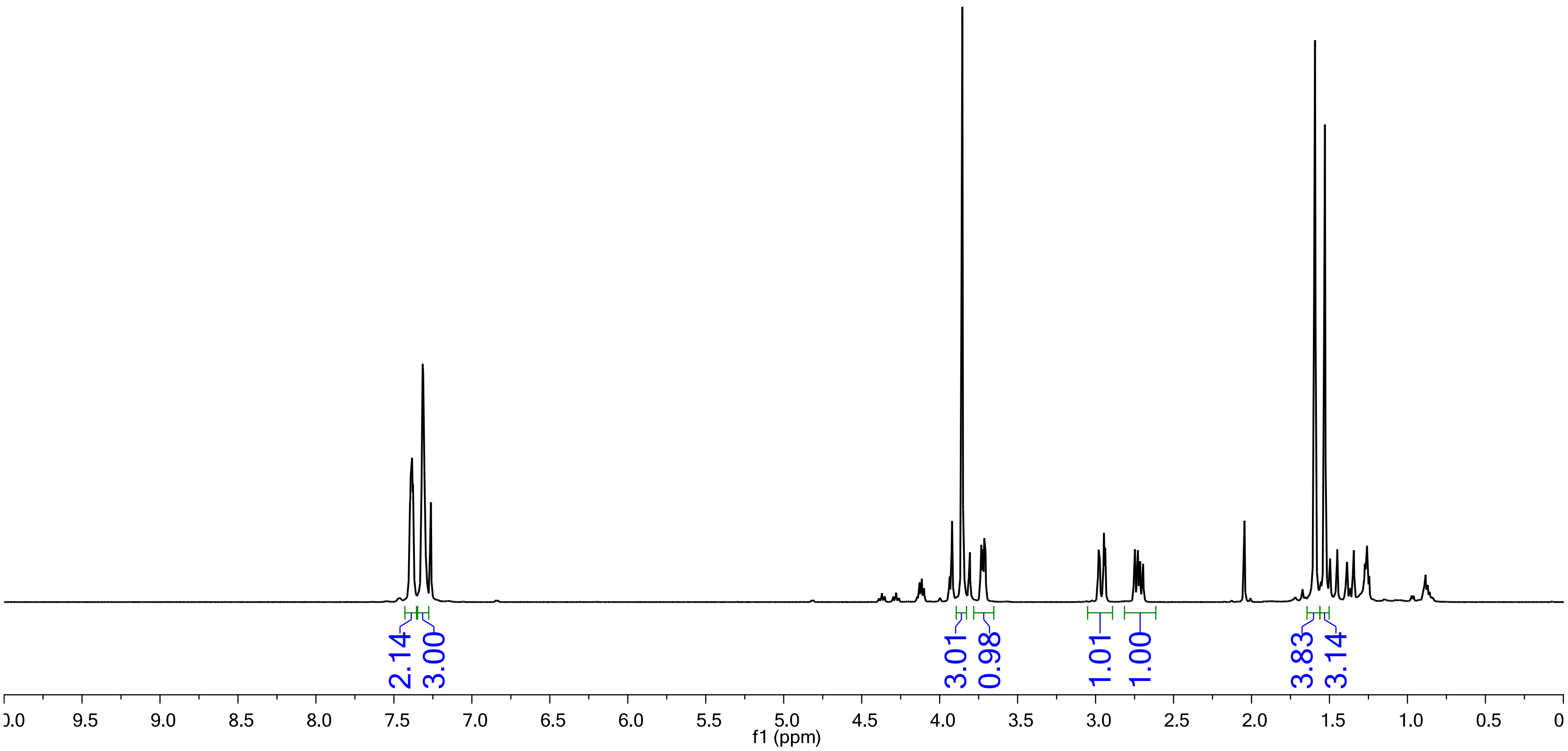
2.14
3.00
3.01
0.98
1.01
1.00
3.83
3.14
f1 (ppm)

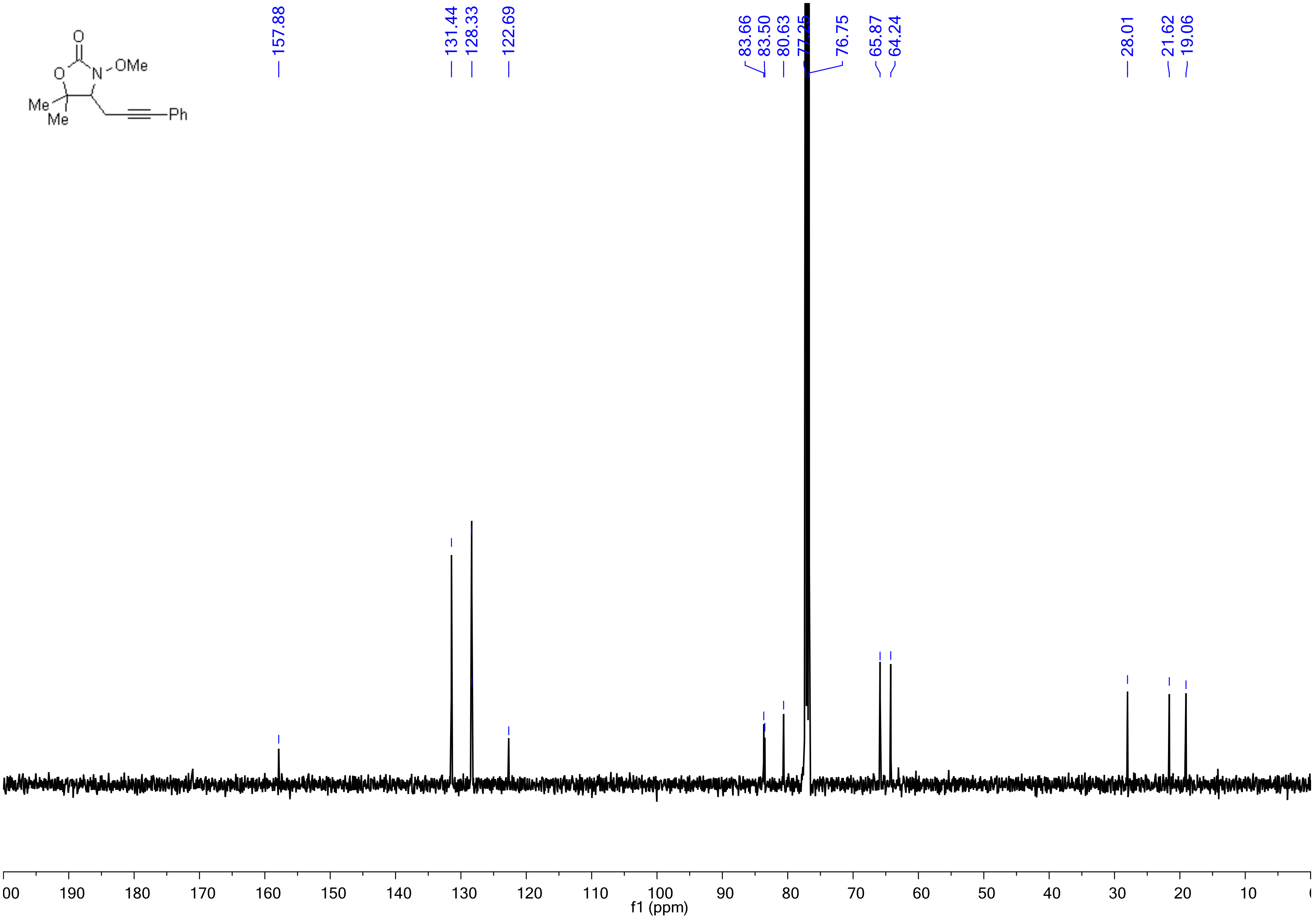
O
O
N
OMe
Me
Me
Ph
157.88
131.44
128.33
122.69
83.66
83.50
80.63
77.25
76.75
65.87
64.24
28.01
21.62
19.06
00
190
180
170
160
150
140
130
120
110
100
90
80
70
60
50
40
30
20
10
f1 (ppm)

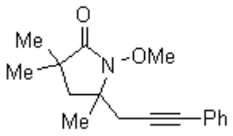

O
Me
Me
N
OMe
Me
Ph

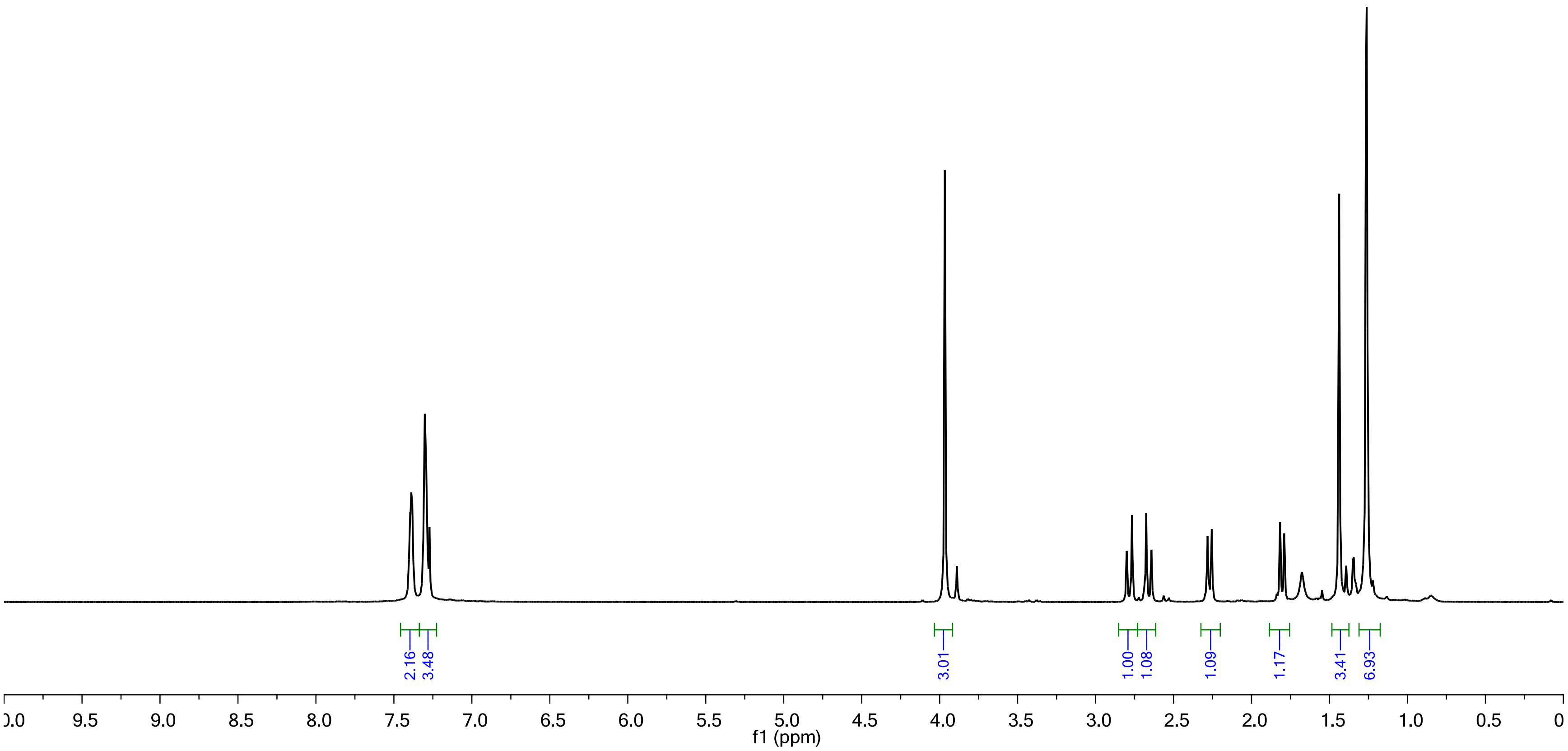

2.16
3.48
3.01
1.00
1.08
1.09
1.17
3.41
6.93
f1 (ppm)

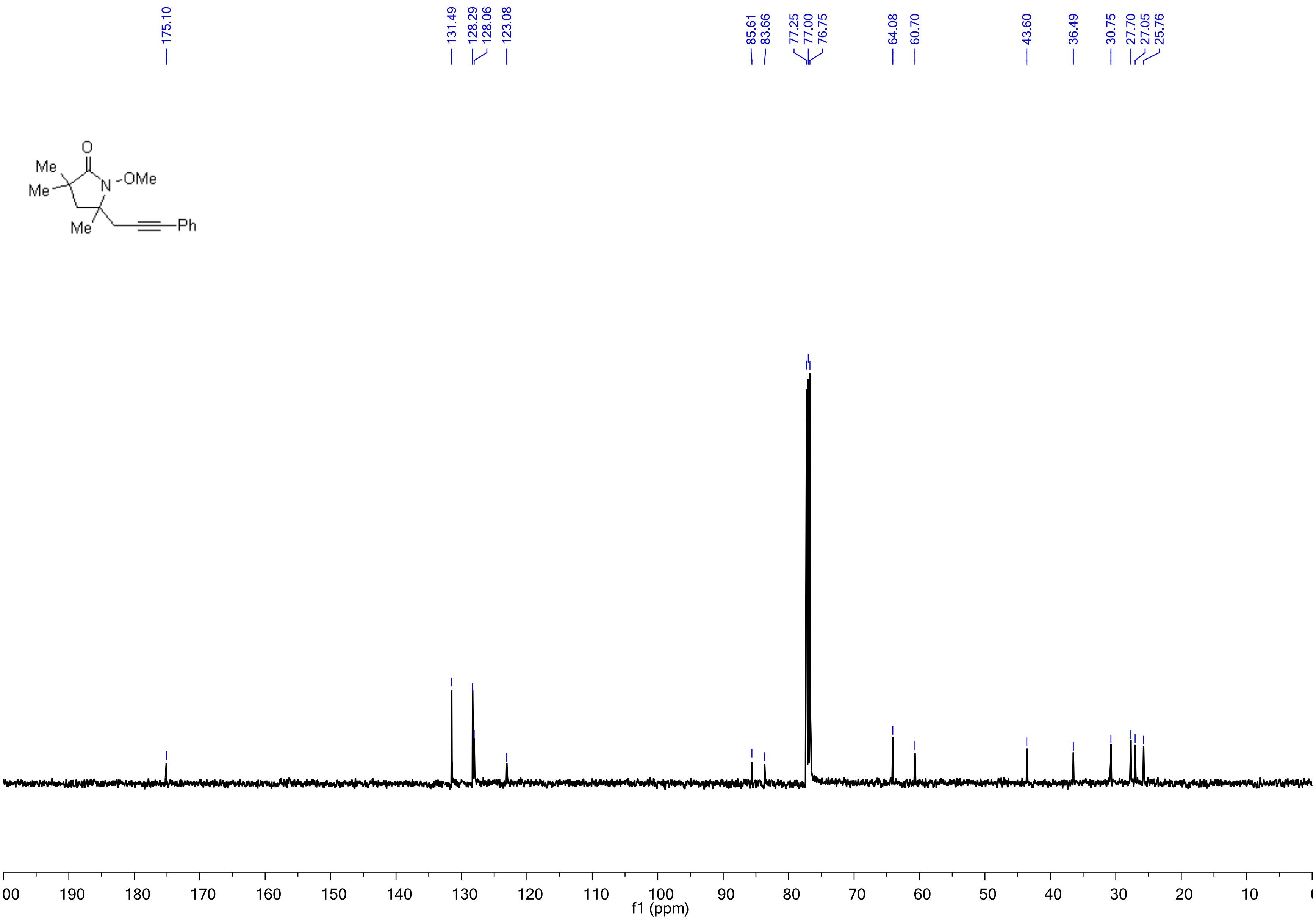
175.10
131.49
128.29
128.06
123.08
85.61
83.66
77.25
77.00
76.75
64.08
60.70
43.60
36.49
30.75
27.70
27.05
25.76
O
Me
Me
N
OMe
Me
Ph
00
190
180
170
160
150
140
130
120
110
100
90
80
70
60
50
40
30
20
10
f1 (ppm)

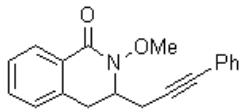
O
N
OMe
Ph

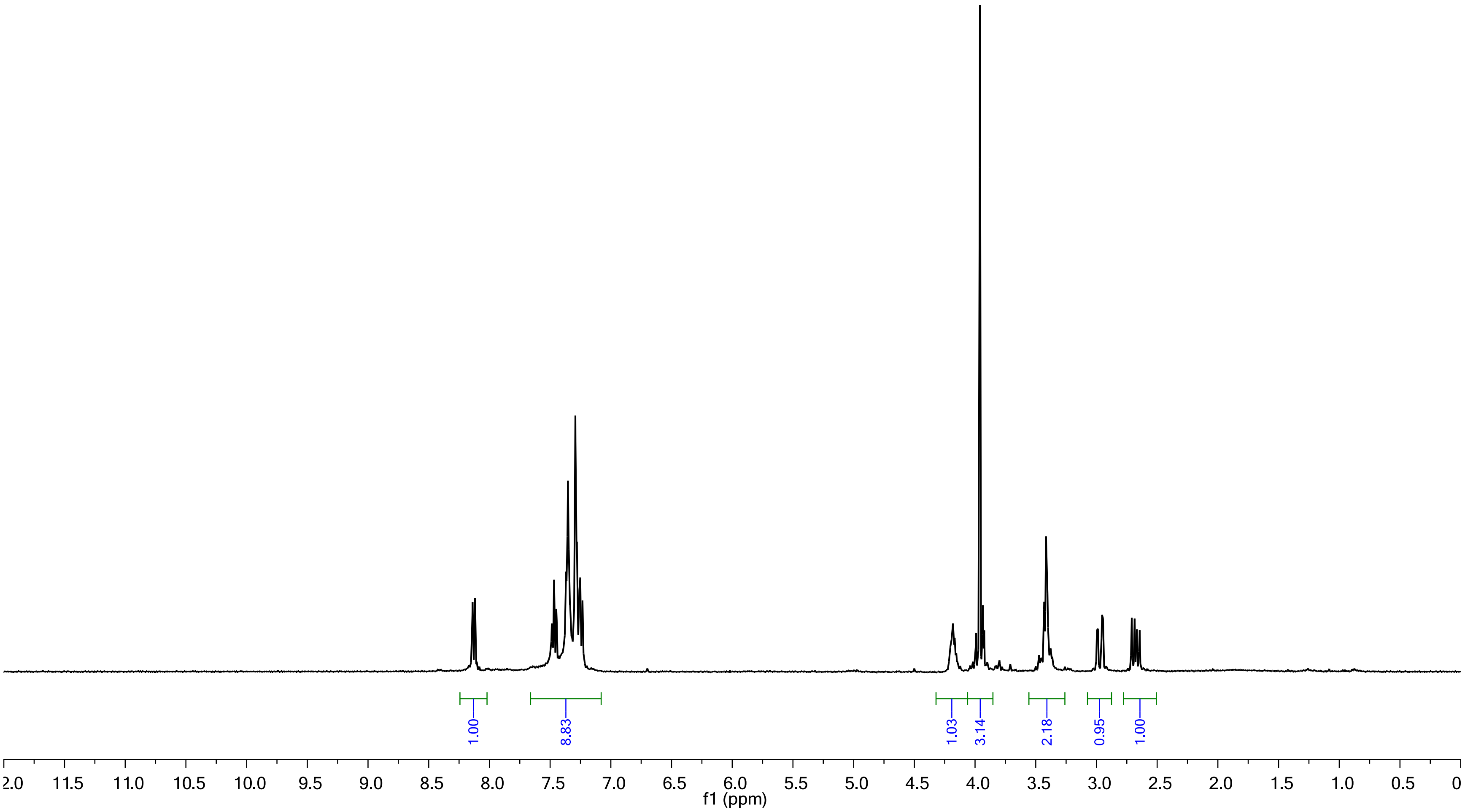
1.00
8.83
1.03
3.14
2.18
0.95
1.00
2.0
11.5
11.0
10.5
10.0
9.5
9.0
8.5
8.0
7.5
7.0
6.5
6.0
5.5
5.0
4.5
4.0
3.5
3.0
2.5
2.0
1.5
1.0
0.5
0
f1 (ppm)

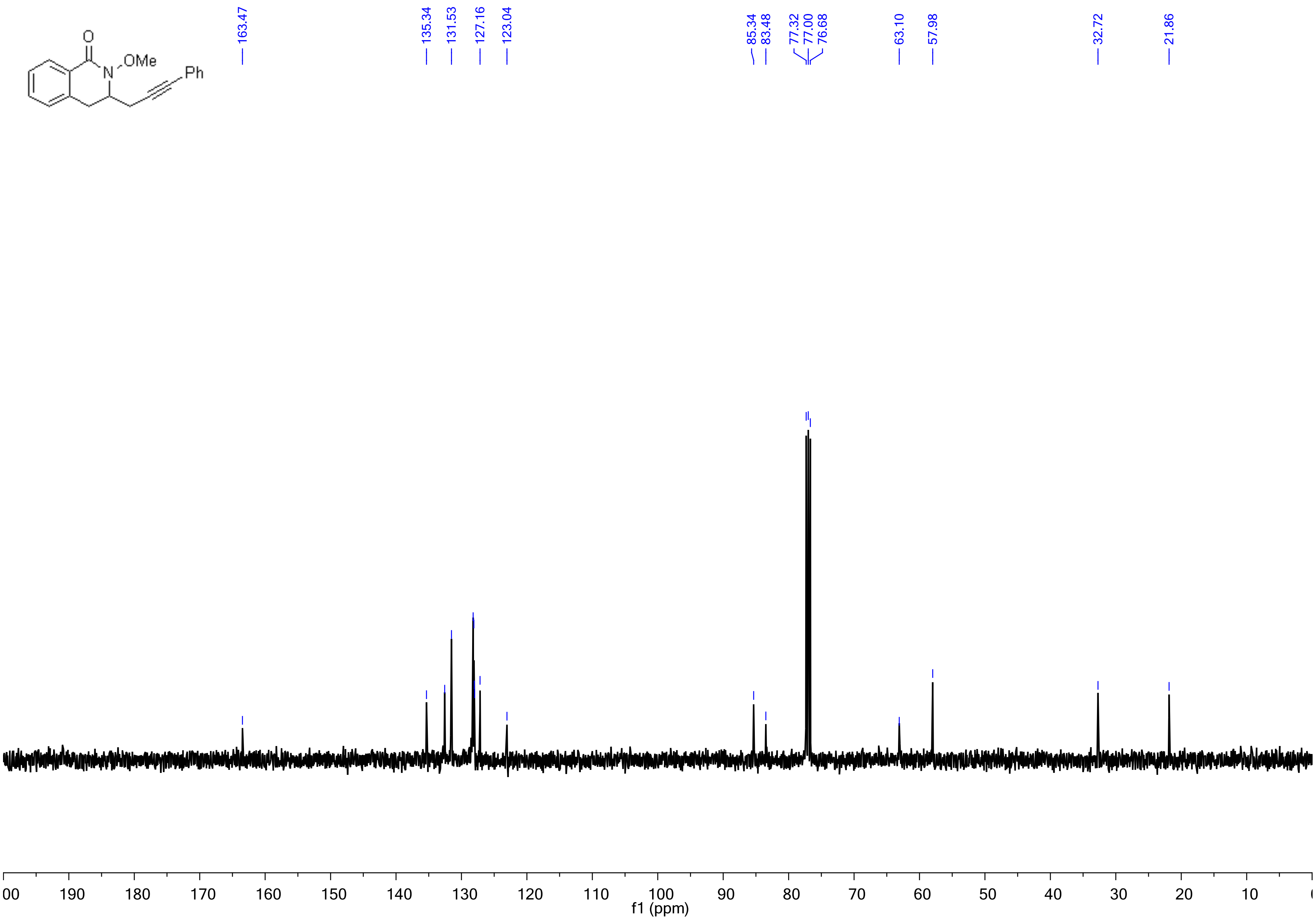

O
N
OMe
Ph
163.47
135.34
131.53
127.16
123.04
85.34
83.48
77.32
77.00
76.68
63.10
57.98
32.72
21.86
f1 (ppm)

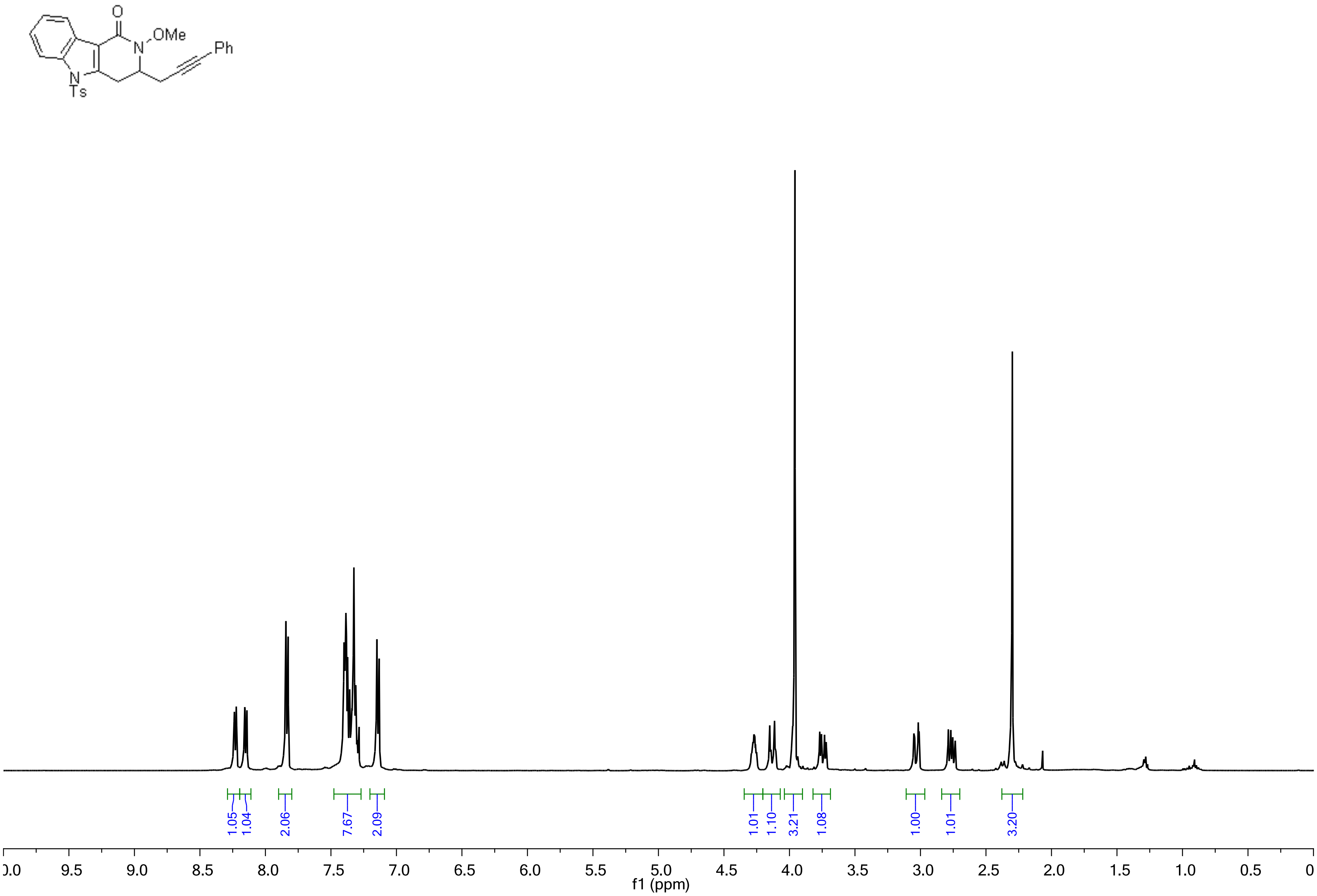
O
N
OMe
Ph
N
Ts
1.05
1.04
2.06
7.67
2.09
1.01
1.10
3.21
1.08
1.00
1.01
3.20
0.0 9.5 9.0 8.5 8.0 7.5 7.0 6.5 6.0 5.5 5.0 4.5 4.0 3.5 3.0 2.5 2.0 1.5 1.0 0.5 0
f1 (ppm)

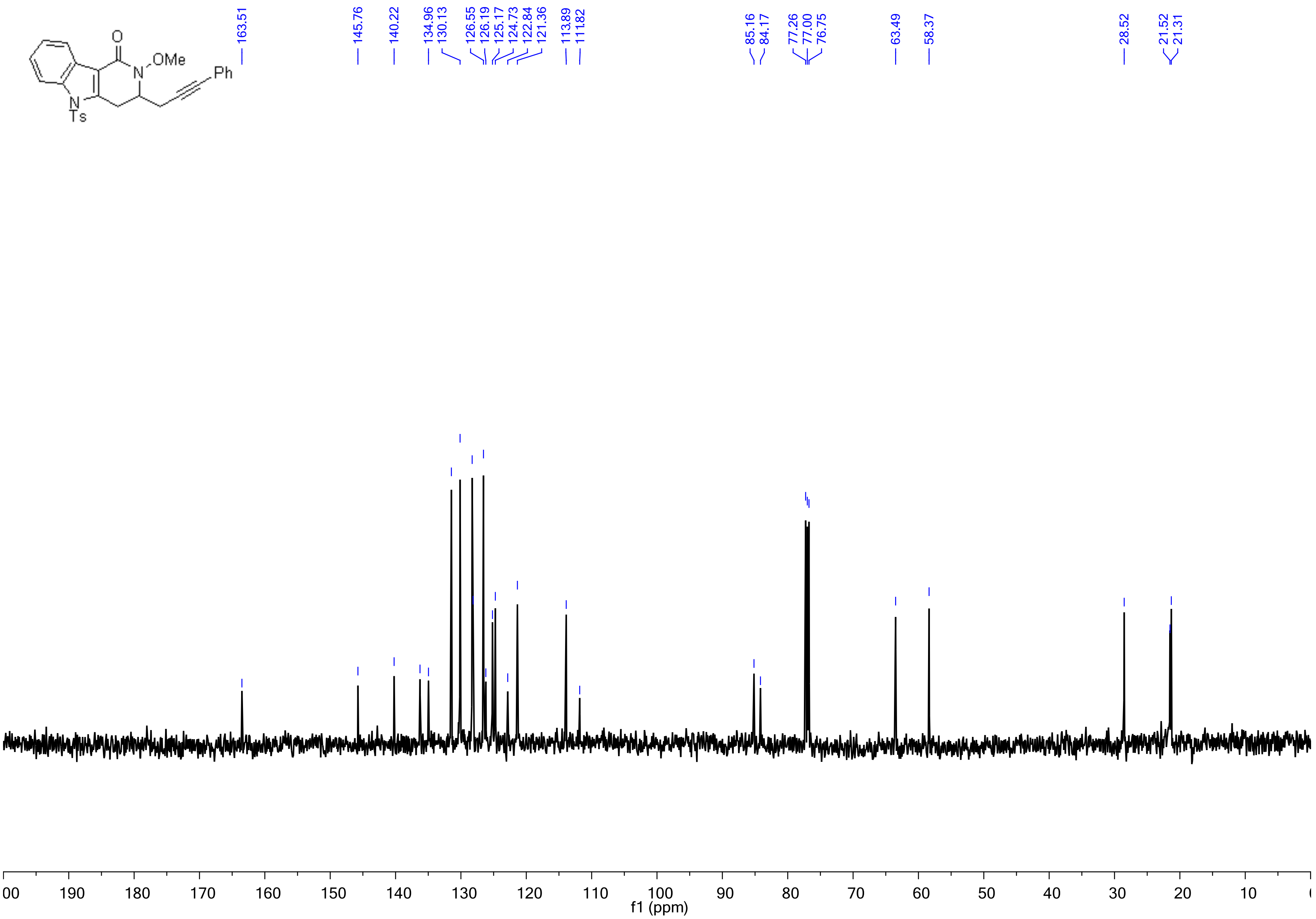
O
N
OMe
Ph
N
Ts
163.51
145.76
140.22
134.96
130.13
126.55
126.19
125.17
124.73
122.84
121.36
113.89
111.82
85.16
84.17
77.26
77.00
76.75
63.49
58.37
28.52
21.52
21.31
00
190
180
170
160
150
140
130
120
110
100
90
80
70
60
50
40
30
20
10
f1 (ppm)

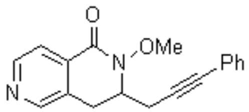
O
N
OMe
Ph
N

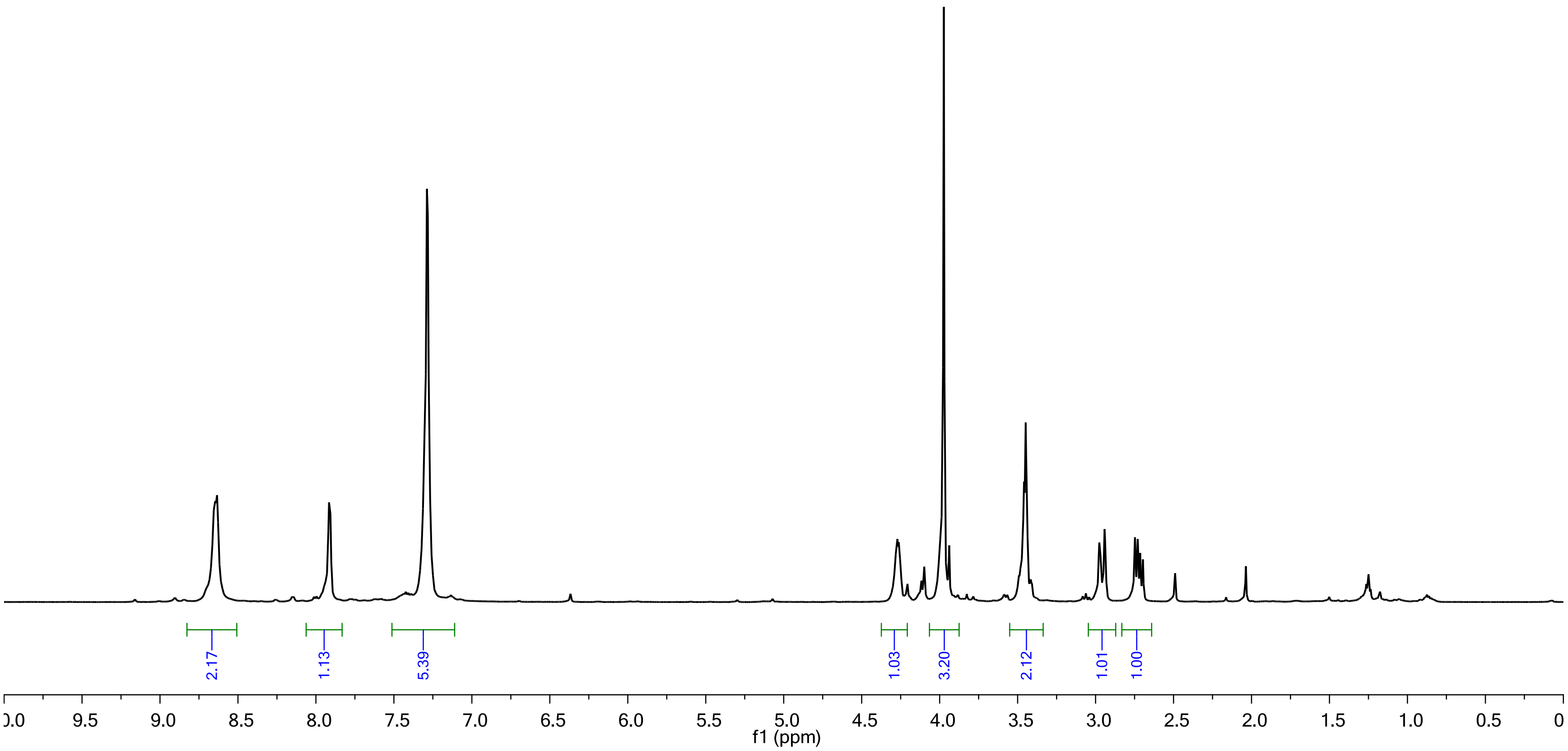
2.17
1.13
5.39
1.03
3.20
2.12
1.01
1.00
9.5
9.0
8.5
8.0
7.5
7.0
6.5
6.0
5.5
5.0
4.5
4.0
3.5
3.0
2.5
2.0
1.5
1.0
0.5
f1 (ppm)

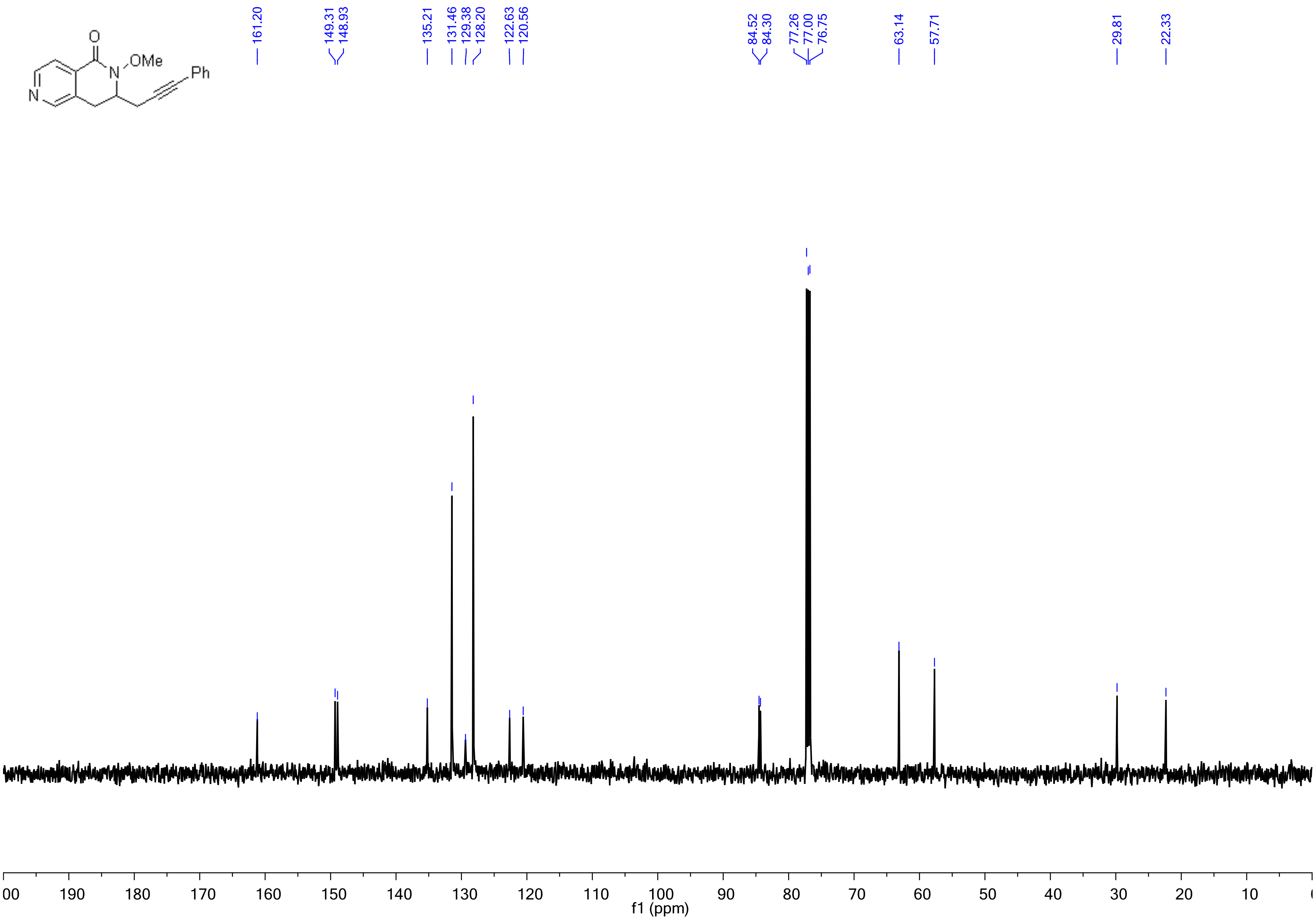

O
N
OMe
N
Ph
161.20
149.31
148.93
135.21
131.46
129.38
128.20
122.63
120.56
84.52
84.30
77.26
77.00
76.75
63.14
57.71
29.81
22.33
00
190
180
170
160
150
140
130
120
110
100
90
80
70
60
50
40
30
20
10
f1 (ppm)

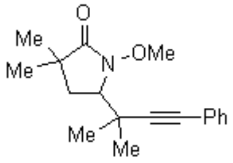
O
Me
Me
N
OMe
Me
Me
Ph

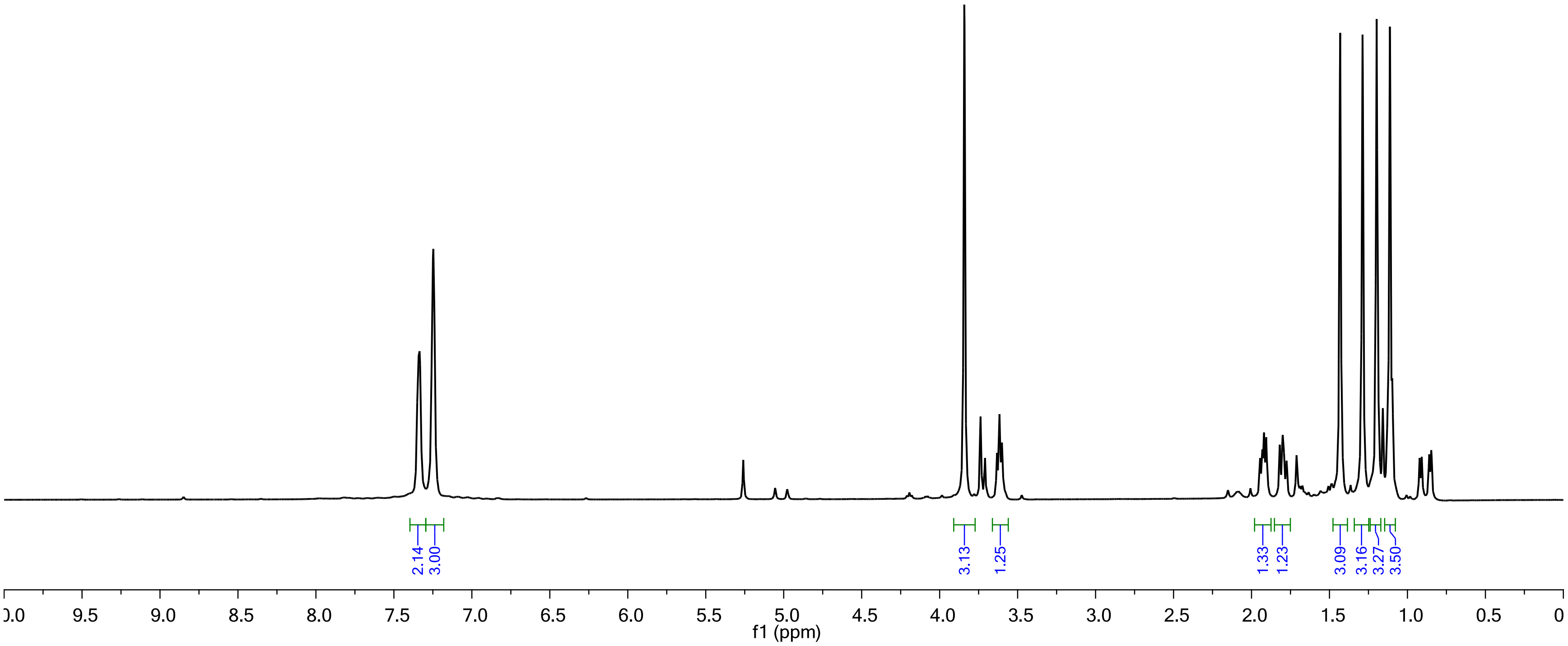
2.14
3.00
3.13
1.25
1.33
1.23
3.09
3.16
3.27
3.50
9.5
9.0
8.5
8.0
7.5
7.0
6.5
6.0
5.5
5.0
4.5
4.0
3.5
3.0
2.5
2.0
1.5
1.0
0.5
f1 (ppm)

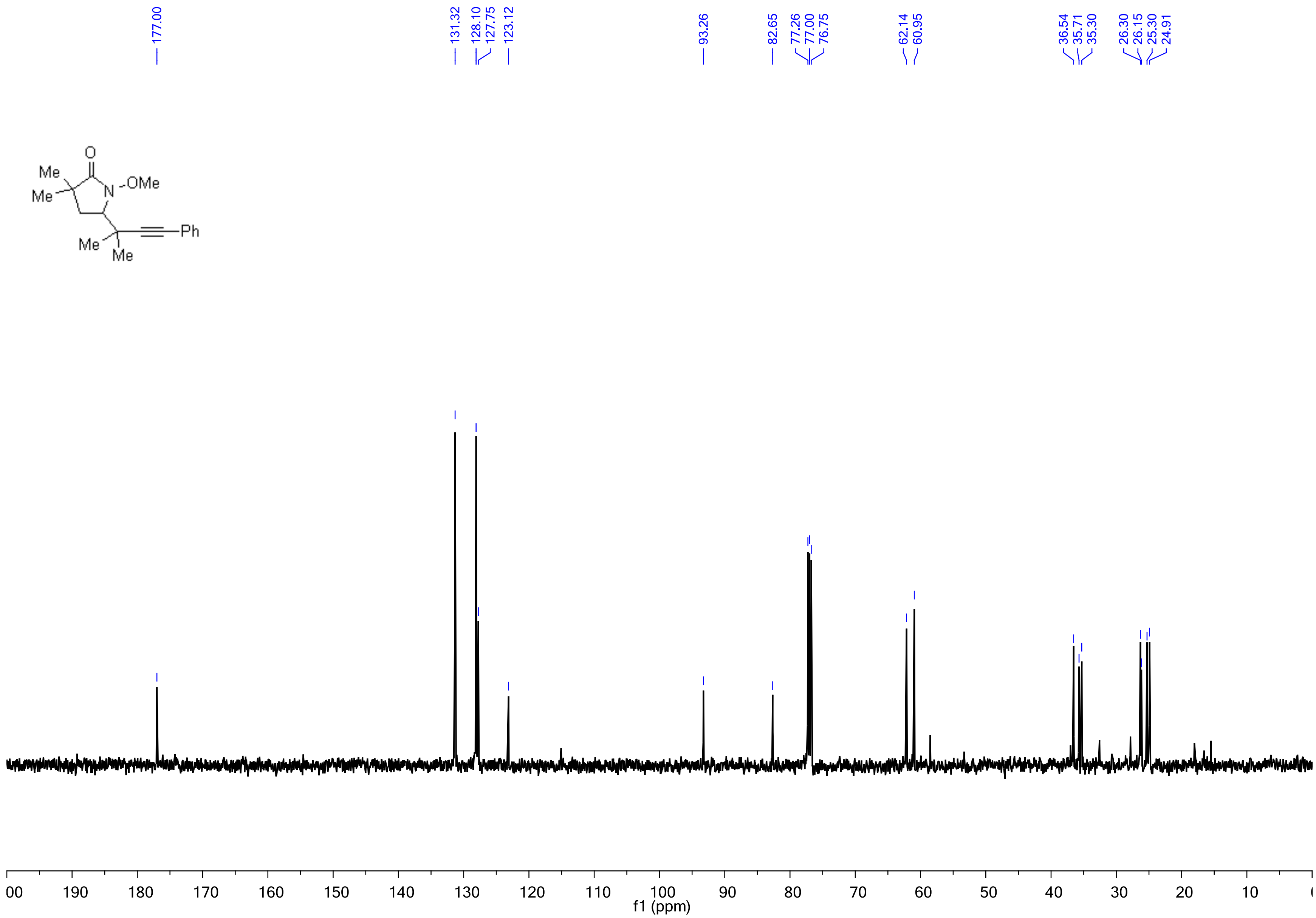

Me
Me
O
N
OMe
Ph
Me
Me
177.00
131.32
128.10
127.75
123.12
93.26
82.65
77.26
77.00
76.75
62.14
60.95
36.54
35.71
35.30
26.30
26.15
25.30
24.91
00
190
180
170
160
150
140
130
120
110
100
90
80
70
60
50
40
30
20
10
f1 (ppm)

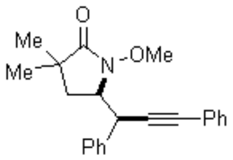

O
Me
Me
N
OMe
Ph
Ph

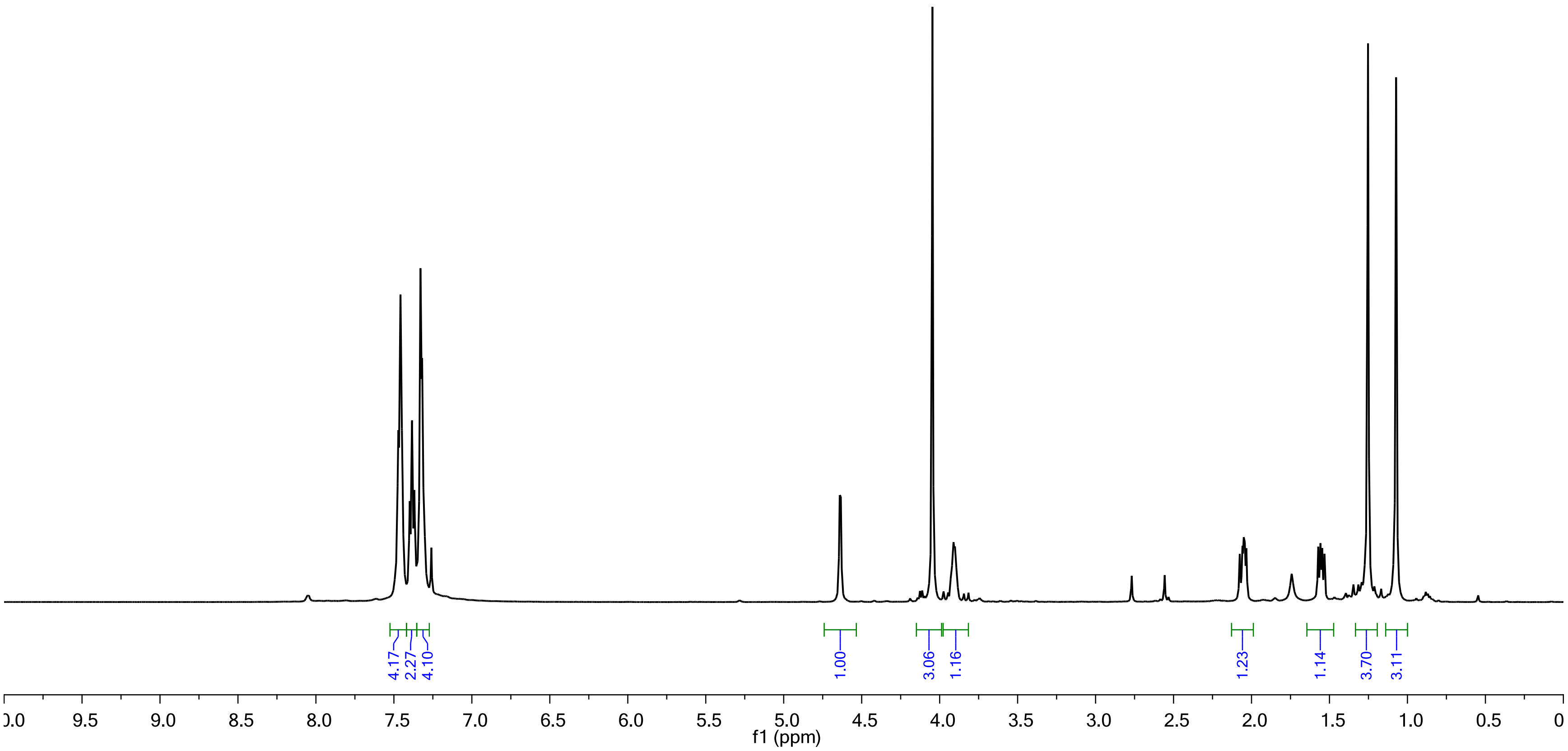

4.17
2.27
4.10
1.00
3.06
1.16
1.23
1.14
3.70
3.11
f1 (ppm)

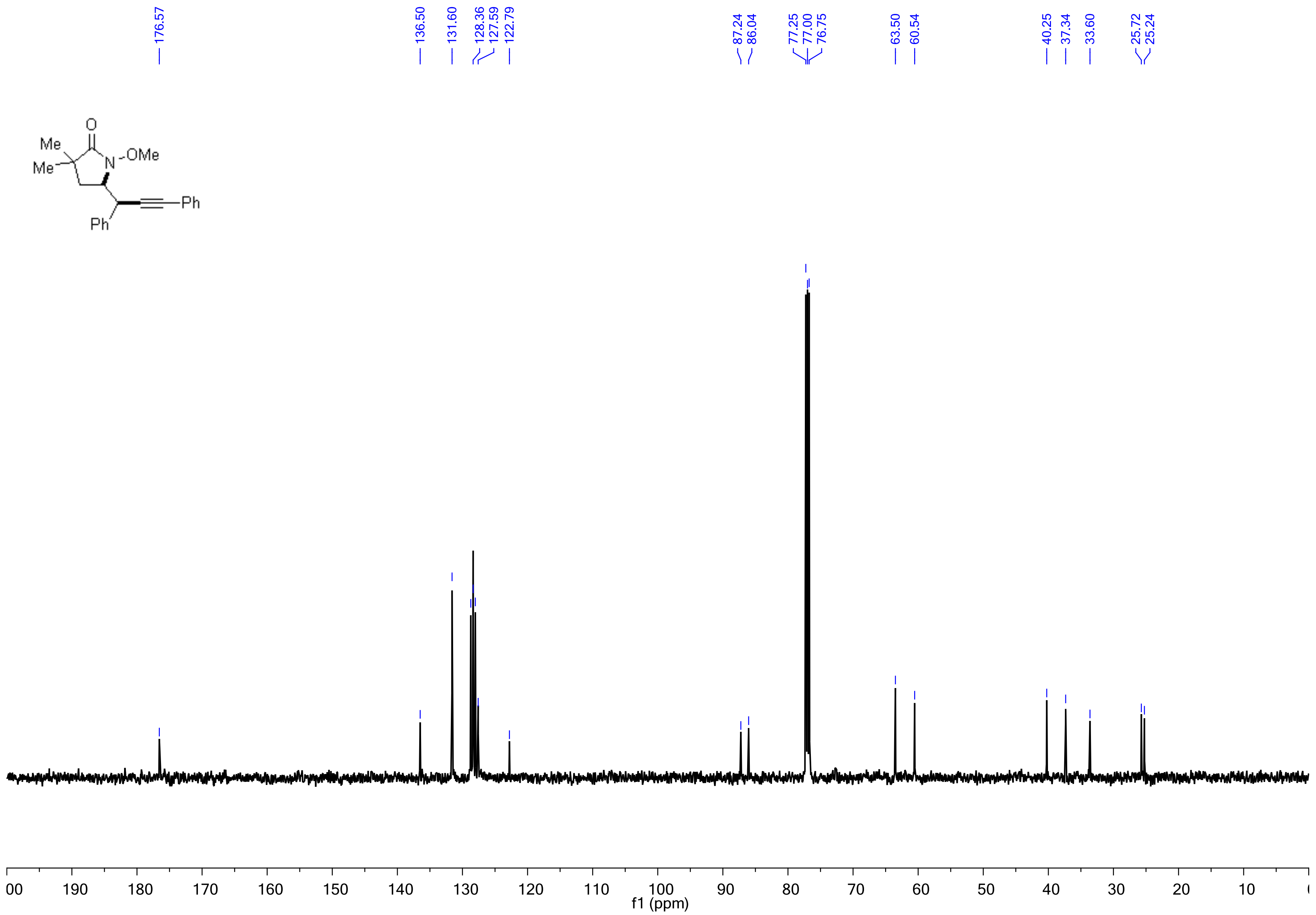

176.57
136.50
131.60
128.36
127.59
122.79
87.24
86.04
77.25
77.00
76.75
63.50
60.54
40.25
37.34
33.60
25.72
25.24
O
Me
Me
N
OMe
Ph
Ph
f1 (ppm)

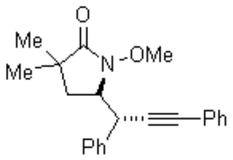

O
Me
Me
N
OMe
Ph
Ph

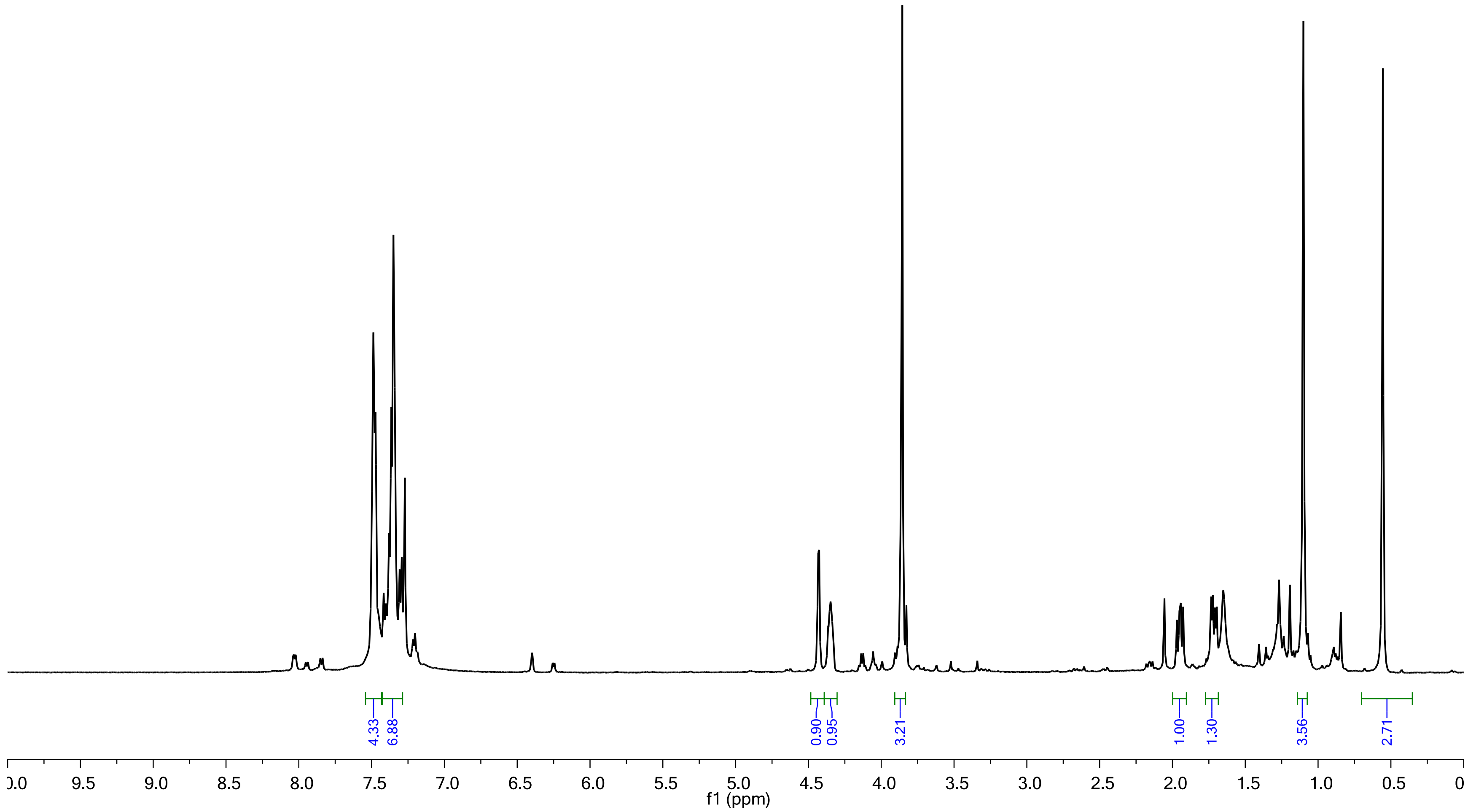

4.33
6.88
0.90
0.95
3.21
1.00
1.30
3.56
2.71
f1 (ppm)

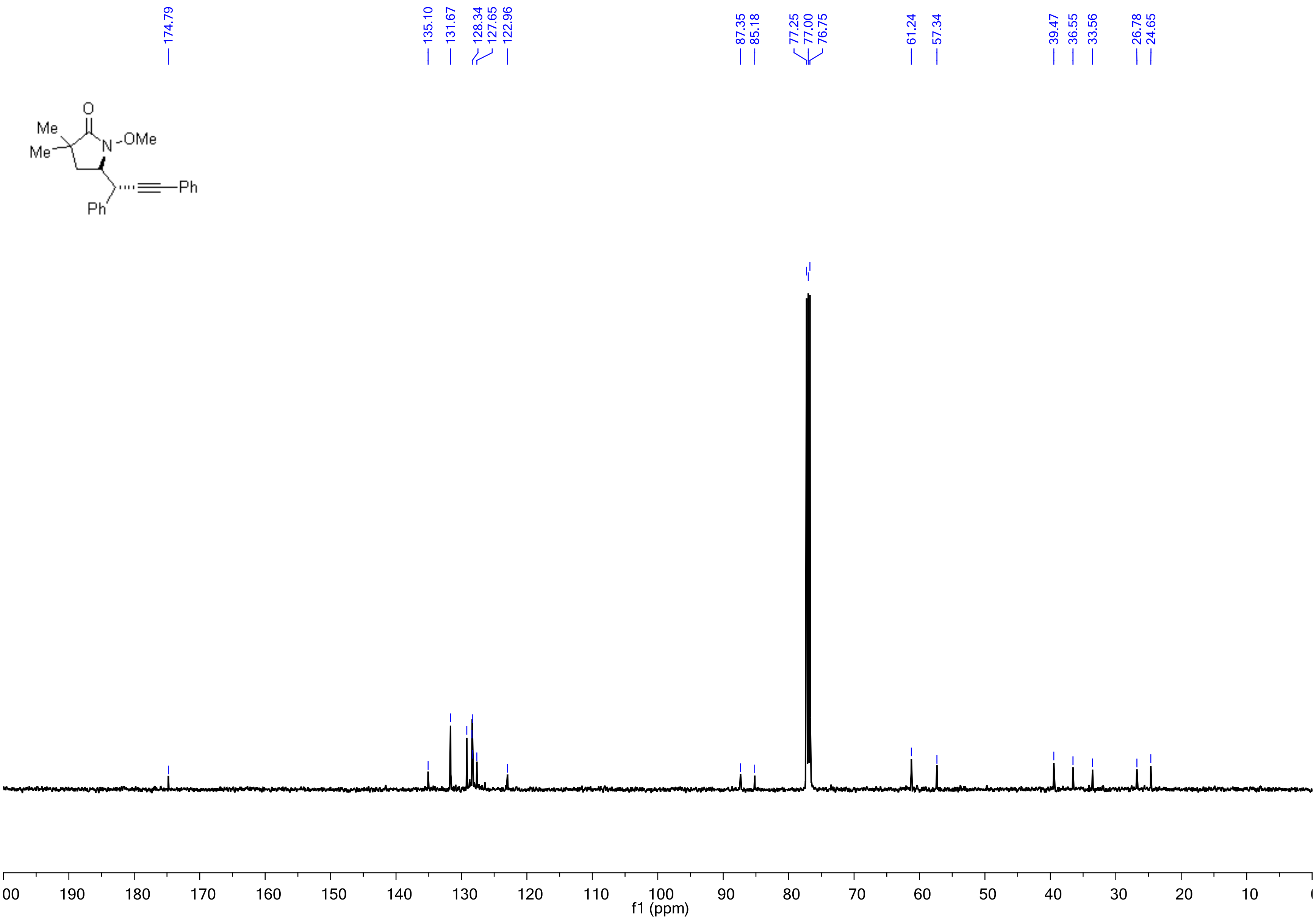
174.79
135.10
131.67
128.34
127.65
122.96
87.35
85.18
77.25
77.00
76.75
61.24
57.34
39.47
36.55
33.56
26.78
24.65
O
Me
Me
N
OMe
Ph
Ph
f1 (ppm)

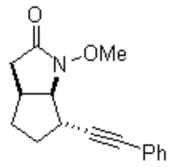

O
N
OMe
Ph

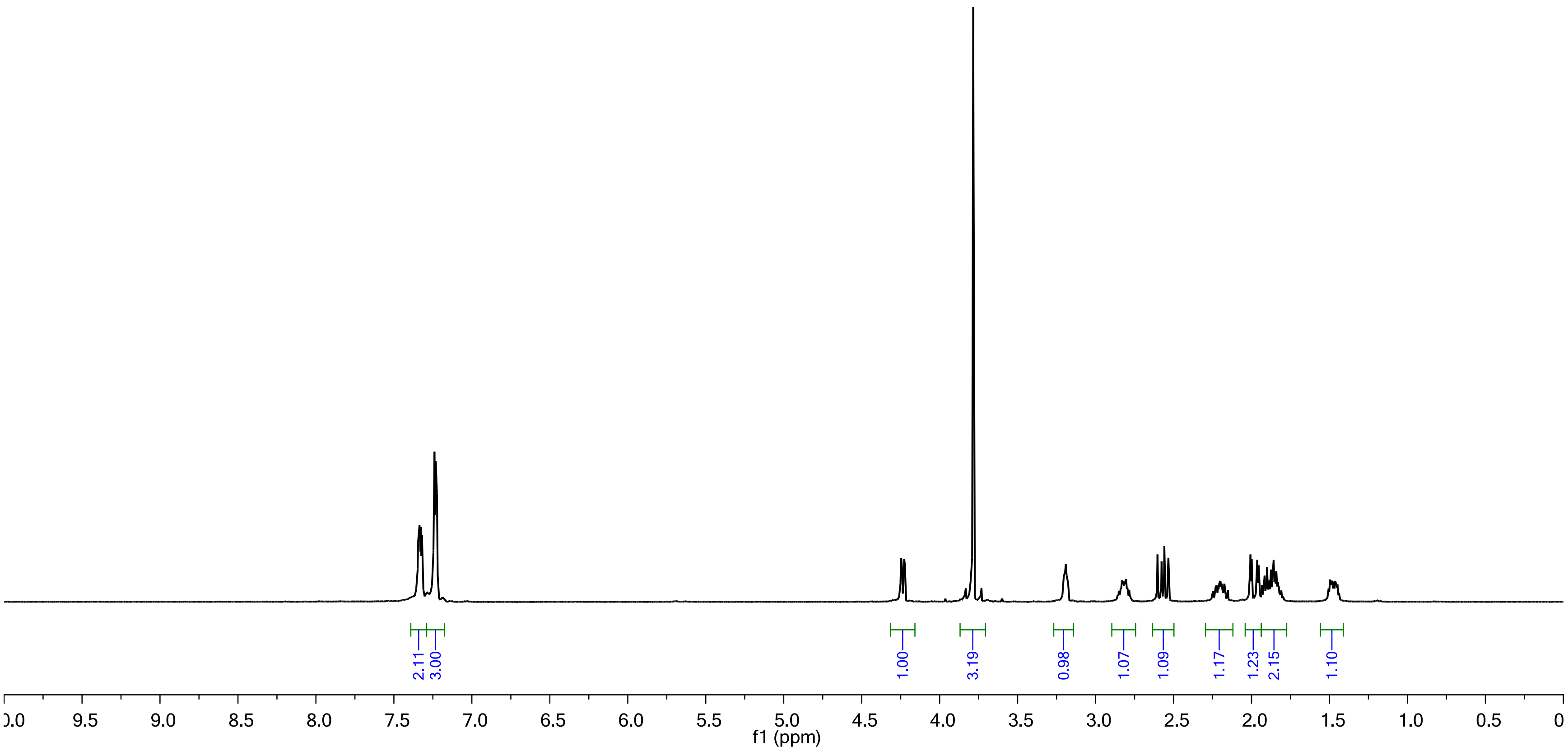

2.11
3.00
1.00
3.19
0.98
1.07
1.09
1.17
1.23
2.15
1.10
9.5
9.0
8.5
8.0
7.5
7.0
6.5
6.0
5.5
5.0
4.5
4.0
3.5
3.0
2.5
2.0
1.5
1.0
0.5
0
f1 (ppm)

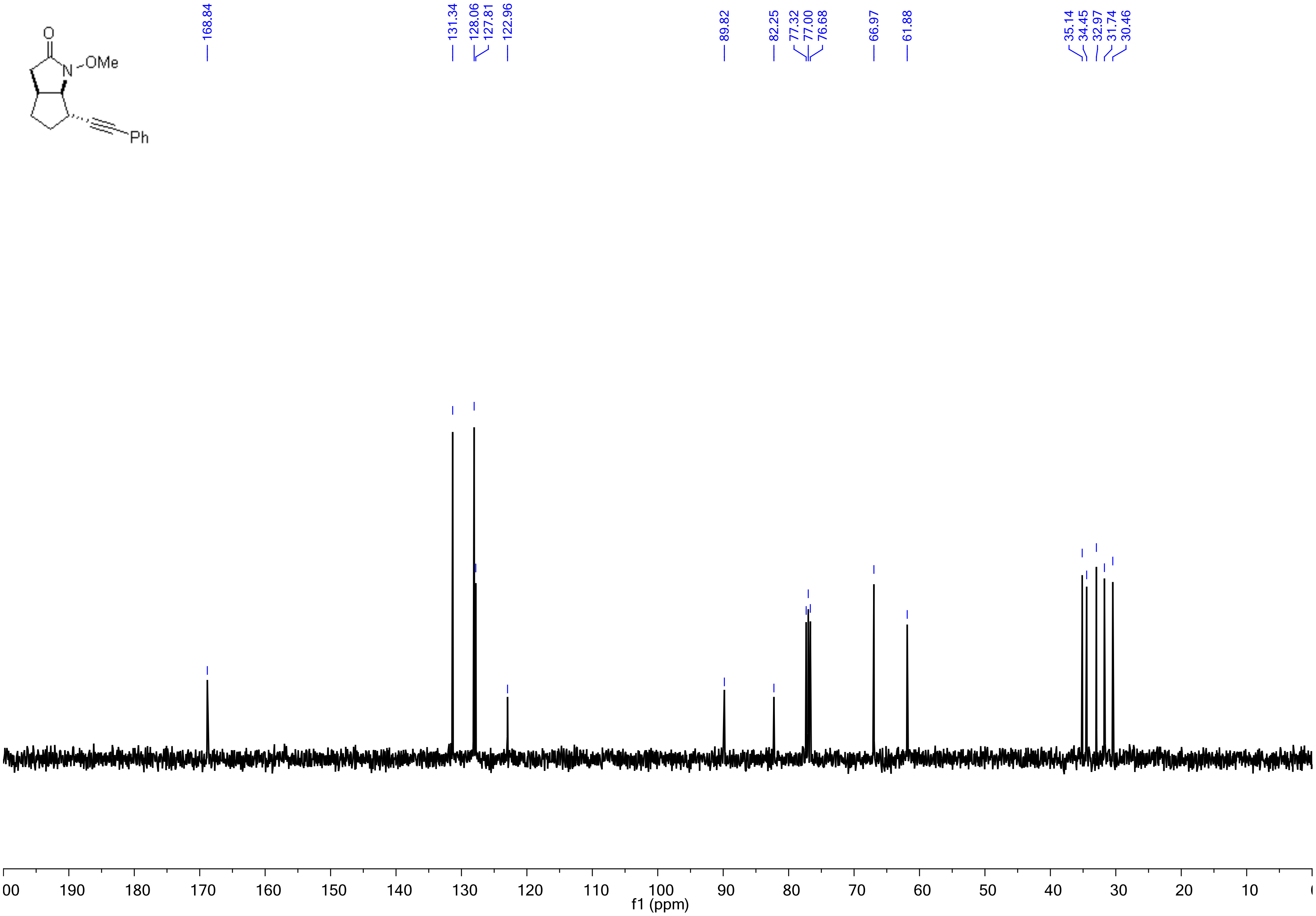
O
N
OMe
Ph
168.84
131.34
128.06
127.81
122.96
89.82
82.25
77.32
77.00
76.68
66.97
61.88
35.14
34.45
32.97
31.74
30.46
00
190
180
170
160
150
140
130
120
110
100
90
80
70
60
50
40
30
20
10
f1 (ppm)

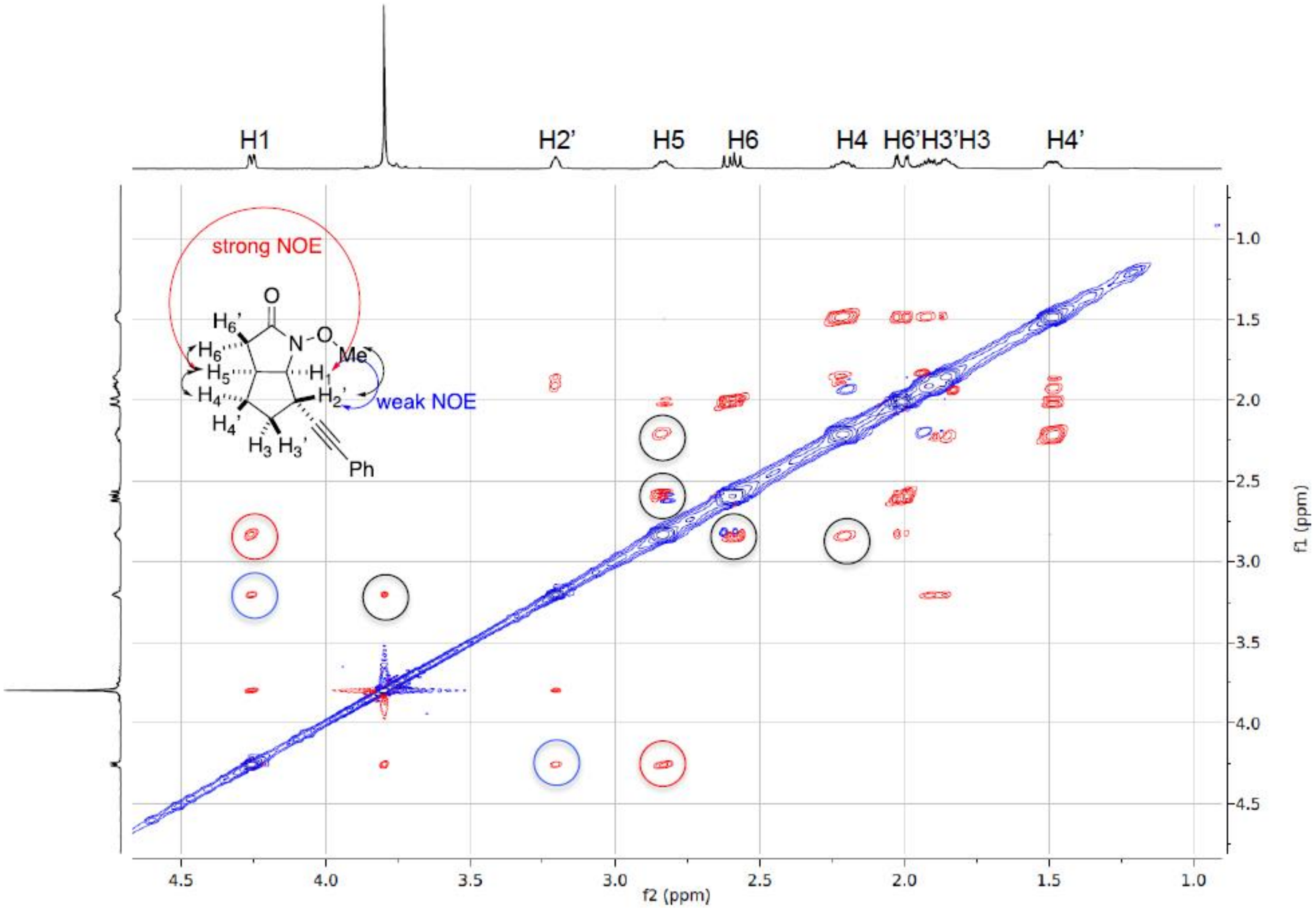
H1
H2'
H5
H6
H4
H6'H3'H3
H4'
strong NOE
weak NOE
O
N
O
Me
H6'
H6
H5
H4
H1
H2'
H4'
H3 H3'
Ph
f2 (ppm)
f1 (ppm)
4.5
4.0
3.5
3.0
2.5
2.0
1.5
1.0

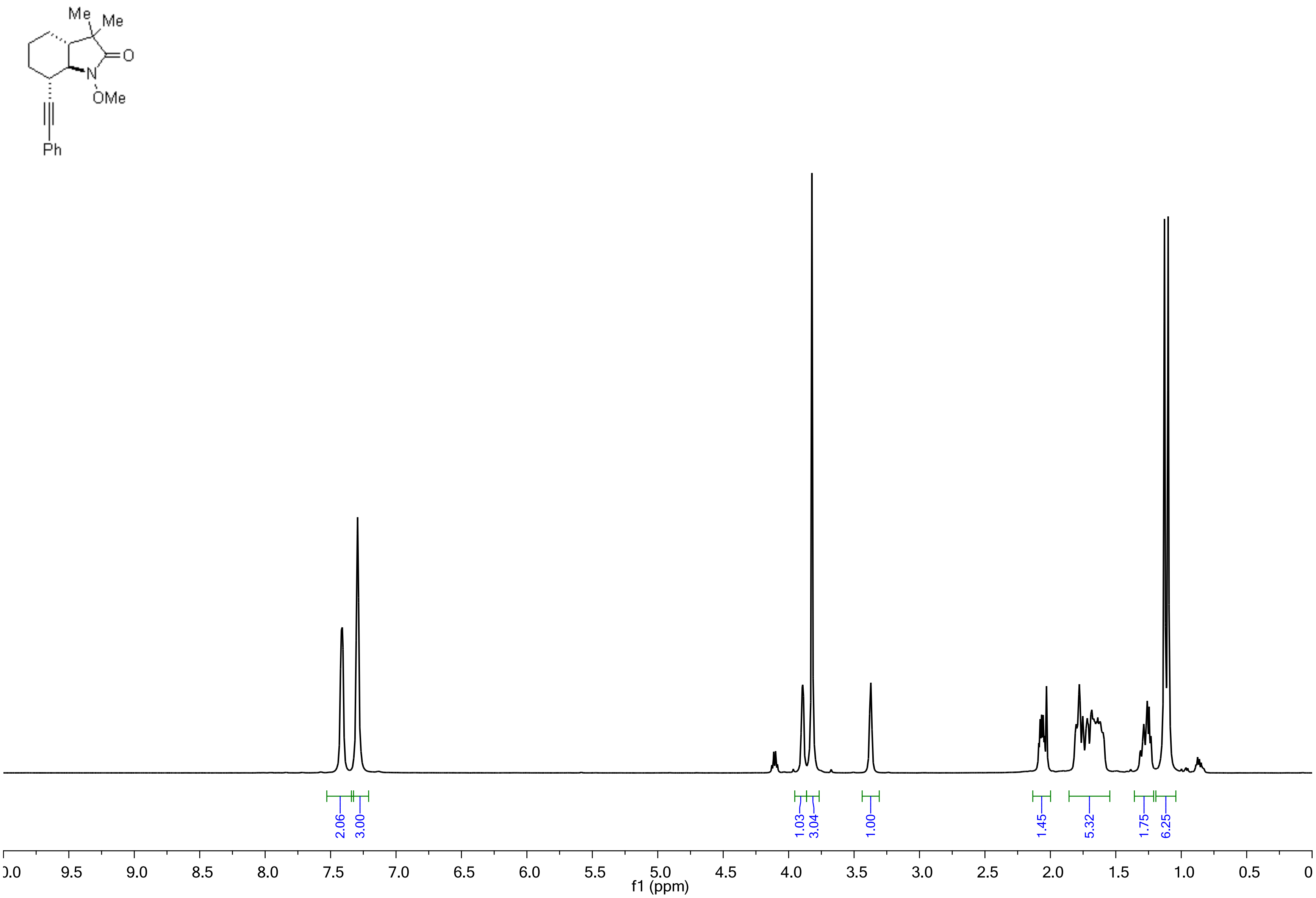
Me
Me
O
N
OMe
Ph
2.06
3.00
1.03
3.04
1.00
1.45
5.32
1.75
6.25
9.5
9.0
8.5
8.0
7.5
7.0
6.5
6.0
5.5
5.0
4.5
4.0
3.5
3.0
2.5
2.0
1.5
1.0
0.5
f1 (ppm)

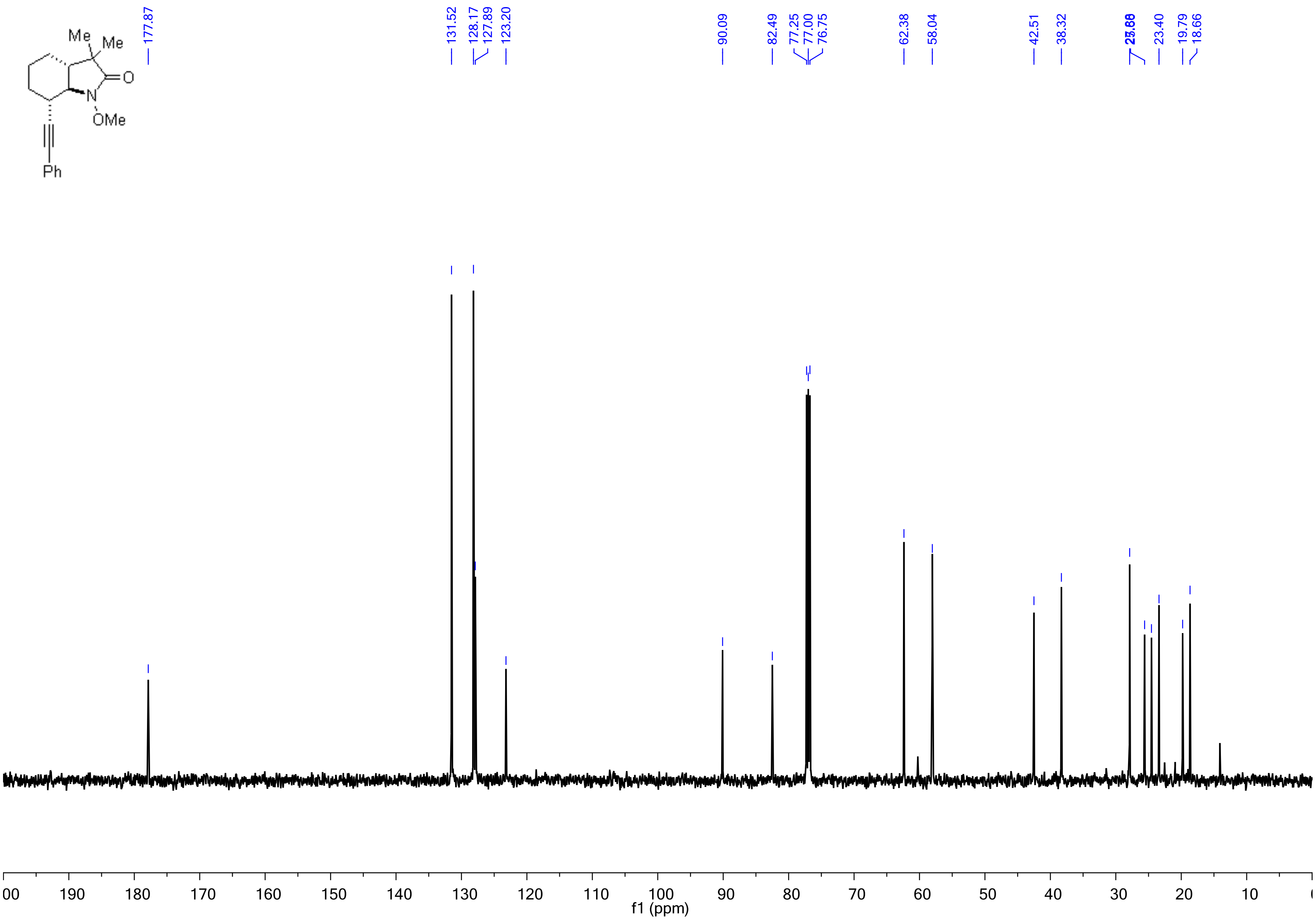
Me
Me
O
N
OMe
Ph
177.87
131.52
128.17
127.89
123.20
90.09
82.49
77.25
77.00
76.75
62.38
58.04
42.51
38.32
23.40
19.79
18.66
f1 (ppm)

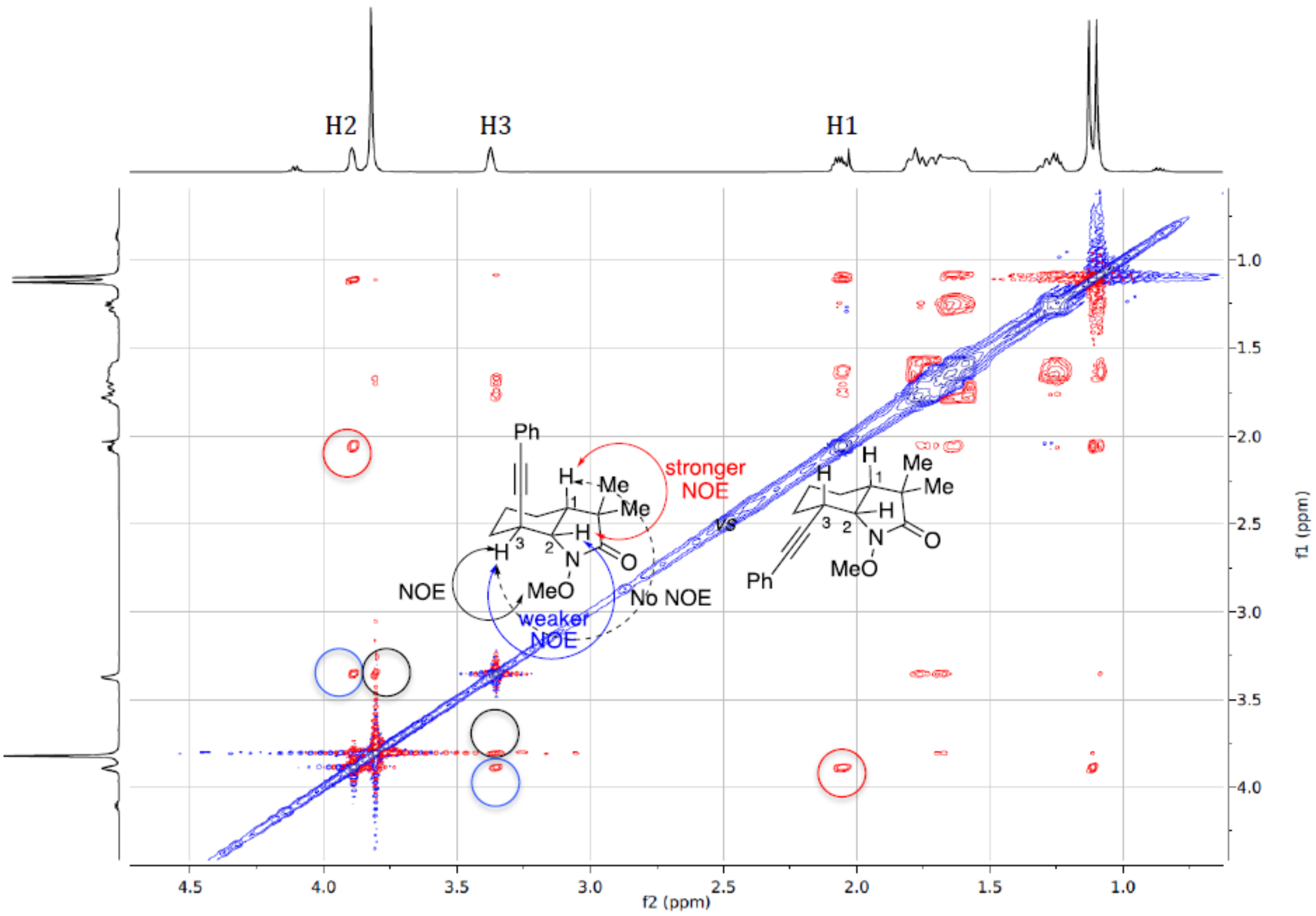
H2
H3
H1
Ph
H
Me
Me
1
3
2
H
N
O
H
MeO
stronger
NOE
NOE
weaker
NOE
No NOE
vs
Ph
f2 (ppm)
f1 (ppm)

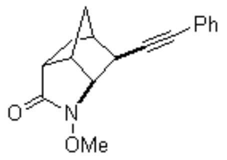
Ph
O
N
OMe

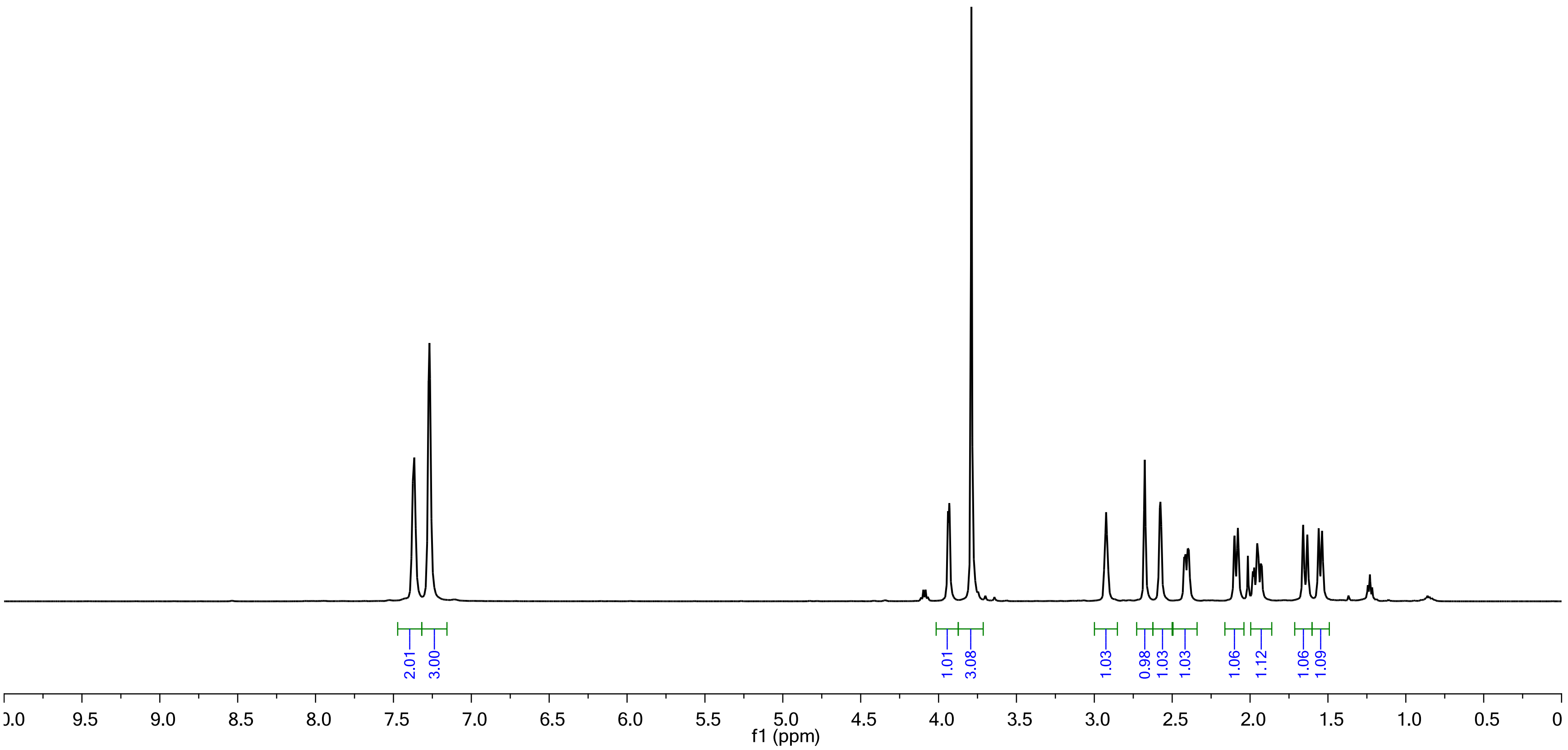
2.01
3.00
1.01
3.08
1.03
0.98
1.03
1.03
1.06
1.12
1.06
1.09
9.5
9.0
8.5
8.0
7.5
7.0
6.5
6.0
5.5
5.0
4.5
4.0
3.5
3.0
2.5
2.0
1.5
1.0
0.5
0
f1 (ppm)

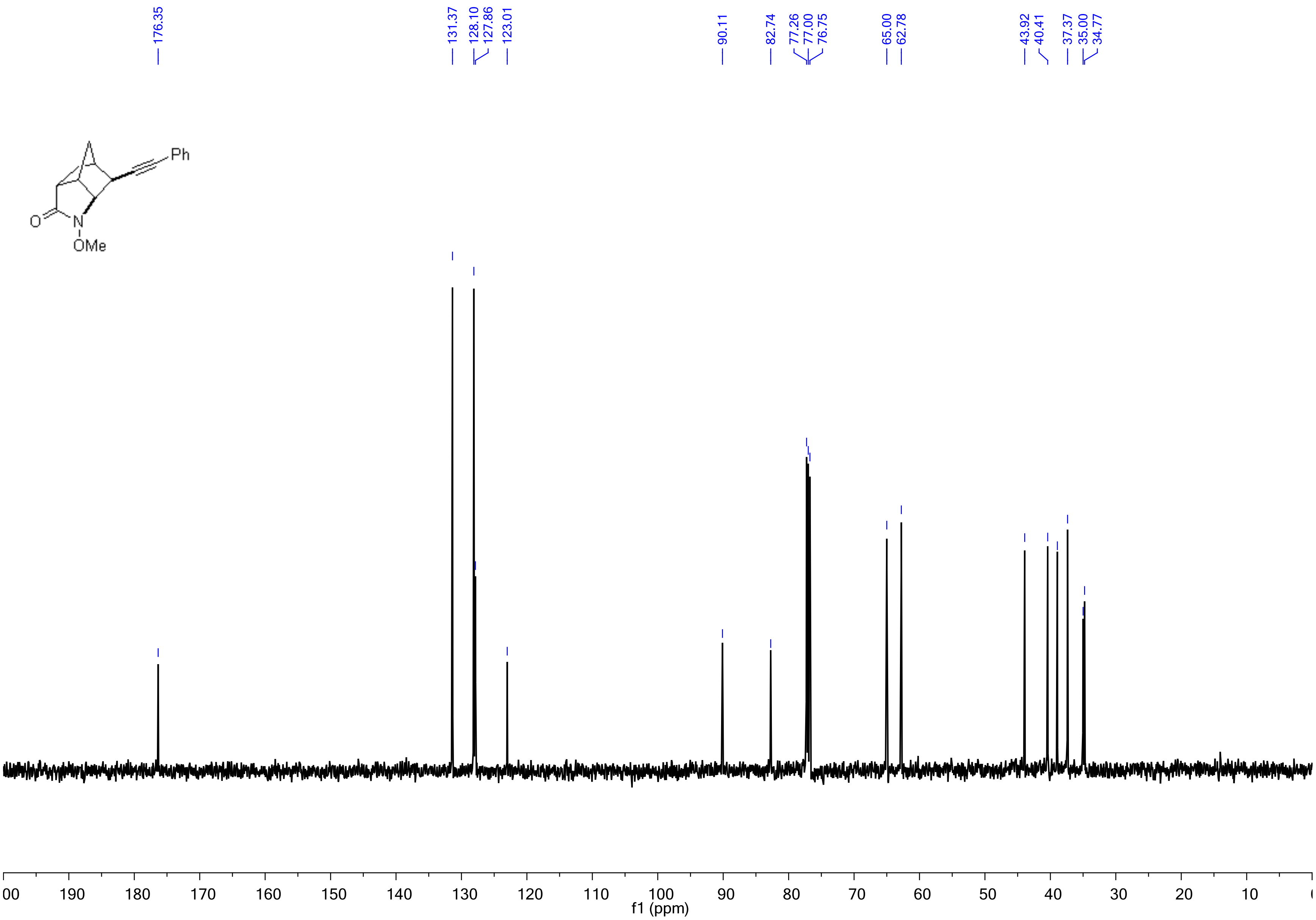
176.35
131.37
128.10
127.86
123.01
90.11
82.74
77.26
77.00
76.75
65.00
62.78
43.92
40.41
37.37
35.00
34.77
Ph
O
N
OMe
f1 (ppm)

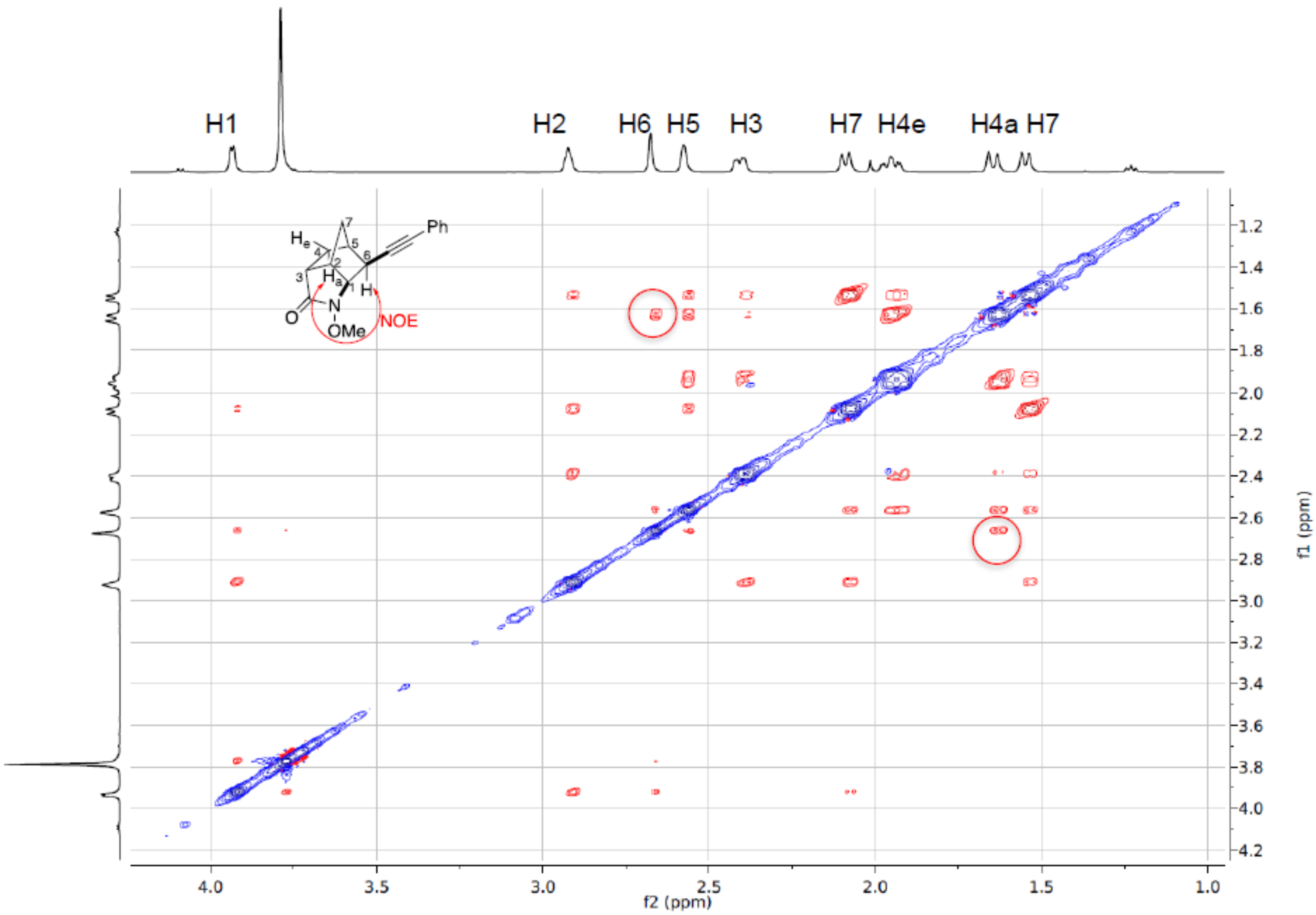
H1
H2
H6
H5
H3
H7
H4e
H4a
H7
Ph
He
Ha
N
O
OMe
H
NOE
f2 (ppm)
f1 (ppm)

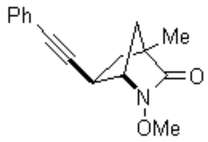
Ph
Me
O
N
OMe

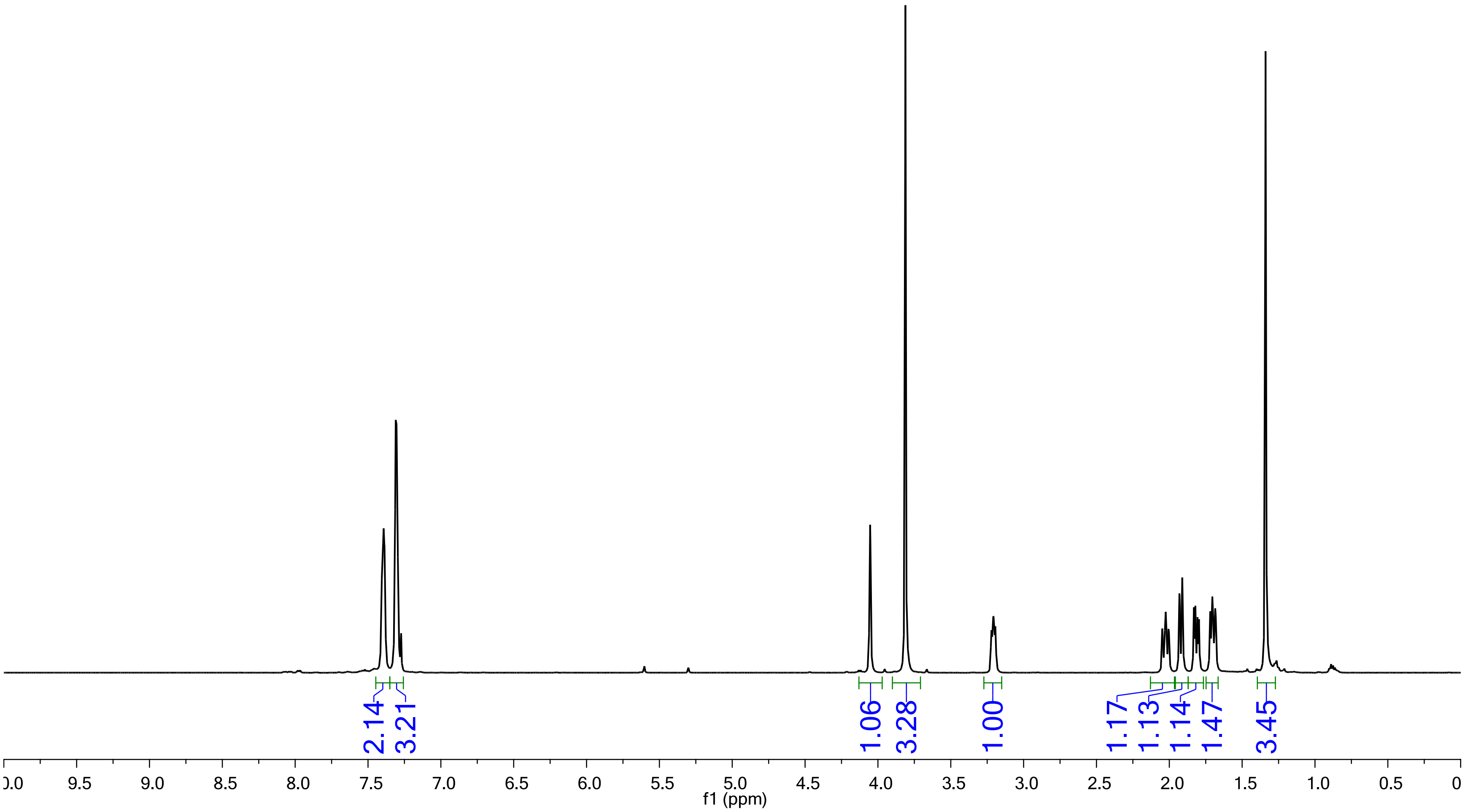
2.14
3.21
1.06
3.28
1.00
1.17
1.13
1.14
1.47
3.45
10.0
9.5
9.0
8.5
8.0
7.5
7.0
6.5
6.0
5.5
5.0
4.5
4.0
3.5
3.0
2.5
2.0
1.5
1.0
0.5
0
f1 (ppm)

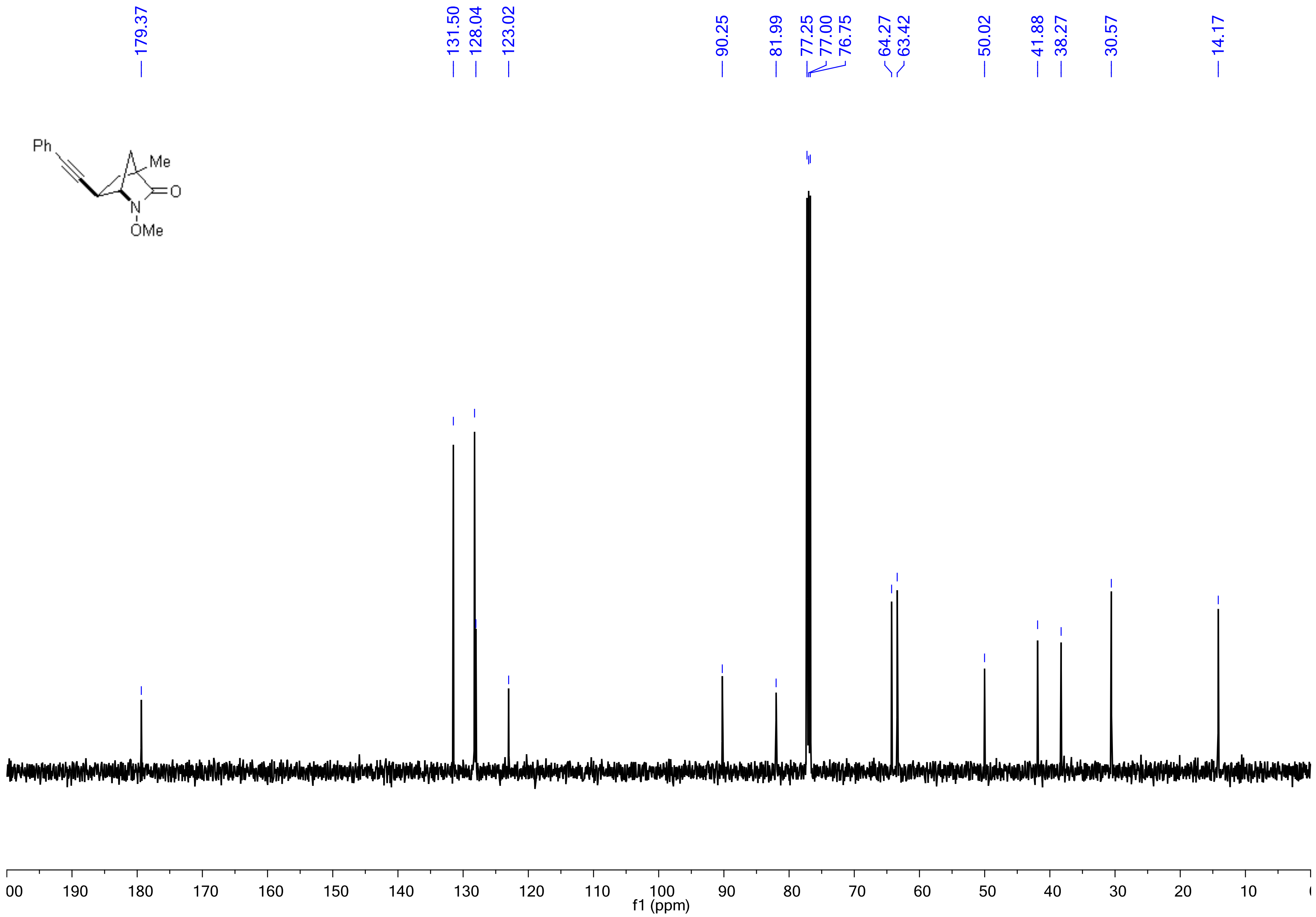
179.37
131.50
128.04
123.02
90.25
81.99
77.25
77.00
76.75
64.27
63.42
50.02
41.88
38.27
30.57
14.17
Ph
Me
O
N
OMe
190
180
170
160
150
140
130
120
110
100
90
80
70
60
50
40
30
20
10
f1 (ppm)

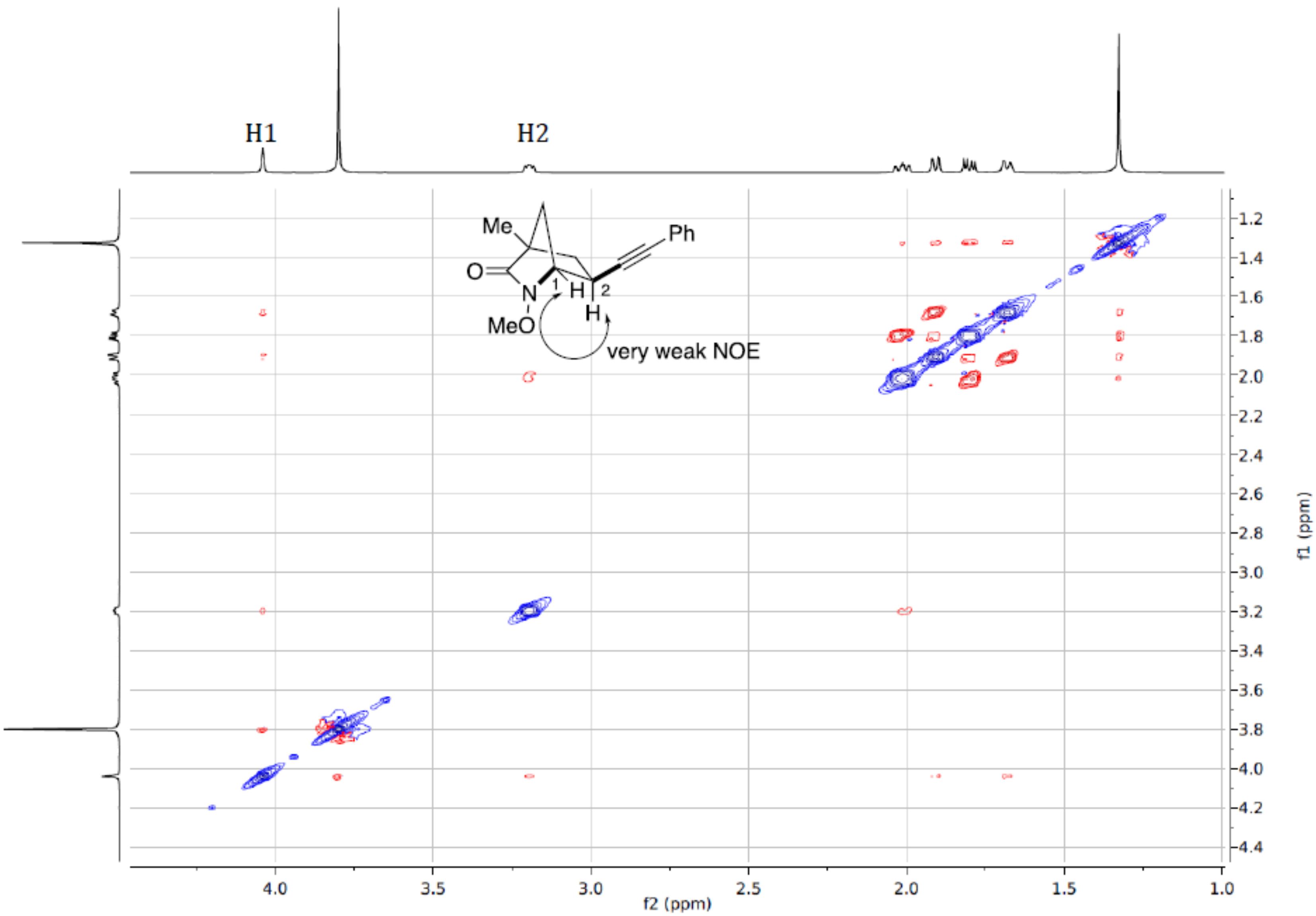
H1
H2
Me
O
N
MeO
1
H
2
H
Ph
very weak NOE
f2 (ppm)
f1 (ppm)

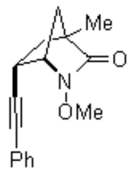
Me
O
N
OMe
Ph

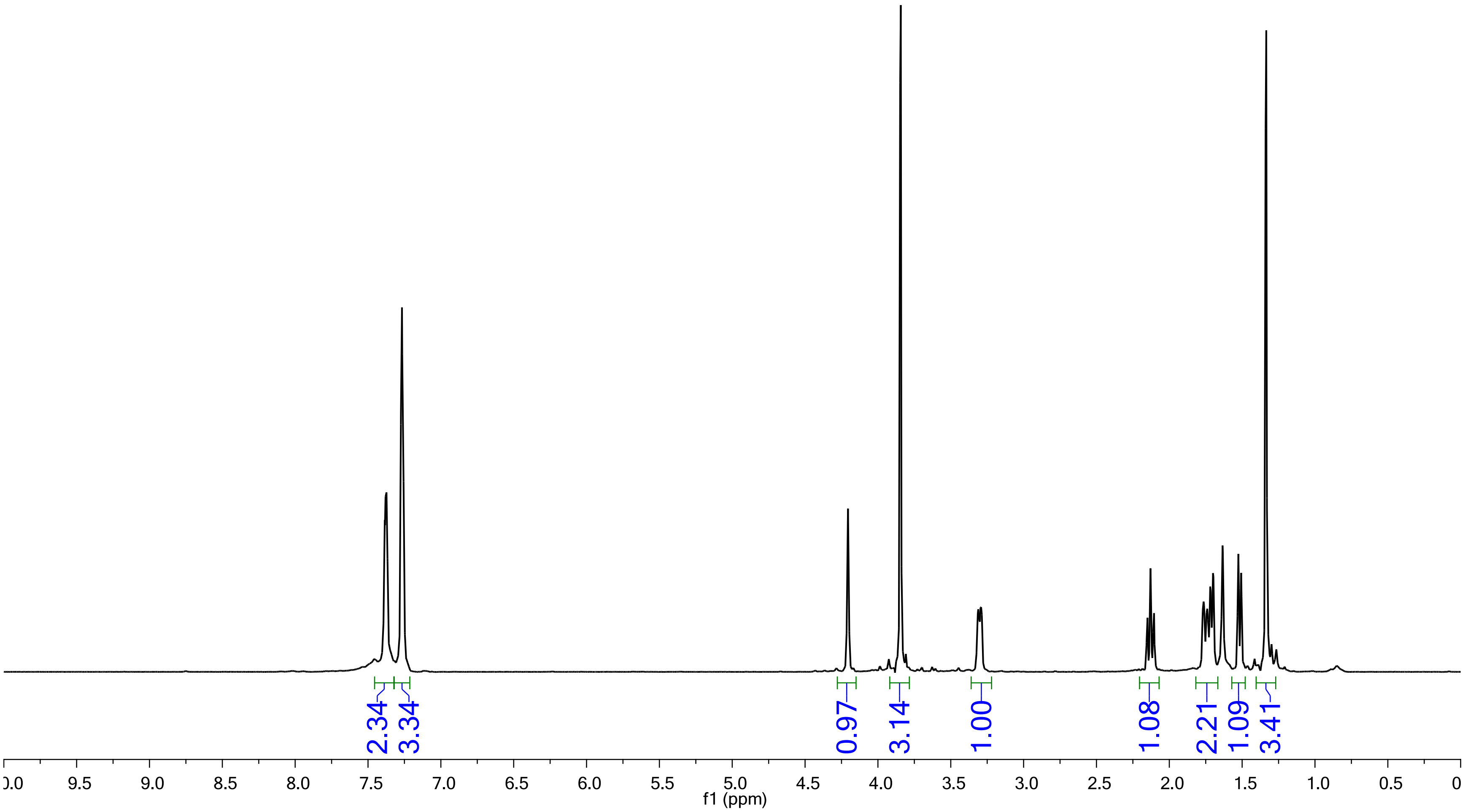
2.34
3.34
0.97
3.14
1.00
1.08
2.21
1.09
3.41
0.0
9.5
9.0
8.5
8.0
7.5
7.0
6.5
6.0
5.5
5.0
4.5
4.0
3.5
3.0
2.5
2.0
1.5
1.0
0.5
0
f1 (ppm)

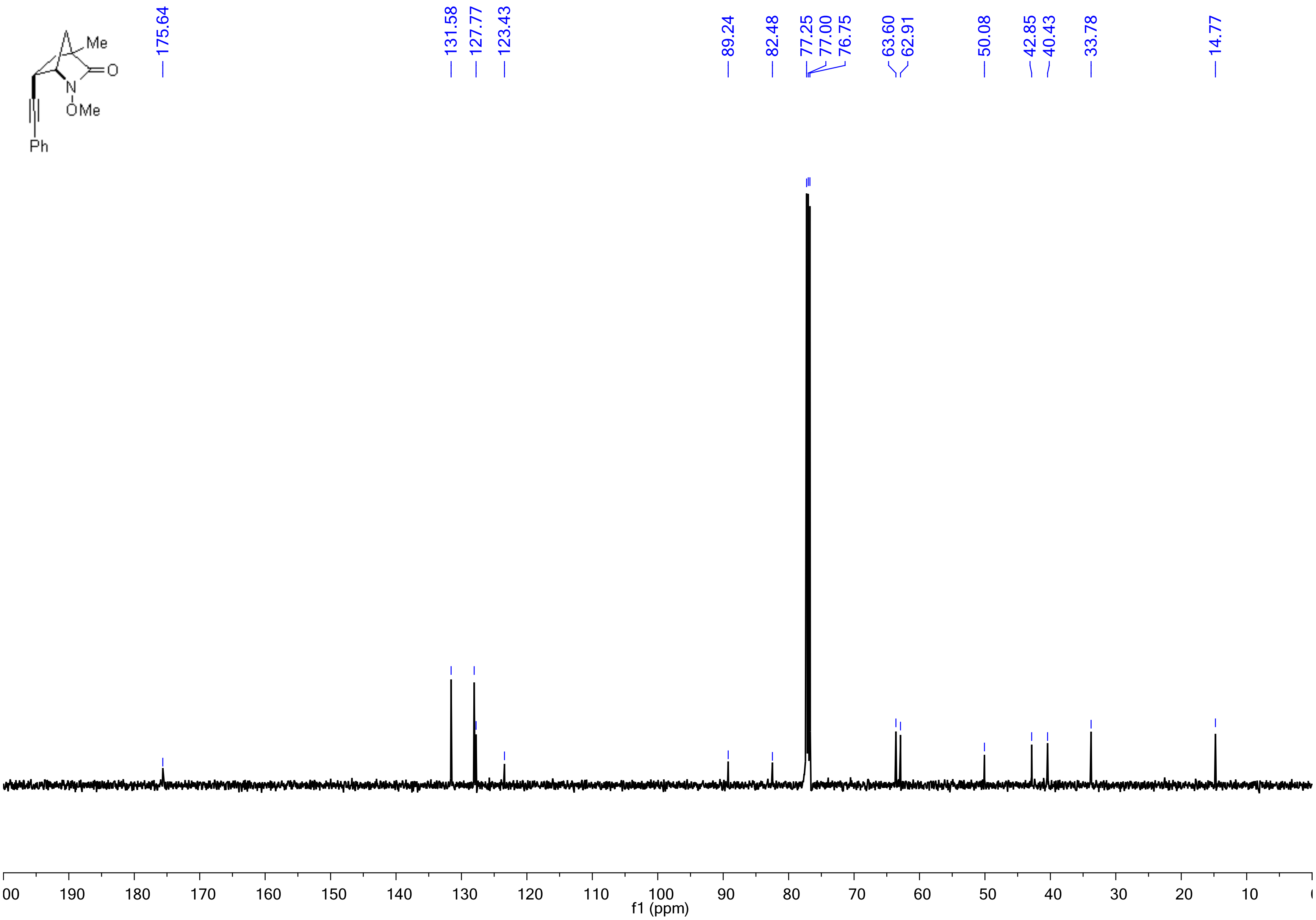

Me
O
N
OMe
Ph
175.64
131.58
127.77
123.43
89.24
82.48
77.25
77.00
76.75
63.60
62.91
50.08
42.85
40.43
33.78
14.77
00
190
180
170
160
150
140
130
120
110
100
90
80
70
60
50
40
30
20
10
f1 (ppm)

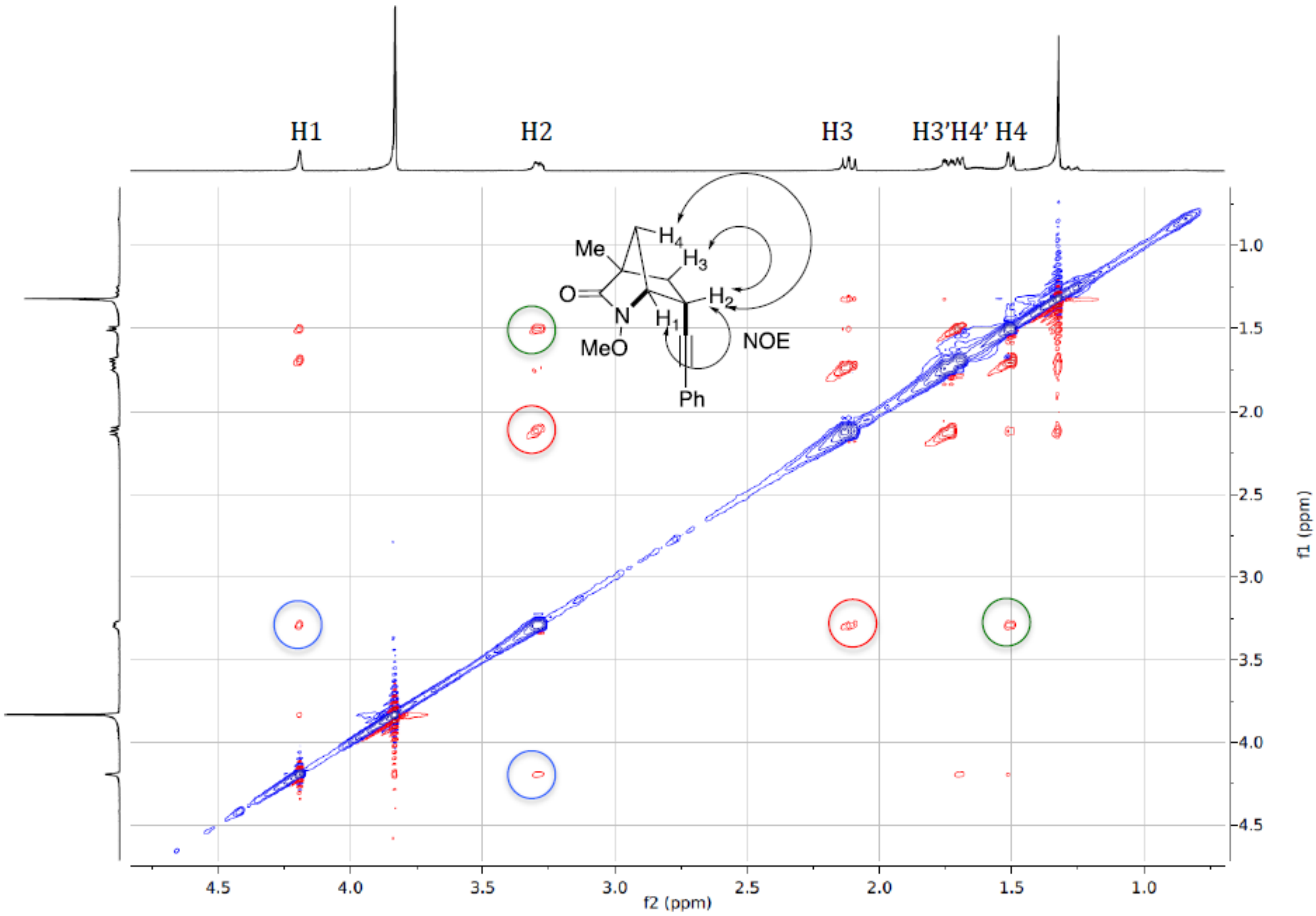
H1
H2
H3
H3'H4' H4
Me
H4
H3
O
N
H2
H1
MeO
NOE
Ph
1.0
1.5
2.0
2.5
3.0
3.5
4.0
4.5
f1 (ppm)
4.5
4.0
3.5
3.0
2.5
2.0
1.5
1.0
f2 (ppm)

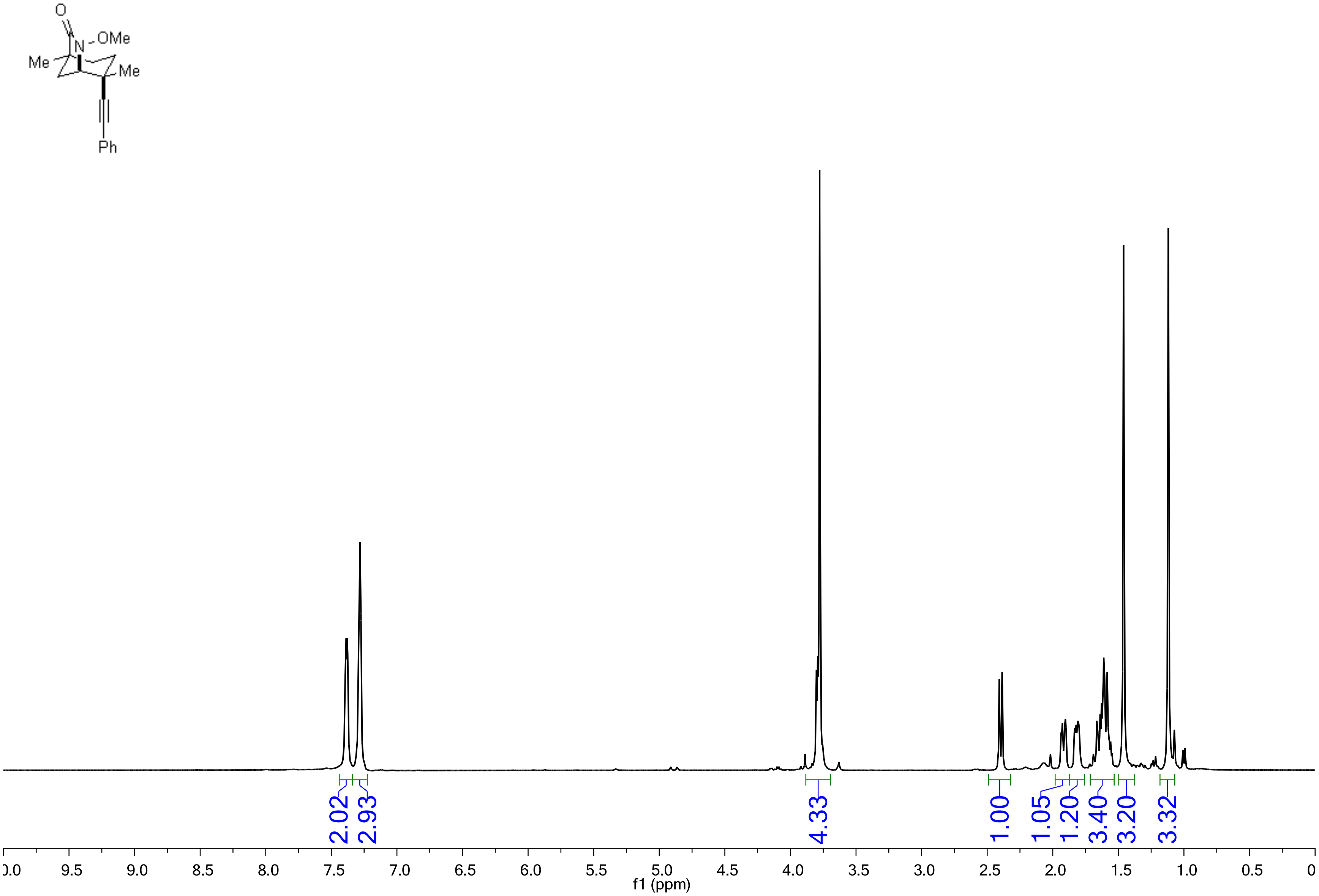
O
N
OMe
Me
Me
Ph
2.02
2.93
4.33
1.00
1.05
1.20
3.40
3.20
3.32
9.5
9.0
8.5
8.0
7.5
7.0
6.5
6.0
5.5
5.0
4.5
4.0
3.5
3.0
2.5
2.0
1.5
1.0
0.5
0
f1 (ppm)

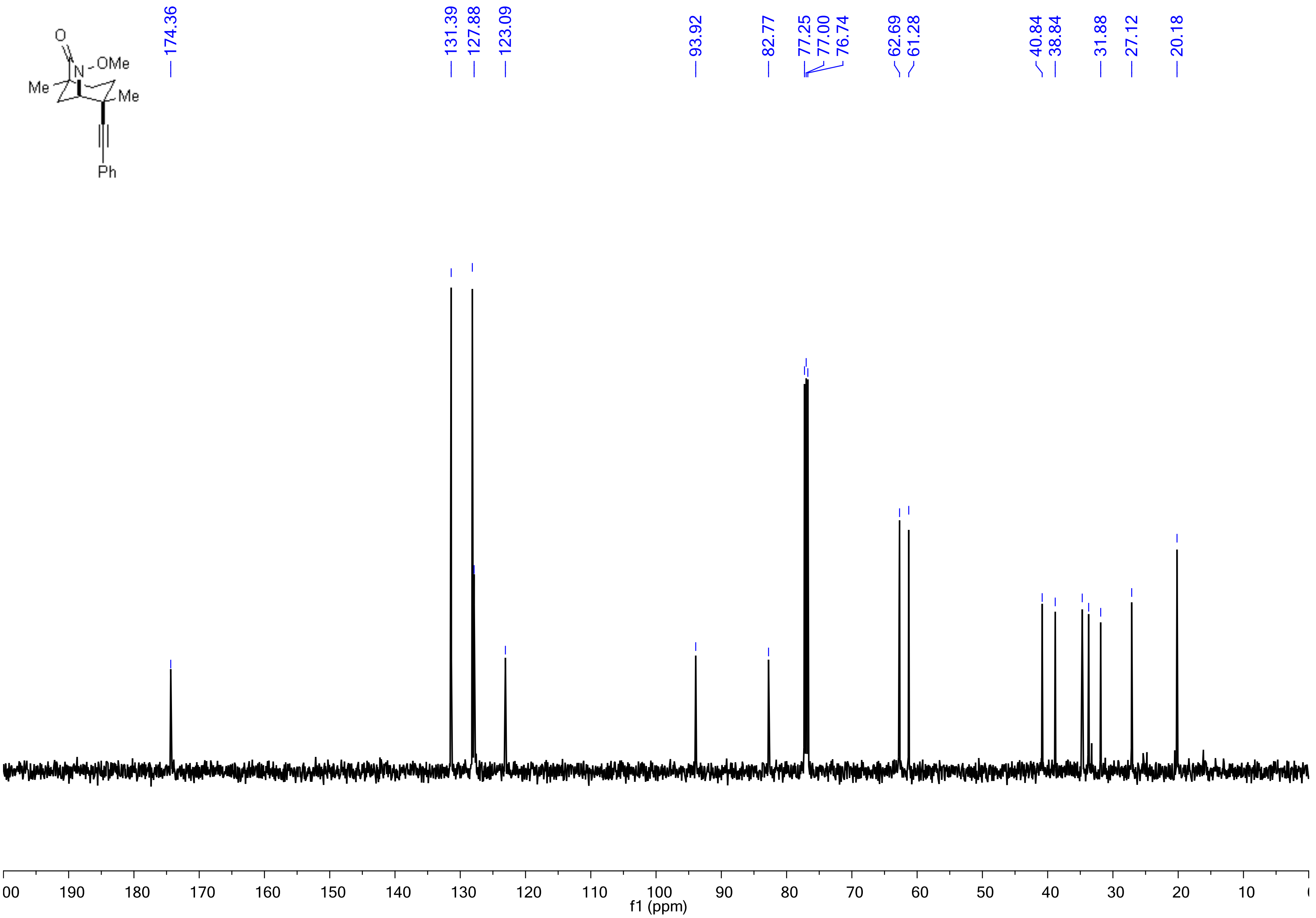
O
N
OMe
Me
Me
Ph
174.36
131.39
127.88
123.09
93.92
82.77
77.25
77.00
76.74
62.69
61.28
40.84
38.84
31.88
27.12
20.18
00
190
180
170
160
150
140
130
120
110
100
90
80
70
60
50
40
30
20
10
f1 (ppm)

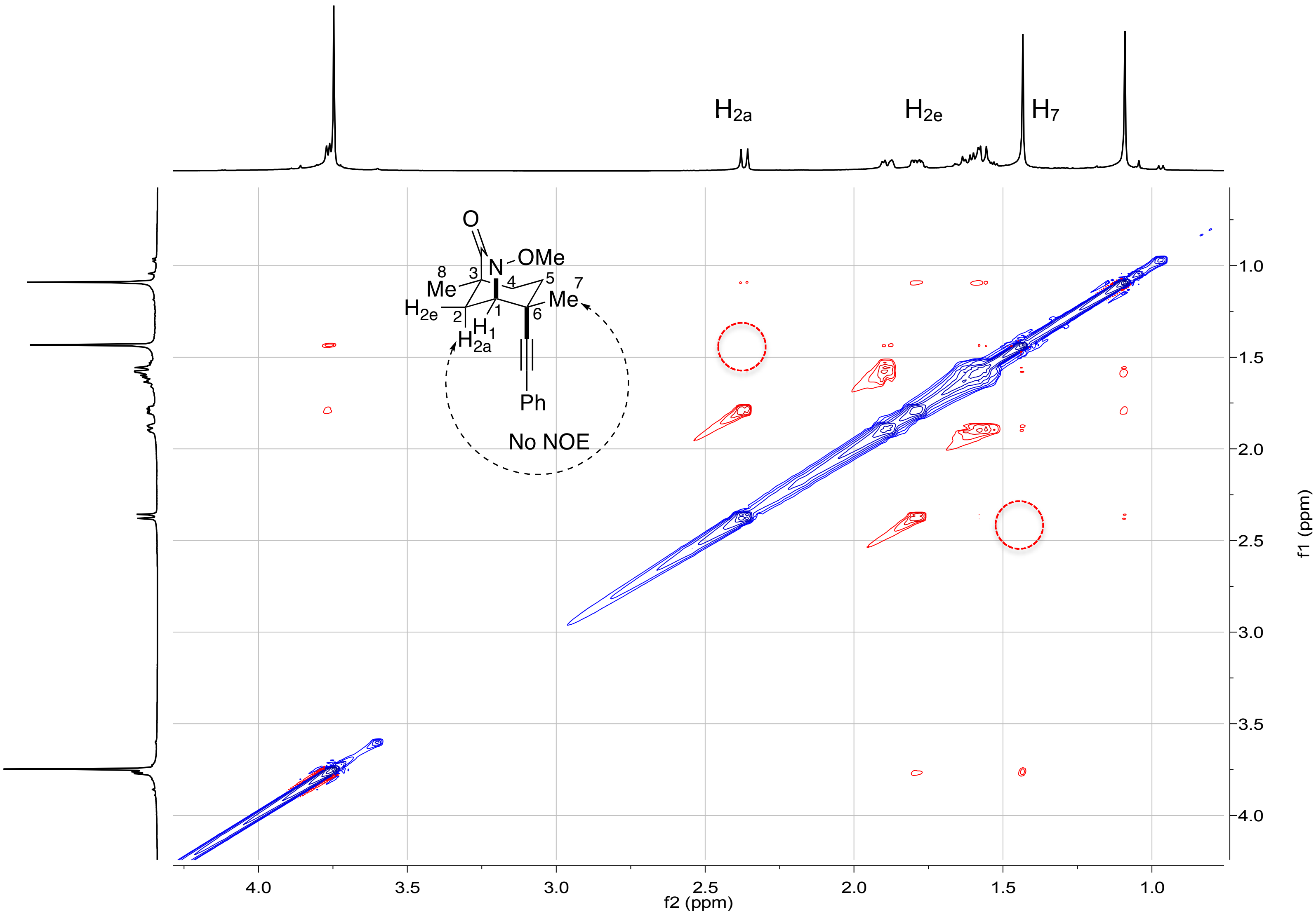

H2a
H2e
H7
O
N
OMe
8
3
Me
4
5
7
Me
H2e
2
1
6
H1
H2a
Ph
No NOE
4.0
3.5
3.0
2.5
2.0
1.5
1.0
f2 (ppm)
f1 (ppm)

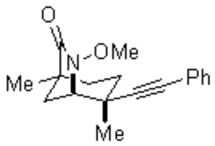
O
N
OMe
Me
Ph
Me

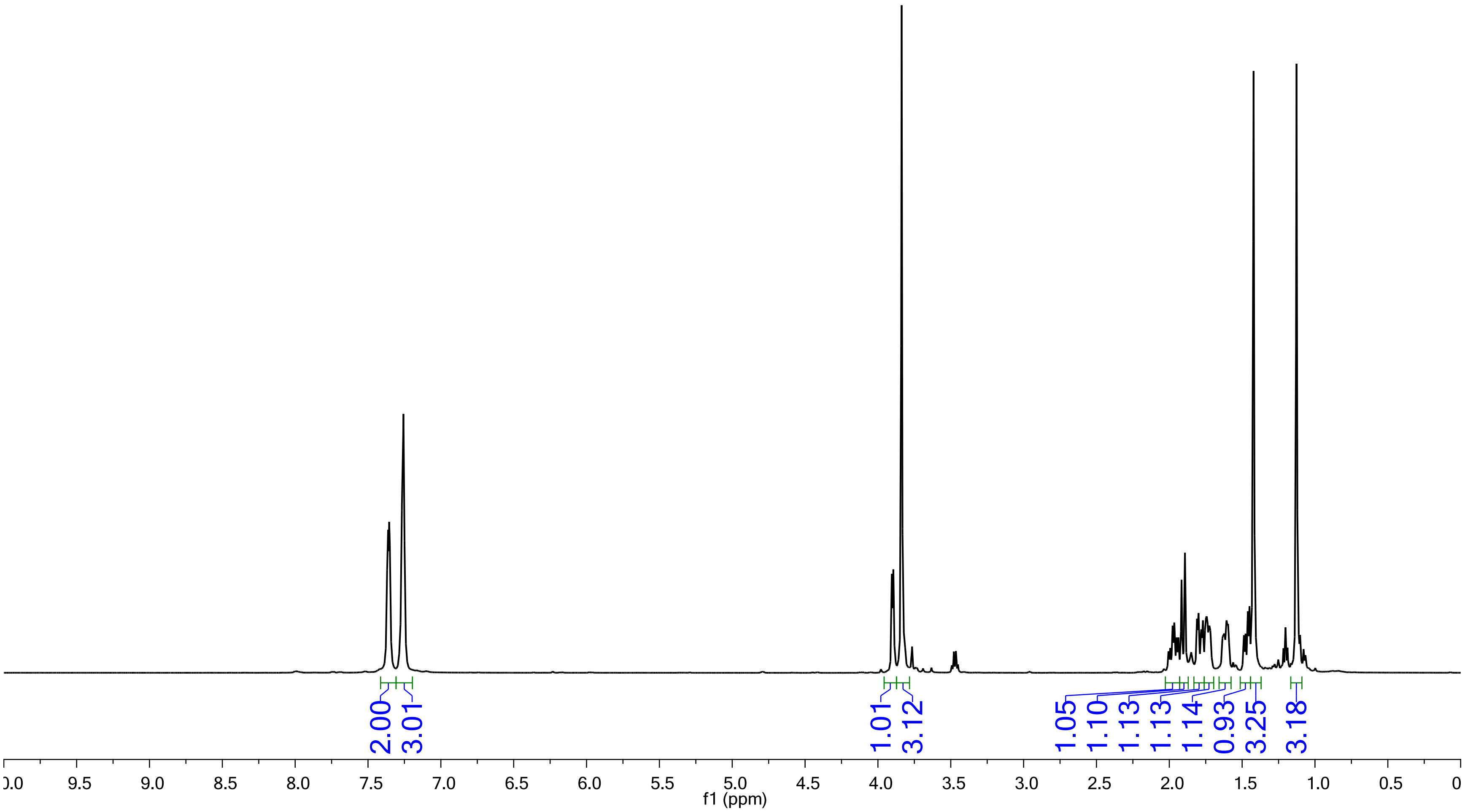
2.00
3.01
1.01
3.12
1.05
1.10
1.13
1.13
1.14
0.93
3.25
3.18
0.0
9.5
9.0
8.5
8.0
7.5
7.0
6.5
6.0
5.5
5.0
4.5
4.0
3.5
3.0
2.5
2.0
1.5
1.0
0.5
0
f1 (ppm)

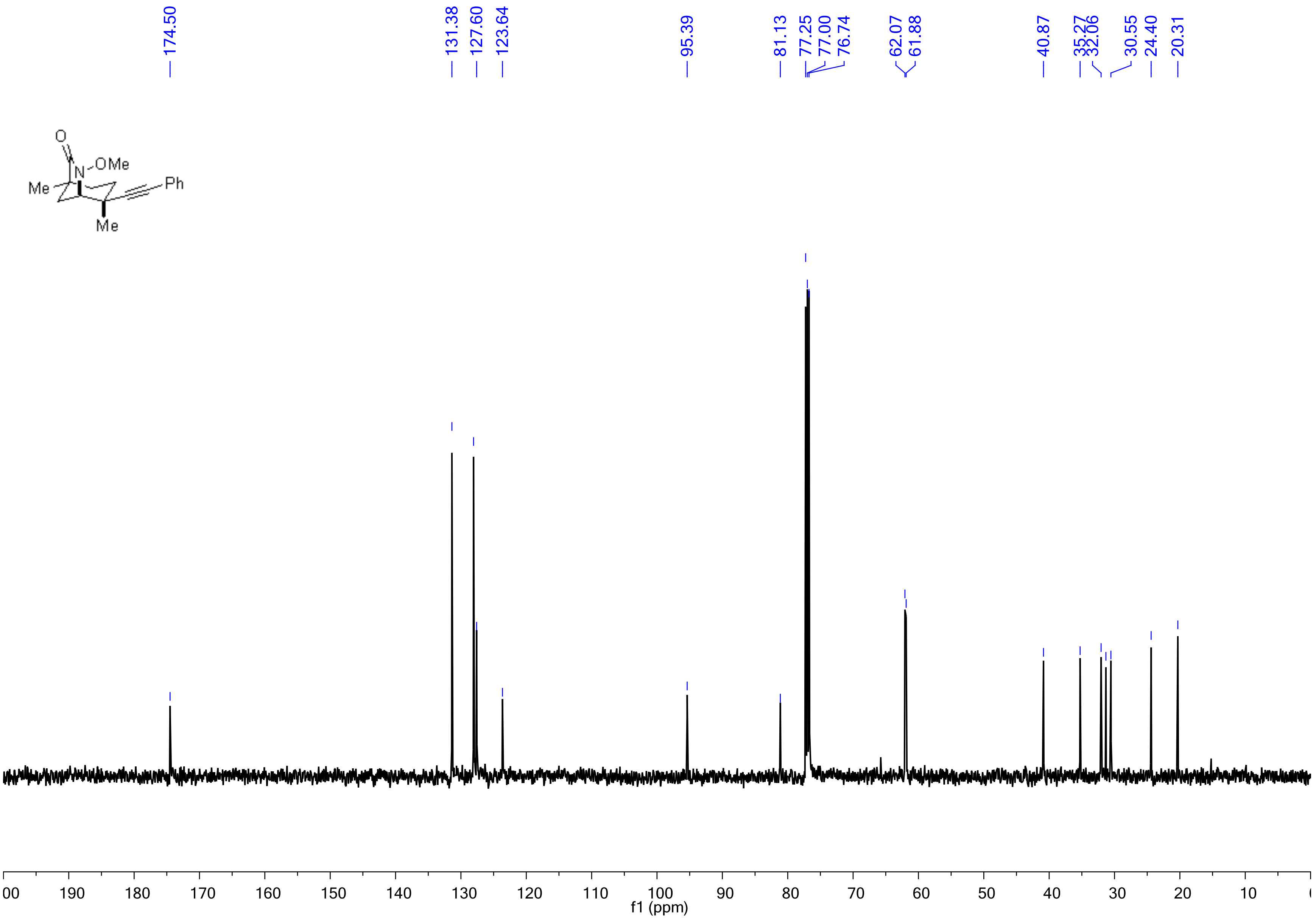

174.50
131.38
127.60
123.64
95.39
81.13
77.25
77.00
76.74
62.07
61.88
40.87
35.27
32.06
30.55
24.40
20.31
O
N
OMe
Me
Ph
Me
f1 (ppm)

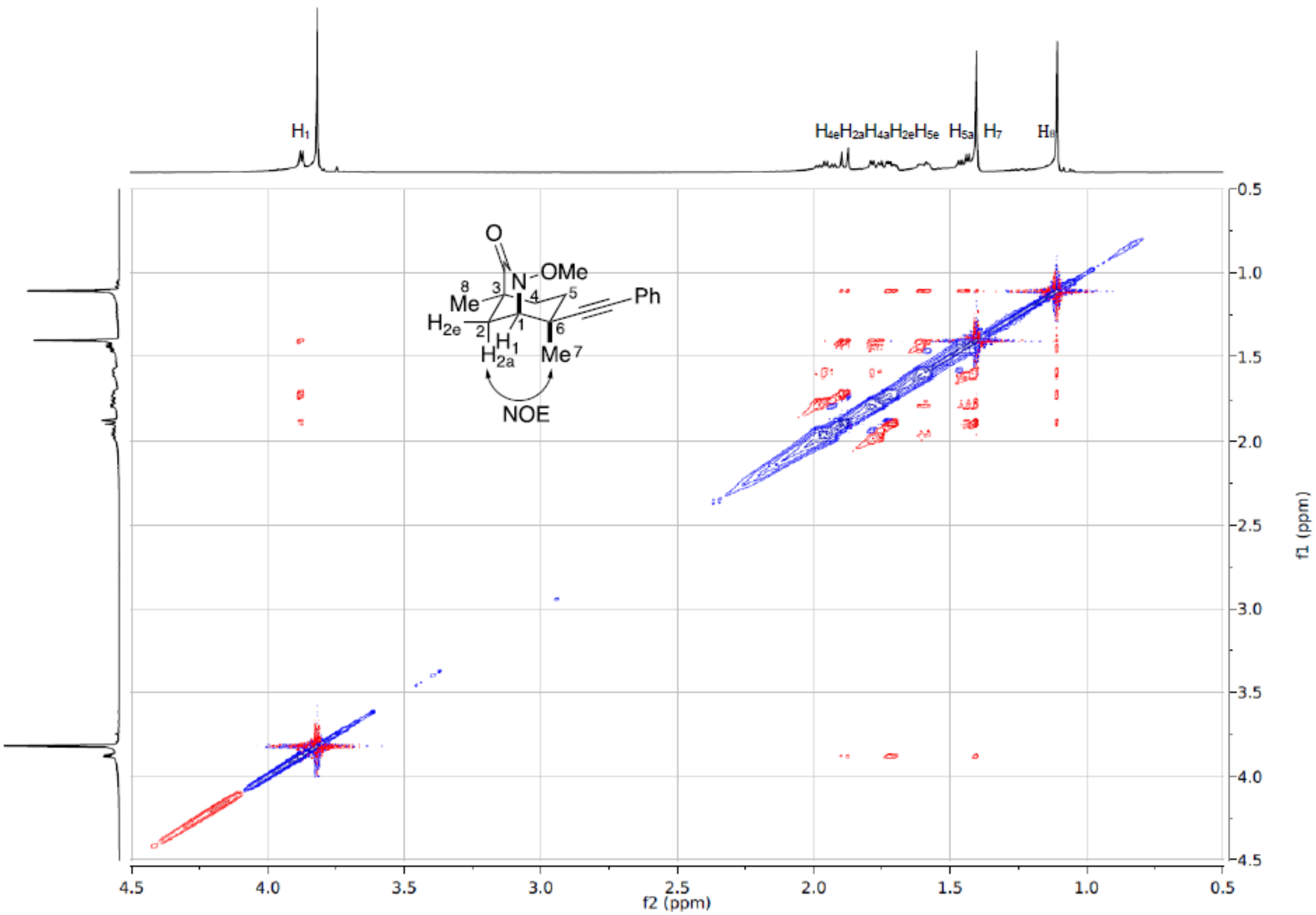
H1
H4eH2aH4aH2eH5e
H5a
H7
H8
O
N
OMe
Ph
Me
H2e
H1
H2a
Me7
NOE
f2 (ppm)
f1 (ppm)

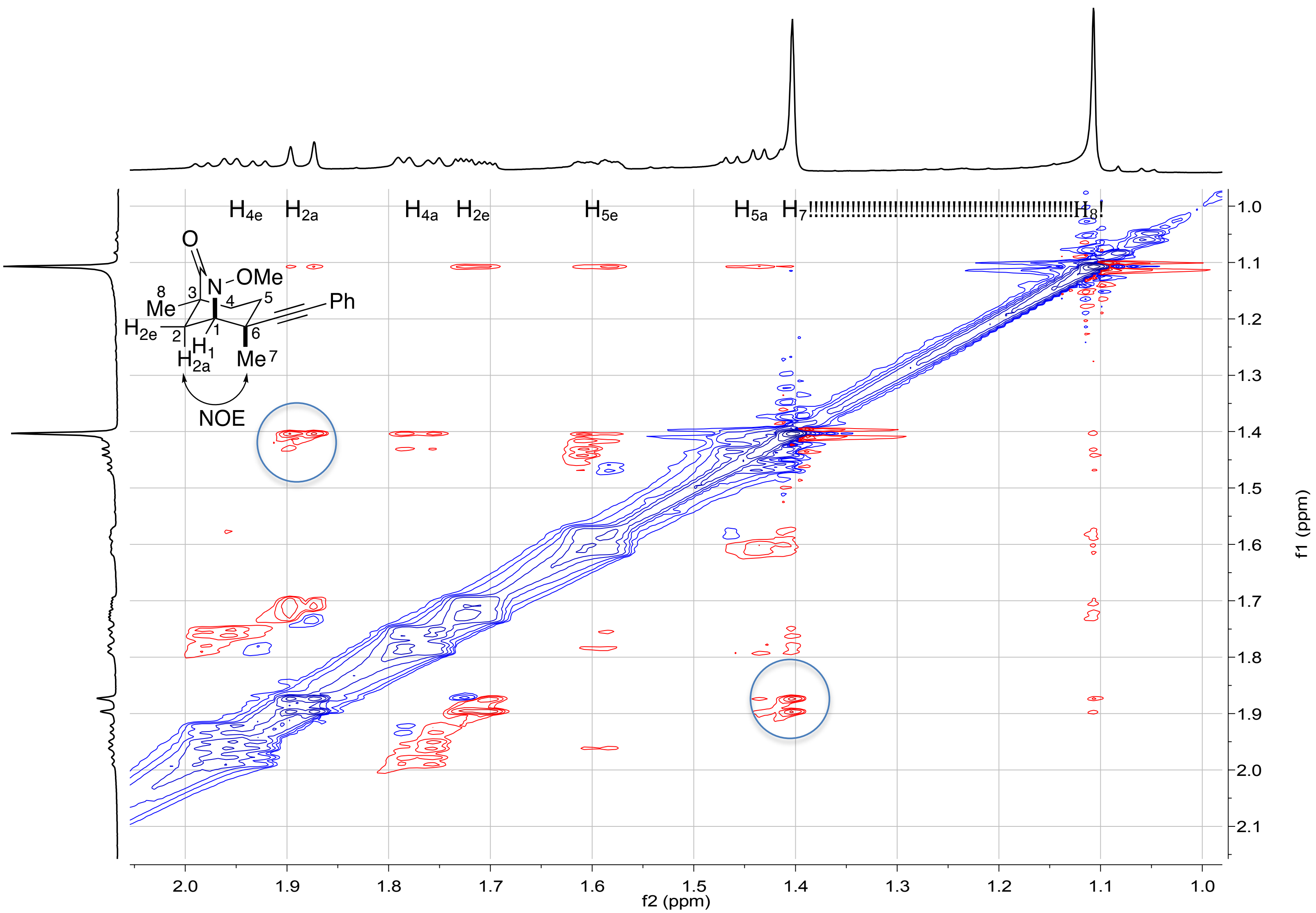

H4e H2a H4a H2e H5e H5a H7!!H8!
O
N-OMe
Me
H2e
H1
H2a
Me 7
Ph
NOE
f2 (ppm)
f1 (ppm)

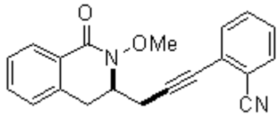
O
N
OMe
CN

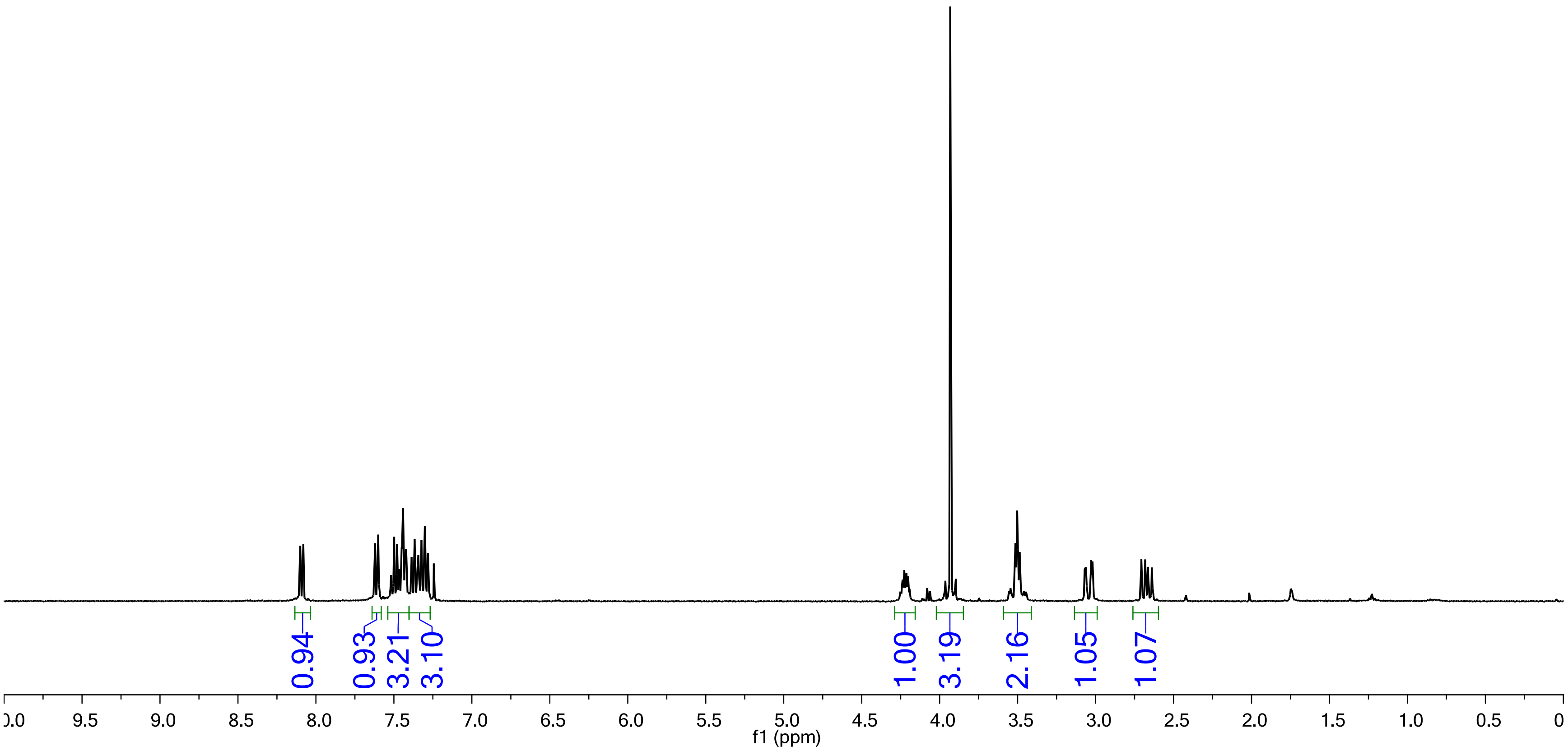
0.94
0.93
3.21
3.10
1.00
3.19
2.16
1.05
1.07
10.0
9.5
9.0
8.5
8.0
7.5
7.0
6.5
6.0
5.5
5.0
4.5
4.0
3.5
3.0
2.5
2.0
1.5
1.0
0.5
0
f1 (ppm)

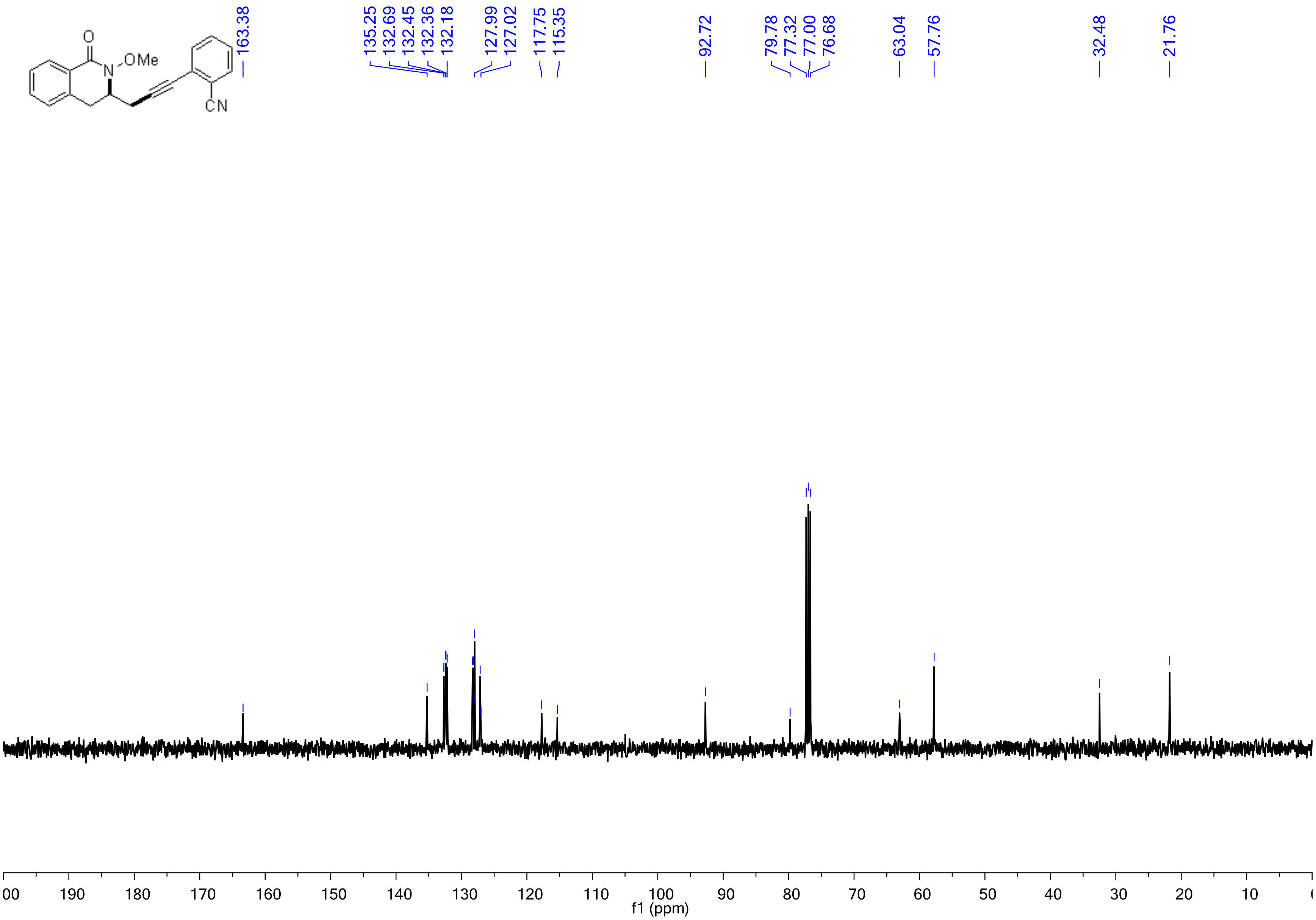
O
N
OMe
CN
163.38
135.25
132.69
132.45
132.36
132.18
127.99
127.02
117.75
115.35
92.72
79.78
77.32
77.00
76.68
63.04
57.76
32.48
21.76
00
190
180
170
160
150
140
130
120
110
100
90
80
70
60
50
40
30
20
10
f1 (ppm)

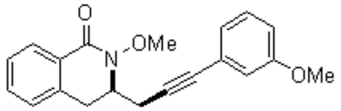
O
N
OMe
OMe

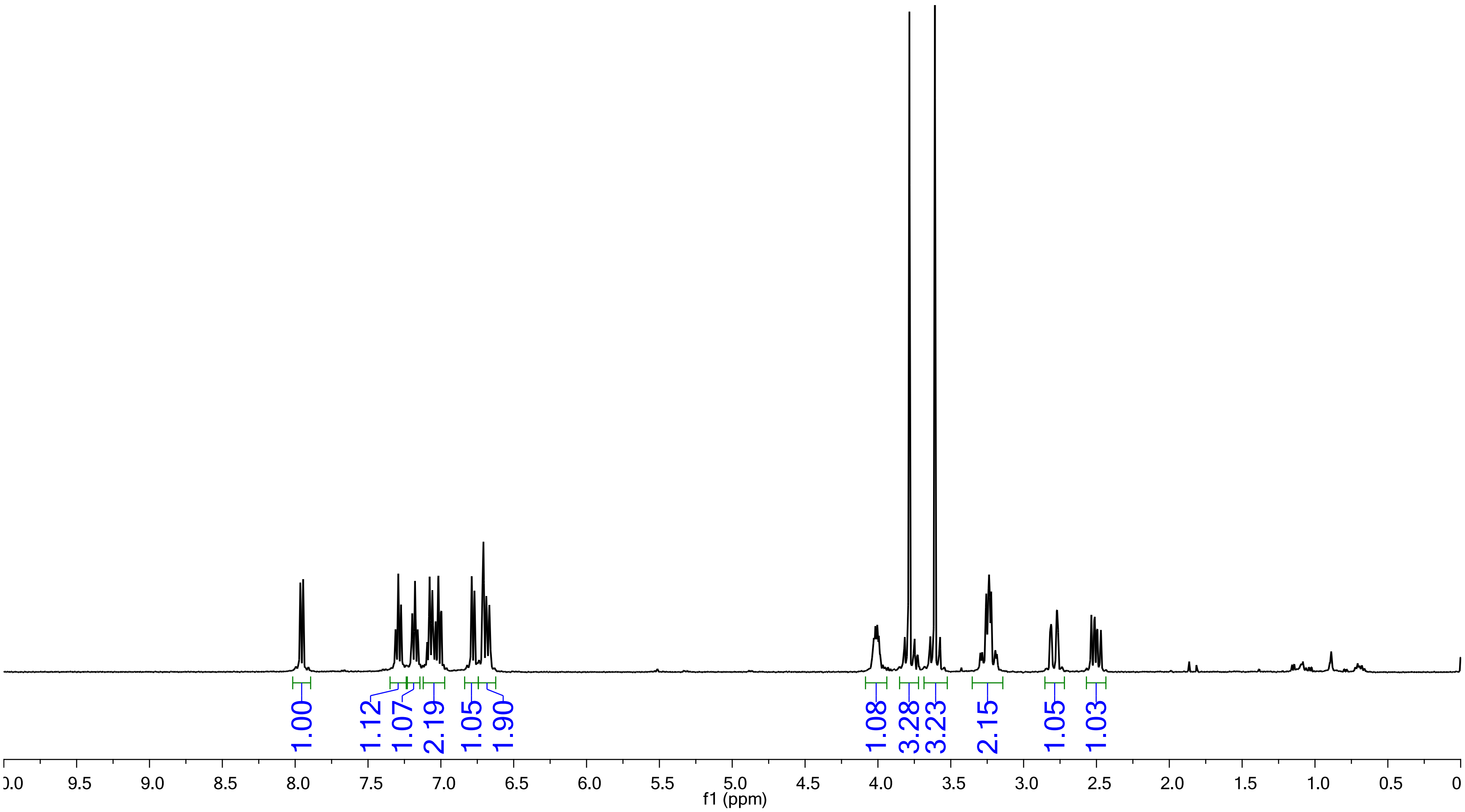
1.00
1.12
1.07
2.19
1.05
1.90
1.08
3.28
3.23
2.15
1.05
1.03
f1 (ppm)

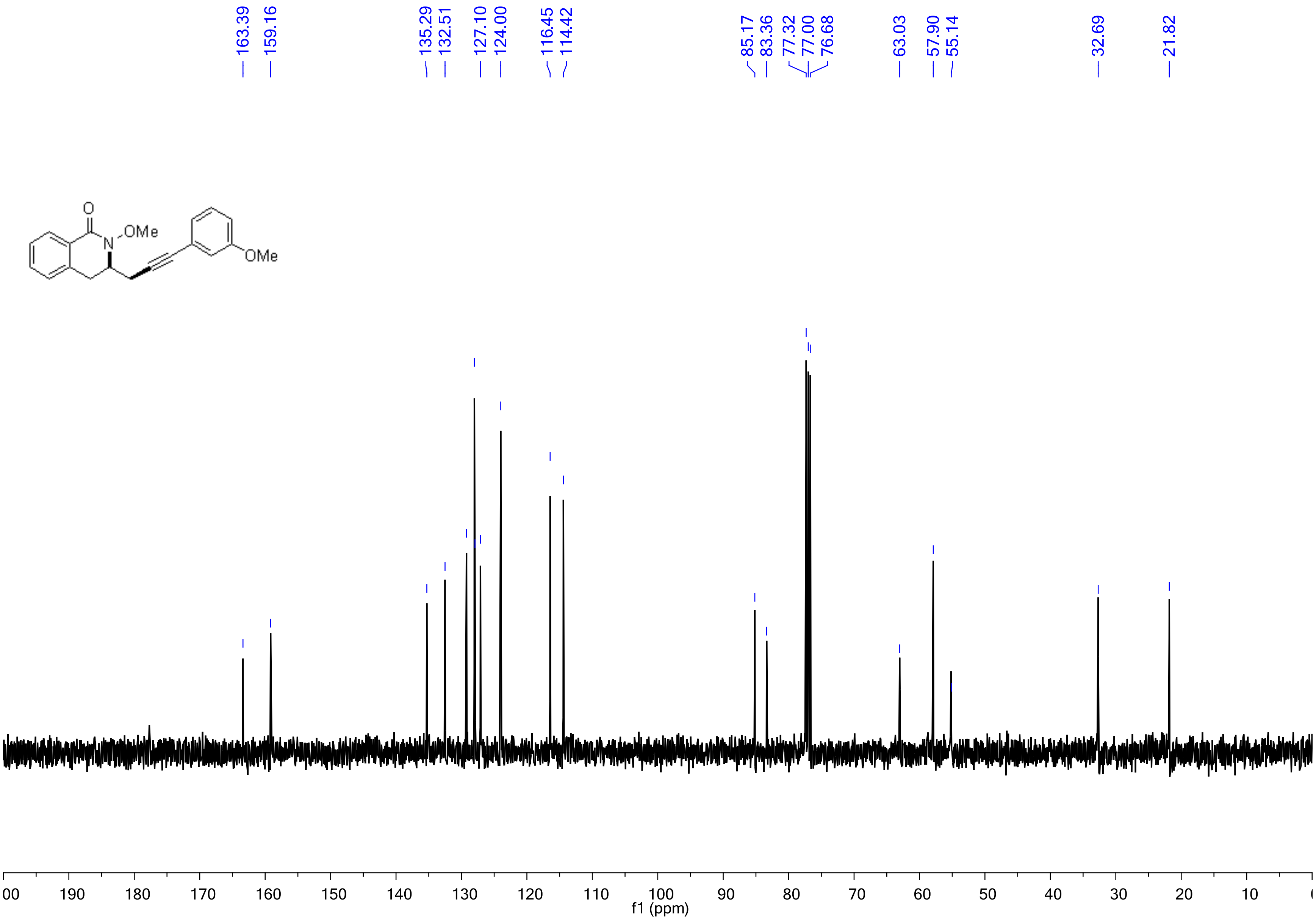
163.39
159.16
135.29
132.51
127.10
124.00
116.45
114.42
85.17
83.36
77.32
77.00
76.68
63.03
57.90
55.14
32.69
21.82
O
N
OMe
OMe
00
190
180
170
160
150
140
130
120
110
100
90
80
70
60
50
40
30
20
10
f1 (ppm)

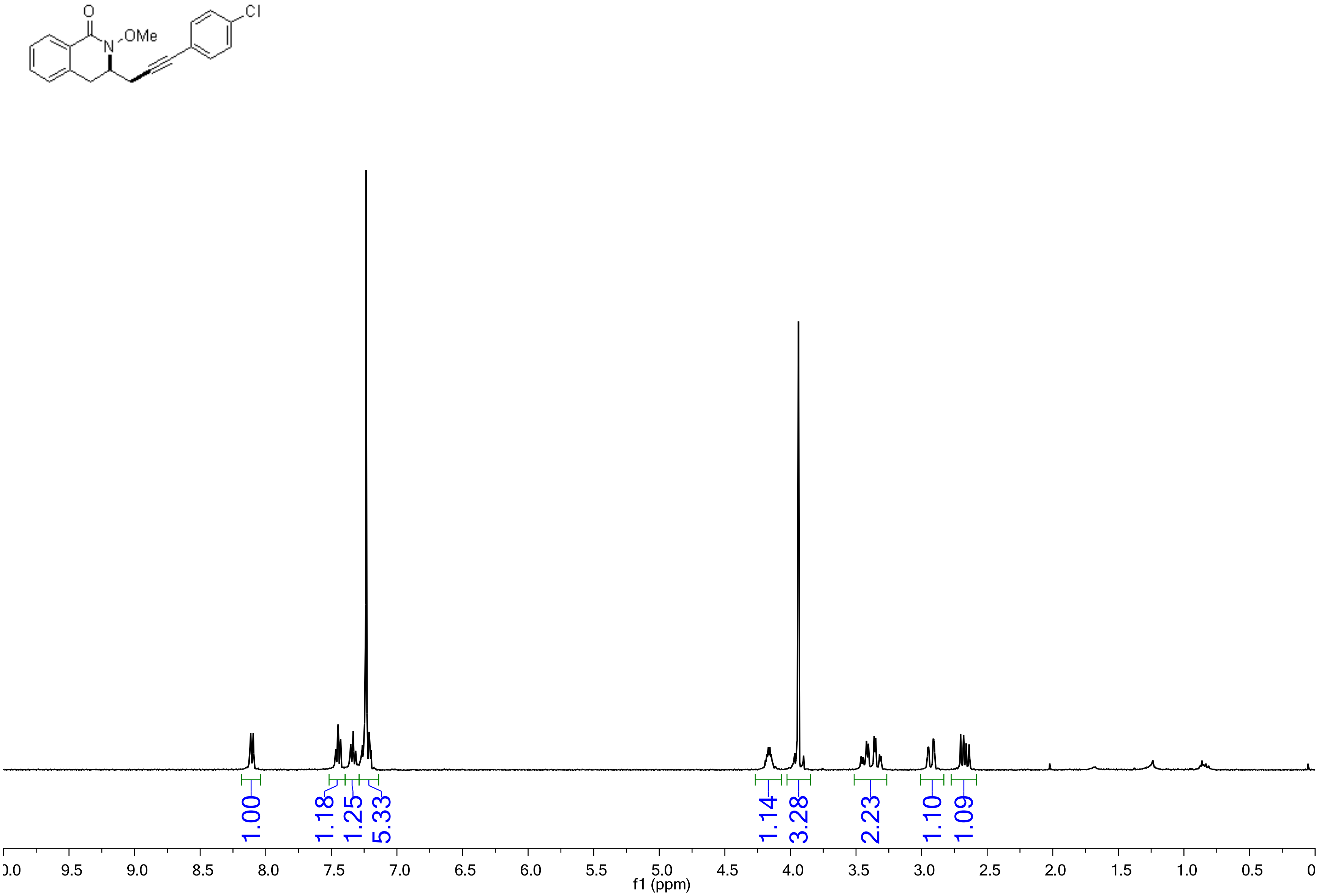
O
N
OMe
Cl
1.00
1.18
1.25
5.33
1.14
3.28
2.23
1.10
1.09
9.5
9.0
8.5
8.0
7.5
7.0
6.5
6.0
5.5
5.0
4.5
4.0
3.5
3.0
2.5
2.0
1.5
1.0
0.5
0
f1 (ppm)

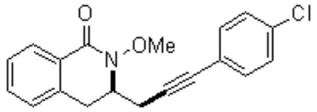
O
N
OMe
Cl

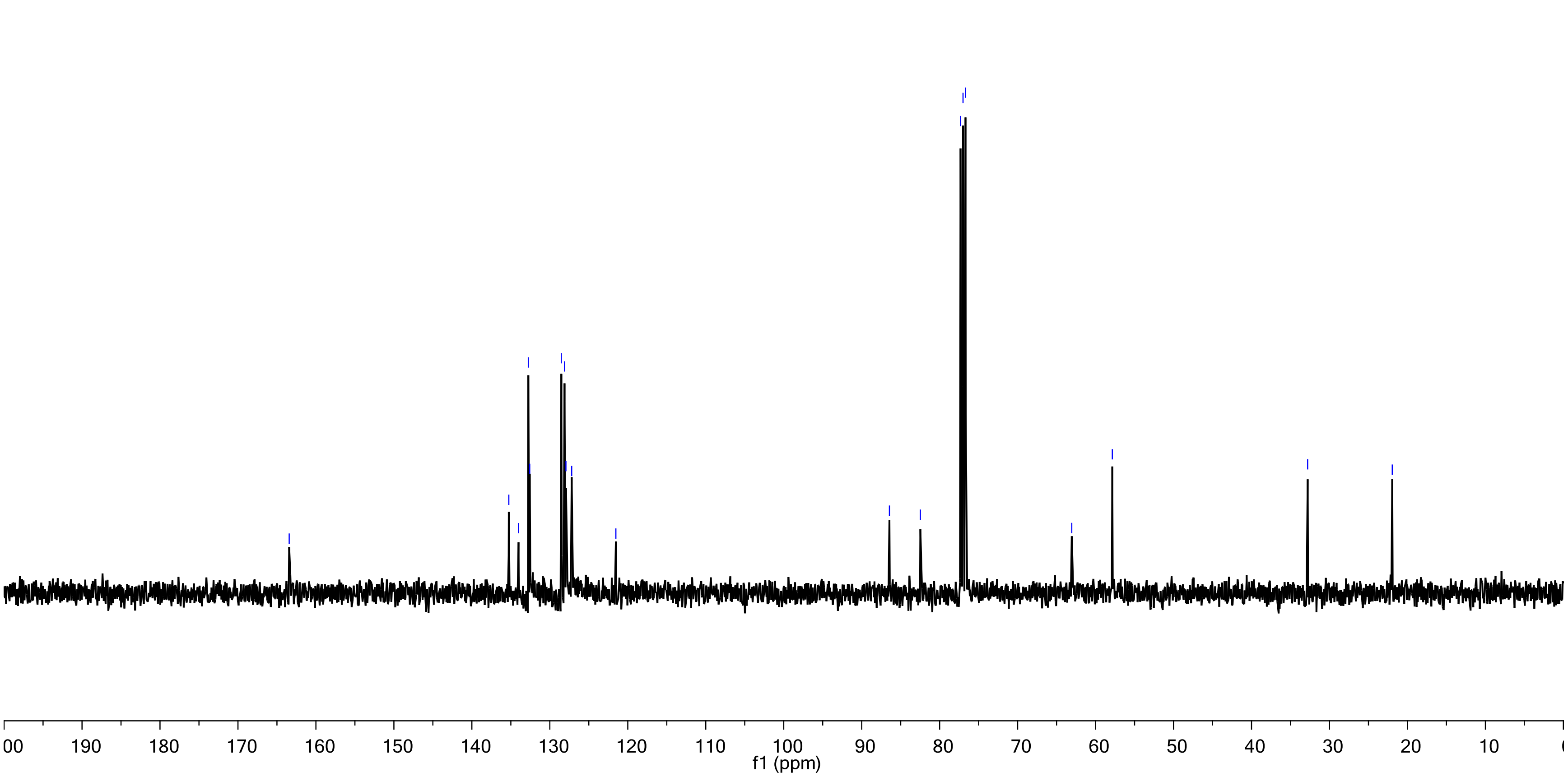
163.43
135.26
134.01
132.76
132.58
127.19
121.53
86.44
82.47
77.32
77.00
76.68
63.05
57.85
32.80
21.95
00
190
180
170
160
150
140
130
120
110
100
90
80
70
60
50
40
30
20
10
f1 (ppm)

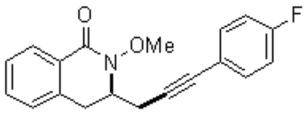

O
N
OMe
F

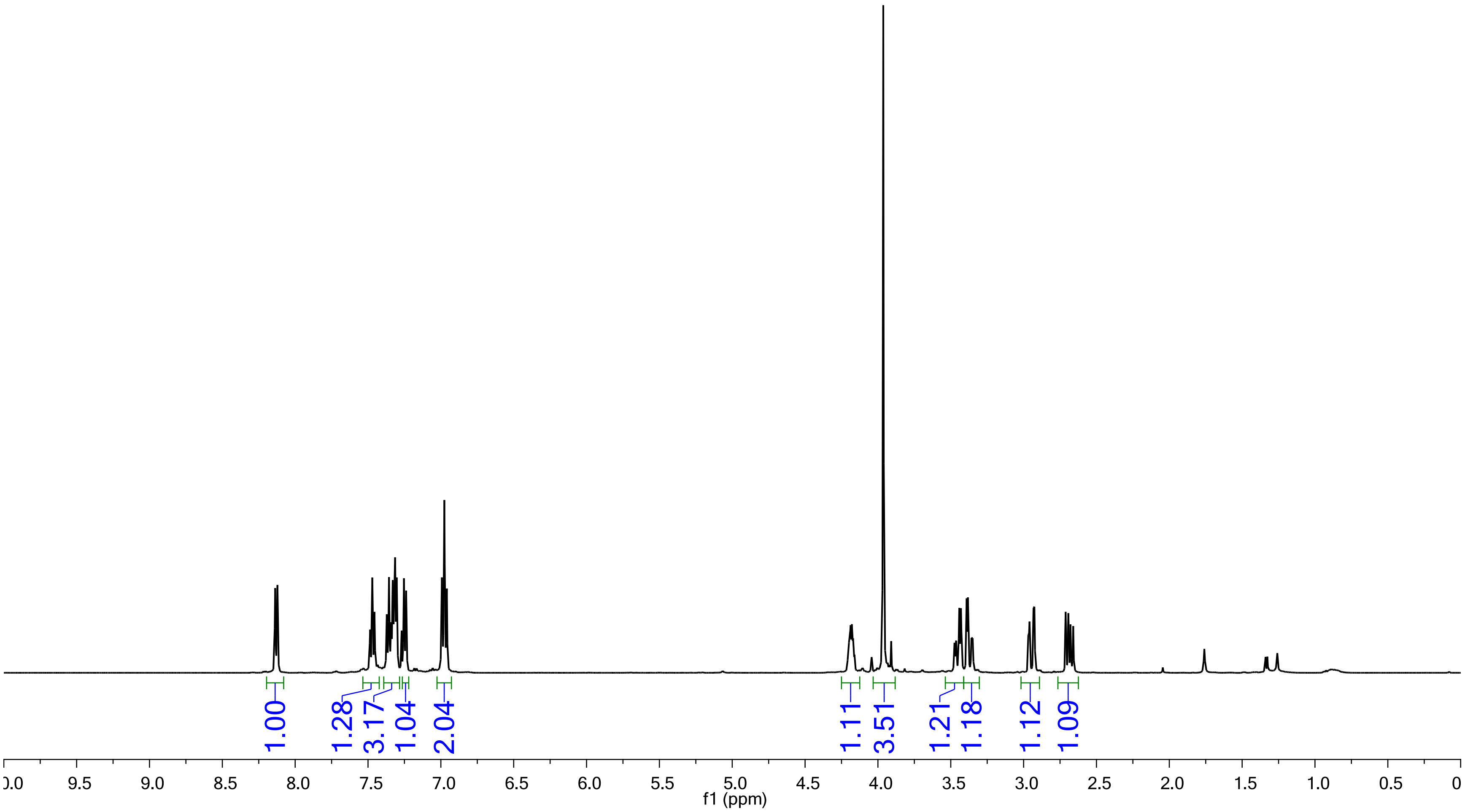

1.00
1.28
3.17
1.04
2.04
1.11
3.51
1.21
1.18
1.12
1.09
9.5
9.0
8.5
8.0
7.5
7.0
6.5
6.0
5.5
5.0
4.5
4.0
3.5
3.0
2.5
2.0
1.5
1.0
0.5
0
f1 (ppm)

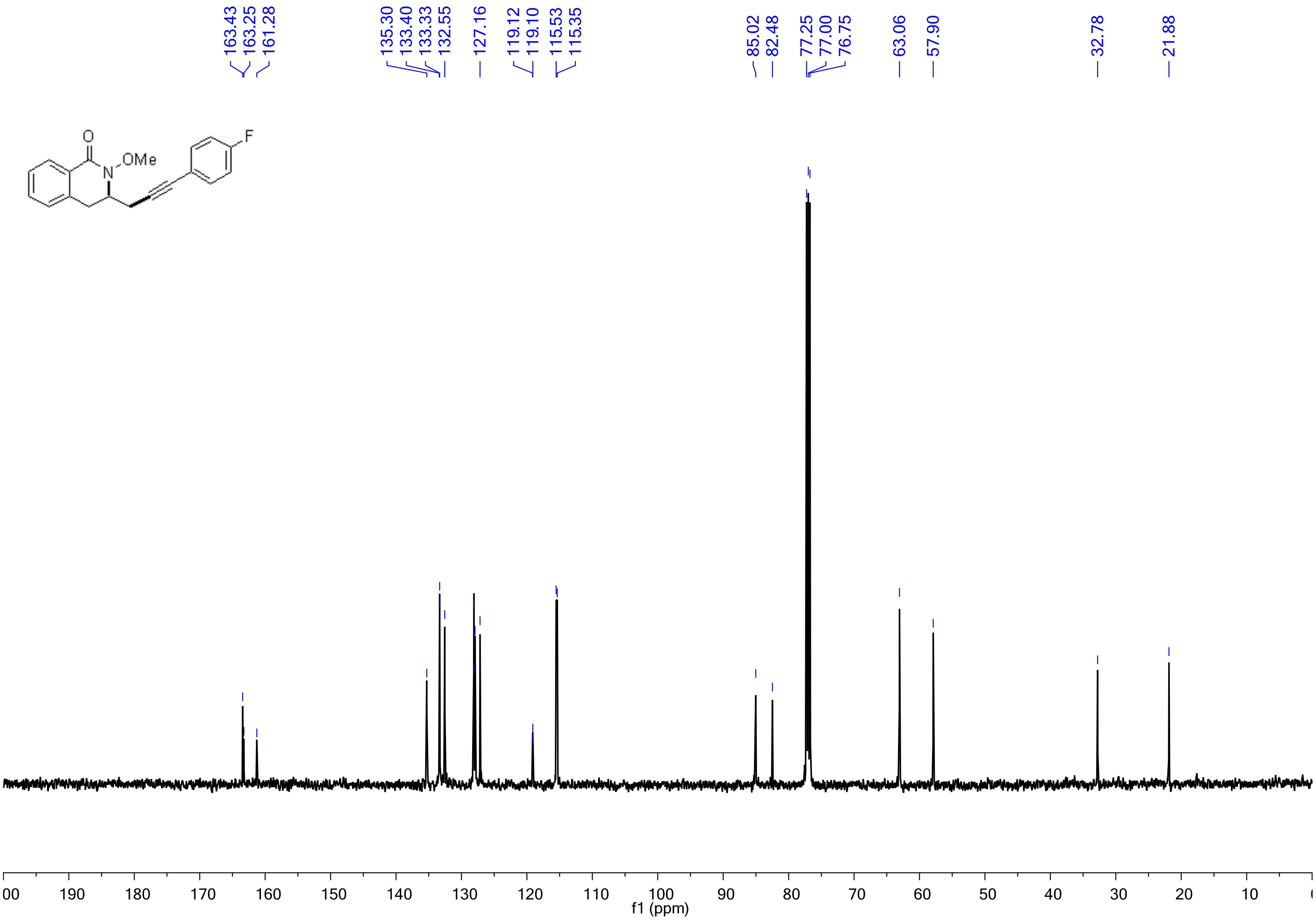
163.43
163.25
161.28
135.30
133.40
133.33
132.55
127.16
119.12
119.10
115.53
115.35
85.02
82.48
77.25
77.00
76.75
63.06
57.90
32.78
21.88
O
N
OMe
F
f1 (ppm)

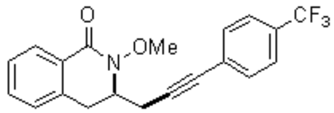
O
N
OMe
CF3

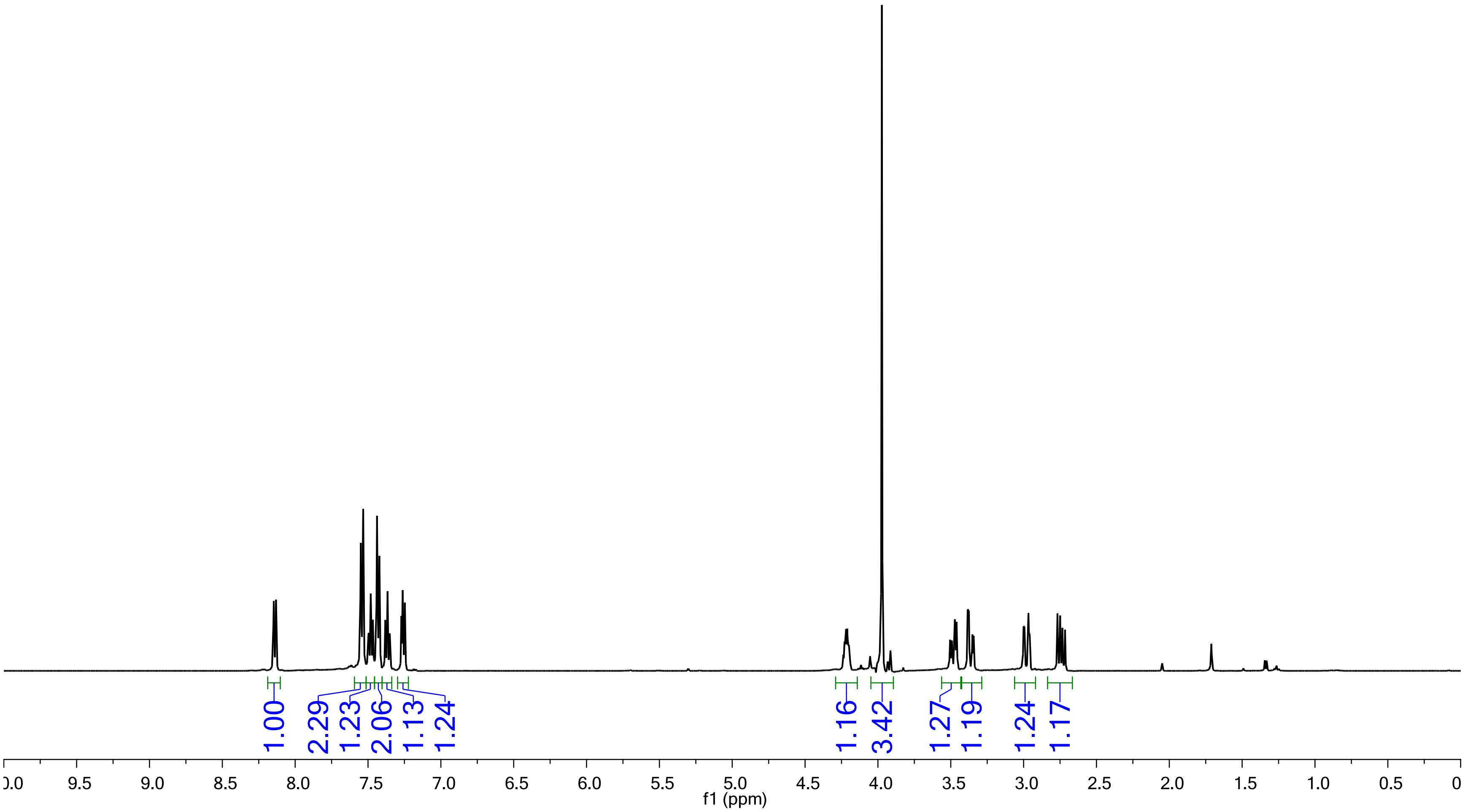
1.00
2.29
1.23
2.06
1.13
1.24
1.16
3.42
1.27
1.19
1.24
1.17
9.5
9.0
8.5
8.0
7.5
7.0
6.5
6.0
5.5
5.0
4.5
4.0
3.5
3.0
2.5
2.0
1.5
1.0
0.5
0
f1 (ppm)

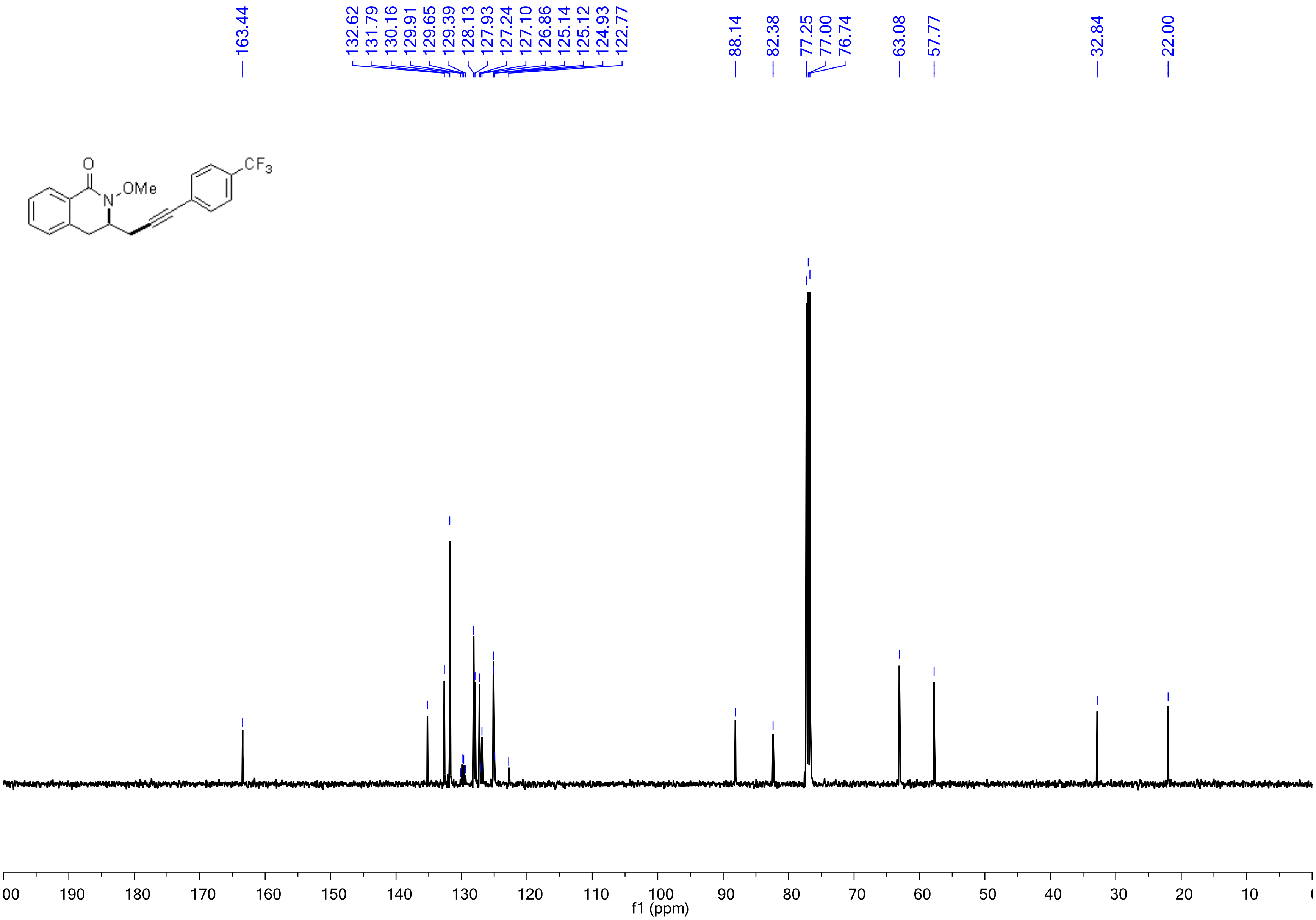

163.44
132.62
131.79
130.16
129.91
129.65
129.39
128.13
127.93
127.24
127.10
126.86
125.14
125.12
124.93
122.77
88.14
82.38
77.25
77.00
76.74
63.08
57.77
32.84
22.00
O
N
OMe
CF3
f1 (ppm)

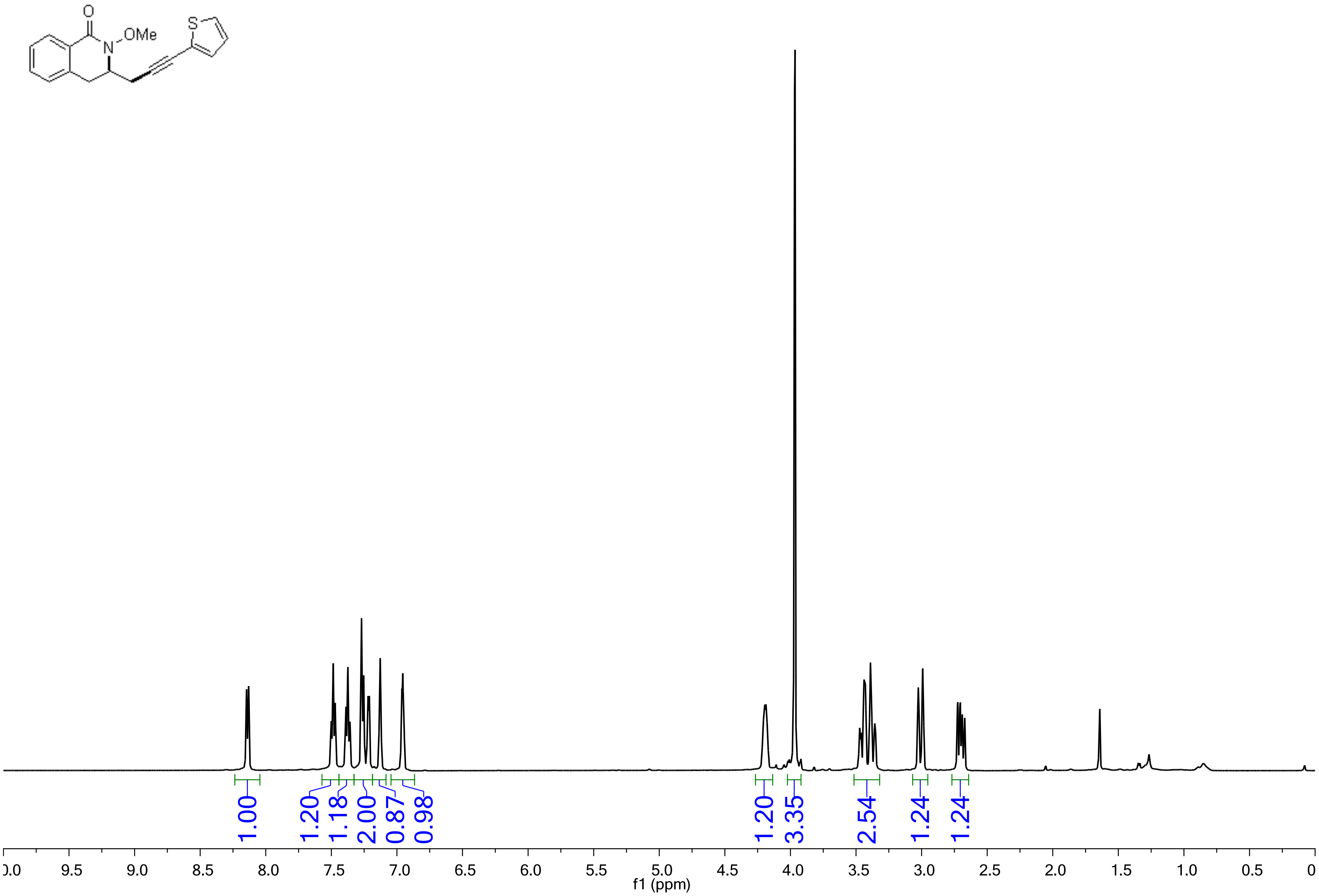
O
N
OMe
S
1.00
1.20
1.18
2.00
0.87
0.98
1.20
3.35
2.54
1.24
1.24
9.5
9.0
8.5
8.0
7.5
7.0
6.5
6.0
5.5
5.0
4.5
4.0
3.5
3.0
2.5
2.0
1.5
1.0
0.5
0
f1 (ppm)

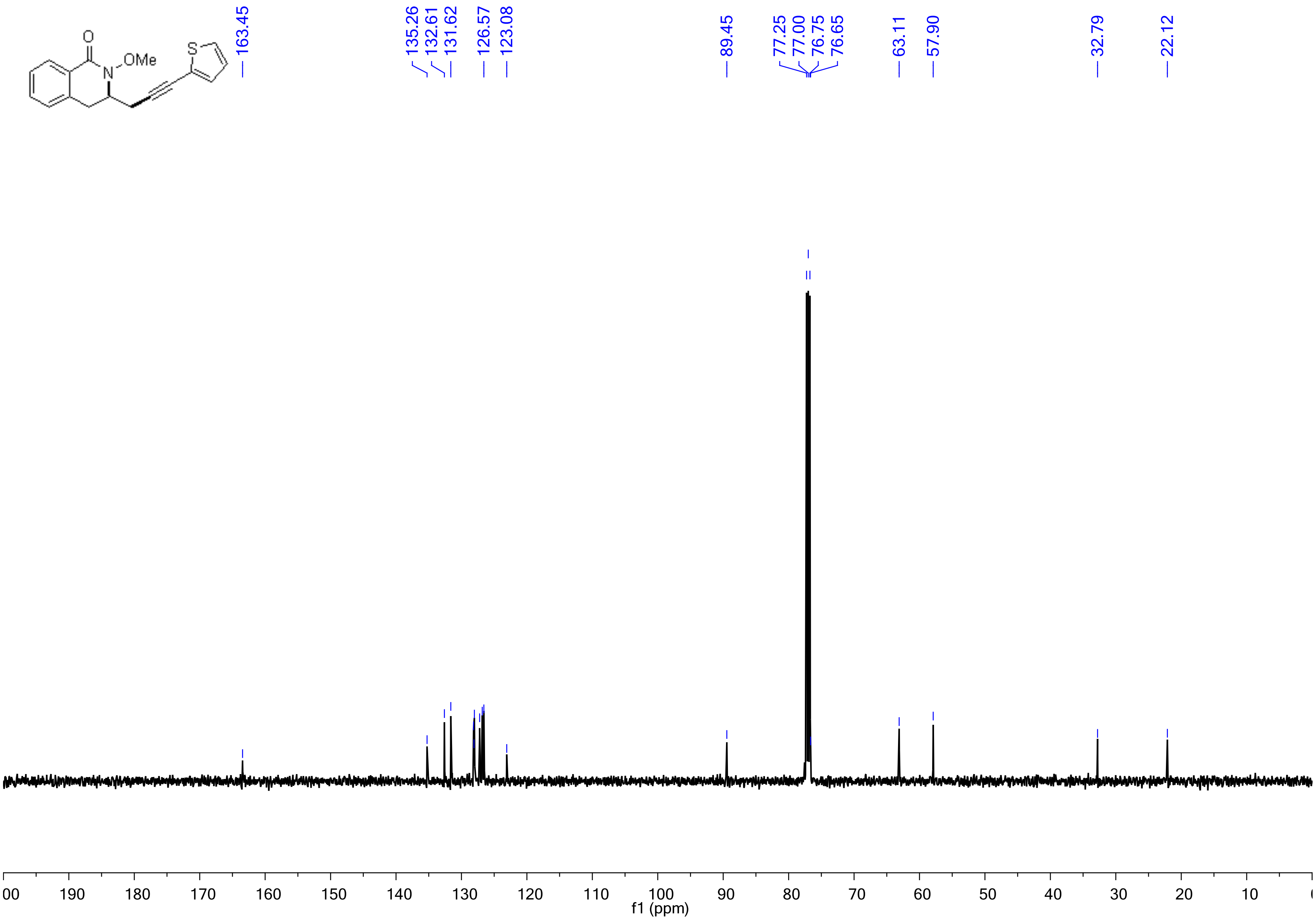
O
N
OMe
S
163.45
135.26
132.61
131.62
126.57
123.08
89.45
77.25
77.00
76.75
76.65
63.11
57.90
32.79
22.12
00
190
180
170
160
150
140
130
120
110
100
90
80
70
60
50
40
30
20
10
f1 (ppm)

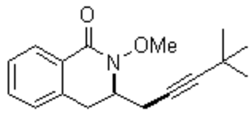
O
N
OMe

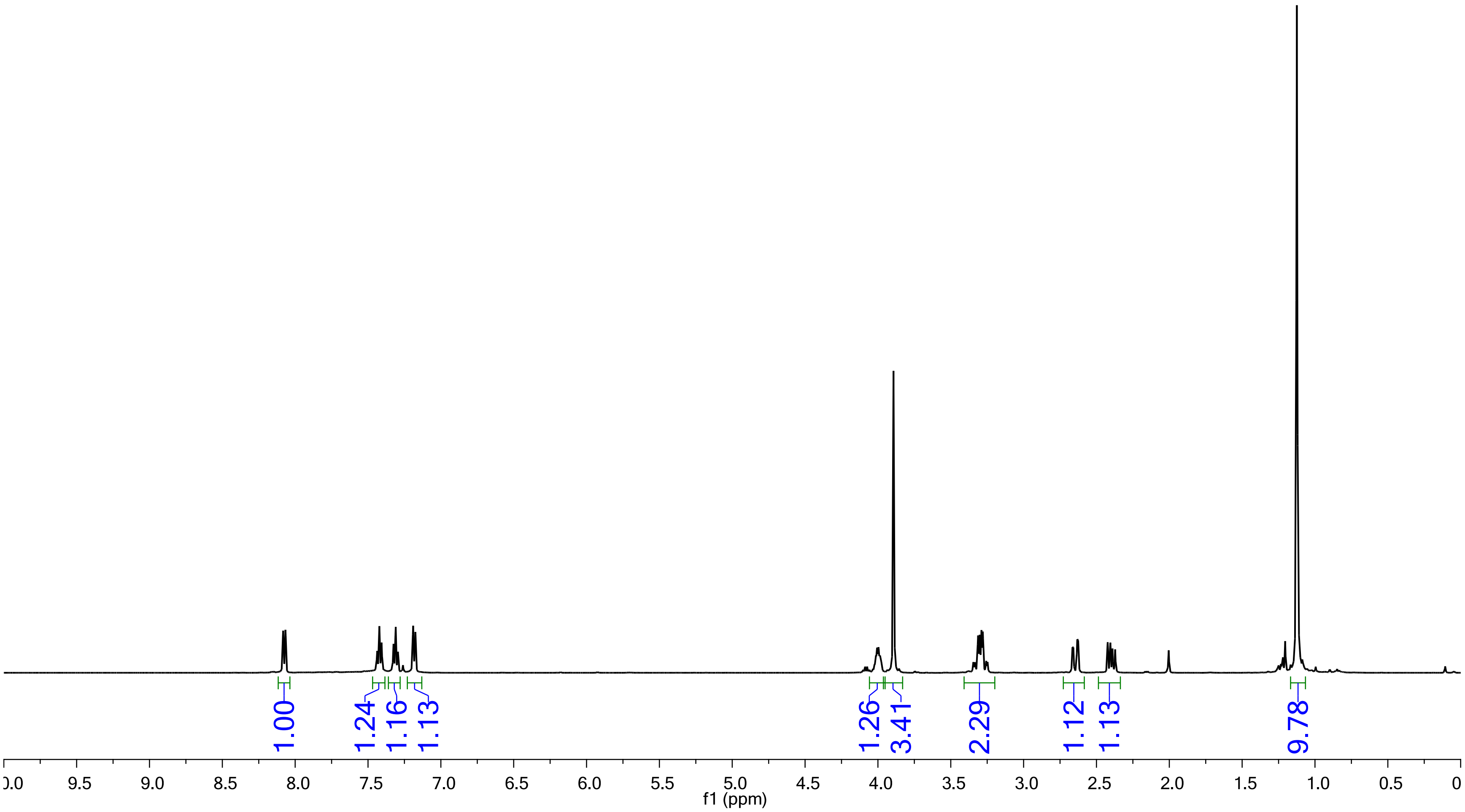
1.00
1.24
1.16
1.13
1.26
3.41
2.29
1.12
1.13
9.78
9.5
9.0
8.5
8.0
7.5
7.0
6.5
6.0
5.5
5.0
4.5
4.0
3.5
3.0
2.5
2.0
1.5
1.0
0.5
0
f1 (ppm)

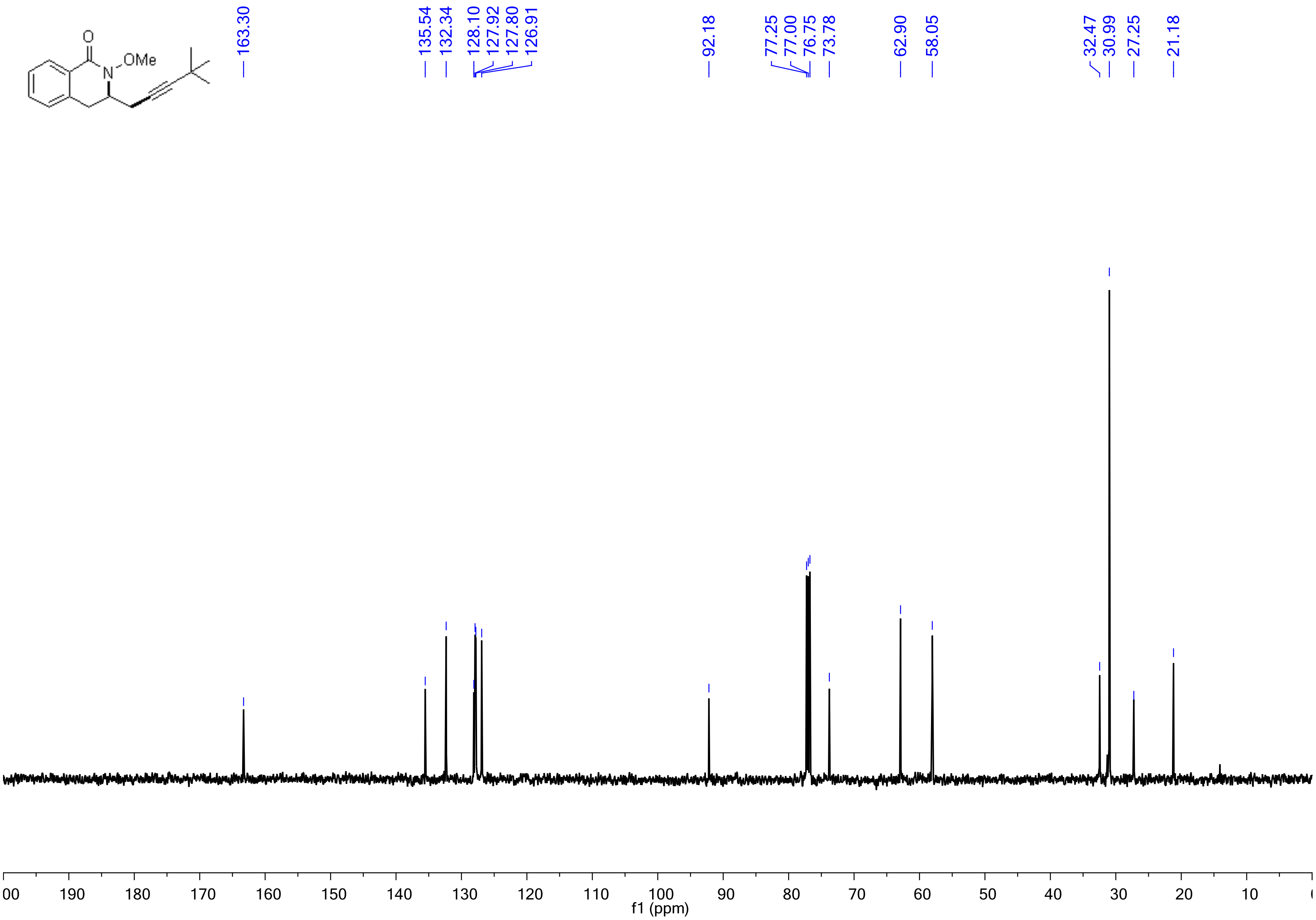
O
N
OMe
163.30
135.54
132.34
128.10
127.92
127.80
126.91
92.18
77.25
77.00
76.75
73.78
62.90
58.05
32.47
30.99
27.25
21.18
190 180 170 160 150 140 130 120 110 100 90 80 70 60 50 40 30 20 10
f1 (ppm)

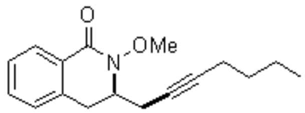
O
N
OMe

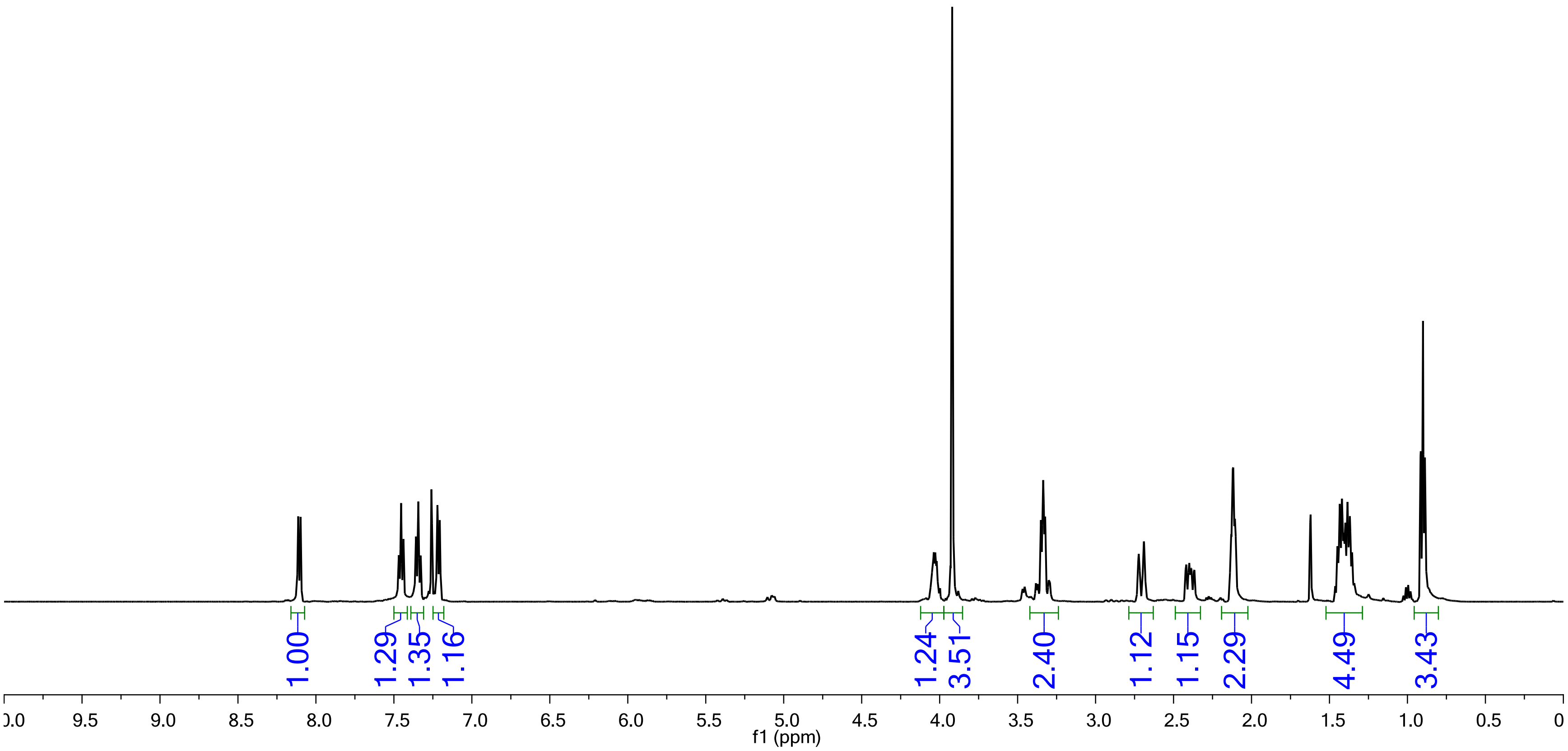
1.00
1.29
1.35
1.16
1.24
3.51
2.40
1.12
1.15
2.29
4.49
3.43
9.5
9.0
8.5
8.0
7.5
7.0
6.5
6.0
5.5
5.0
4.5
4.0
3.5
3.0
2.5
2.0
1.5
1.0
0.5
f1 (ppm)

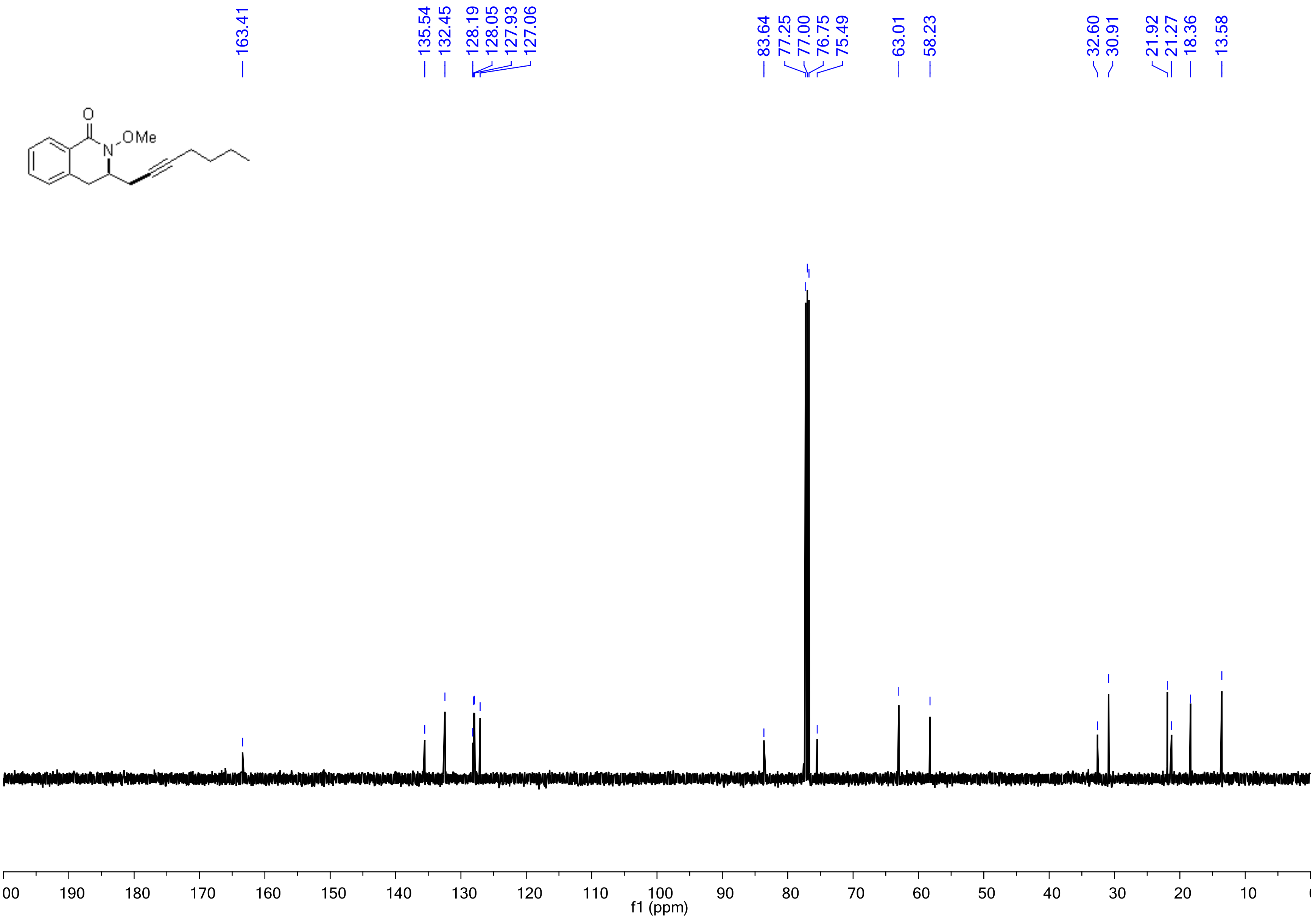
163.41
135.54
132.45
128.19
128.05
127.93
127.06
83.64
77.25
77.00
76.75
75.49
63.01
58.23
32.60
30.91
21.92
21.27
18.36
13.58
O
N
OMe
00
190
180
170
160
150
140
130
120
110
100
90
80
70
60
50
40
30
20
10
f1 (ppm)

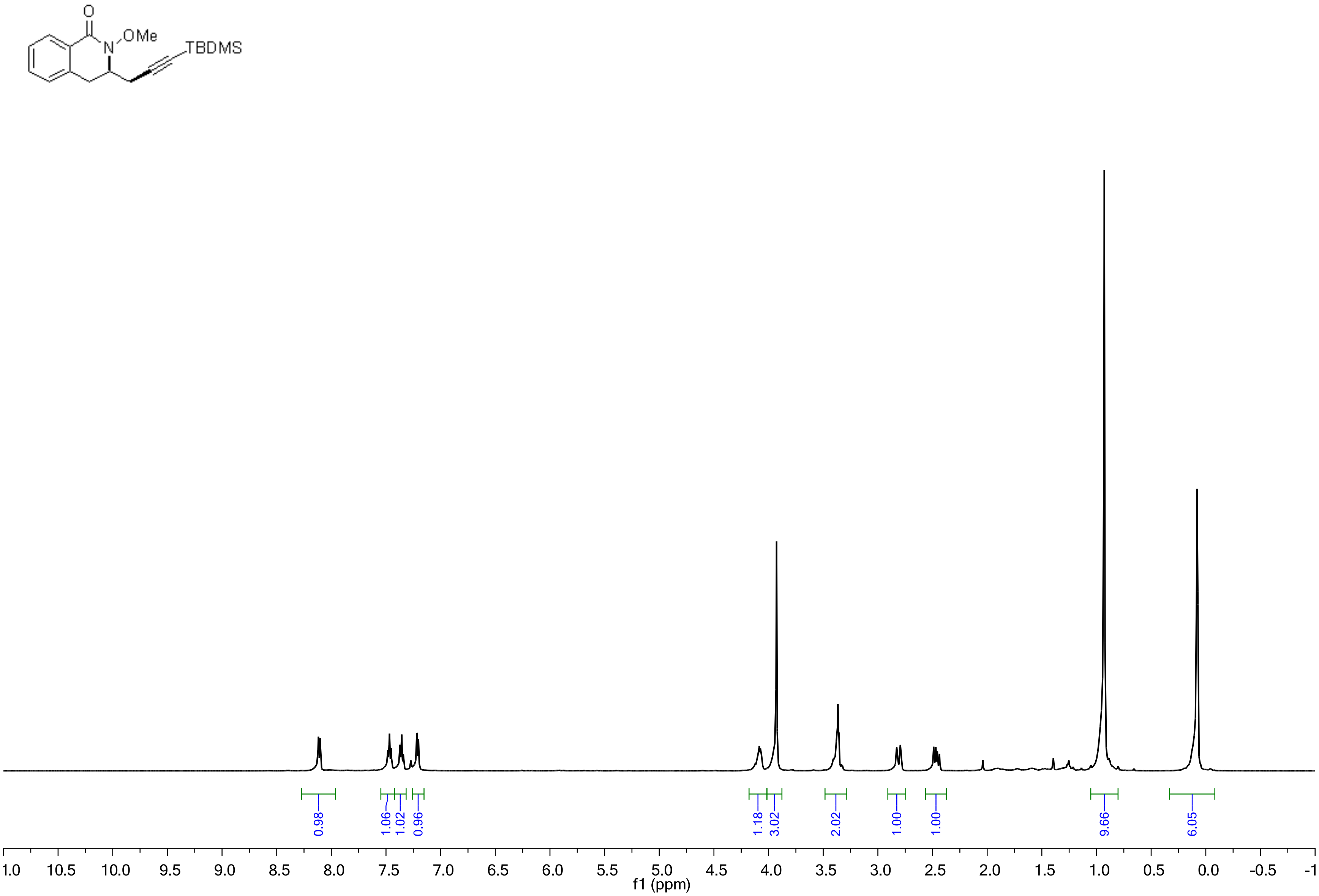

O
N
OMe
TBDMS
0.98
1.06
1.02
0.96
1.18
3.02
2.02
1.00
1.00
9.66
6.05
1.0
10.5
10.0
9.5
9.0
8.5
8.0
7.5
7.0
6.5
6.0
5.5
5.0
4.5
4.0
3.5
3.0
2.5
2.0
1.5
1.0
0.5
0.0
-0.5
-1
f1 (ppm)

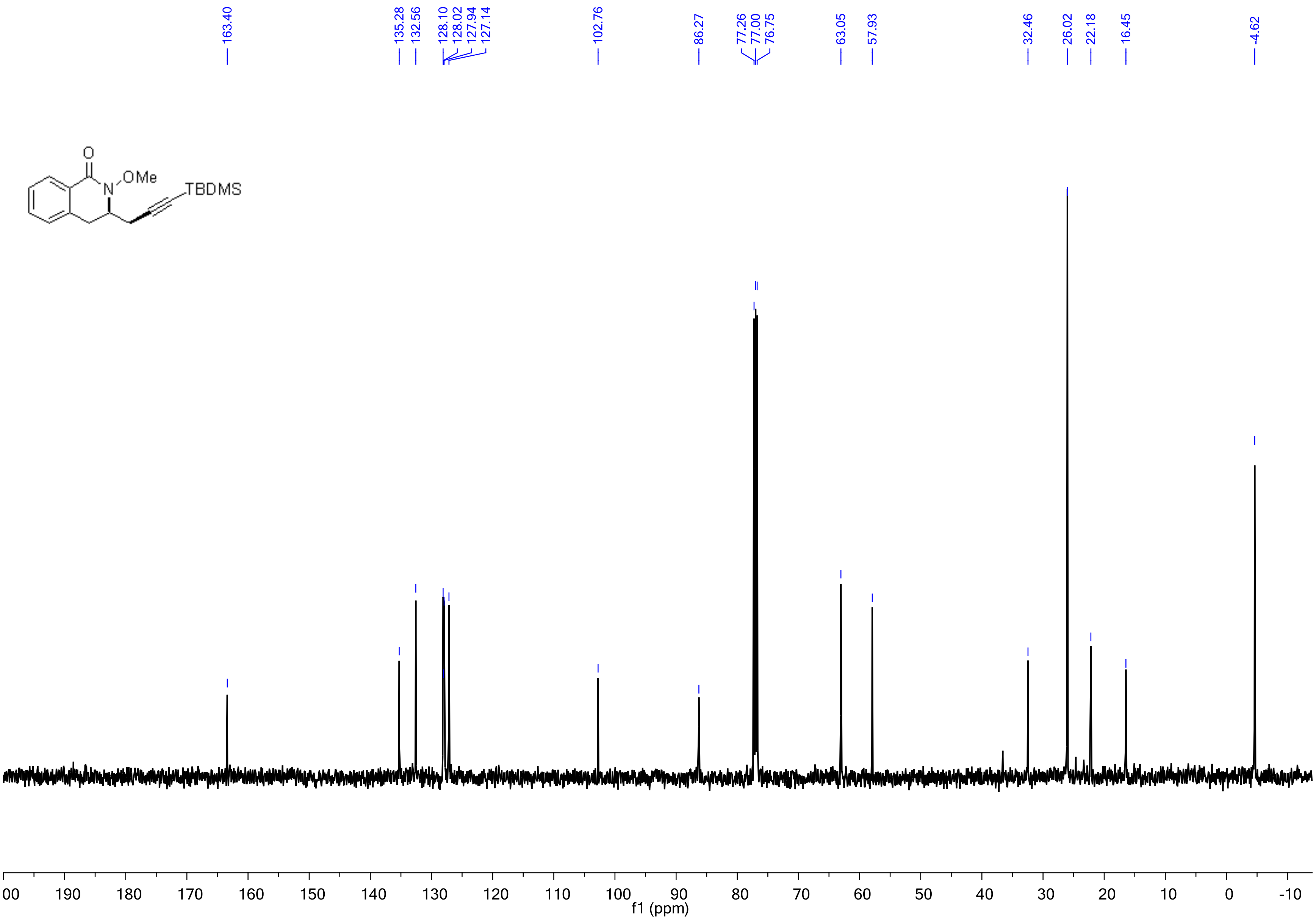

163.40
135.28
132.56
128.10
128.02
127.94
127.14
102.76
86.27
77.26
77.00
76.75
63.05
57.93
32.46
26.02
22.18
16.45
-4.62
O
N
OMe
TBDMS
00
190
180
170
160
150
140
130
120
110
100
90
80
70
60
50
40
30
20
10
0
-10
f1 (ppm)

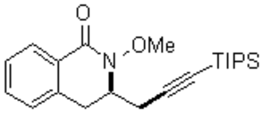
O
N
OMe
TIPS

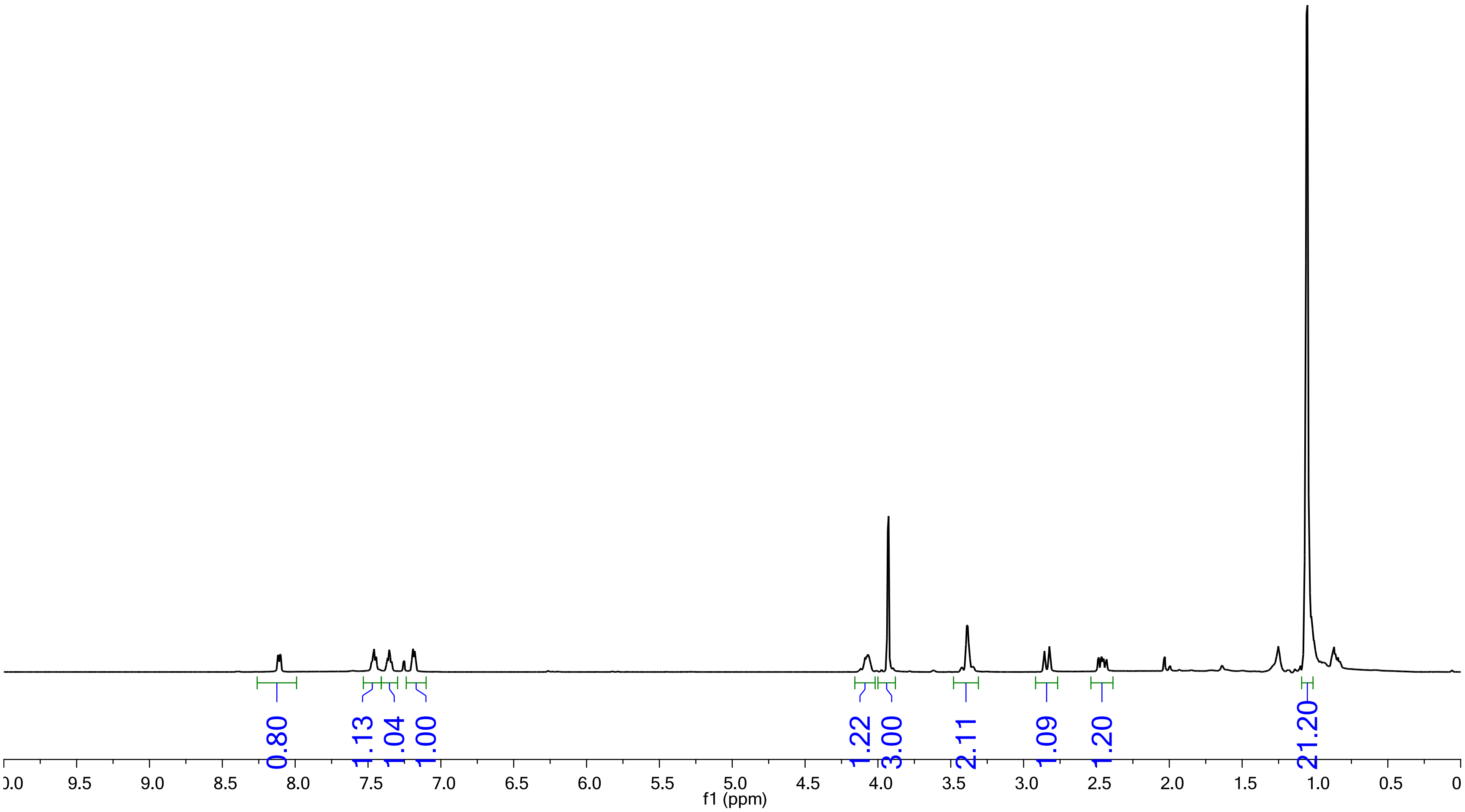
0.80
1.13
1.04
1.00
1.22
3.00
2.11
1.09
1.20
21.20
10.0
9.5
9.0
8.5
8.0
7.5
7.0
6.5
6.0
5.5
5.0
4.5
4.0
3.5
3.0
2.5
2.0
1.5
1.0
0.5
0
f1 (ppm)

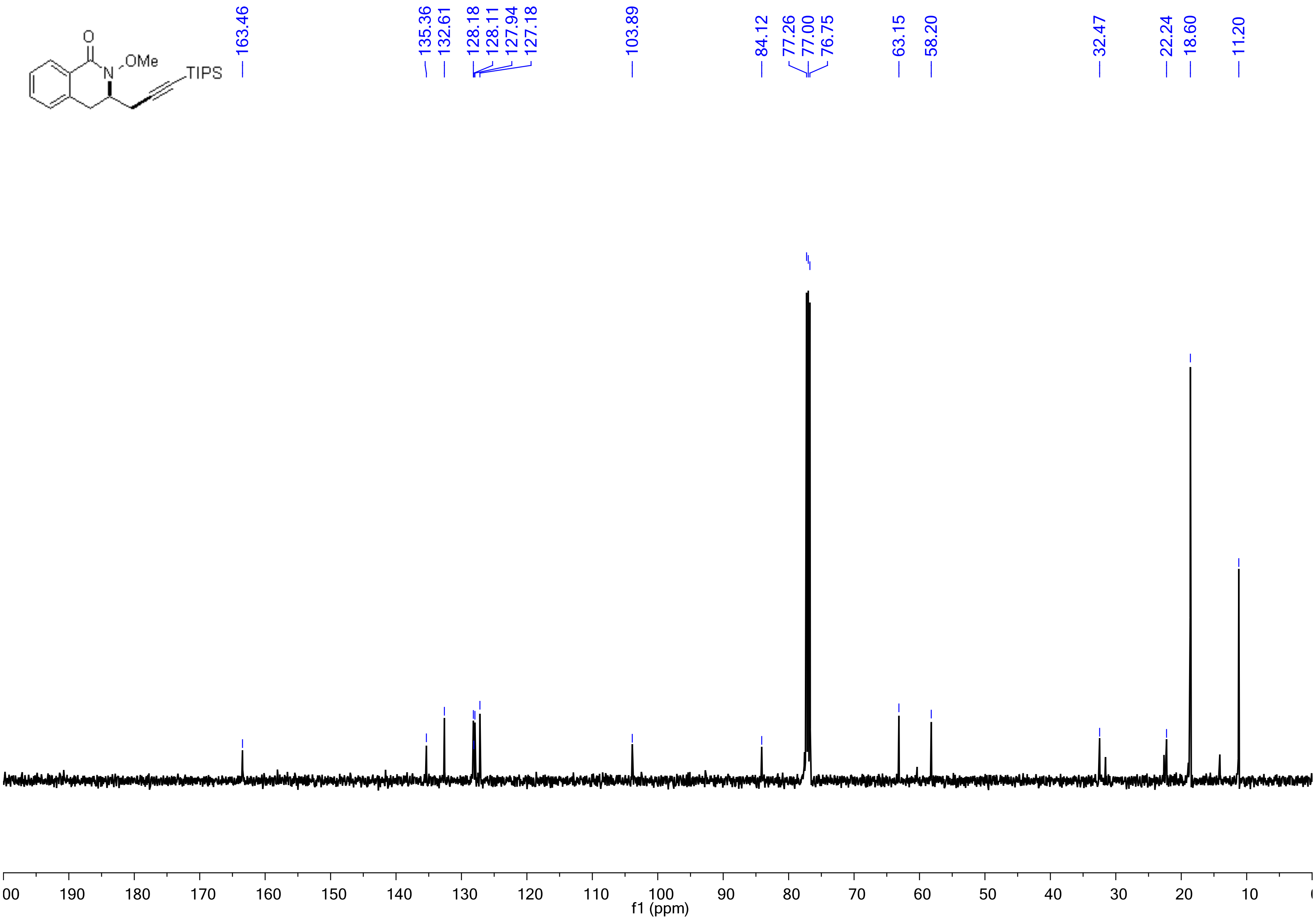
O
N
OMe
TIPS
163.46
135.36
132.61
128.18
128.11
127.94
127.18
103.89
84.12
77.26
77.00
76.75
63.15
58.20
32.47
22.24
18.60
11.20
190
180
170
160
150
140
130
120
110
100
90
80
70
60
50
40
30
20
10
f1 (ppm)

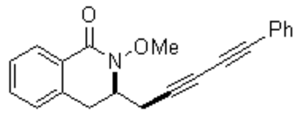
O
N
OMe
Ph

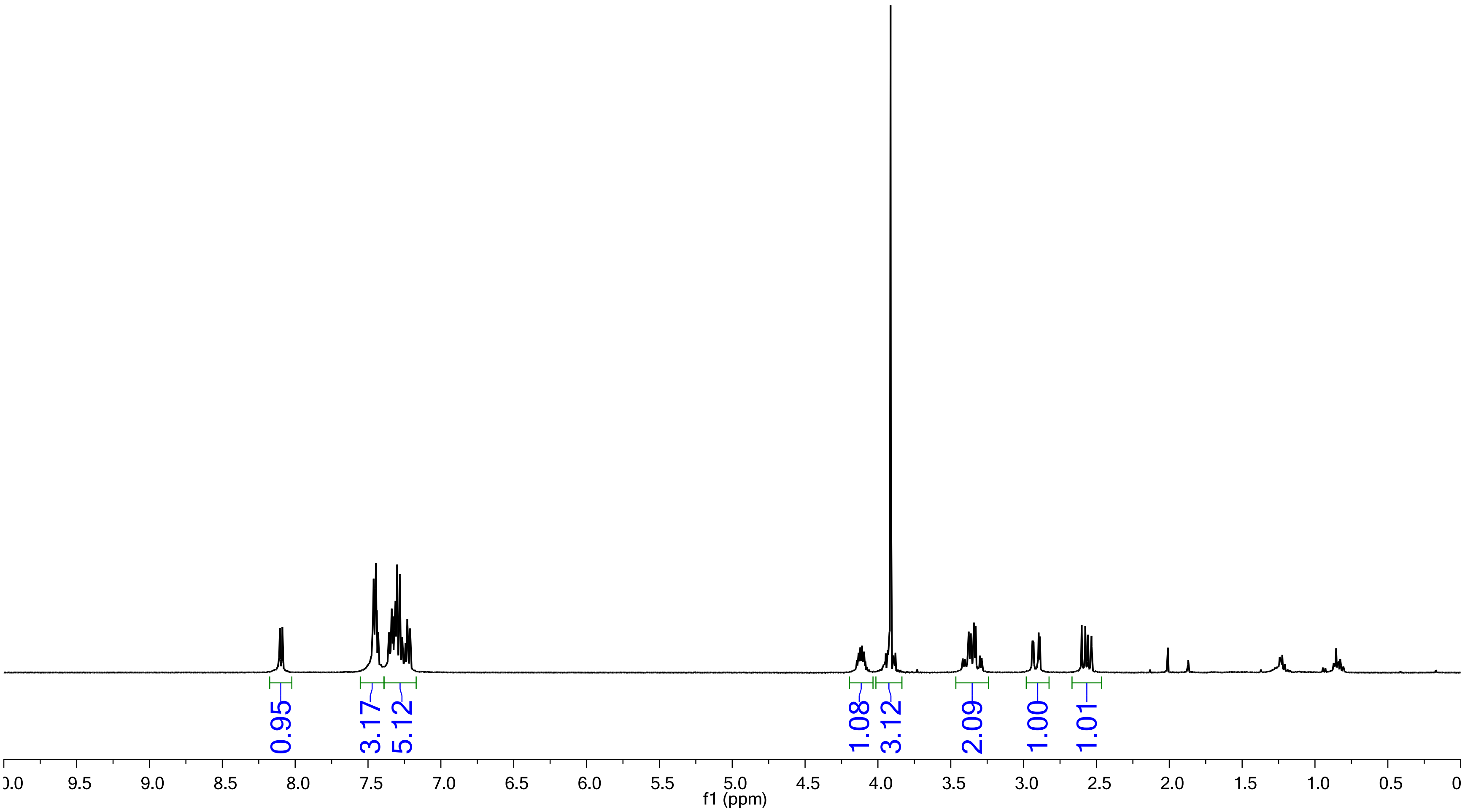
0.95
3.17
5.12
1.08
3.12
2.09
1.00
1.01
9.5
9.0
8.5
8.0
7.5
7.0
6.5
6.0
5.5
5.0
4.5
4.0
3.5
3.0
2.5
2.0
1.5
1.0
0.5
0
f1 (ppm)

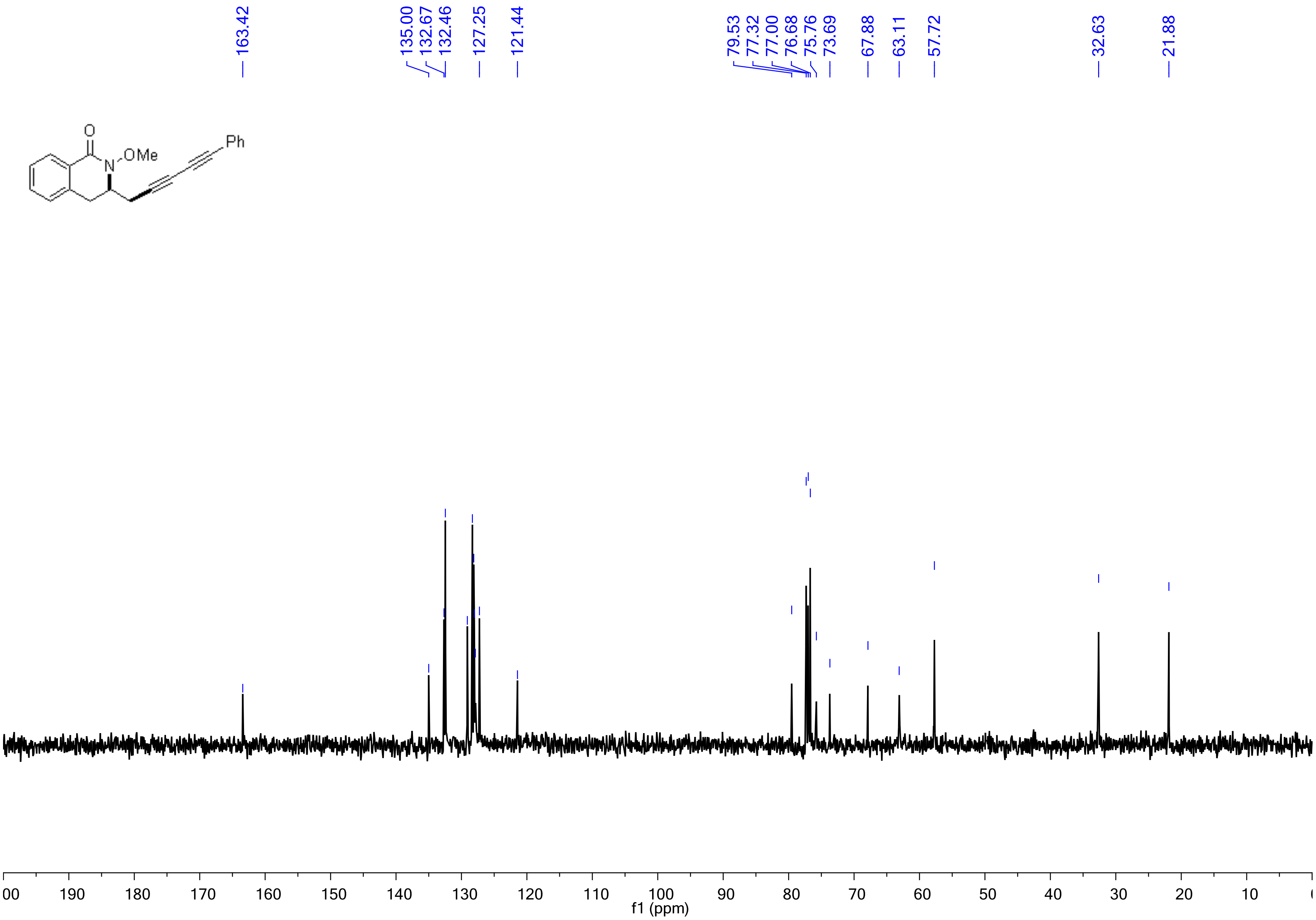

163.42
135.00
132.67
132.46
127.25
121.44
79.53
77.32
77.00
76.68
75.76
73.69
67.88
63.11
57.72
32.63
21.88
O
N
OMe
Ph
00
190
180
170
160
150
140
130
120
110
100
90
80
70
60
50
40
30
20
10
f1 (ppm)

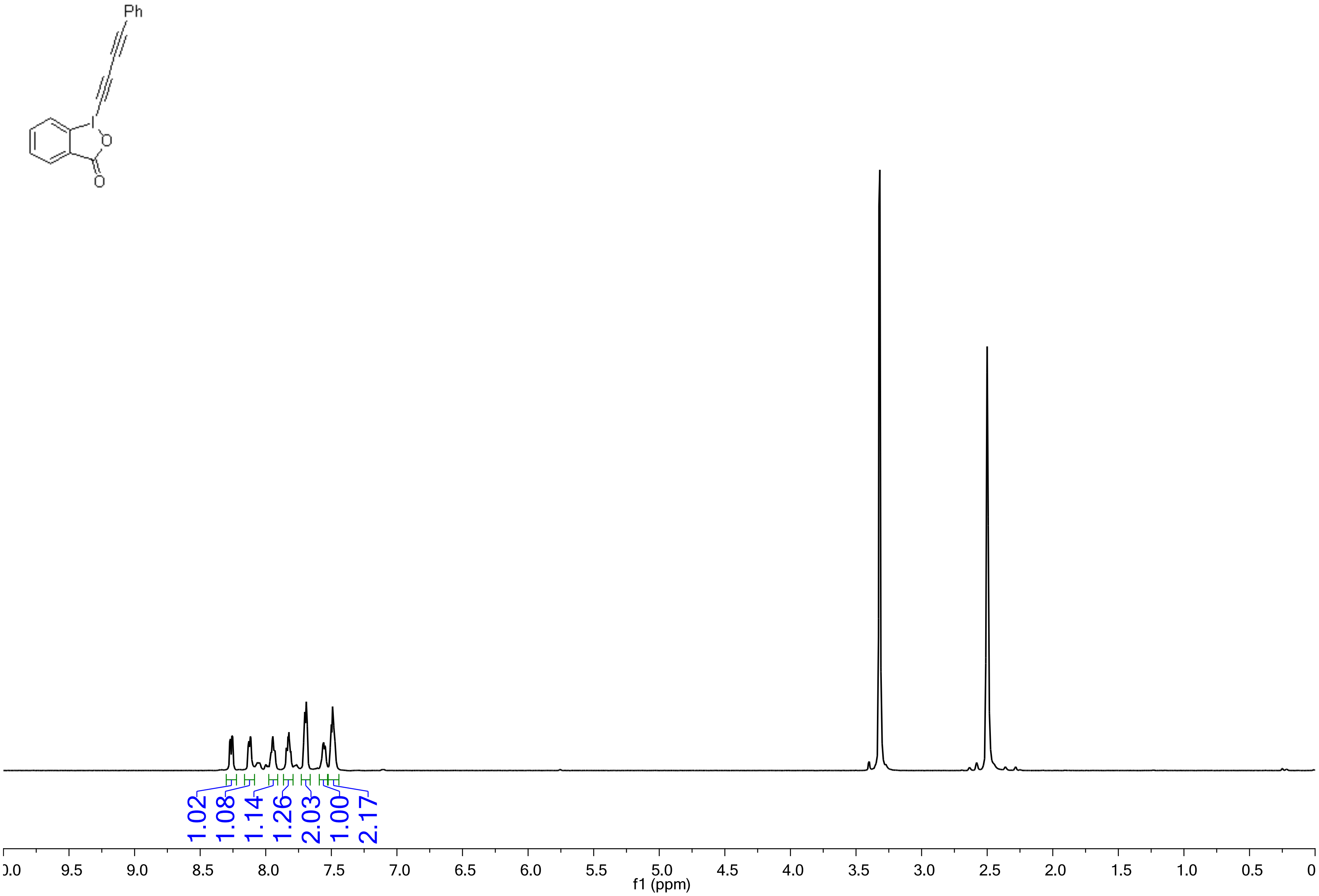
Ph
I
O
O
1.02
1.08
1.14
1.26
2.03
1.00
2.17
10.0
9.5
9.0
8.5
8.0
7.5
7.0
6.5
6.0
5.5
5.0
4.5
4.0
3.5
3.0
2.5
2.0
1.5
1.0
0.5
0
f1 (ppm)

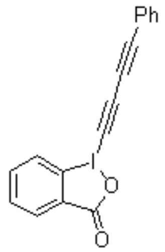
Ph
I
O
O

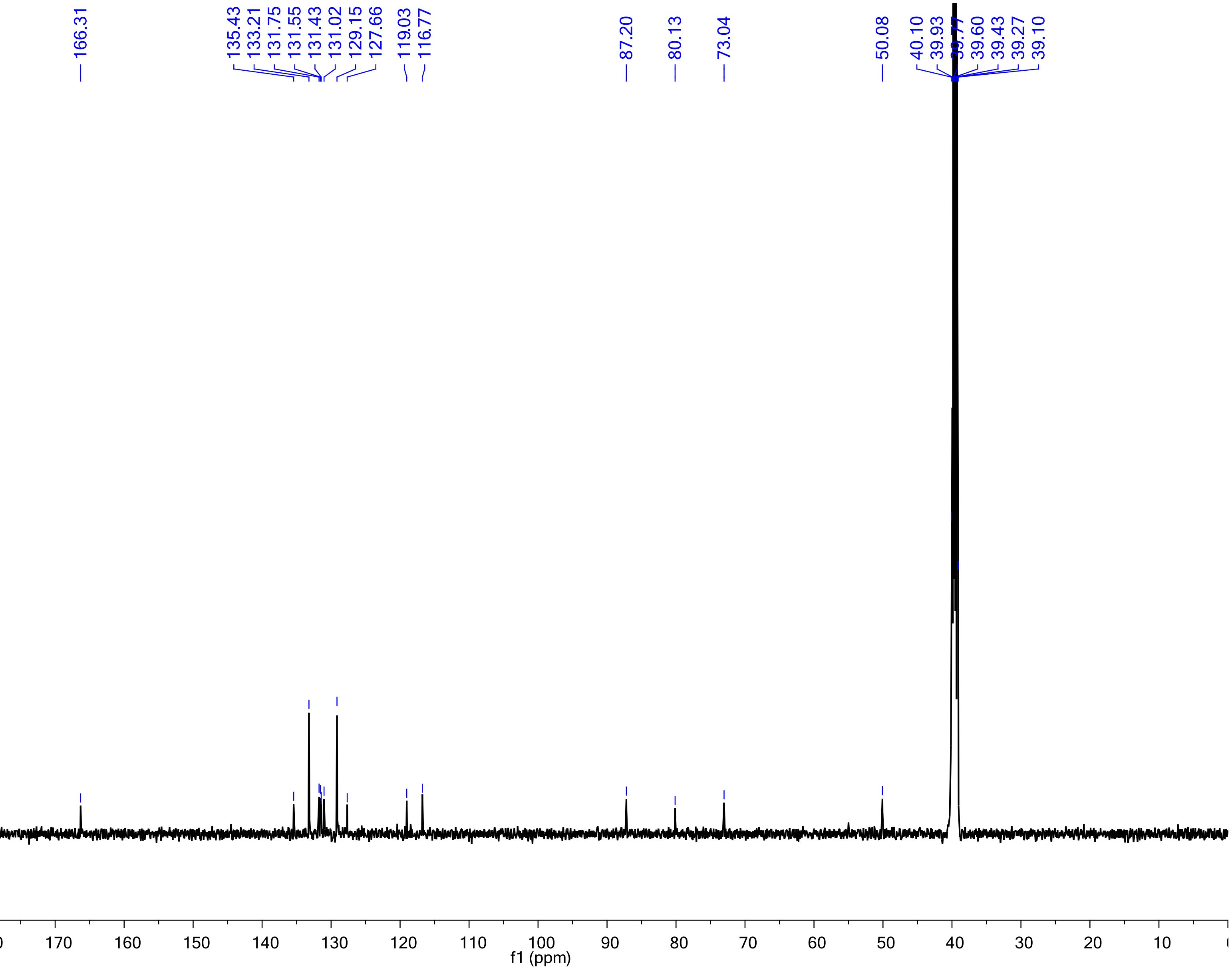
166.31
135.43
133.21
131.75
131.55
131.43
131.02
129.15
127.66
119.03
116.77
87.20
80.13
73.04
50.08
40.10
39.93
39.77
39.60
39.43
39.27
39.10
00
190
180
170
160
150
140
130
120
110
100
90
80
70
60
50
40
30
20
10
f1 (ppm)

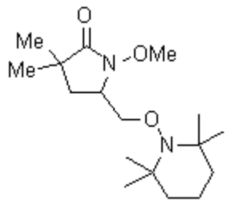
O
Me
Me
N
OMe
O
N

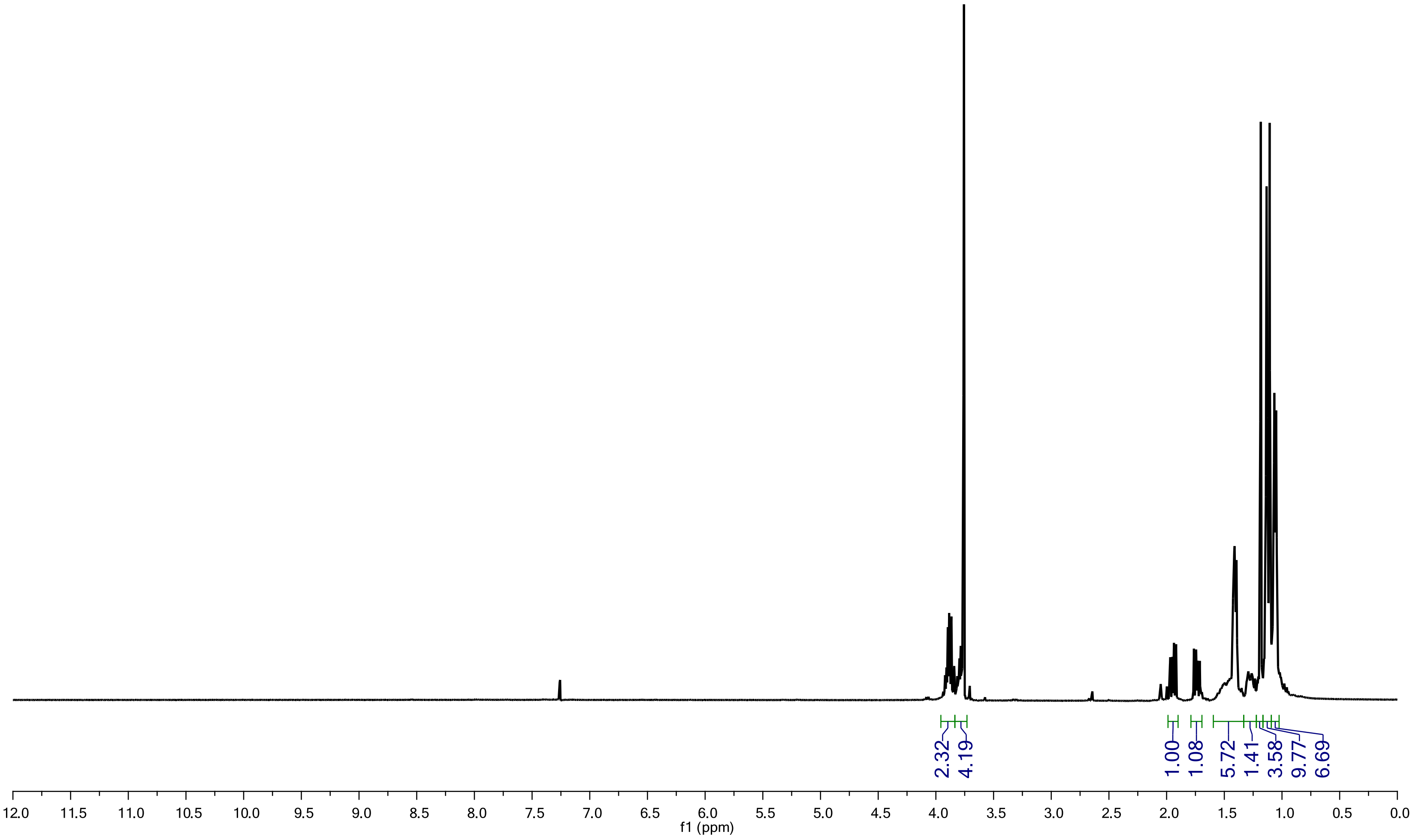
2.32
4.19
1.00
1.08
5.72
1.41
3.58
9.77
6.69
12.0 11.5 11.0 10.5 10.0 9.5 9.0 8.5 8.0 7.5 7.0 6.5 6.0 5.5 5.0 4.5 4.0 3.5 3.0 2.5 2.0 1.5 1.0 0.5 0.0
f1 (ppm)

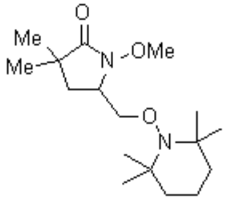

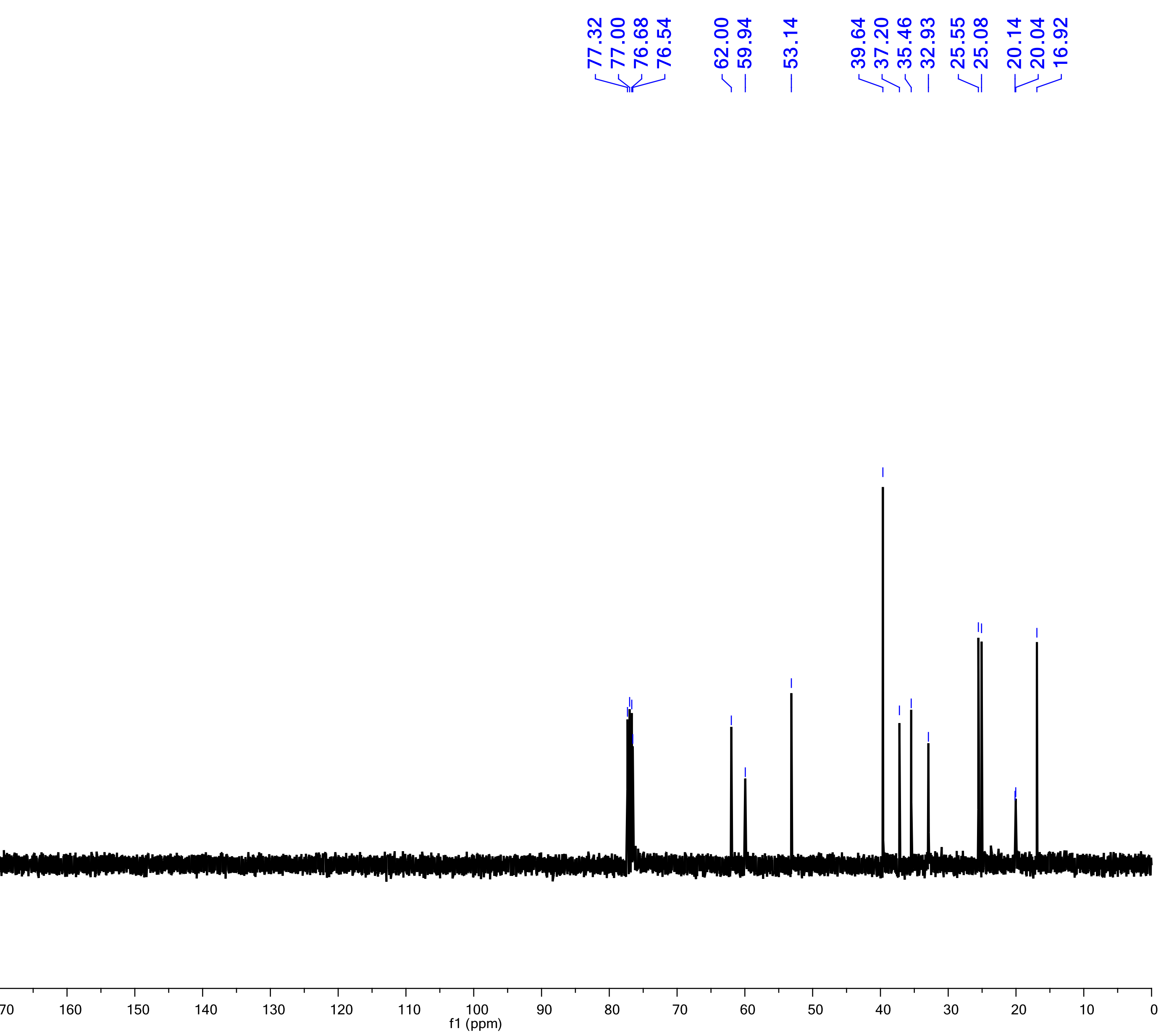
175.70
77.32
77.00
76.68
76.54
62.00
59.94
53.14
39.64
37.20
35.46
32.93
25.55
25.08
20.14
20.04
16.92
200 190 180 170 160 150 140 130 120 110 100 90 80 70 60 50 40 30 20 10 0
f1 (ppm)

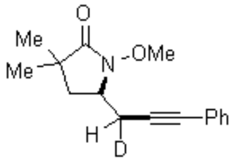

O
Me
Me
N
OMe
H
D
Ph

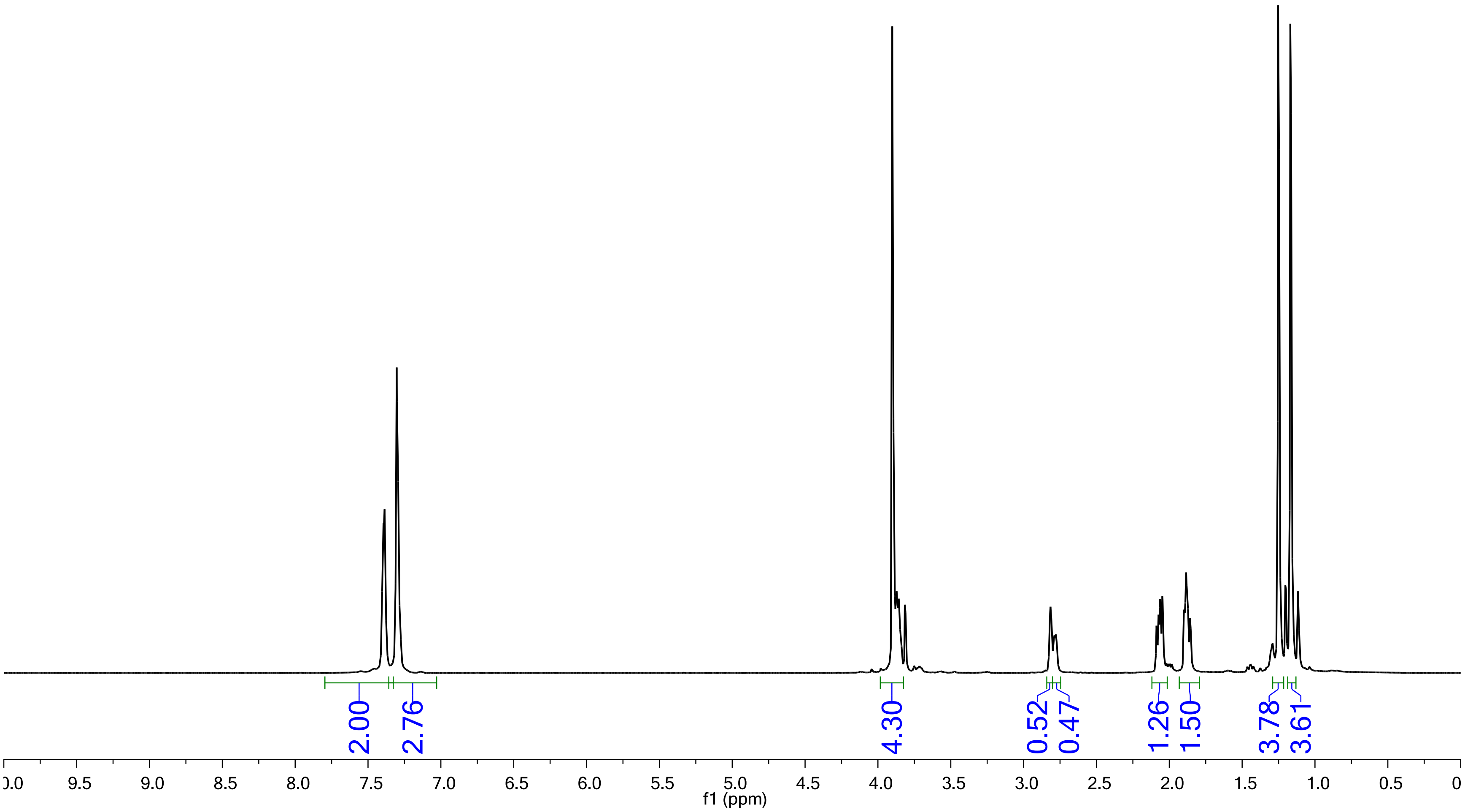

2.00
2.76
4.30
0.52
0.47
1.26
1.50
3.78
3.61
f1 (ppm)

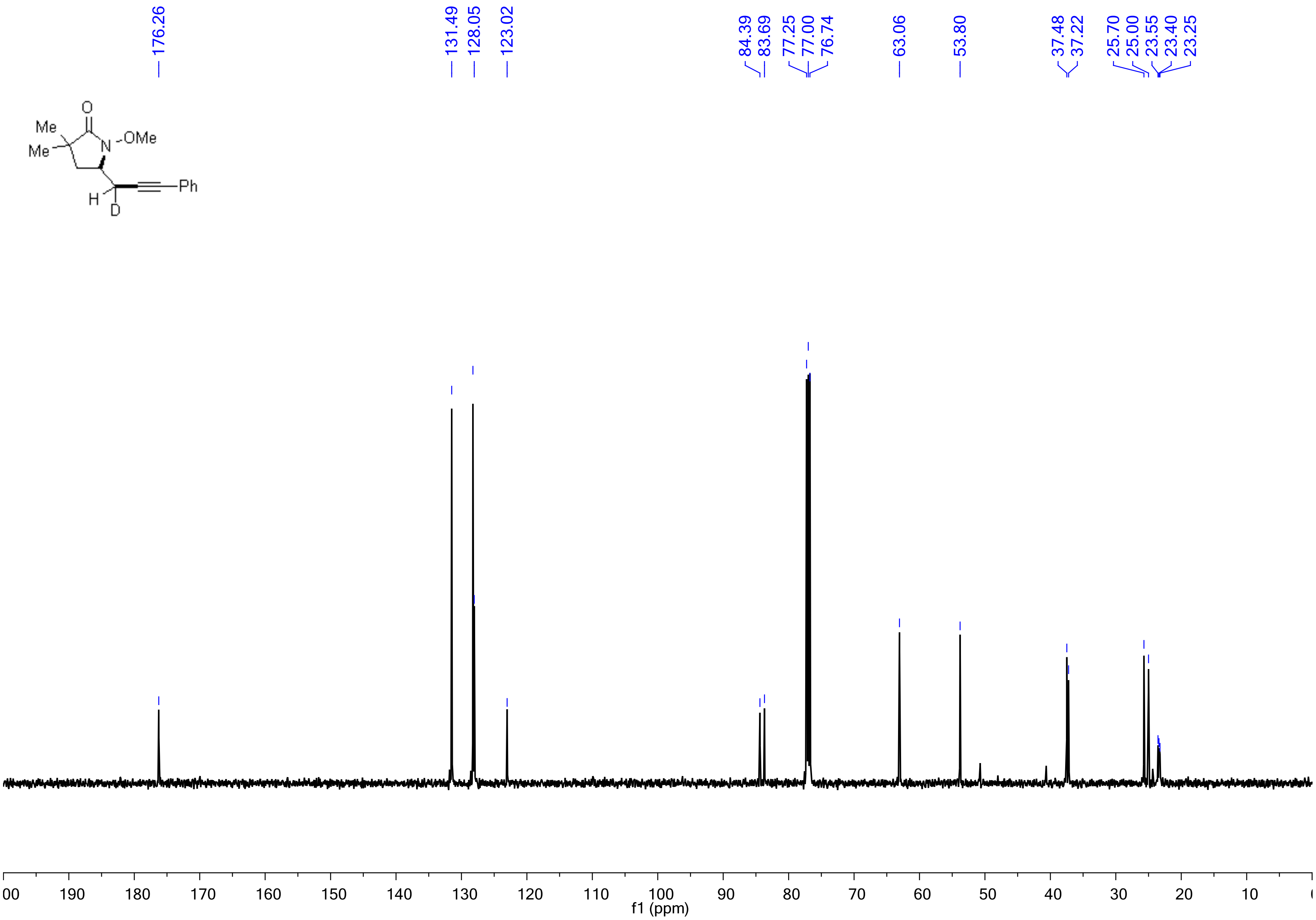

176.26
131.49
128.05
123.02
84.39
83.69
77.25
77.00
76.74
63.06
53.80
37.48
37.22
25.70
25.00
23.55
23.40
23.25
O
Me
Me
N
OMe
H
D
Ph
f1 (ppm)

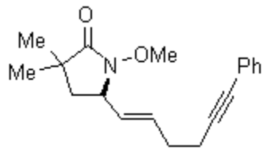
Me
Me
O
N
OMe
Ph

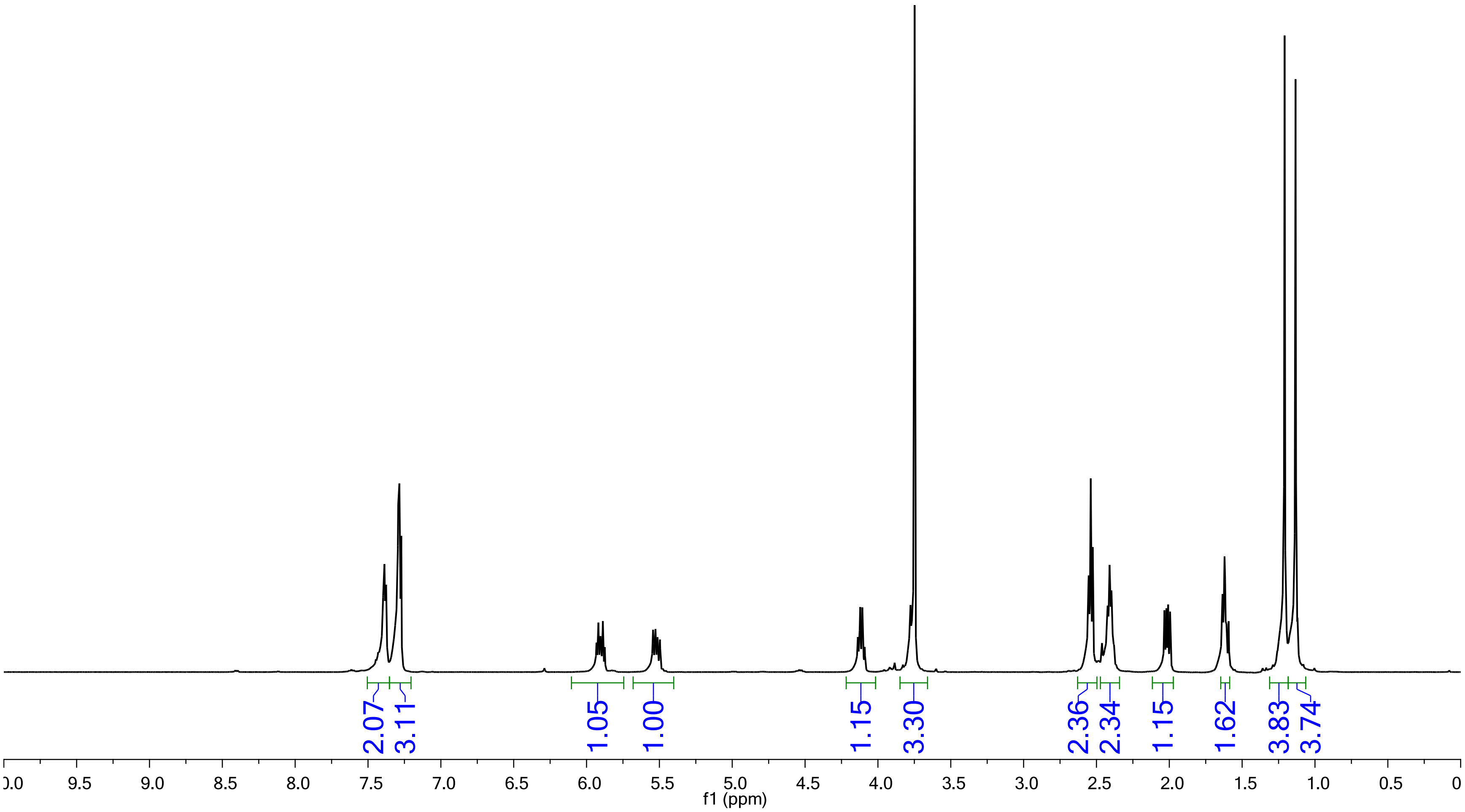
2.07
3.11
1.05
1.00
1.15
3.30
2.36
2.34
1.15
1.62
3.83
3.74
9.5
9.0
8.5
8.0
7.5
7.0
6.5
6.0
5.5
5.0
4.5
4.0
3.5
3.0
2.5
2.0
1.5
1.0
0.5
0
f1 (ppm)

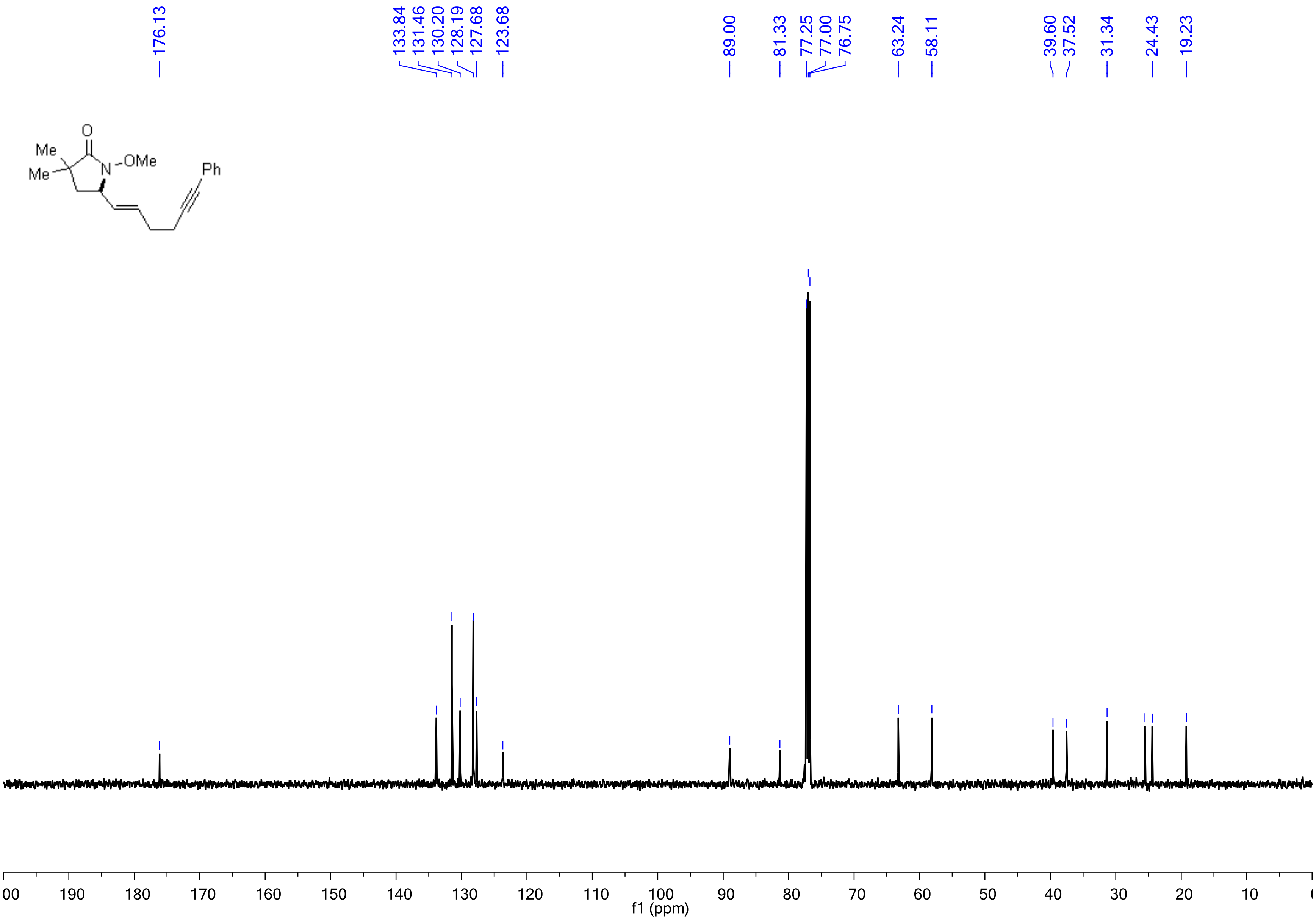

176.13
133.84
131.46
130.20
128.19
127.68
123.68
89.00
81.33
77.25
77.00
76.75
63.24
58.11
39.60
37.52
31.34
24.43
19.23
Me
Me
O
N
OMe
Ph
f1 (ppm)

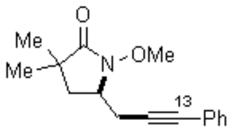

O
Me
Me
N
OMe
13
Ph

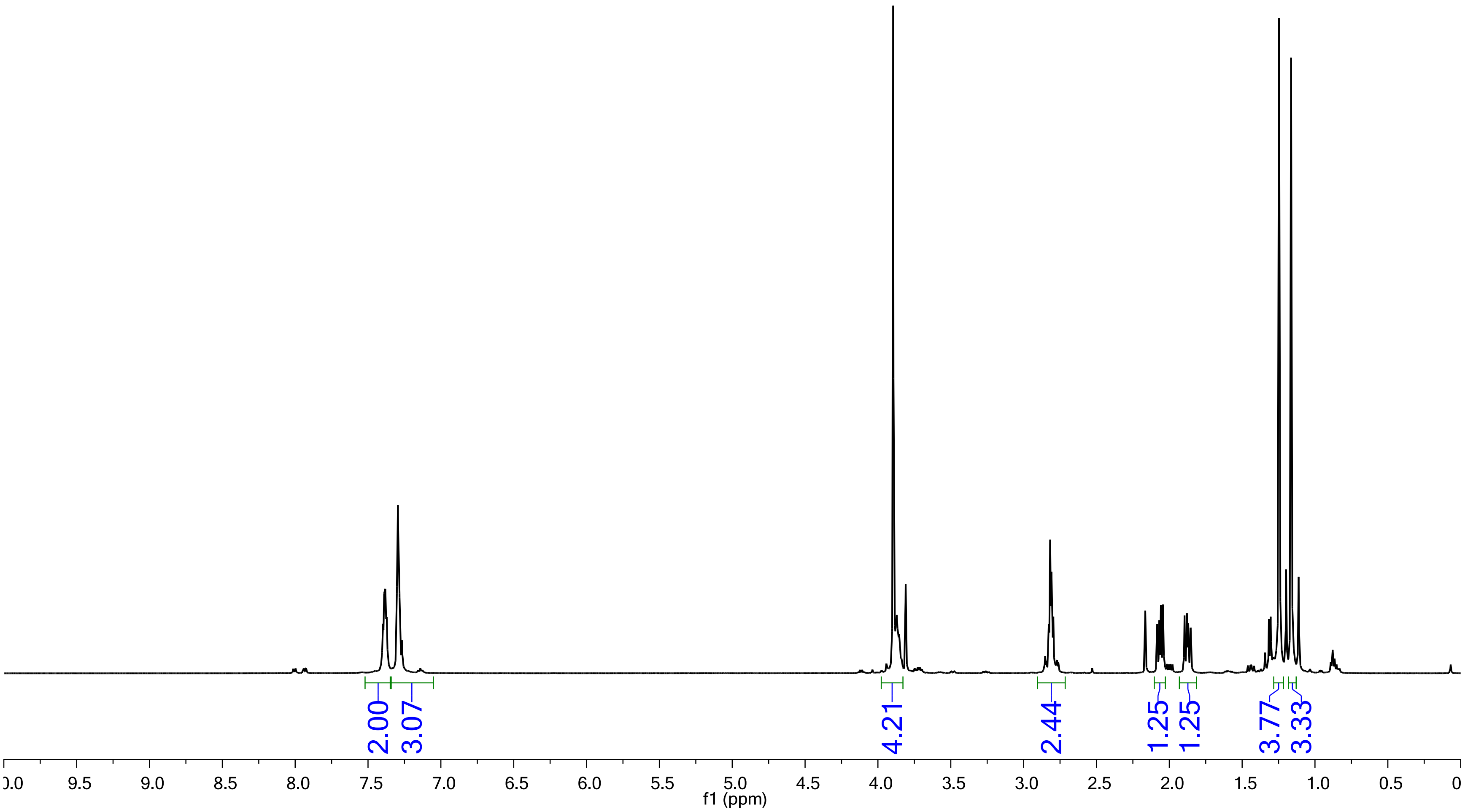

2.00
3.07
4.21
2.44
1.25
1.25
3.77
3.33
10.0
9.5
9.0
8.5
8.0
7.5
7.0
6.5
6.0
5.5
5.0
4.5
4.0
3.5
3.0
2.5
2.0
1.5
1.0
0.5
0
f1 (ppm)

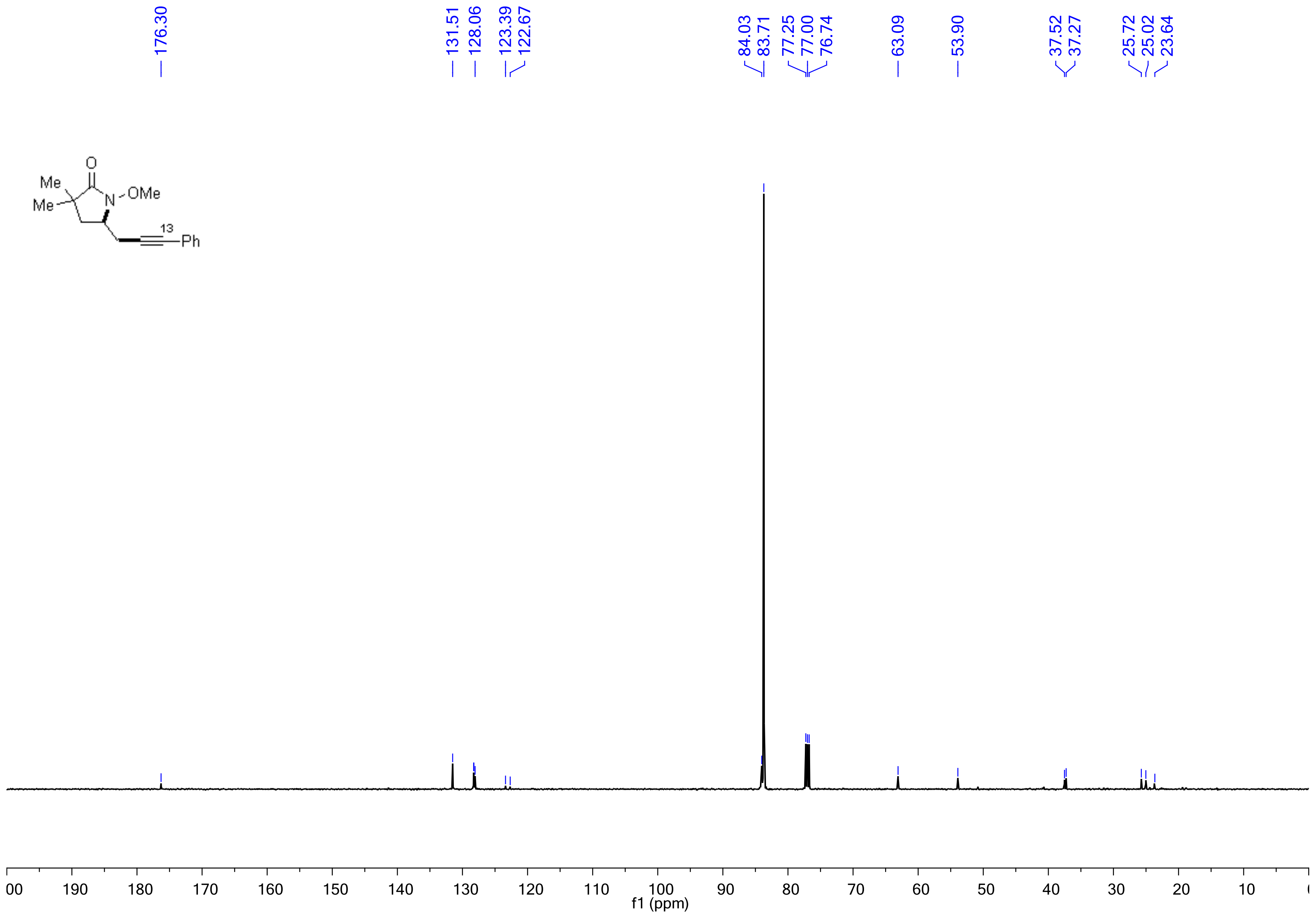

176.30
131.51
128.06
123.39
122.67
84.03
83.71
77.25
77.00
76.74
63.09
53.90
37.52
37.27
25.72
25.02
23.64
O
Me
Me
N
OMe
13
Ph
190
180
170
160
150
140
130
120
110
100
90
80
70
60
50
40
30
20
10
f1 (ppm)

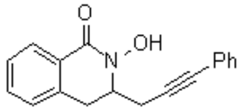

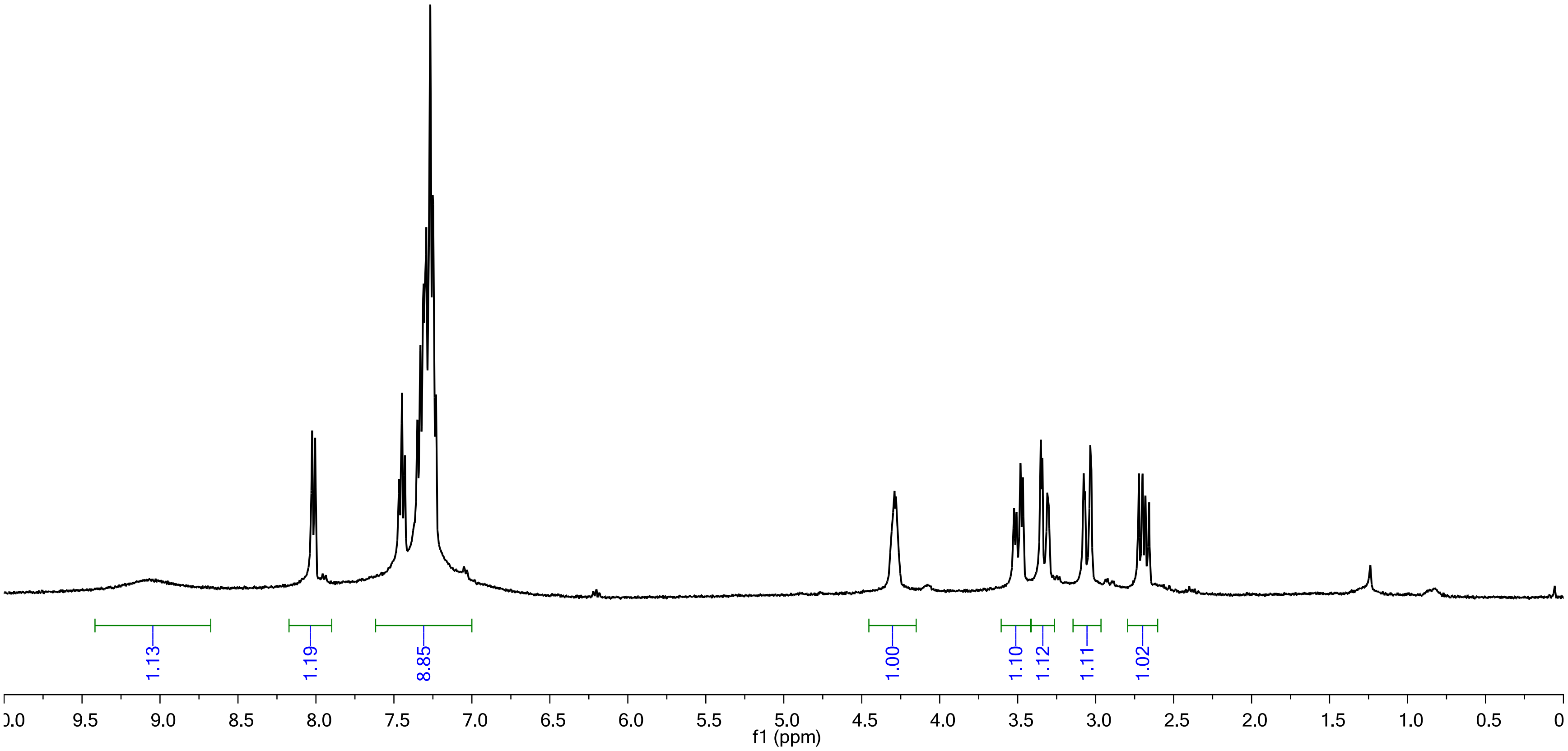
1.13
1.19
8.85
1.00
1.10
1.12
1.11
1.02
10.0
9.5
9.0
8.5
8.0
7.5
7.0
6.5
6.0
5.5
5.0
4.5
4.0
3.5
3.0
2.5
2.0
1.5
1.0
0.5
0
f1 (ppm)

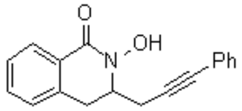
O
OH
N
Ph

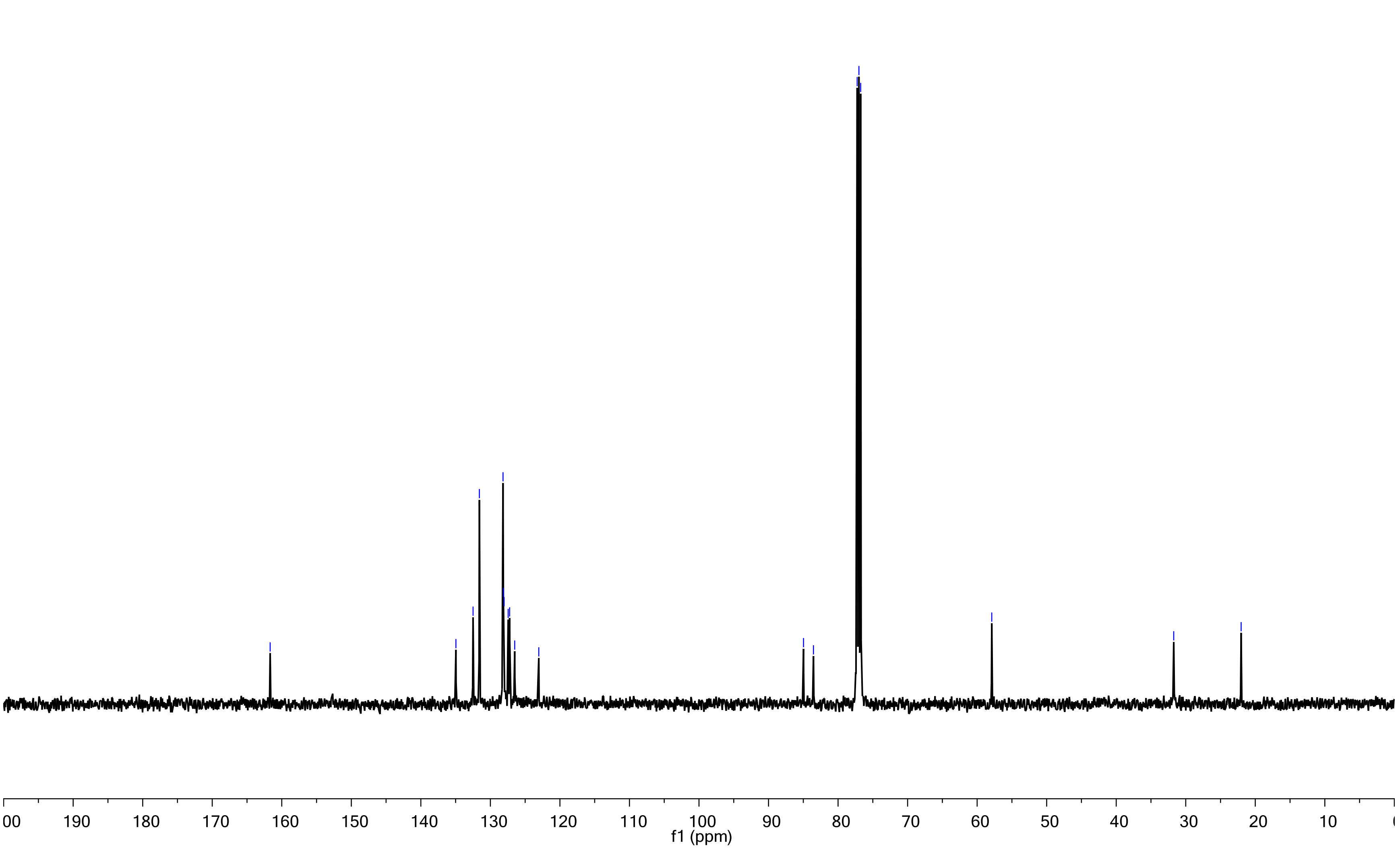
161.669
134.955
132.490
131.582
126.500
123.038
84.968
83.539
77.254
77.000
76.746
57.895
31.727
22.035
f1 (ppm)

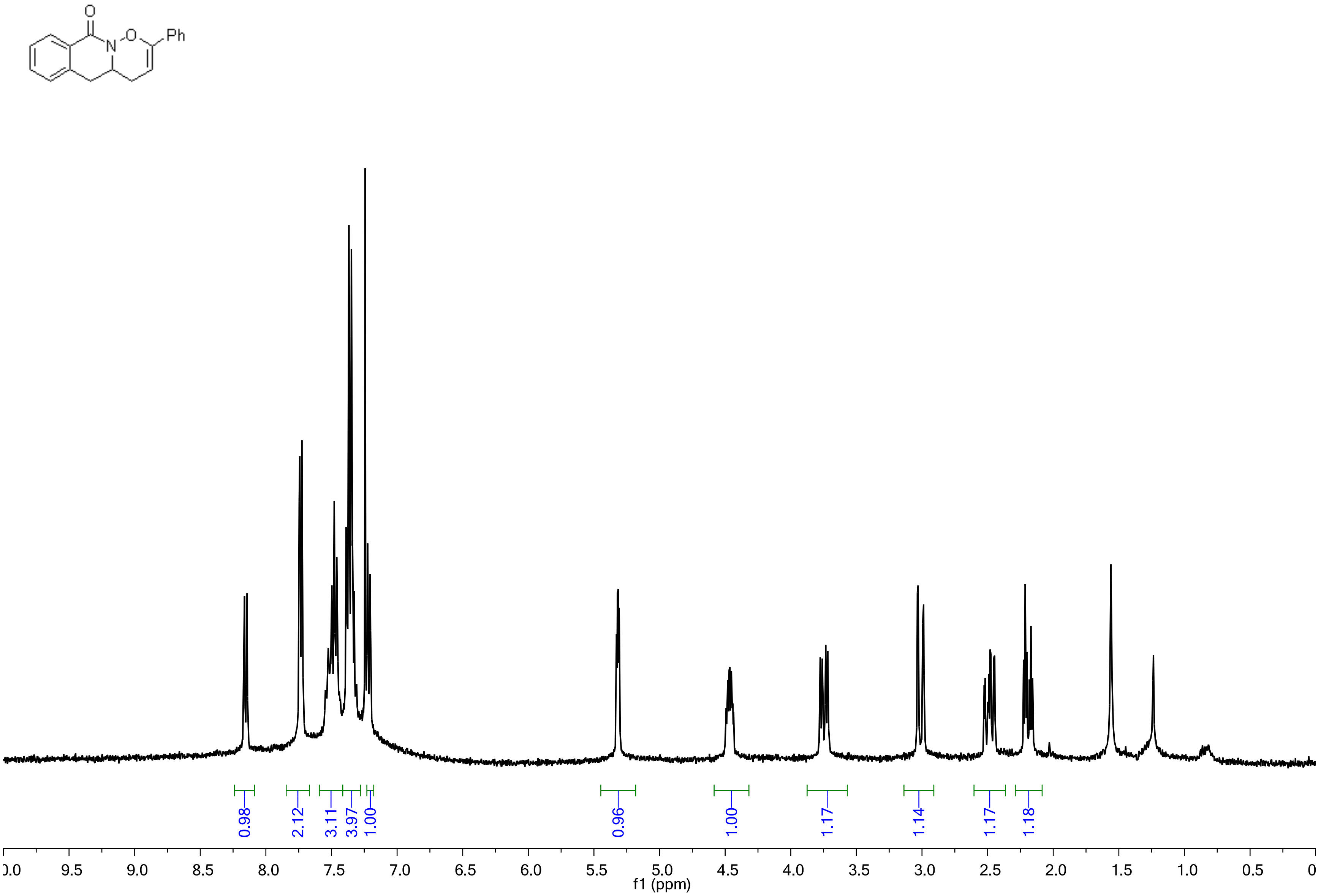
O
N
O
Ph
0.98
2.12
3.11
3.97
1.00
0.96
1.00
1.17
1.14
1.17
1.18
9.5 9.0 8.5 8.0 7.5 7.0 6.5 6.0 5.5 5.0 4.5 4.0 3.5 3.0 2.5 2.0 1.5 1.0 0.5 0
f1 (ppm)

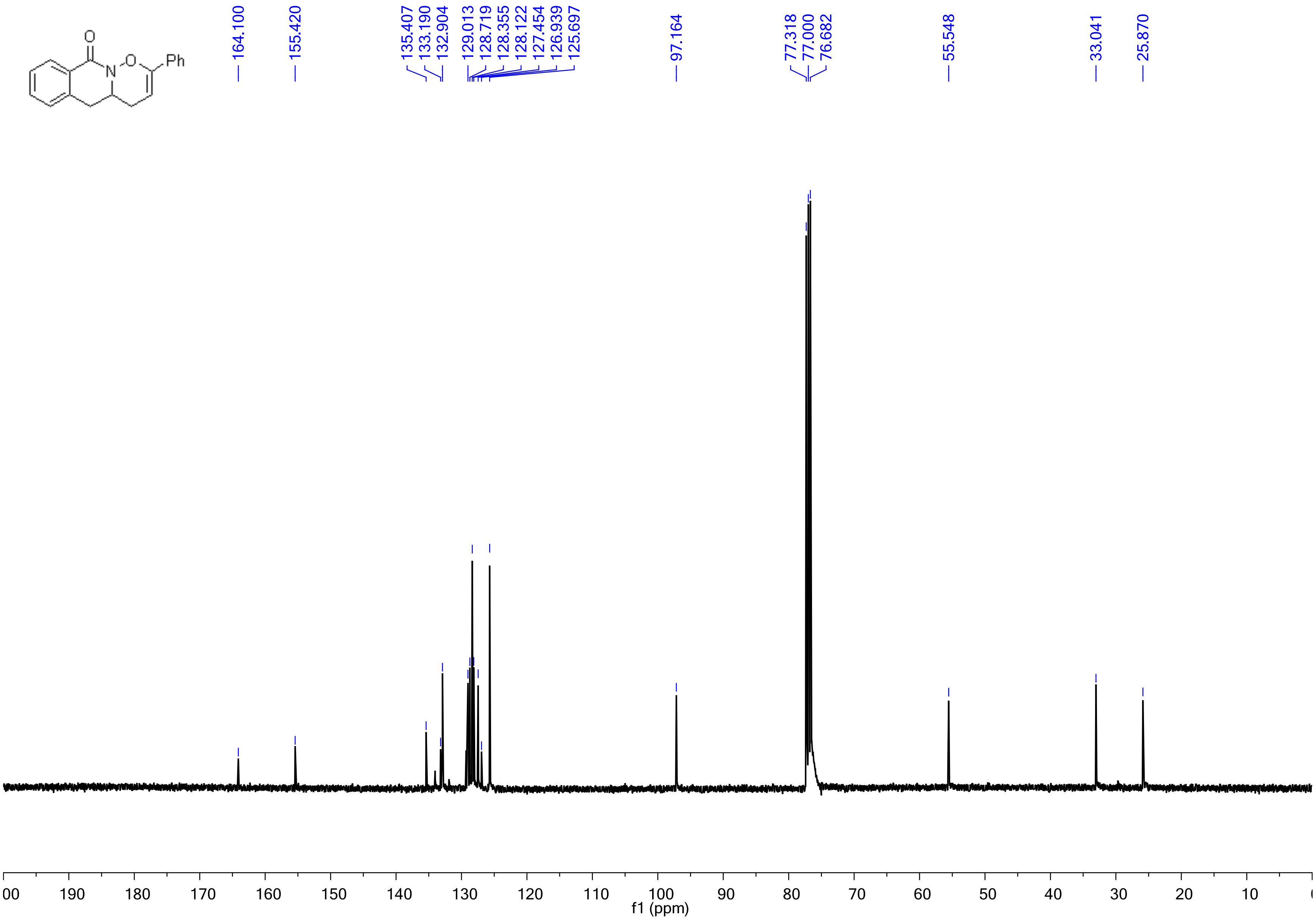
O
N
O
Ph
164.100
155.420
135.407
133.190
132.904
129.013
128.719
128.355
128.122
127.454
126.939
125.697
97.164
77.318
77.000
76.682
55.548
33.041
25.870
00
190
180
170
160
150
140
130
120
110
100
90
80
70
60
50
40
30
20
10
f1 (ppm)

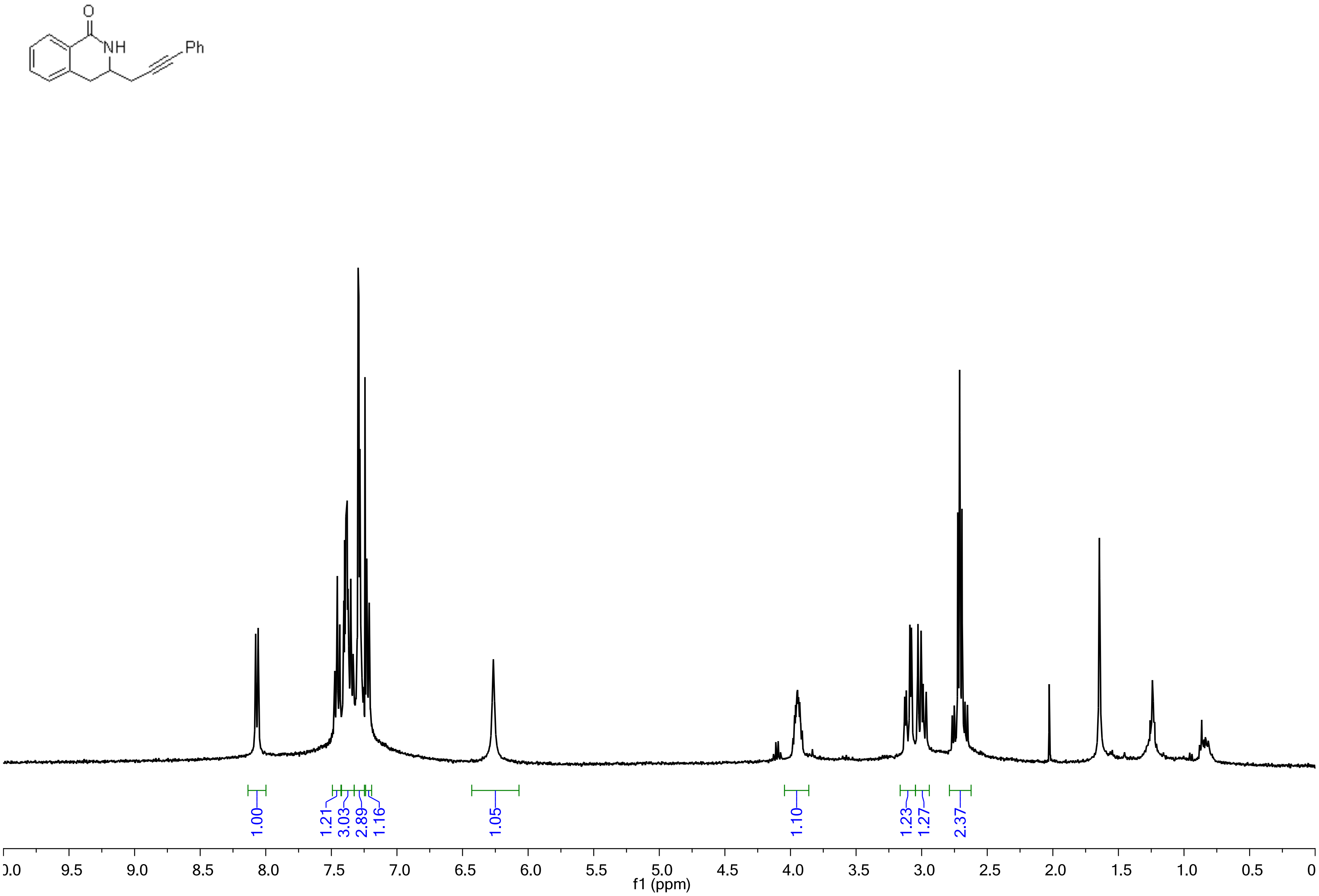
O
NH
Ph
1.00
1.21
3.03
2.89
1.16
1.05
1.10
1.23
1.27
2.37
10.0
9.5
9.0
8.5
8.0
7.5
7.0
6.5
6.0
5.5
5.0
4.5
4.0
3.5
3.0
2.5
2.0
1.5
1.0
0.5
0
f1 (ppm)

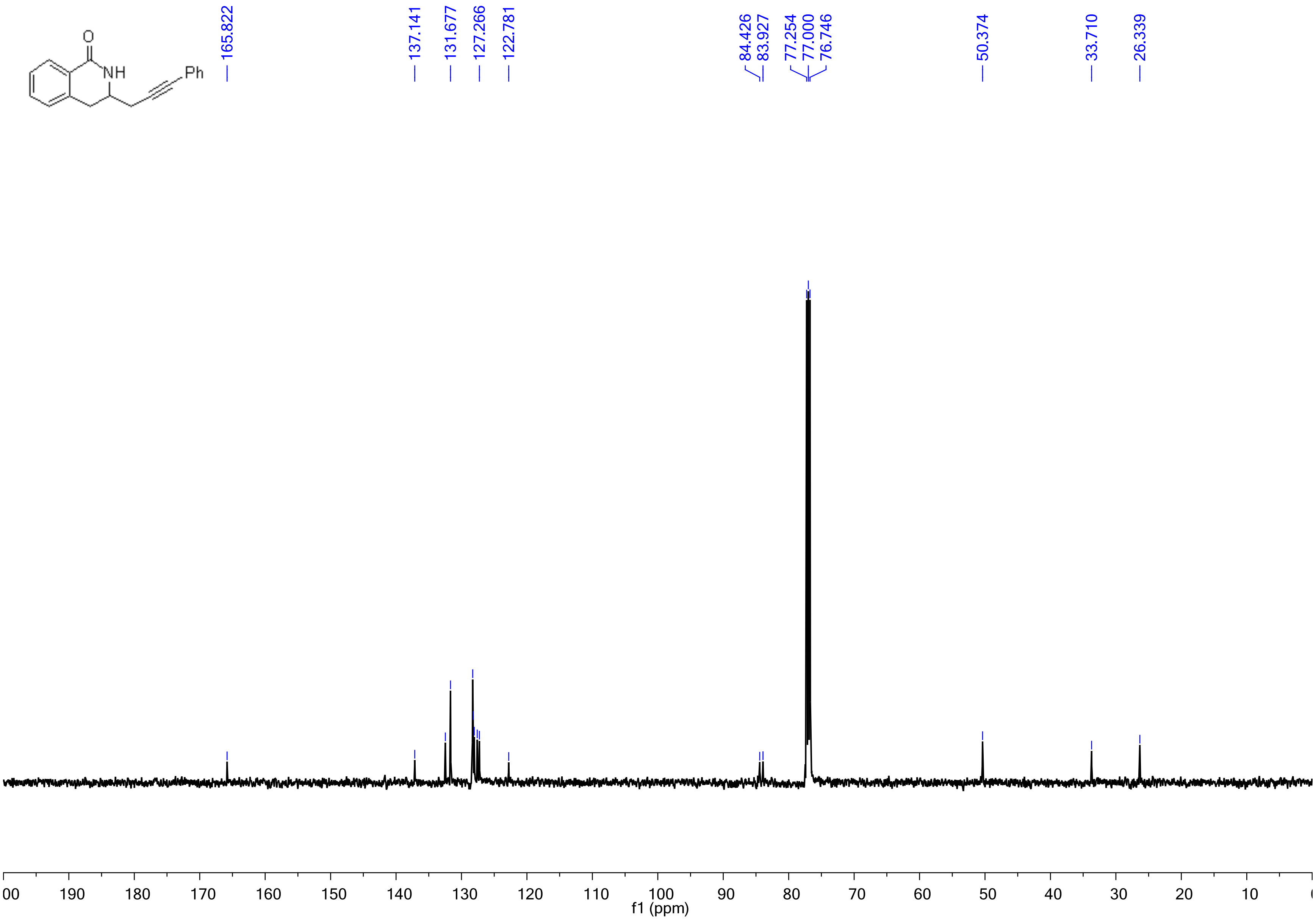
O
NH
Ph
165.822
137.141
131.677
127.266
122.781
84.426
83.927
77.254
77.000
76.746
50.374
33.710
26.339
00 190 180 170 160 150 140 130 120 110 100 90 80 70 60 50 40 30 20 10
f1 (ppm)

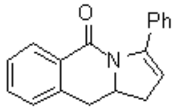
O
Ph
N

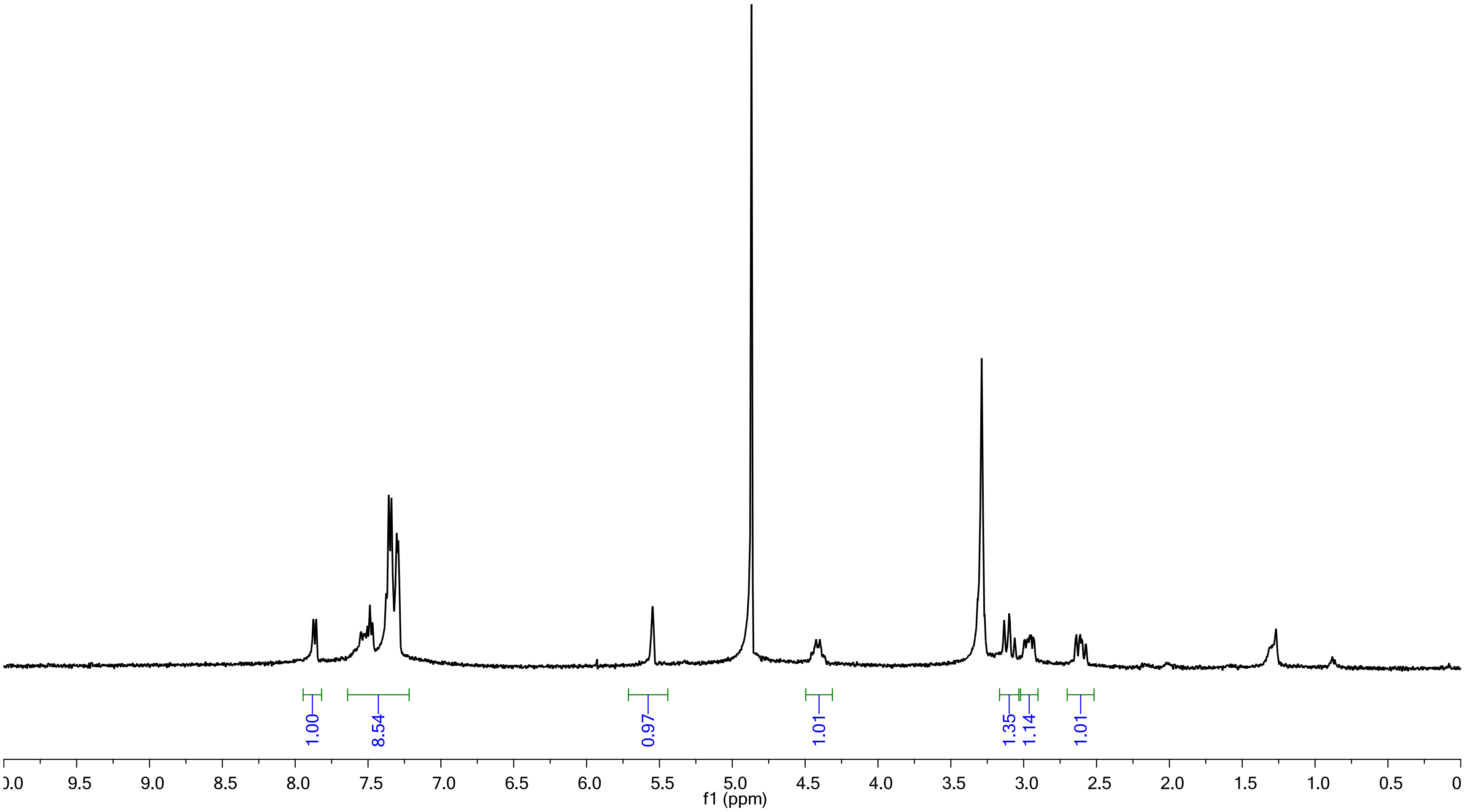
1.00
8.54
0.97
1.01
1.35
1.14
1.01
0.0
9.5
9.0
8.5
8.0
7.5
7.0
6.5
6.0
5.5
5.0
4.5
4.0
3.5
3.0
2.5
2.0
1.5
1.0
0.5
0
f1 (ppm)

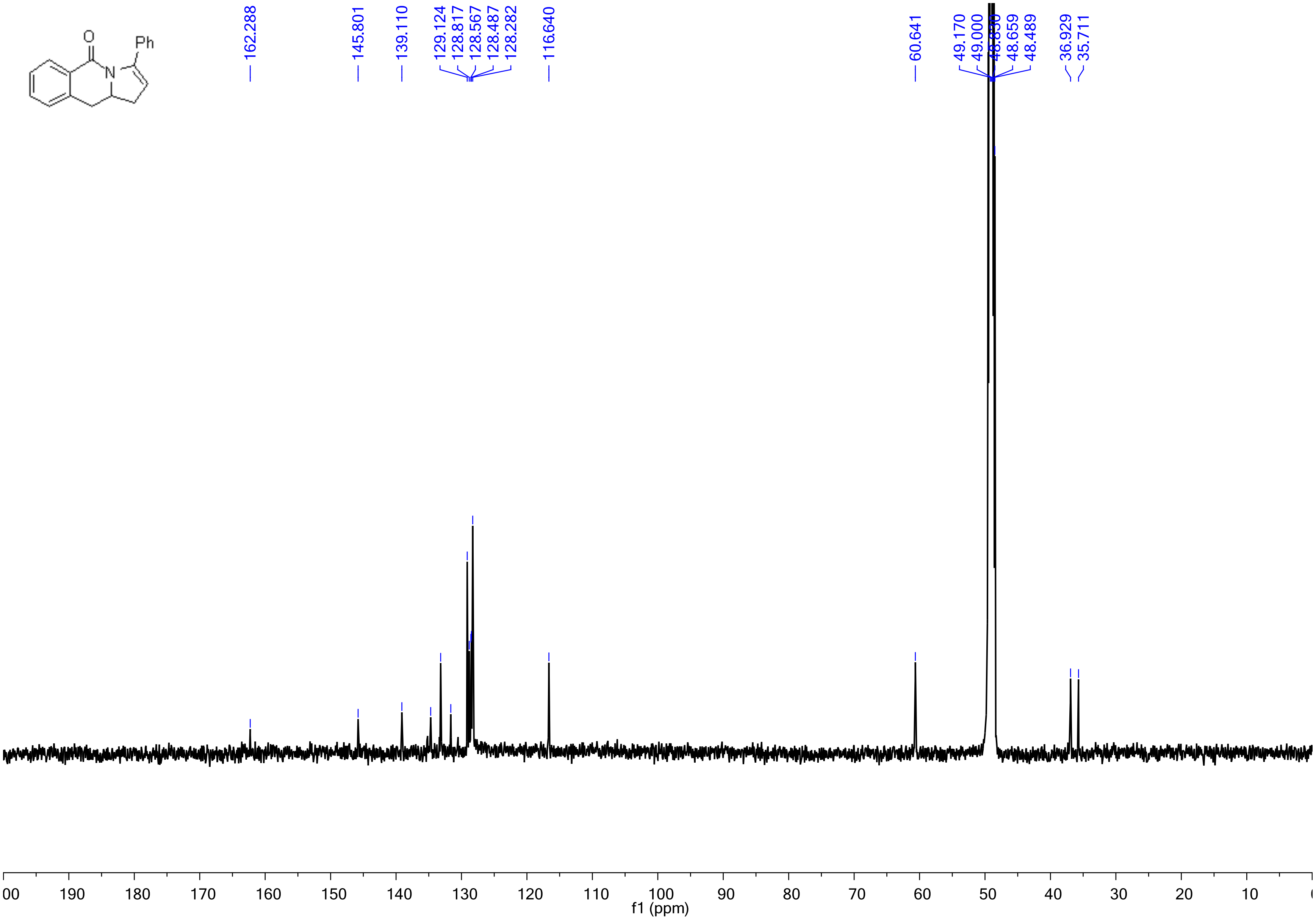
O
N
Ph
162.288
145.801
139.110
129.124
128.817
128.567
128.487
128.282
116.640
60.641
49.170
49.000
48.659
48.489
36.929
35.711
190
180
170
160
150
140
130
120
110
100
90
80
70
60
50
40
30
20
10
f1 (ppm)

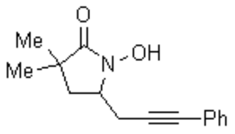
O
Me
Me
N
OH
Ph

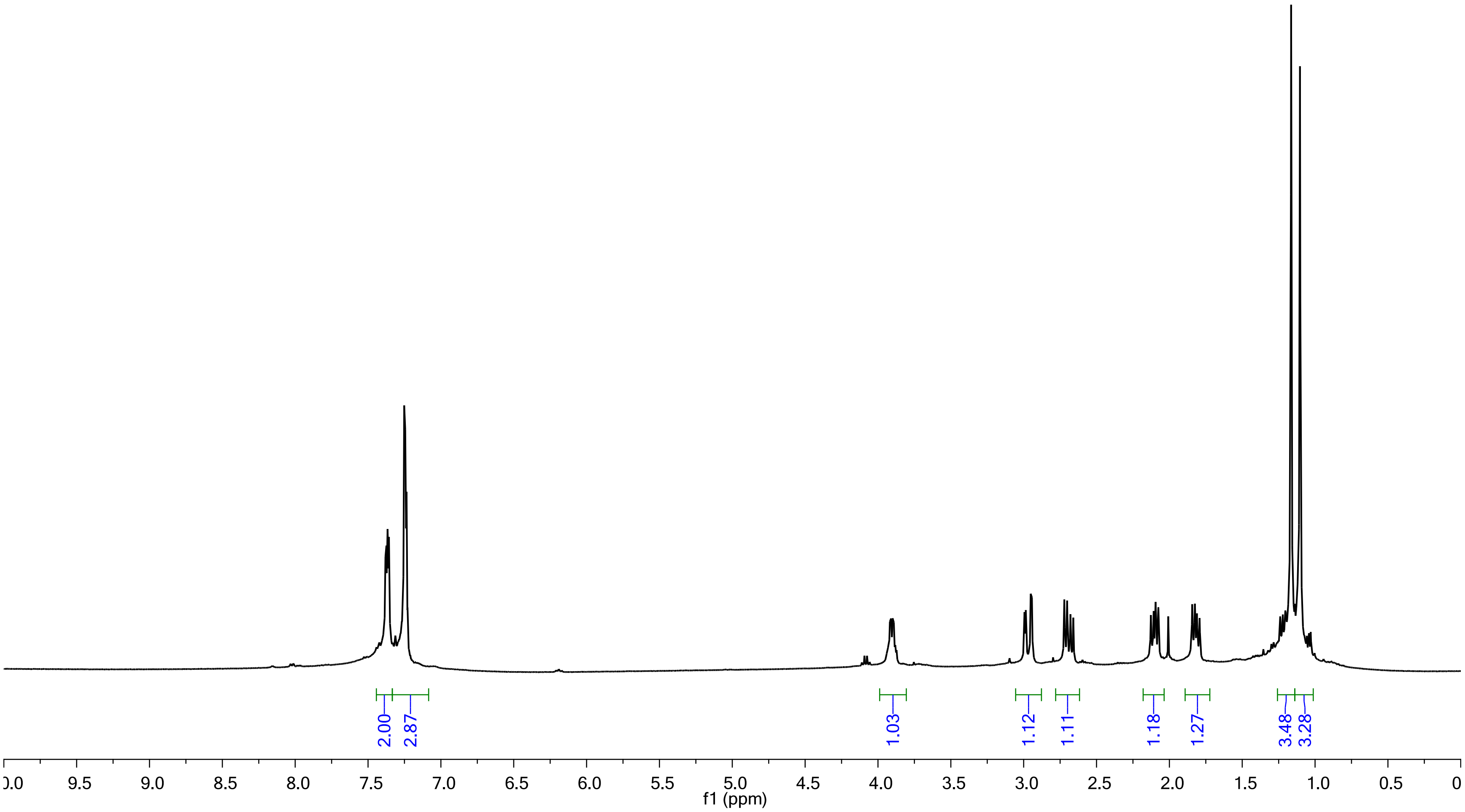
2.00
2.87
1.03
1.12
1.11
1.18
1.27
3.48
3.28
).0
9.5
9.0
8.5
8.0
7.5
7.0
6.5
6.0
5.5
5.0
4.5
4.0
3.5
3.0
2.5
2.0
1.5
1.0
0.5
0
f1 (ppm)

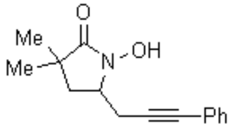
Me
Me
O
N
OH
Ph

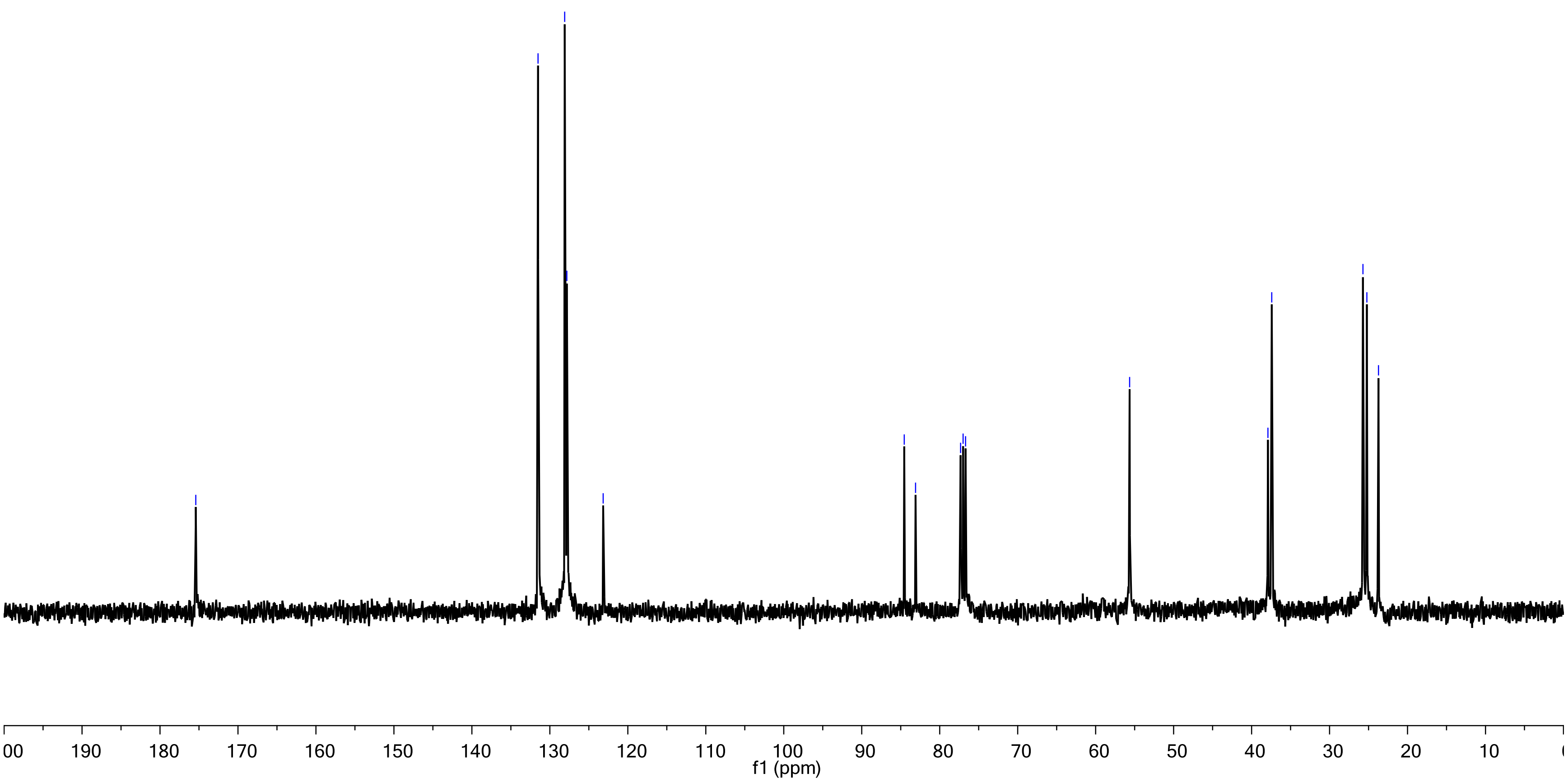
175.411
131.514
127.821
123.162
84.548
83.097
77.319
77.000
76.682
55.643
37.905
37.414
25.718
25.214
23.726
00
190
180
170
160
150
140
130
120
110
100
90
80
70
60
50
40
30
20
10
f1 (ppm)

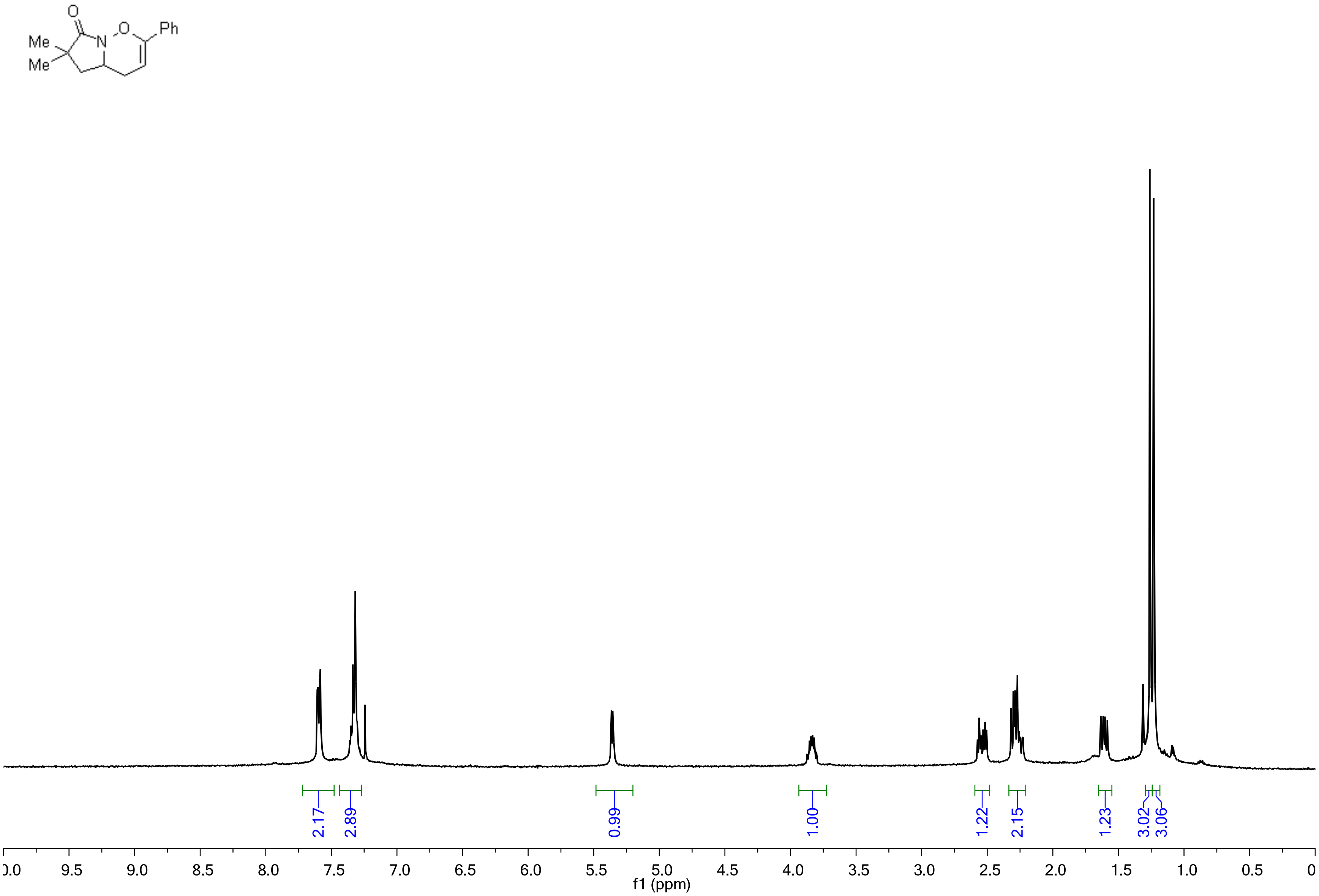

O
Me
Me
N
O
Ph
2.17
2.89
0.99
1.00
1.22
2.15
1.23
3.02
3.06
9.5
9.0
8.5
8.0
7.5
7.0
6.5
6.0
5.5
5.0
4.5
4.0
3.5
3.0
2.5
2.0
1.5
1.0
0.5
f1 (ppm)

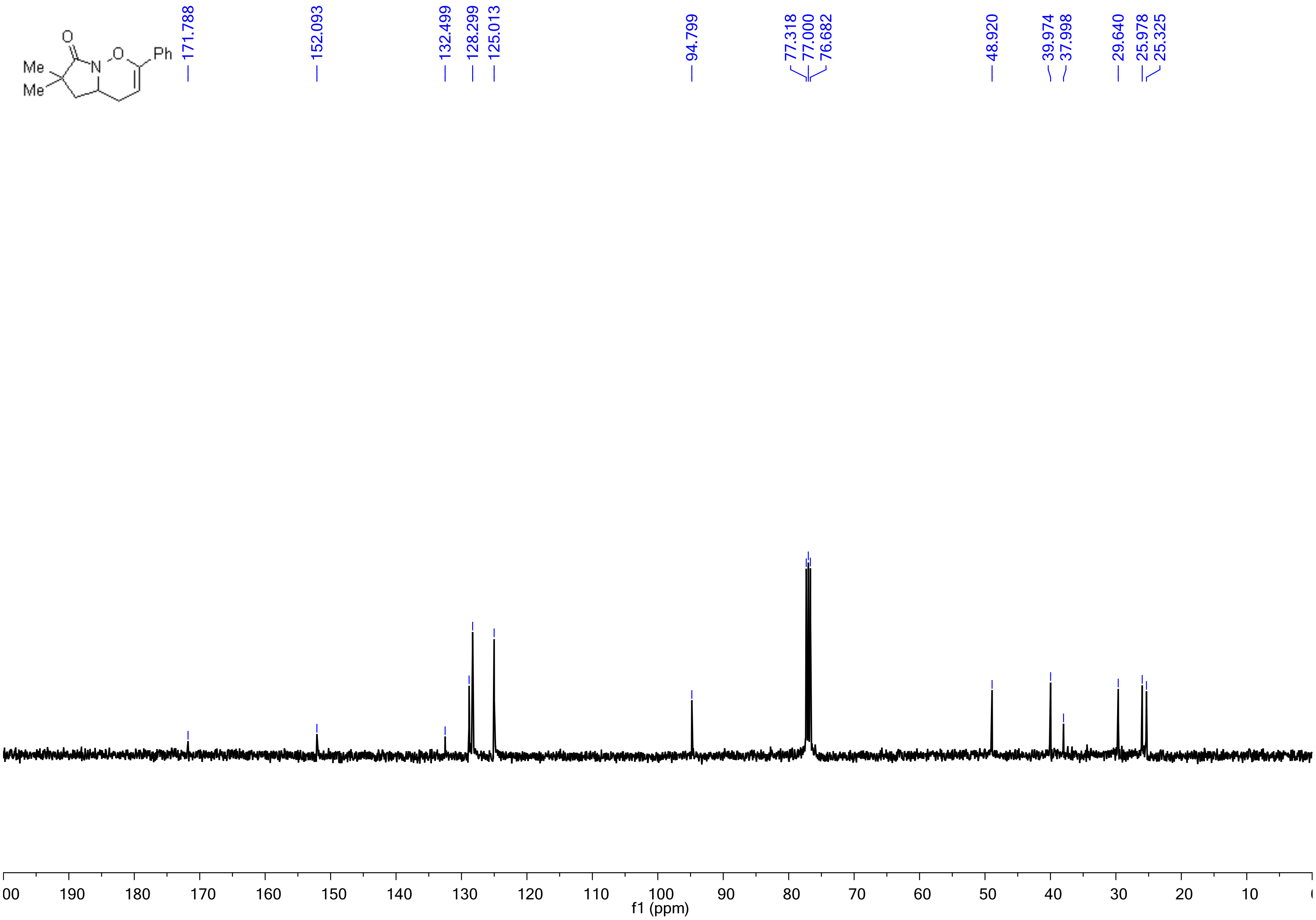
O
N
O
Ph
Me
Me
171.788
152.093
132.499
128.299
125.013
94.799
77.318
77.000
76.682
48.920
39.974
37.998
29.640
25.978
25.325
f1 (ppm)

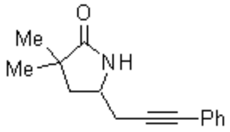
O
Me
Me
NH
Ph

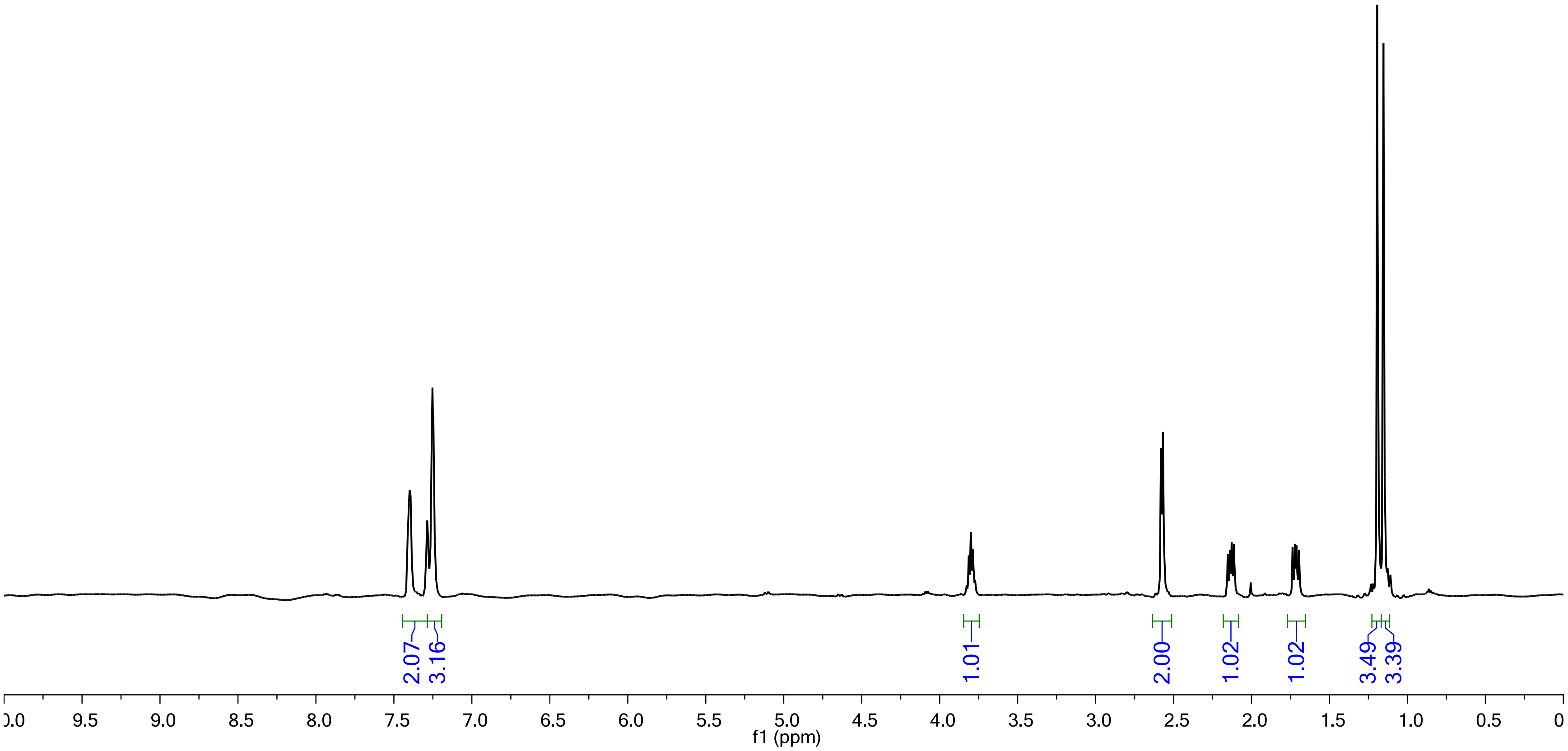
2.07
3.16
1.01
2.00
1.02
1.02
3.49
3.39
9.5
9.0
8.5
8.0
7.5
7.0
6.5
6.0
5.5
5.0
4.5
4.0
3.5
3.0
2.5
2.0
1.5
1.0
0.5
0
f1 (ppm)

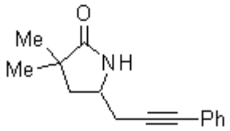

Me
Me
O
NH
Ph

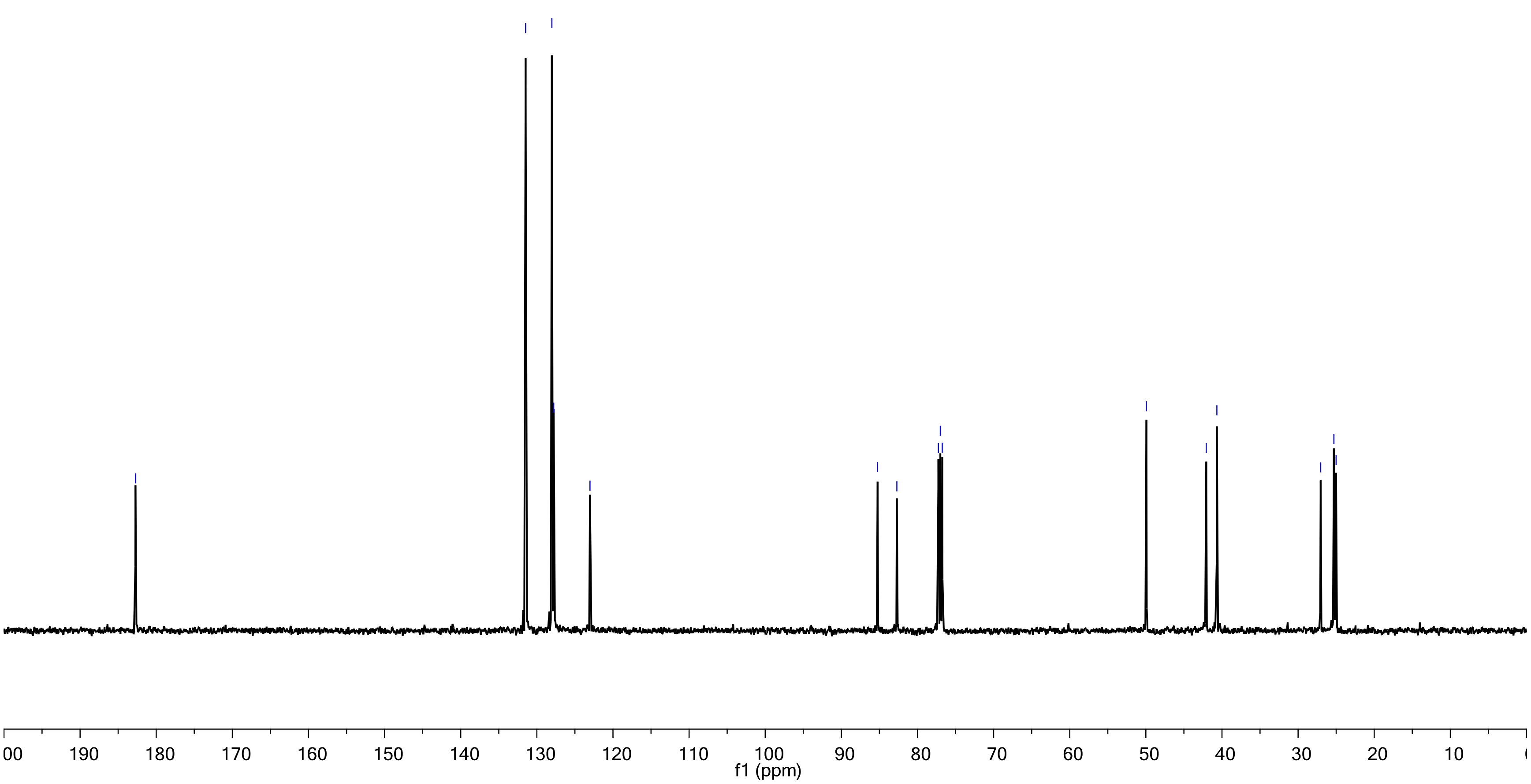

182.72
131.47
127.79
123.02
85.24
82.71
77.25
77.00
76.75
49.93
42.06
40.67
27.04
25.30
25.01
00
190
180
170
160
150
140
130
120
110
100
90
80
70
60
50
40
30
20
10
f1 (ppm)

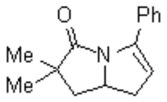
O
Ph
Me
Me
N

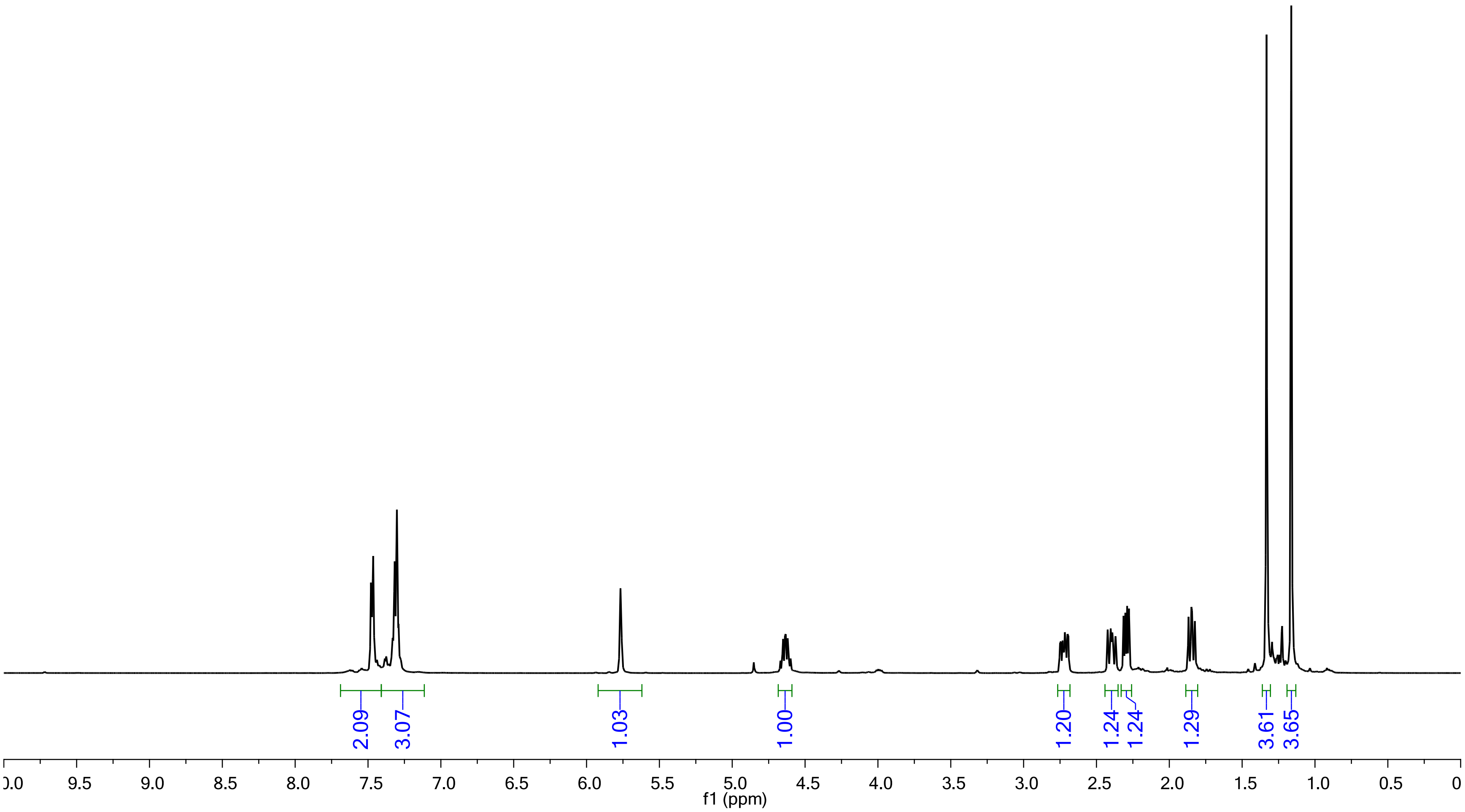
2.09
3.07
1.03
1.00
1.20
1.24
1.24
1.29
3.61
3.65
f1 (ppm)

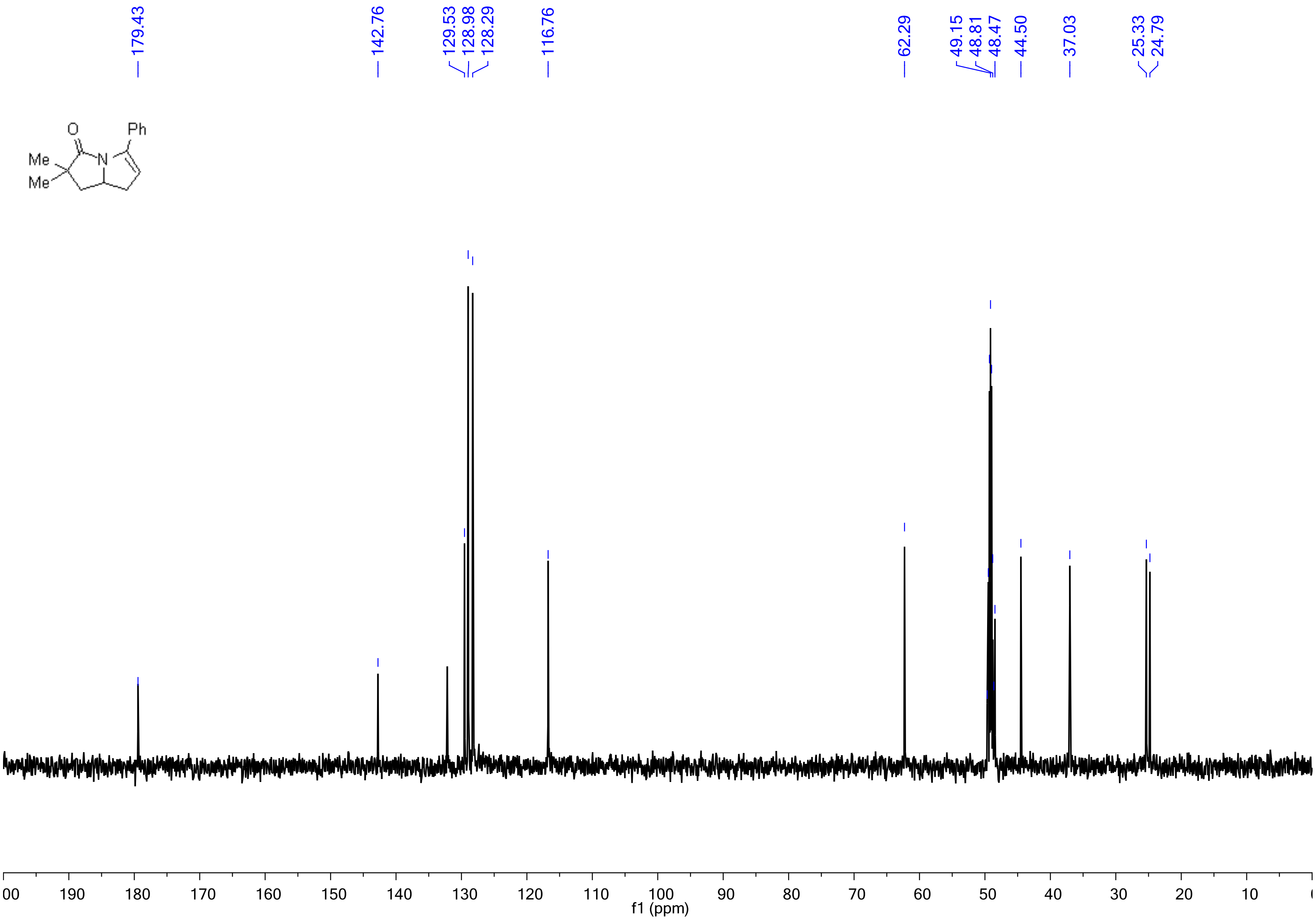

179.43
142.76
129.53
128.98
128.29
116.76
62.29
49.15
48.81
48.47
44.50
37.03
25.33
24.79
O
Ph
Me
N
Me
f1 (ppm)

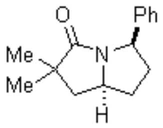
O
Ph
Me
Me
N
H

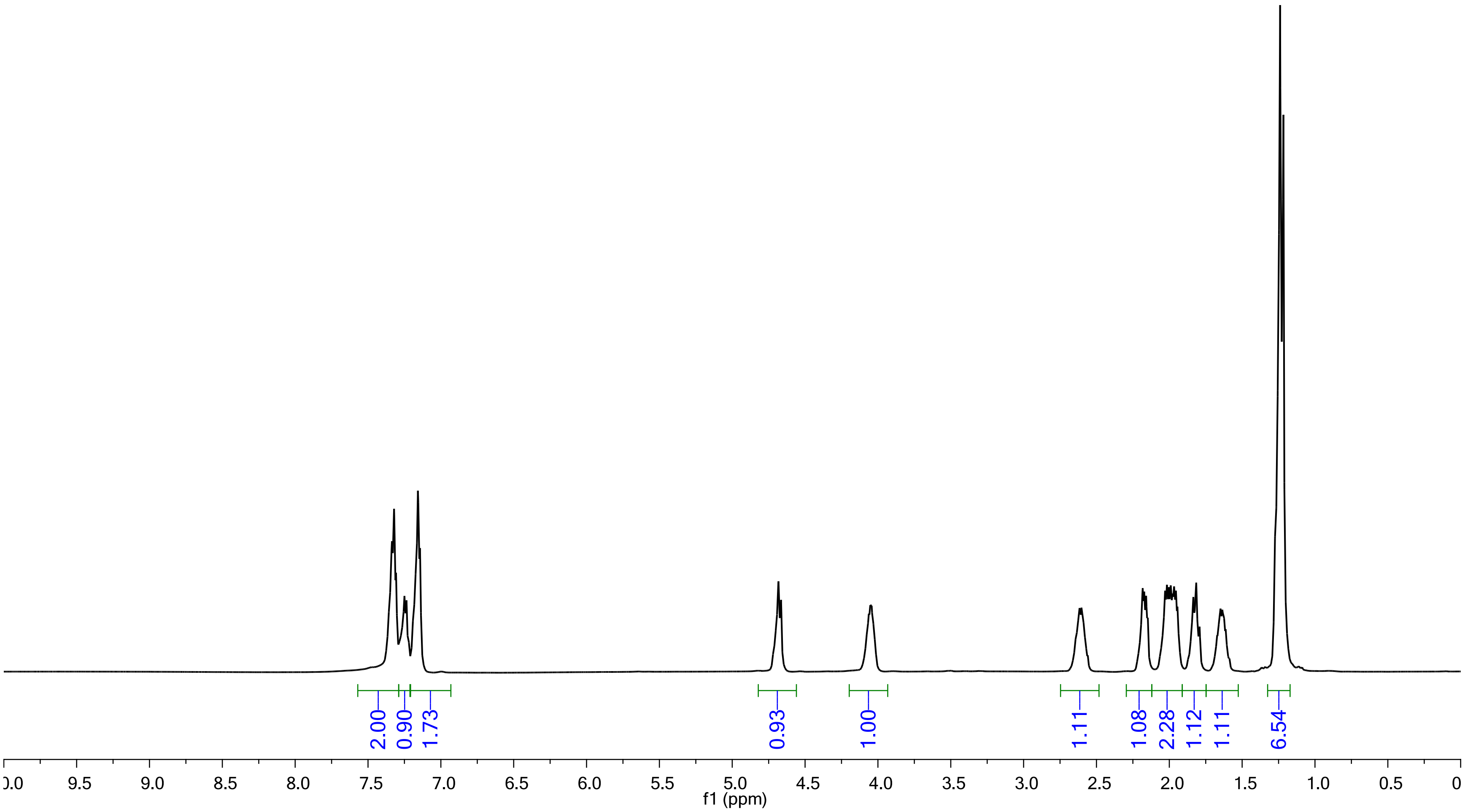
2.00
0.90
1.73
0.93
1.00
1.11
1.08
2.28
1.12
1.11
6.54
f1 (ppm)

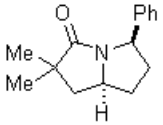
O
Ph
Me
Me
N
H

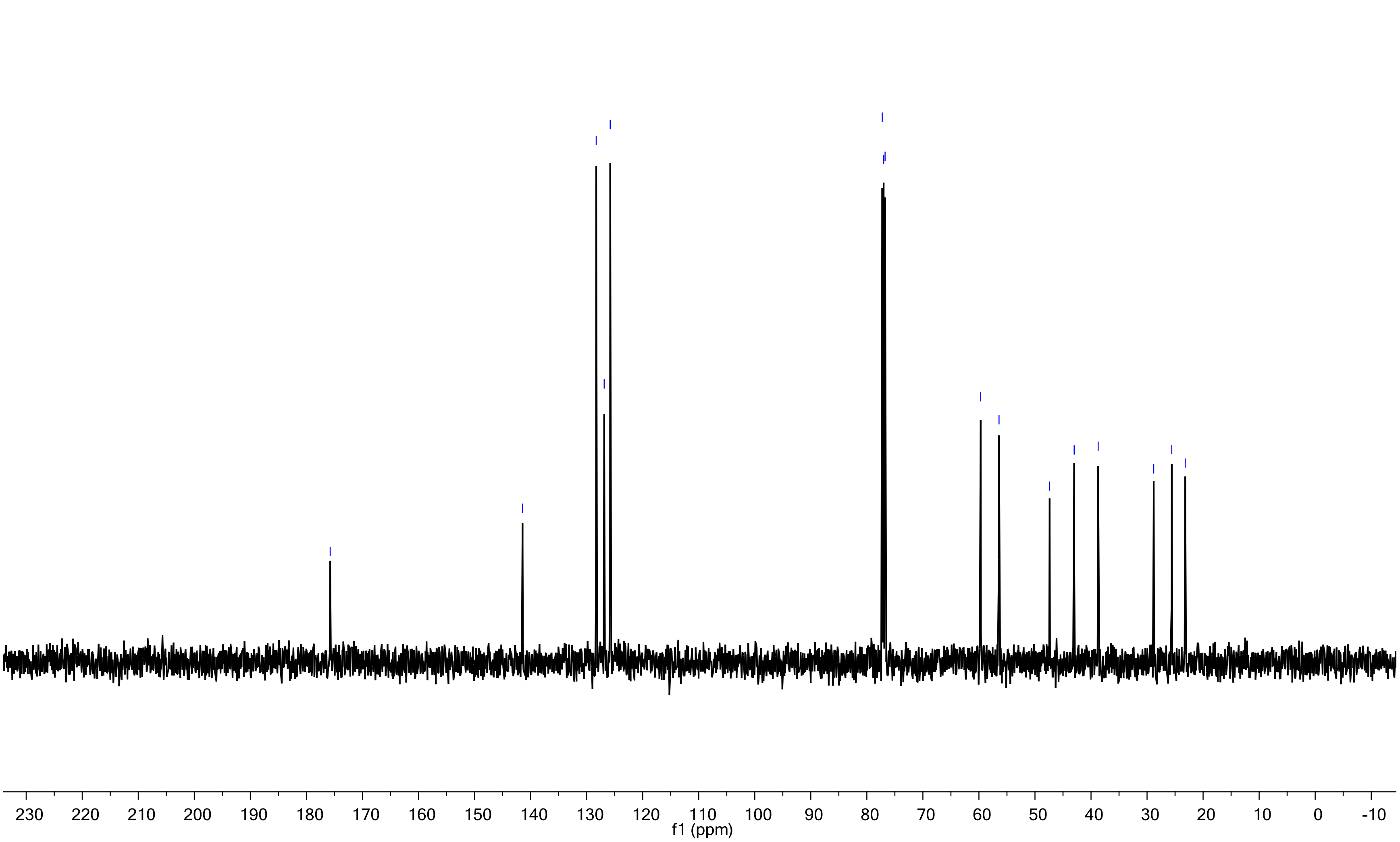
175.76
141.43
128.29
126.87
125.79
77.26
77.00
76.75
59.69
56.41
47.39
43.01
38.72
28.82
25.59
23.19
230 220 210 200 190 180 170 160 150 140 130 120 110 100 90 80 70 60 50 40 30 20 10 0 -10
f1 (ppm)

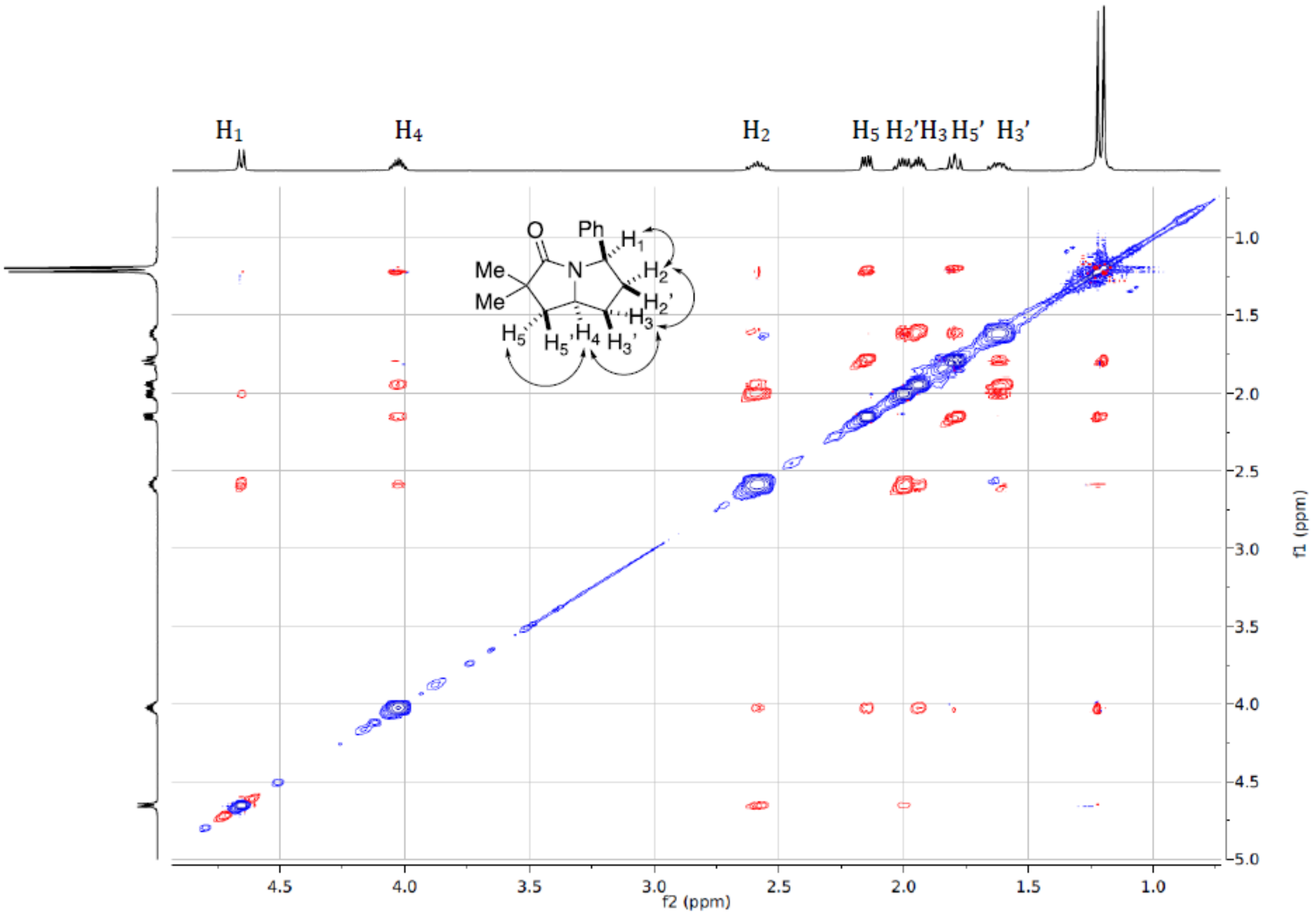

H1
H4
H2
H5 H2'H3 H5' H3'
O
Ph
Me
Me
N
H1
H2
H2'
H3
H5
H5'
H4
H3'
f2 (ppm)
f1 (ppm)

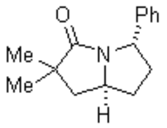
O
Ph
Me
Me
N
H

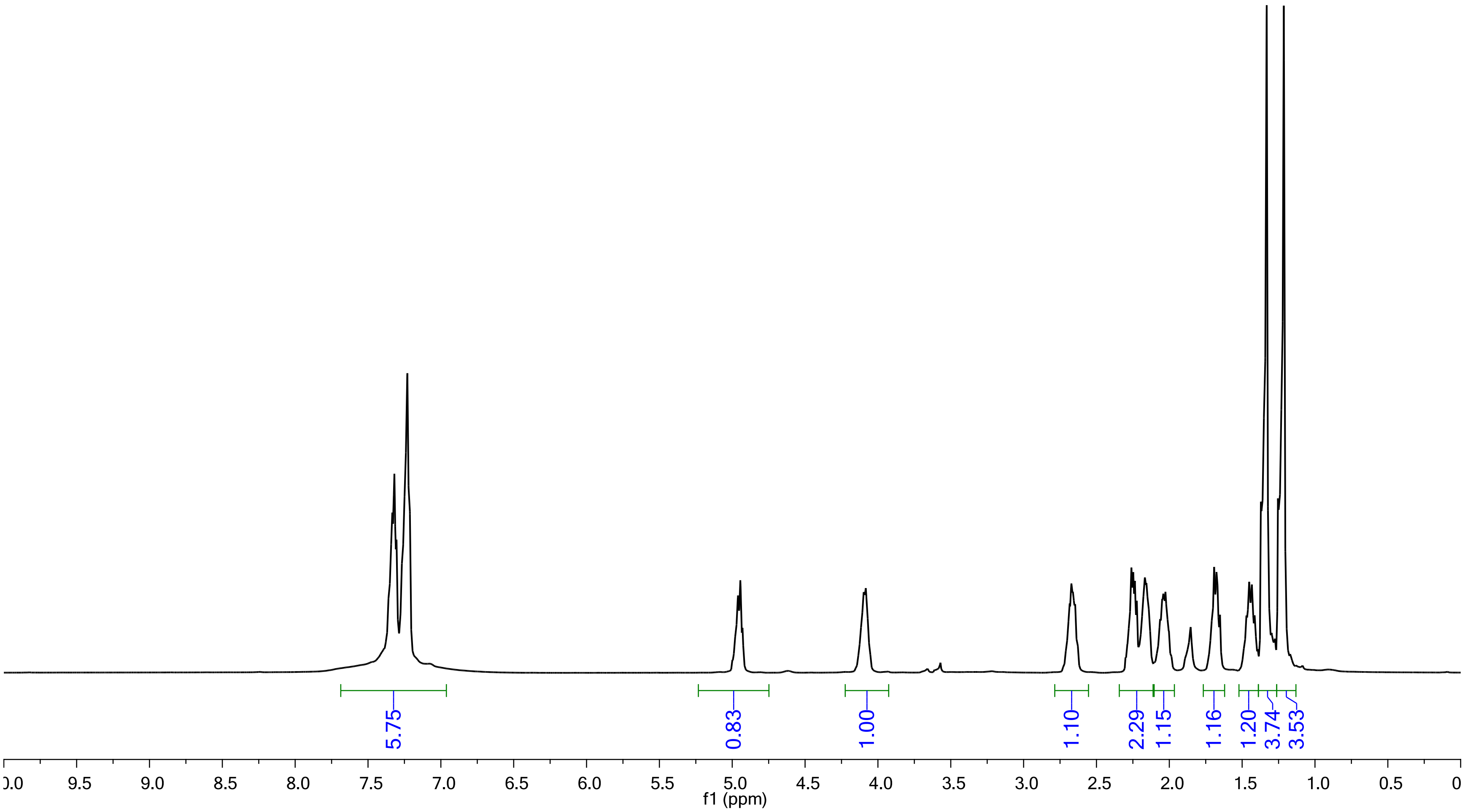
5.75
0.83
1.00
1.10
2.29
1.15
1.16
1.20
3.74
3.53
).0
9.5
9.0
8.5
8.0
7.5
7.0
6.5
6.0
5.5
5.0
4.5
4.0
3.5
3.0
2.5
2.0
1.5
1.0
0.5
0
f1 (ppm)

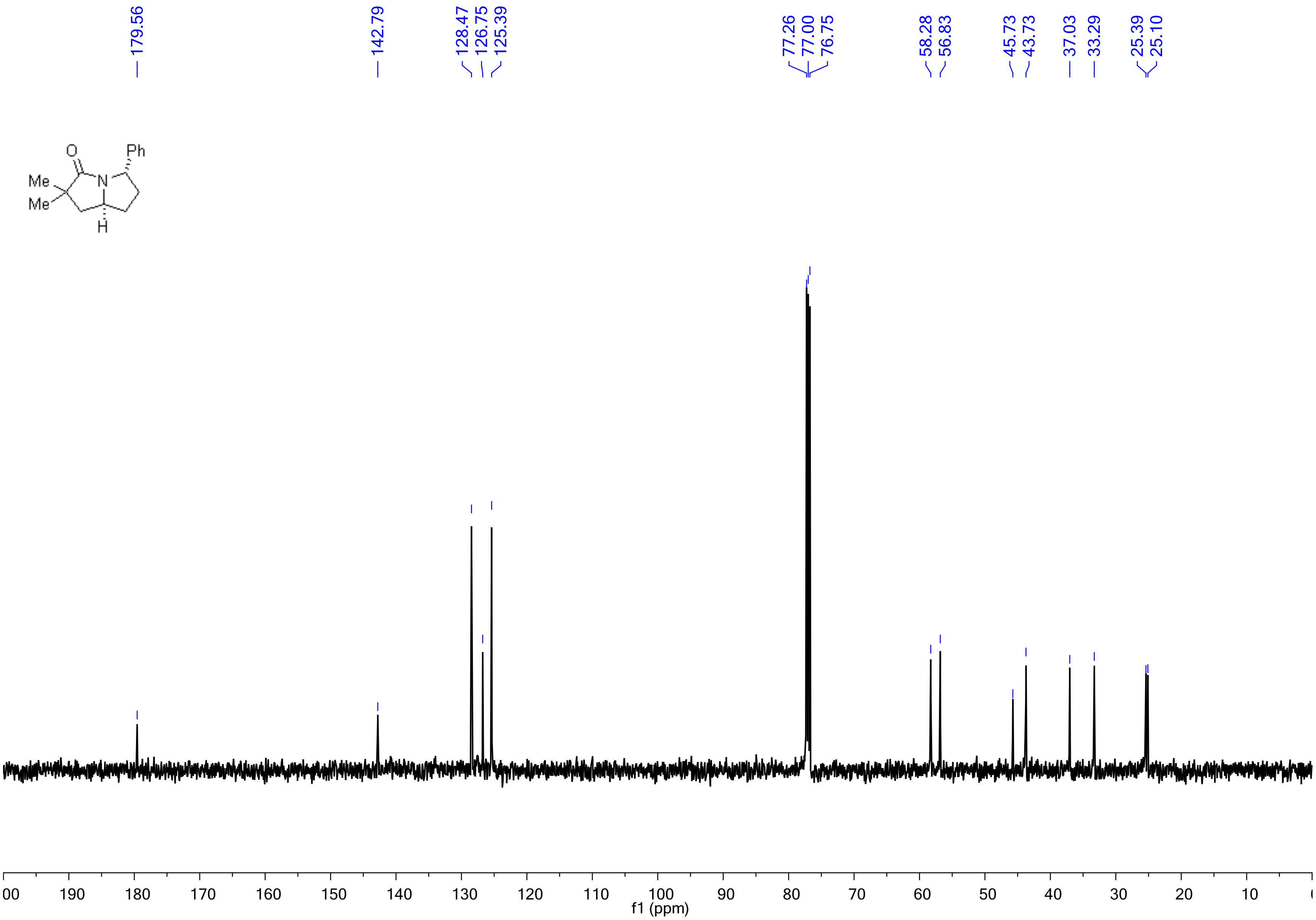
179.56
142.79
128.47
126.75
125.39
77.26
77.00
76.75
58.28
56.83
45.73
43.73
37.03
33.29
25.39
25.10
O
Ph
Me
Me
N
H
00
190
180
170
160
150
140
130
120
110
100
90
80
70
60
50
40
30
20
10
f1 (ppm)

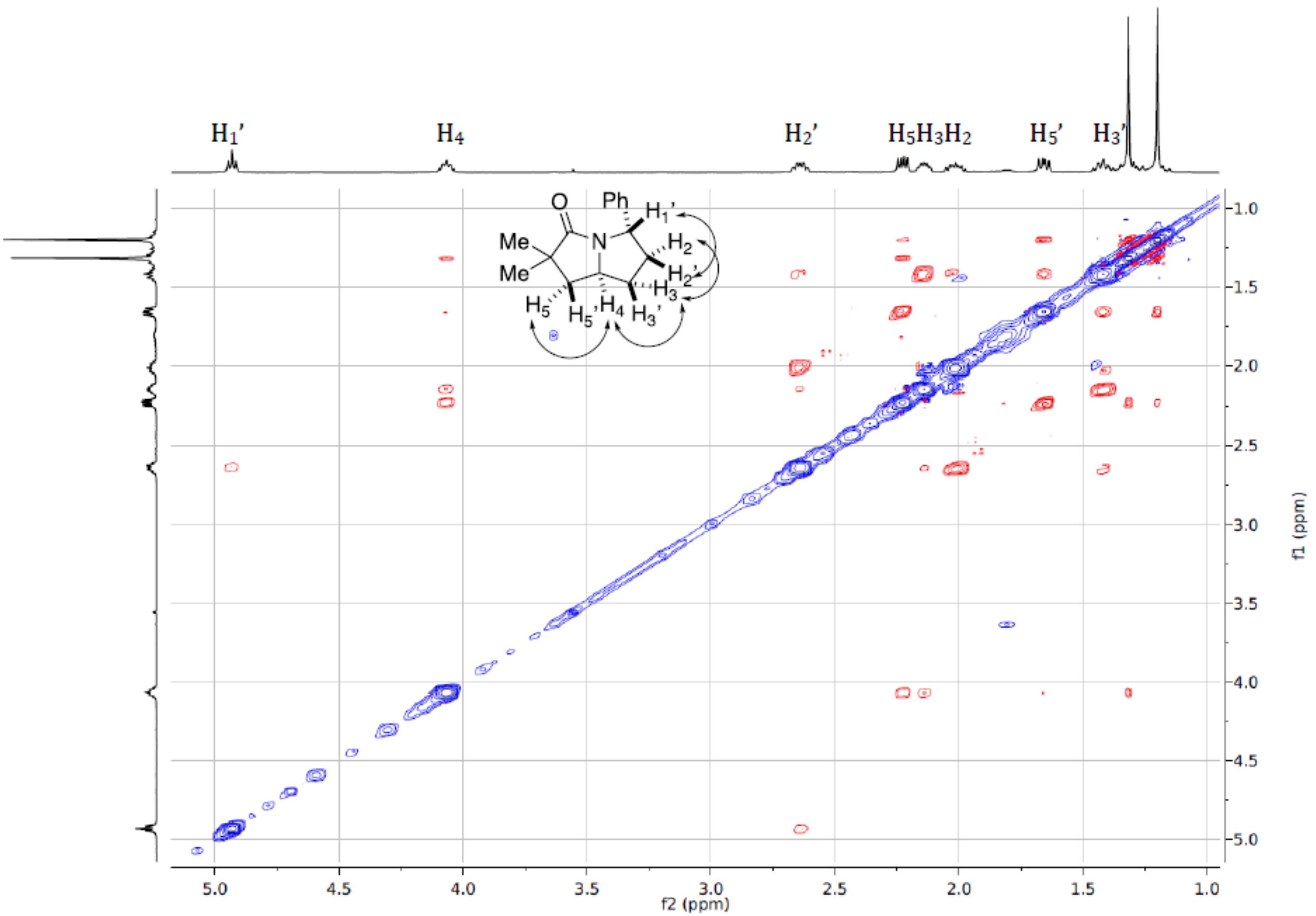

H1'
H4
H2'
H5H3H2
H5'
H3'
O
Ph
H1'
Me
N
H2
Me
H2'
H3
H5
H5'
H4
H3'
f1 (ppm)
f2 (ppm)

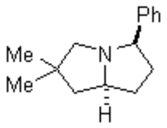
Ph
Me
Me
N
H

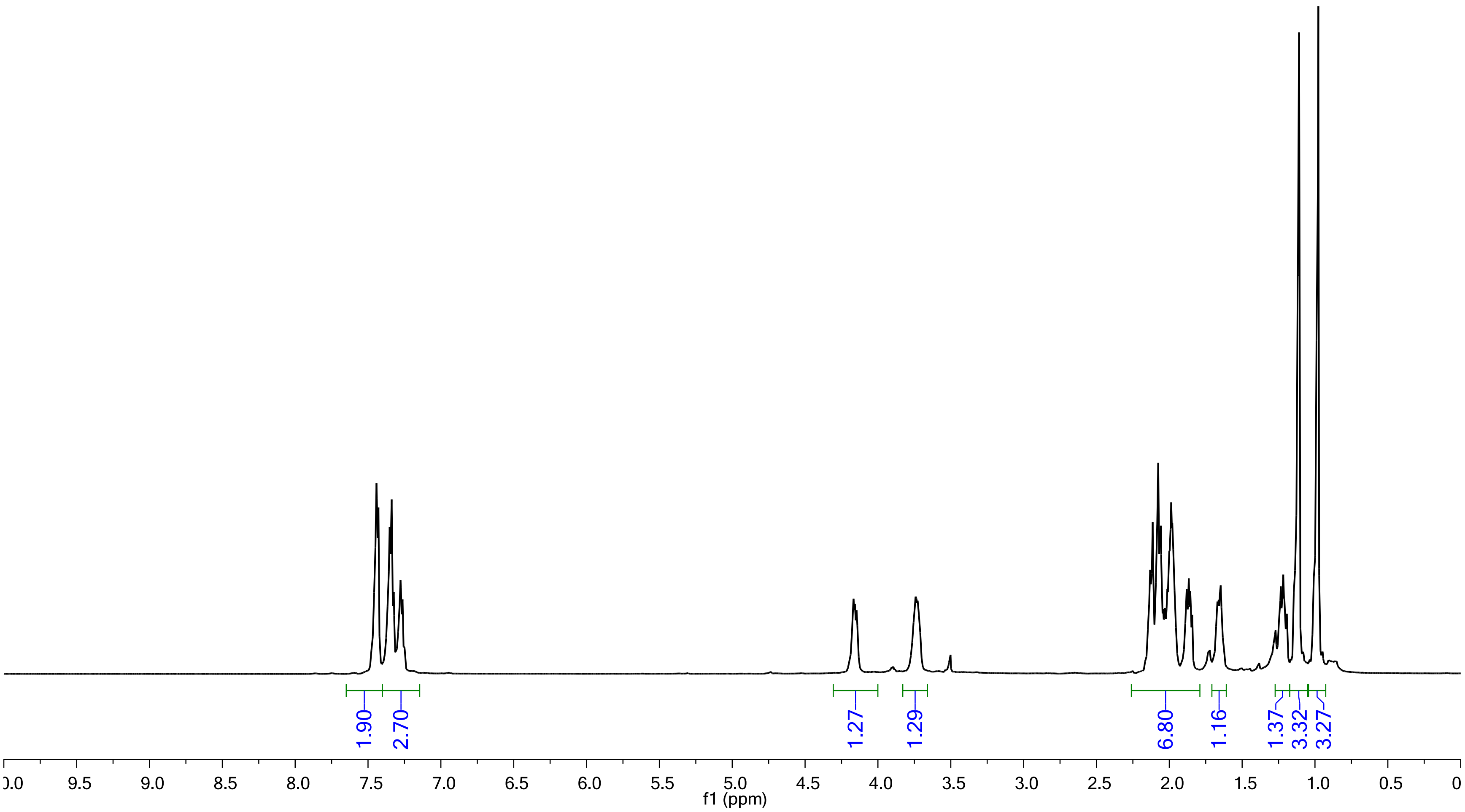
1.90
2.70
1.27
1.29
6.80
1.16
1.37
3.32
3.27
9.5
9.0
8.5
8.0
7.5
7.0
6.5
6.0
5.5
5.0
4.5
4.0
3.5
3.0
2.5
2.0
1.5
1.0
0.5
f1 (ppm)

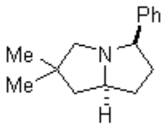
Ph
Me
Me
N
H

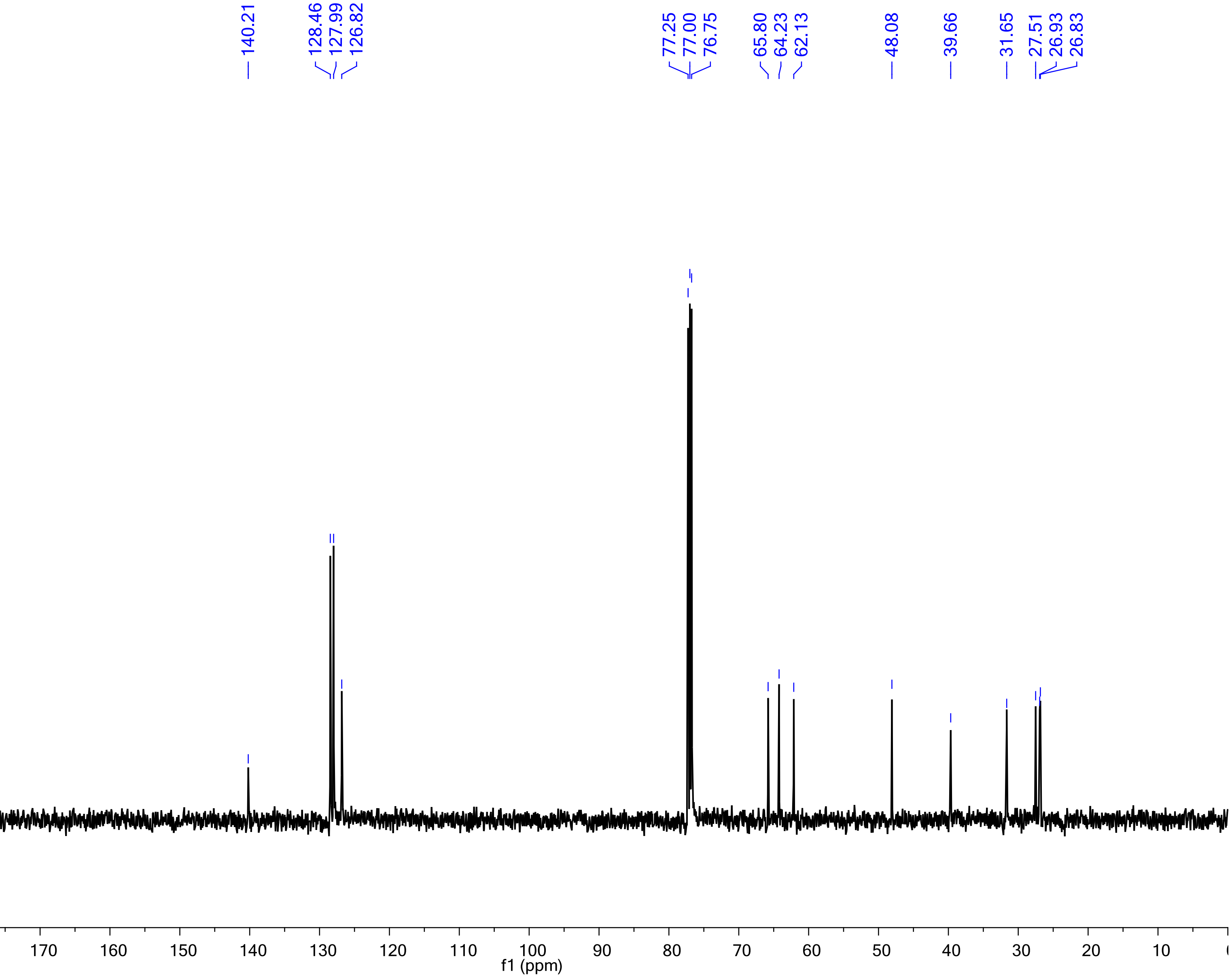
140.21
128.46
127.99
126.82
77.25
77.00
76.75
65.80
64.23
62.13
48.08
39.66
31.65
27.51
26.93
26.83
f1 (ppm)

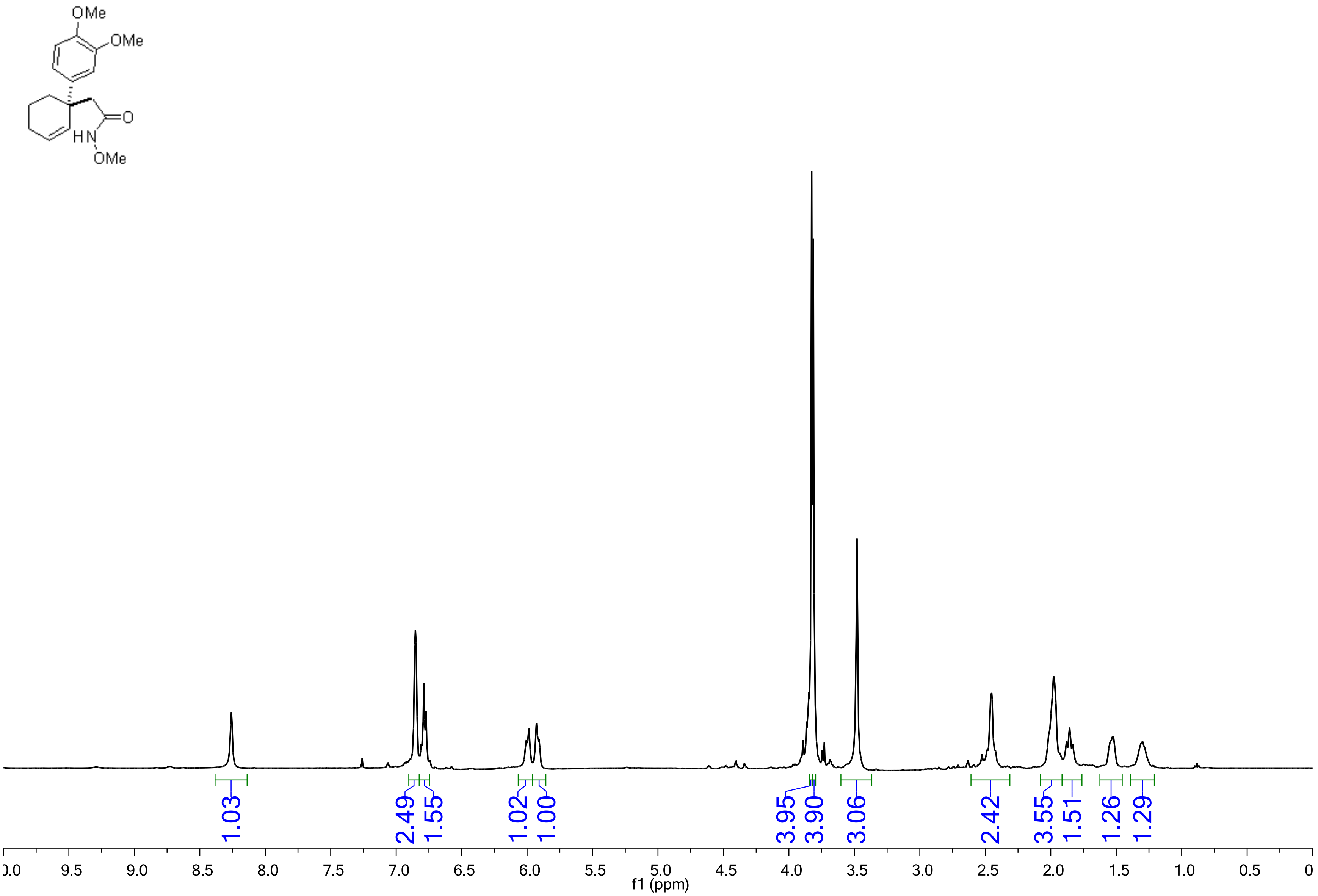
OMe
OMe
O
HN
OMe
1.03
2.49
1.55
1.02
1.00
3.95
3.90
3.06
2.42
3.55
1.51
1.26
1.29
9.5
9.0
8.5
8.0
7.5
7.0
6.5
6.0
5.5
5.0
4.5
4.0
3.5
3.0
2.5
2.0
1.5
1.0
0.5
f1 (ppm)

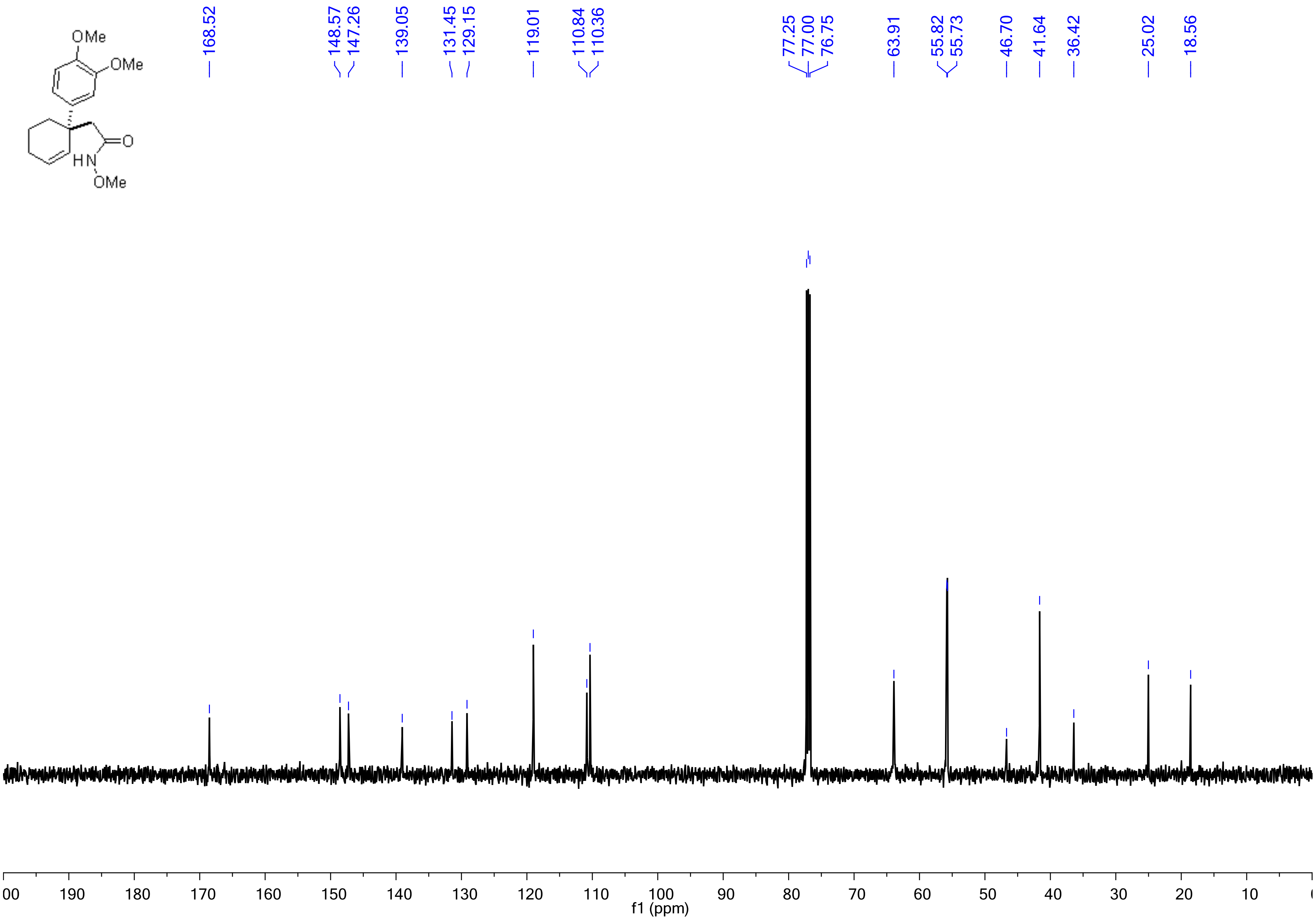
OMe
OMe
O
HN
OMe
168.52
148.57
147.26
139.05
131.45
129.15
119.01
110.84
110.36
77.25
77.00
76.75
63.91
55.82
55.73
46.70
41.64
36.42
25.02
18.56
f1 (ppm)

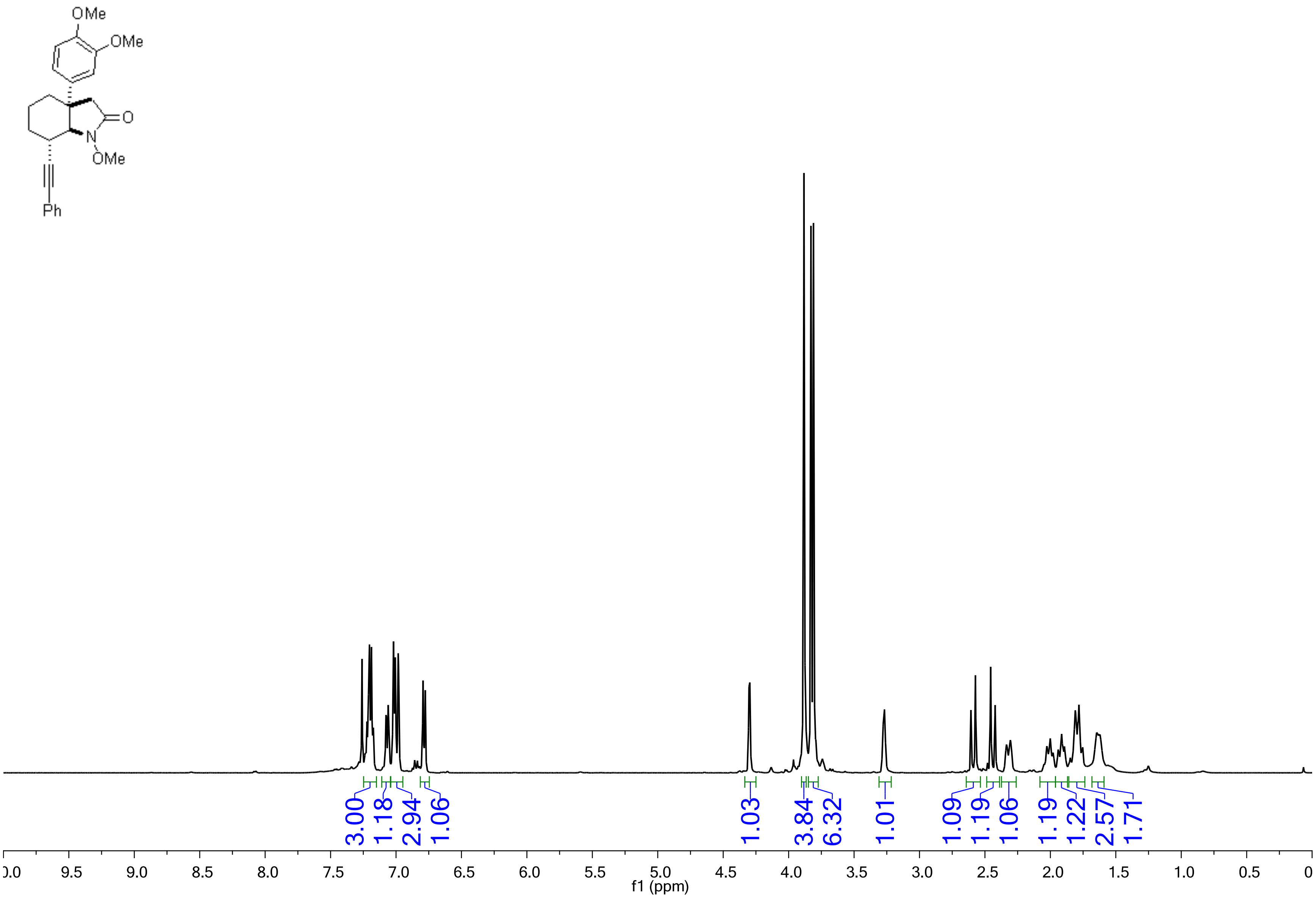

OMe
OMe
O
N
OMe
Ph
3.00
1.18
2.94
1.06
1.03
3.84
6.32
1.01
1.09
1.19
1.06
1.19
1.22
2.57
1.71
f1 (ppm)

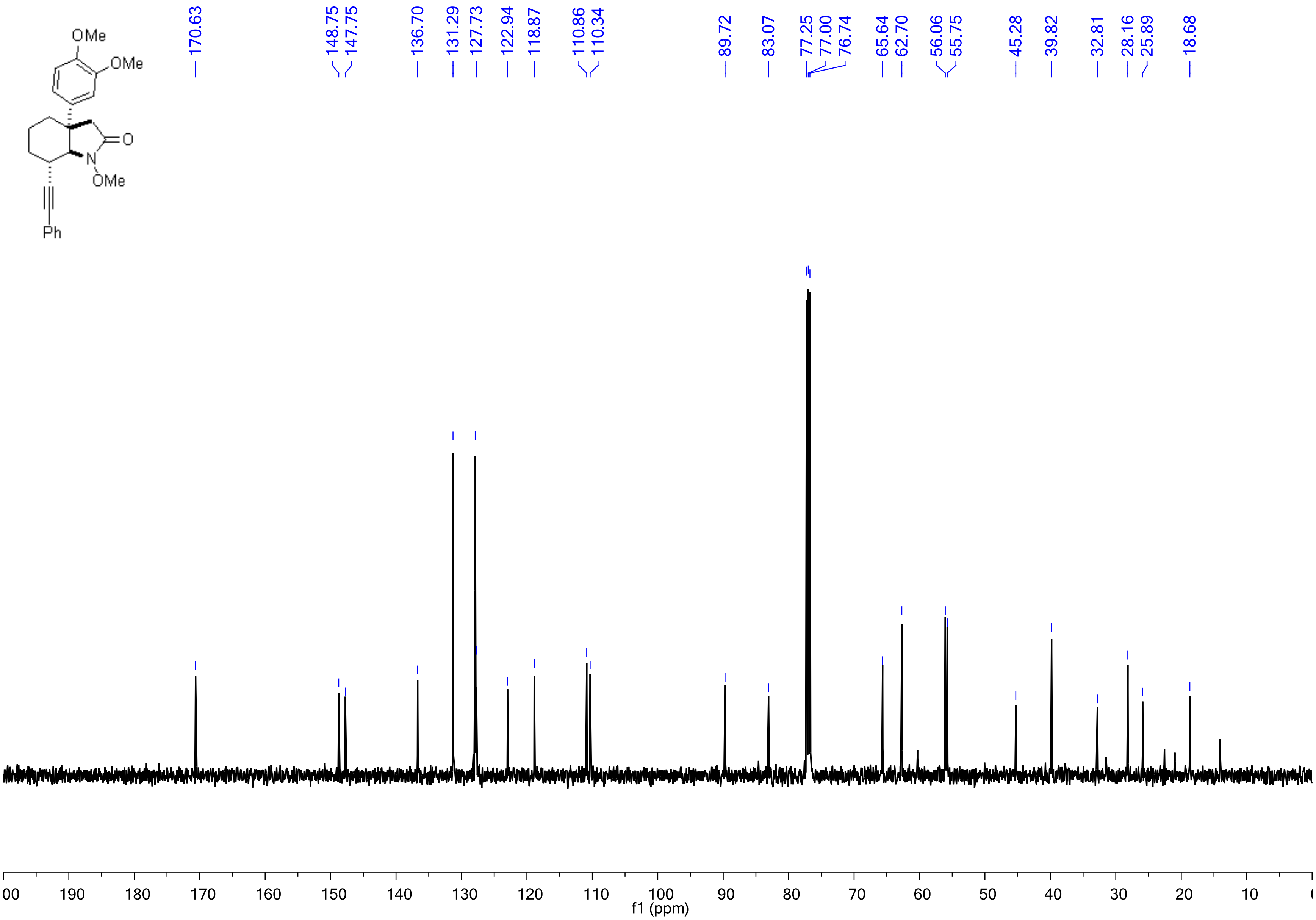
OMe
OMe
O
N
OMe
Ph
170.63
148.75
147.75
136.70
131.29
127.73
122.94
118.87
110.86
110.34
89.72
83.07
77.25
77.00
76.74
65.64
62.70
56.06
55.75
45.28
39.82
32.81
28.16
25.89
18.68
f1 (ppm)

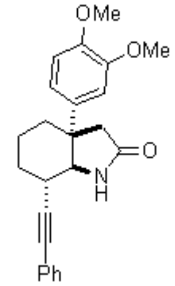

OMe
OMe
O
N
H
Ph

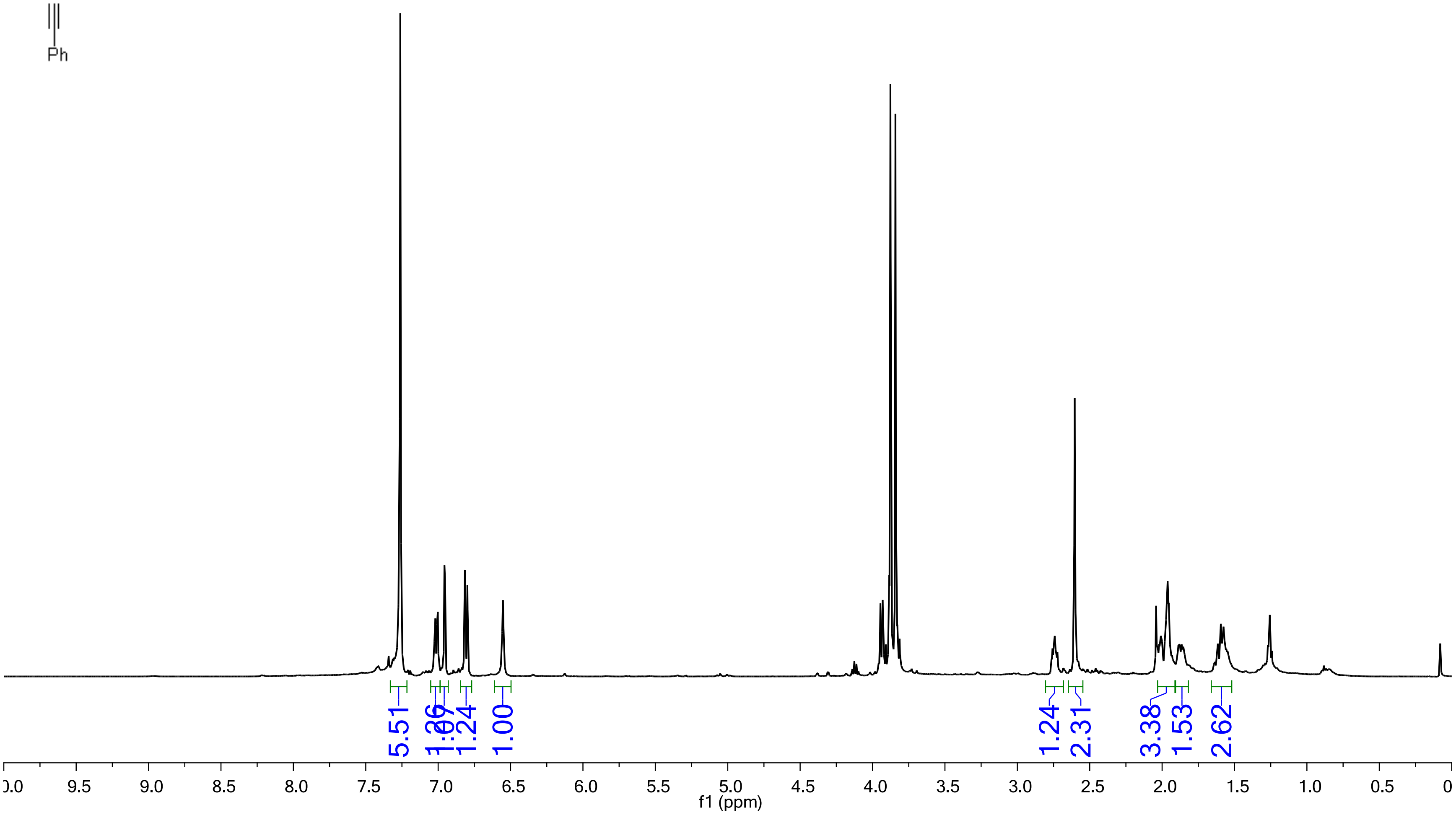

5.51
1.36
1.07
1.24
1.00
1.24
2.31
3.38
1.53
2.62
10.0
9.5
9.0
8.5
8.0
7.5
7.0
6.5
6.0
5.5
5.0
4.5
4.0
3.5
3.0
2.5
2.0
1.5
1.0
0.5
0
f1 (ppm)

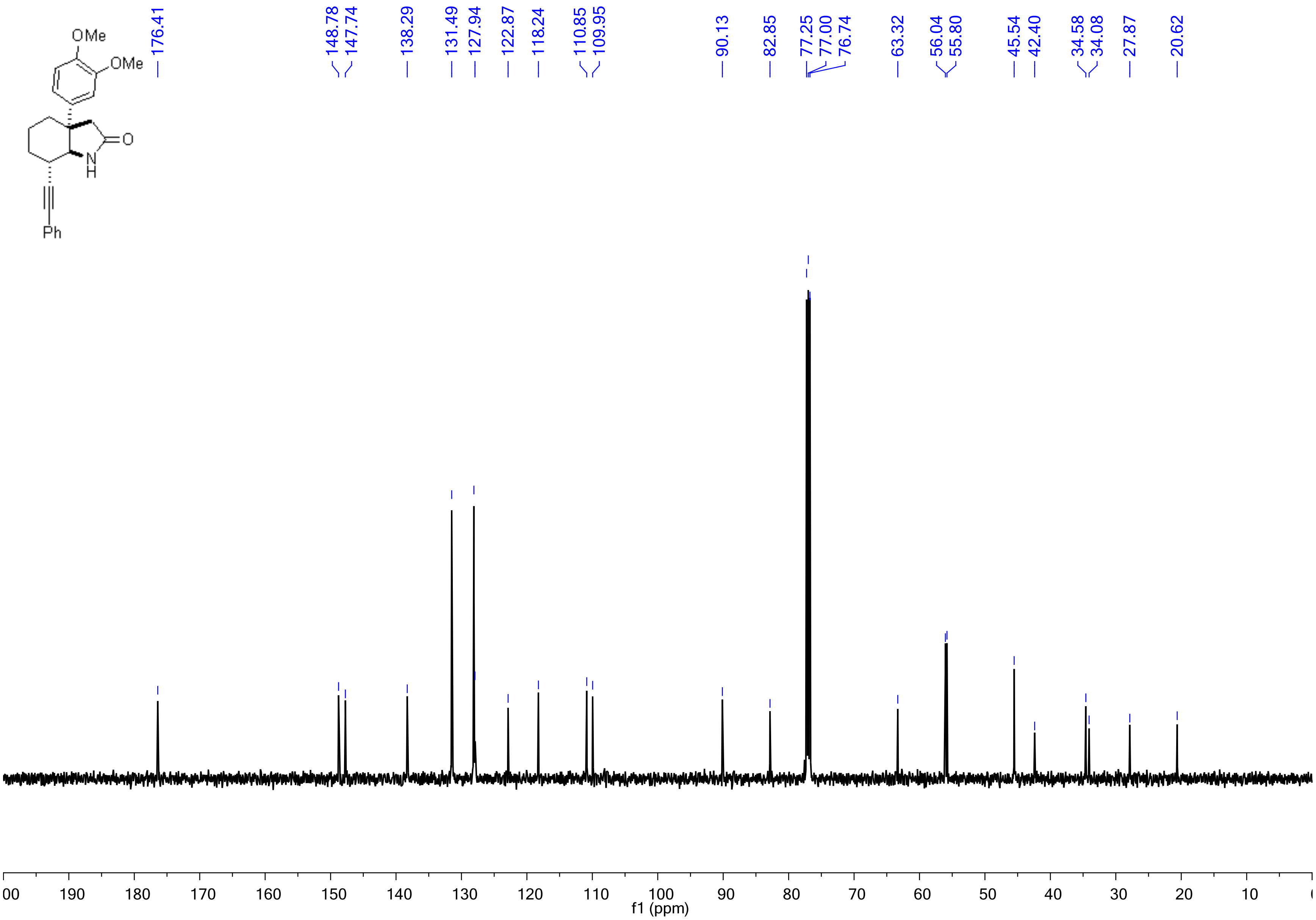
OMe
OMe
O
N
H
Ph
176.41
148.78
147.74
138.29
131.49
127.94
122.87
118.24
110.85
109.95
90.13
82.85
77.25
77.00
76.74
63.32
56.04
55.80
45.54
42.40
34.58
34.08
27.87
20.62
f1 (ppm)

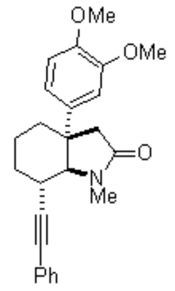

OMe
OMe
O
N
Me
Ph

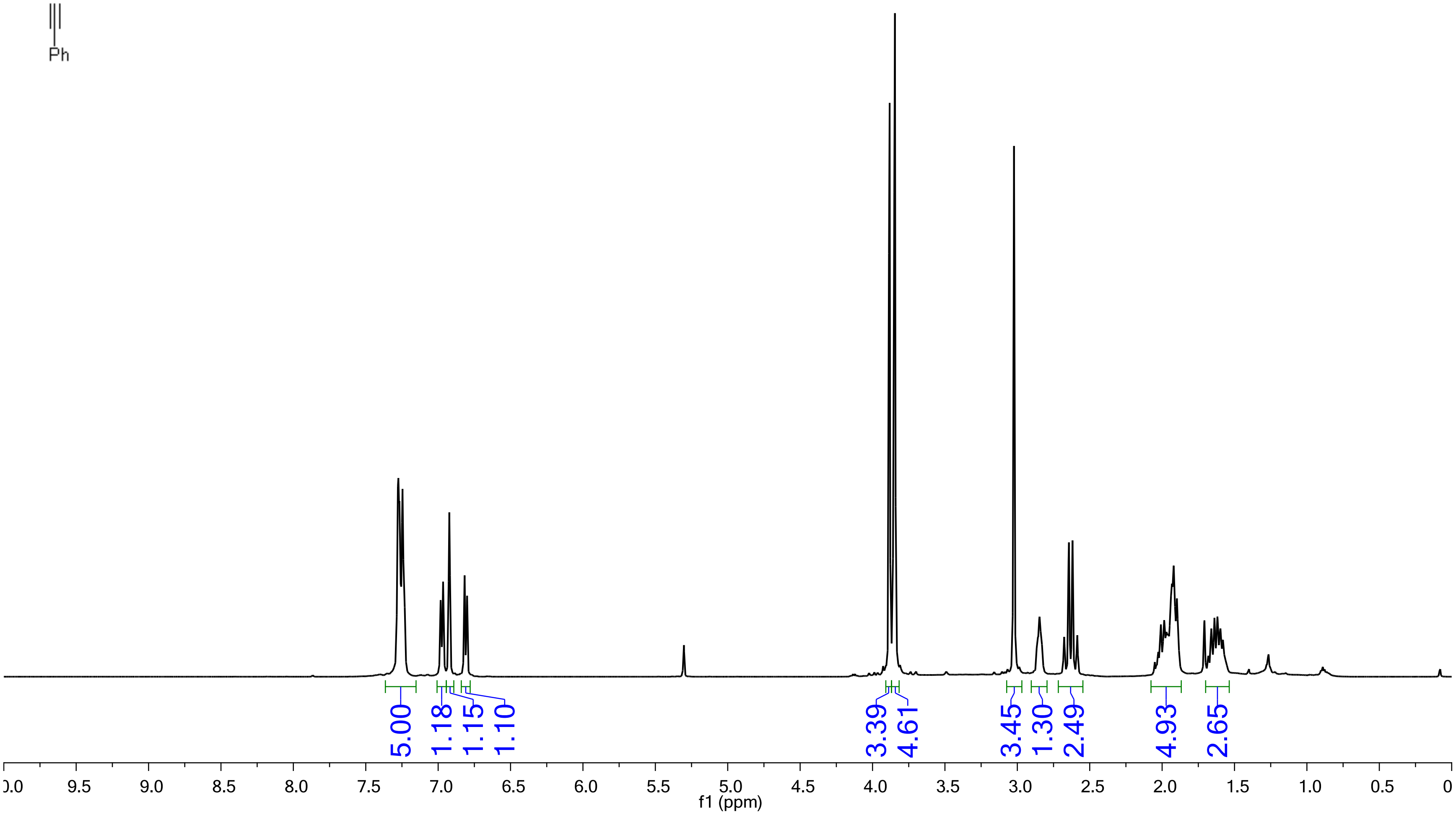

5.00
1.18
1.15
1.10
3.39
4.61
3.45
1.30
2.49
4.93
2.65
10.0
9.5
9.0
8.5
8.0
7.5
7.0
6.5
6.0
5.5
5.0
4.5
4.0
3.5
3.0
2.5
2.0
1.5
1.0
0.5
0
f1 (ppm)

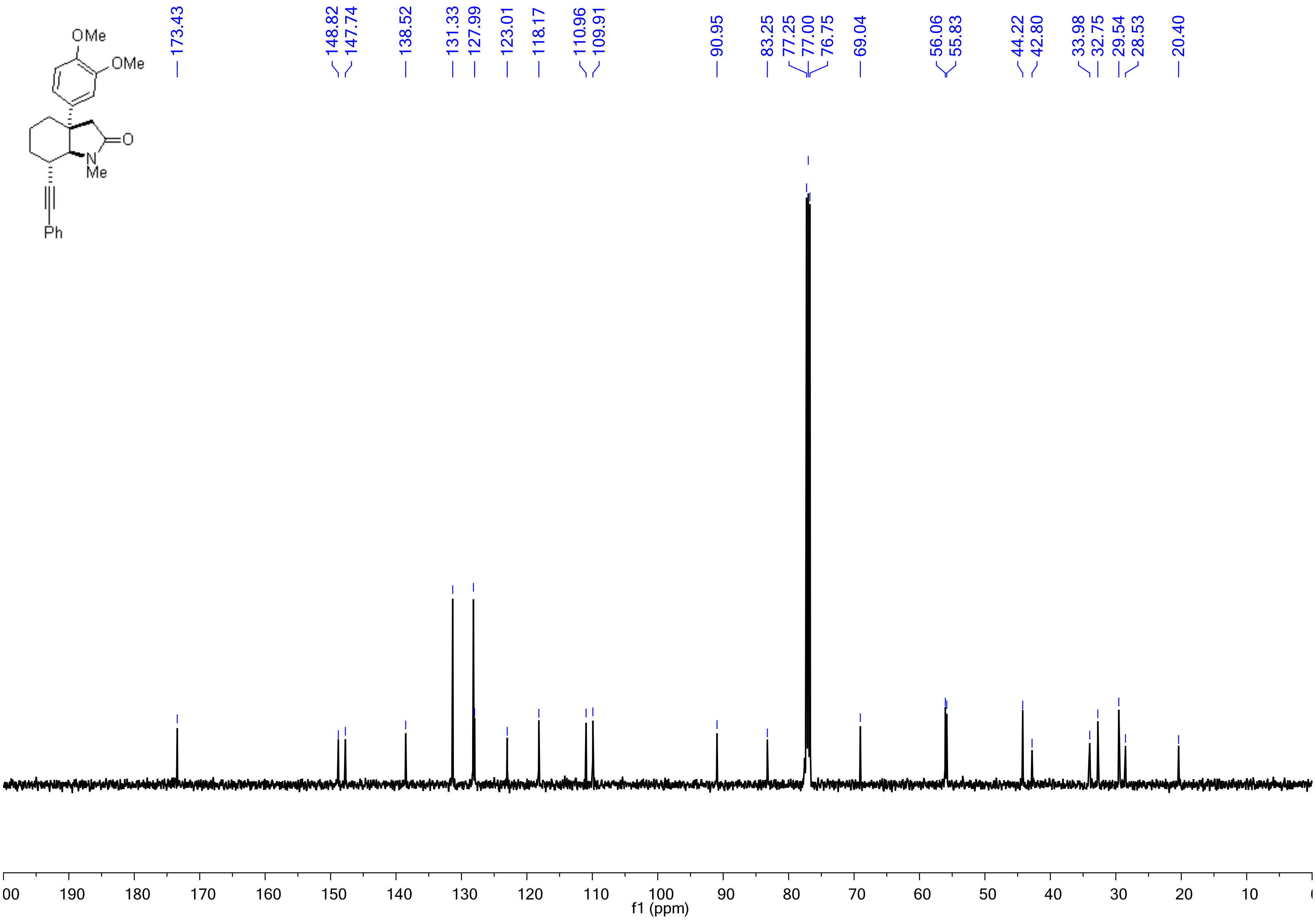
OMe
OMe
N
Me
O
Ph
173.43
148.82
147.74
138.52
131.33
127.99
123.01
118.17
110.96
109.91
90.95
83.25
77.25
77.00
76.75
69.04
56.06
55.83
44.22
42.80
33.98
32.75
29.54
28.53
20.40
f1 (ppm)

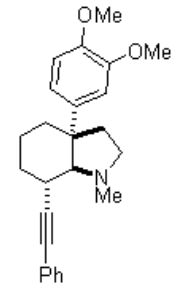
OMe
OMe
N
Me
Ph

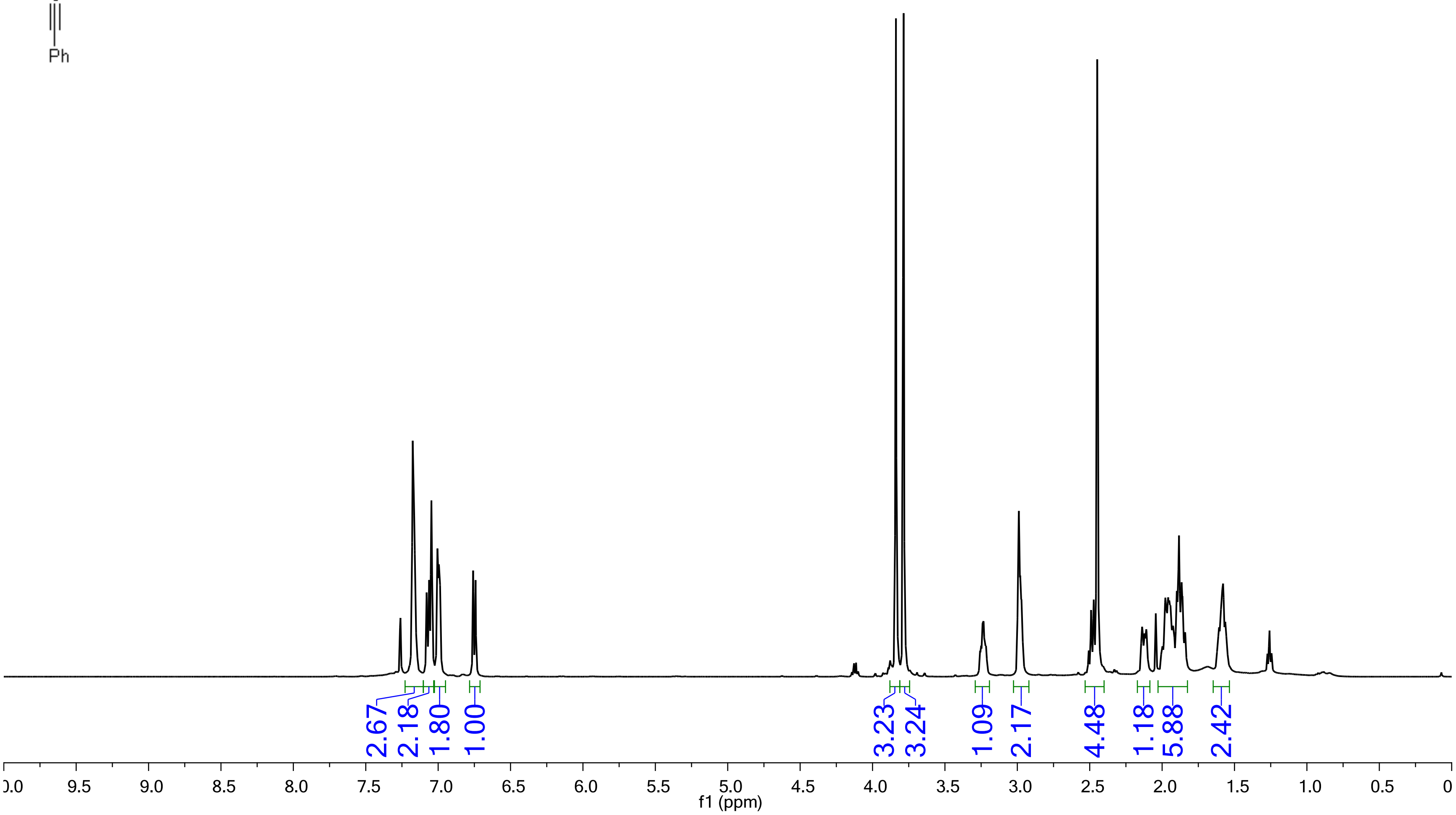
2.67
2.18
1.80
1.00
3.23
3.24
1.09
2.17
4.48
1.18
5.88
2.42
f1 (ppm)

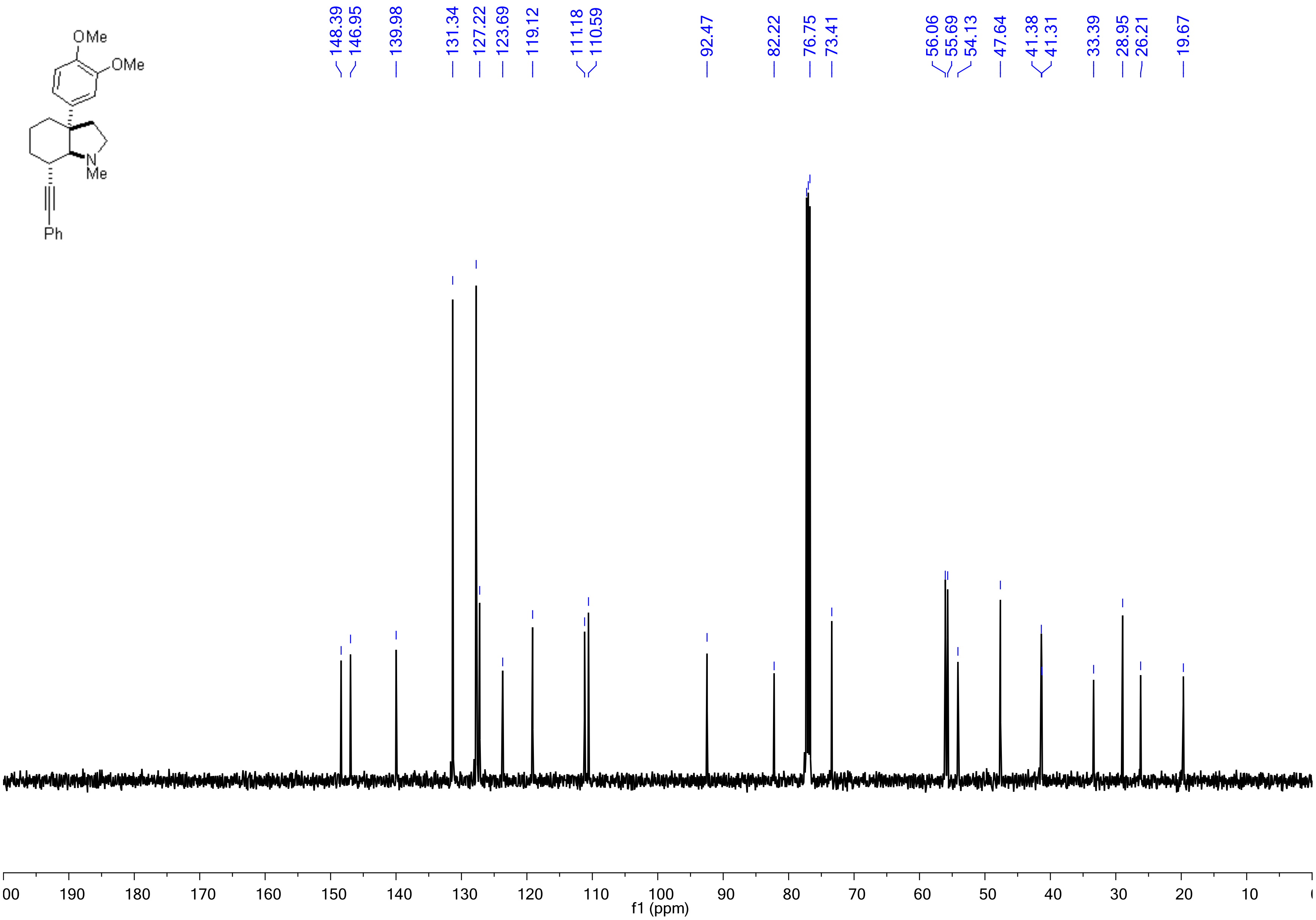
OMe
OMe
N
Me
Ph
148.39
146.95
139.98
131.34
127.22
123.69
119.12
111.18
110.59
92.47
82.22
76.75
73.41
56.06
55.69
54.13
47.64
41.38
41.31
33.39
28.95
26.21
19.67
190
180
170
160
150
140
130
120
110
100
90
80
70
60
50
40
30
20
10
f1 (ppm)

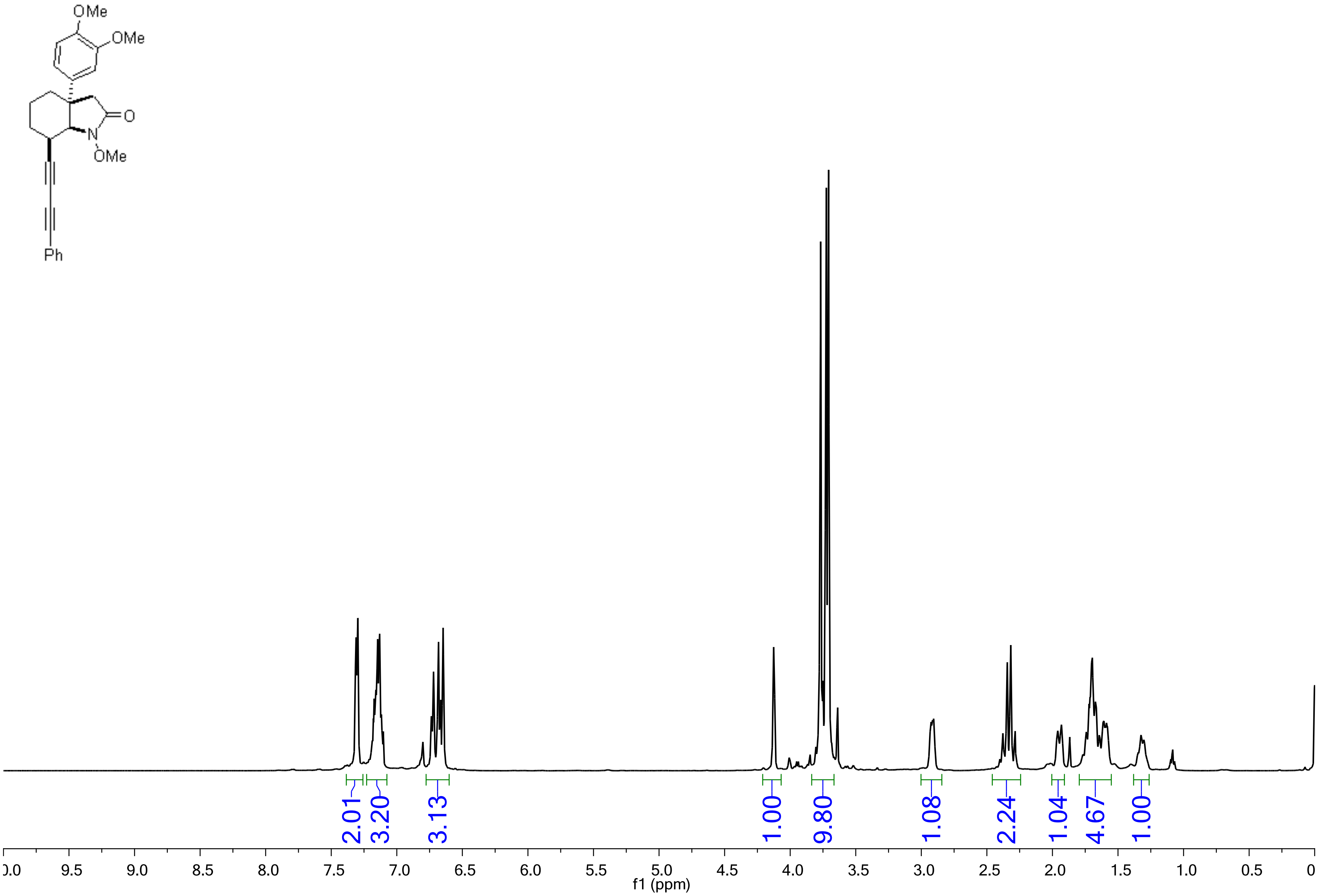
OMe
OMe
O
N
OMe
Ph
2.01
3.20
3.13
1.00
9.80
1.08
2.24
1.04
4.67
1.00
).0
9.5
9.0
8.5
8.0
7.5
7.0
6.5
6.0
5.5
5.0
4.5
4.0
3.5
3.0
2.5
2.0
1.5
1.0
0.5
0
f1 (ppm)

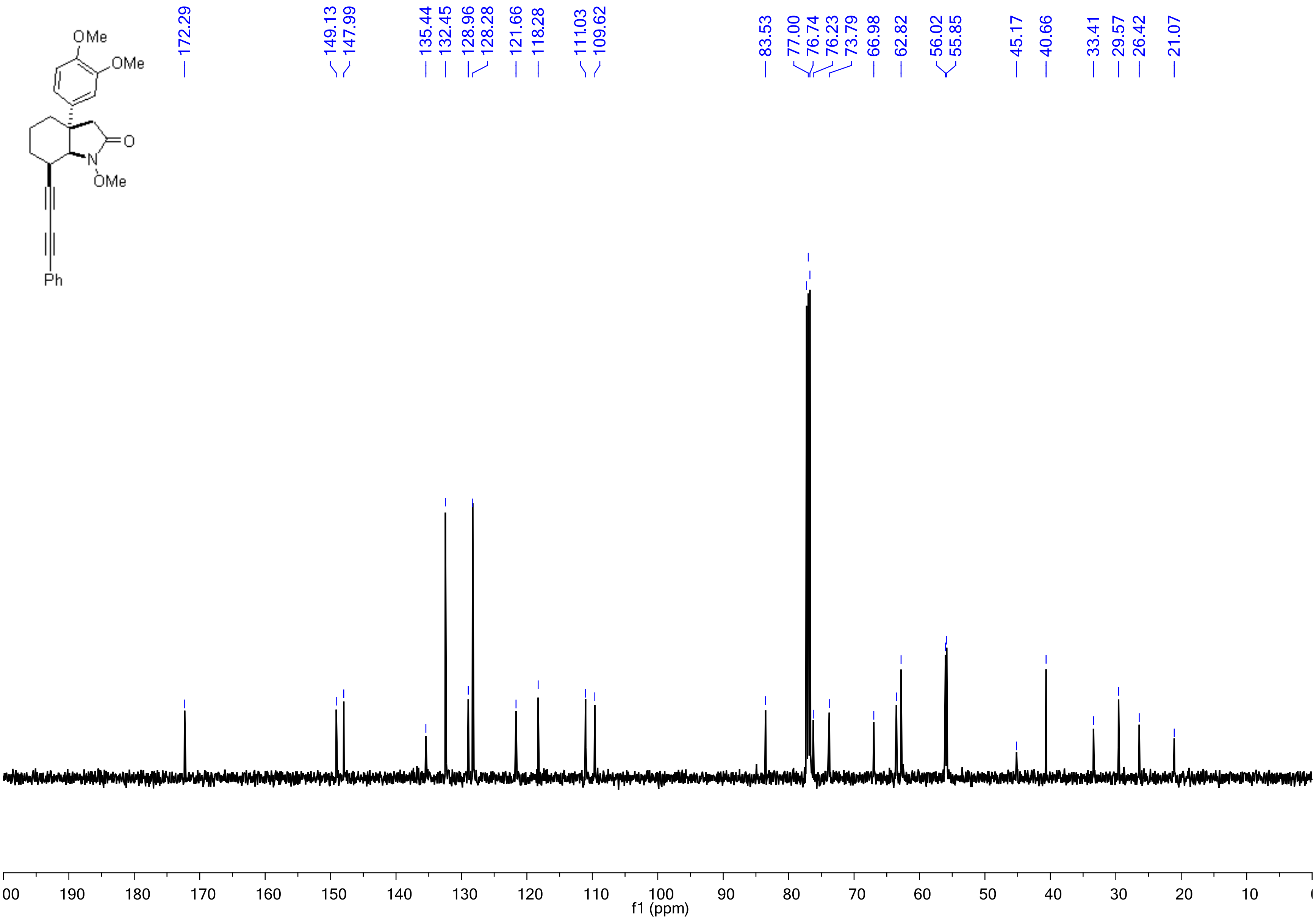
OMe
OMe
O
N
OMe
Ph
172.29
149.13
147.99
135.44
132.45
128.96
128.28
121.66
118.28
111.03
109.62
83.53
77.00
76.74
76.23
73.79
66.98
62.82
56.02
55.85
45.17
40.66
33.41
29.57
26.42
21.07
190
180
170
160
150
140
130
120
110
100
90
80
70
60
50
40
30
20
10
f1 (ppm)

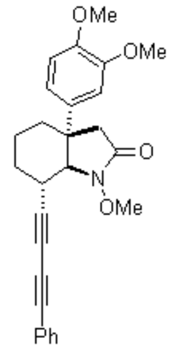
OMe
OMe
O
N
OMe
Ph

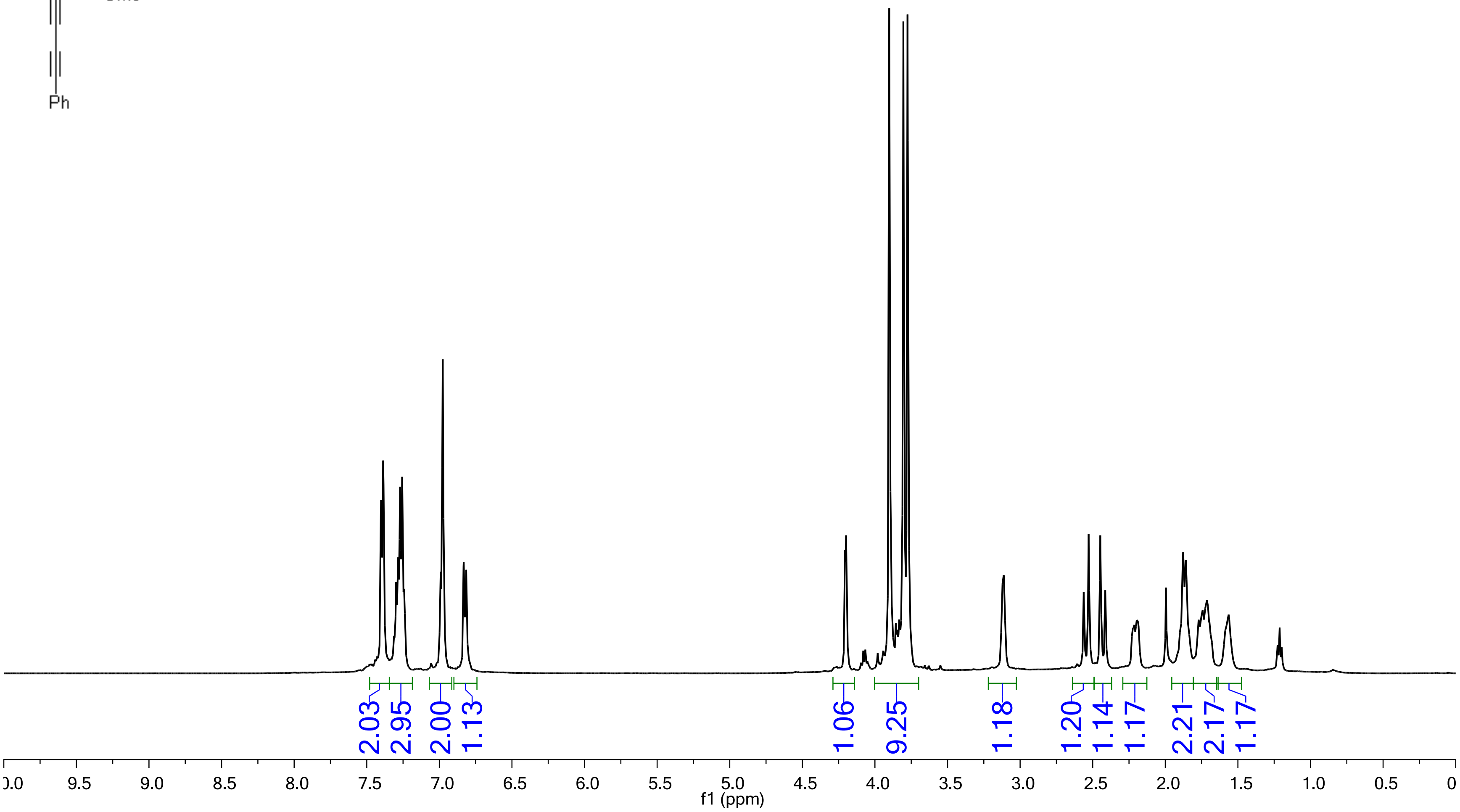
2.03
2.95
2.00
1.13
1.06
9.25
1.18
1.20
1.14
1.17
2.21
2.17
1.17
f1 (ppm)

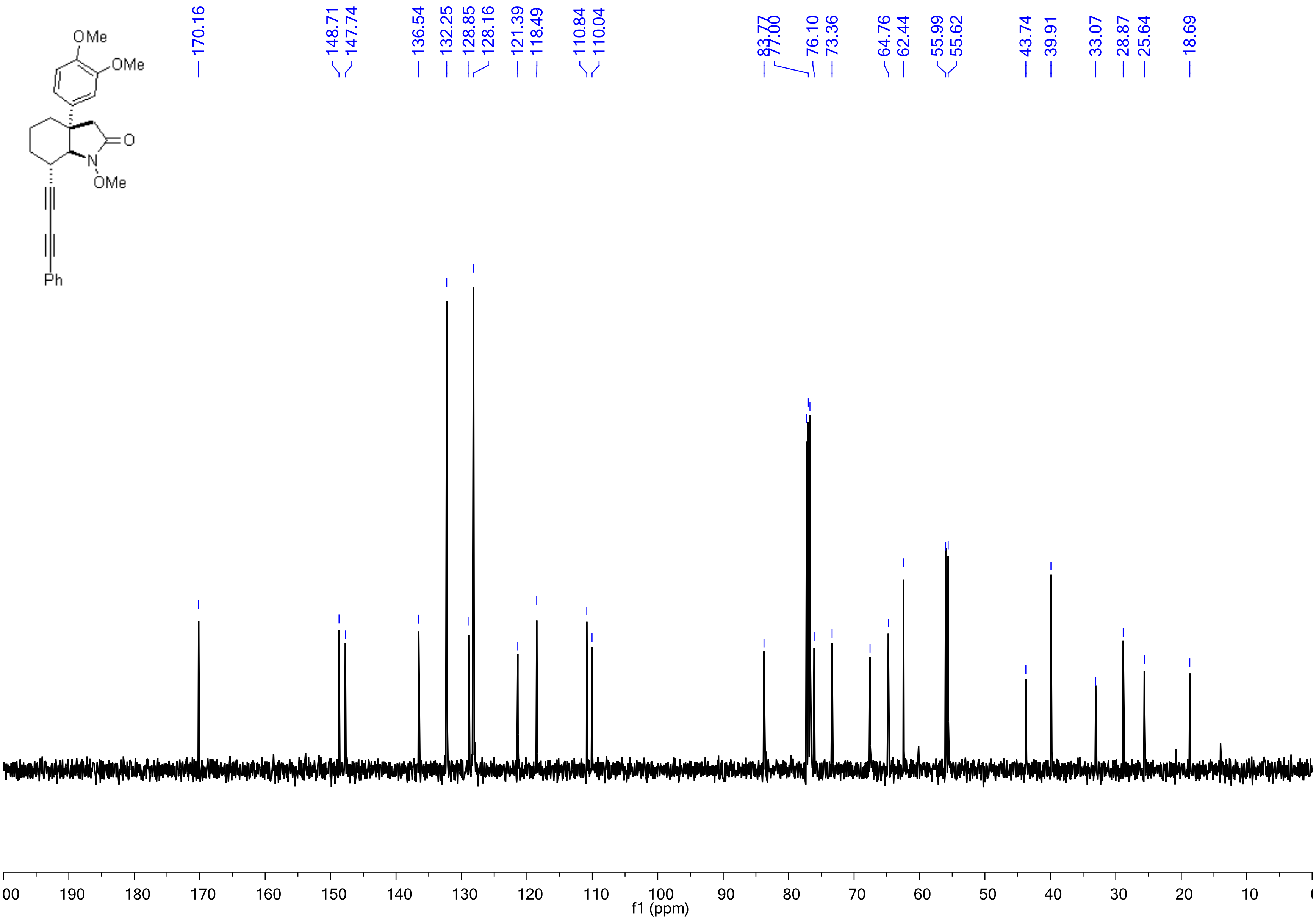
OMe
OMe
O
N
OMe
Ph
170.16
148.71
147.74
136.54
132.25
128.85
128.16
121.39
118.49
110.84
110.04
83.77
77.00
76.10
73.36
64.76
62.44
55.99
55.62
43.74
39.91
33.07
28.87
25.64
18.69
00
190
180
170
160
150
140
130
120
110
100
90
80
70
60
50
40
30
20
10
f1 (ppm)

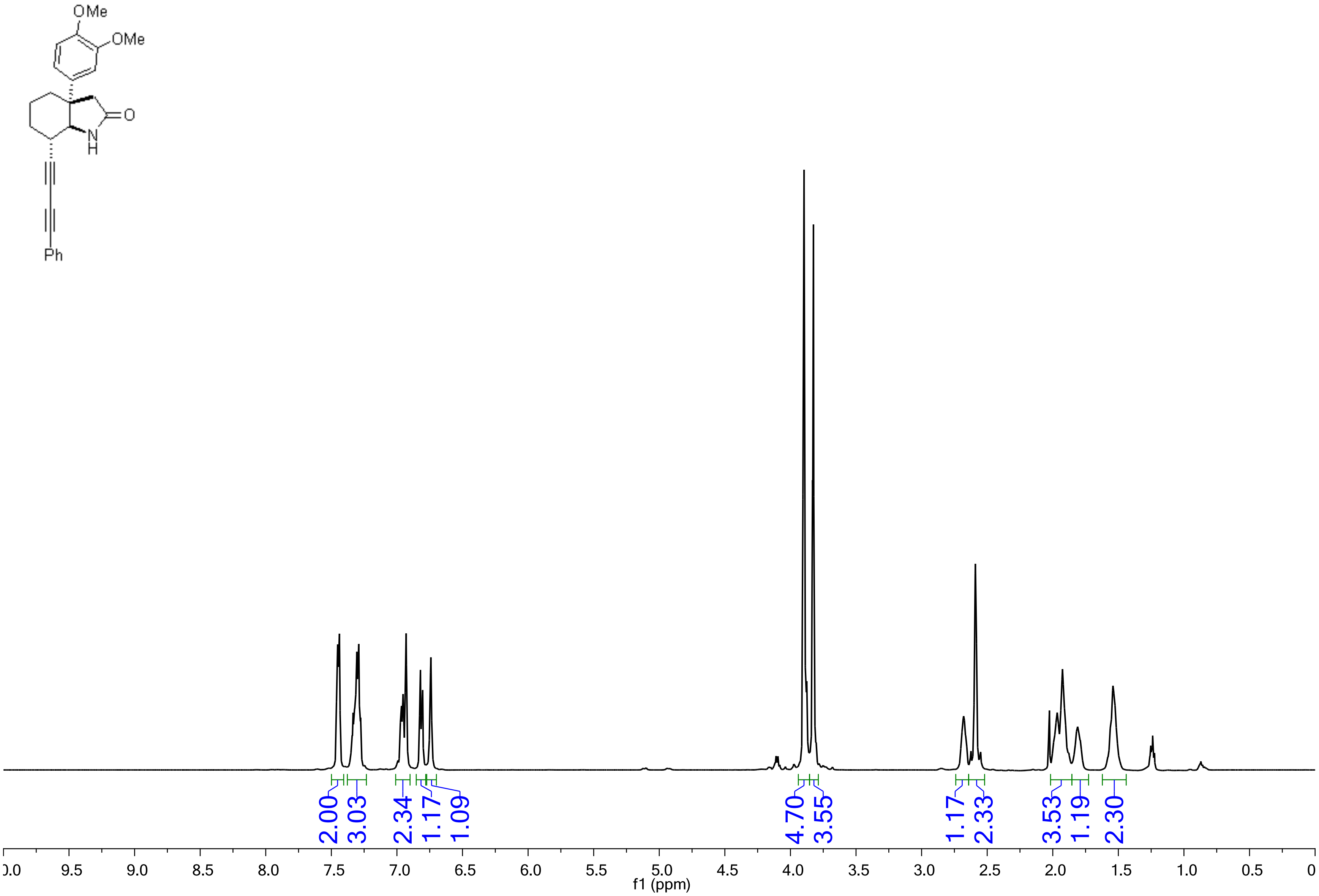
OMe
OMe
O
N
H
Ph
2.00
3.03
2.34
1.17
1.09
4.70
3.55
1.17
2.33
3.53
1.19
2.30
9.5 9.0 8.5 8.0 7.5 7.0 6.5 6.0 5.5 5.0 4.5 4.0 3.5 3.0 2.5 2.0 1.5 1.0 0.5 0
f1 (ppm)

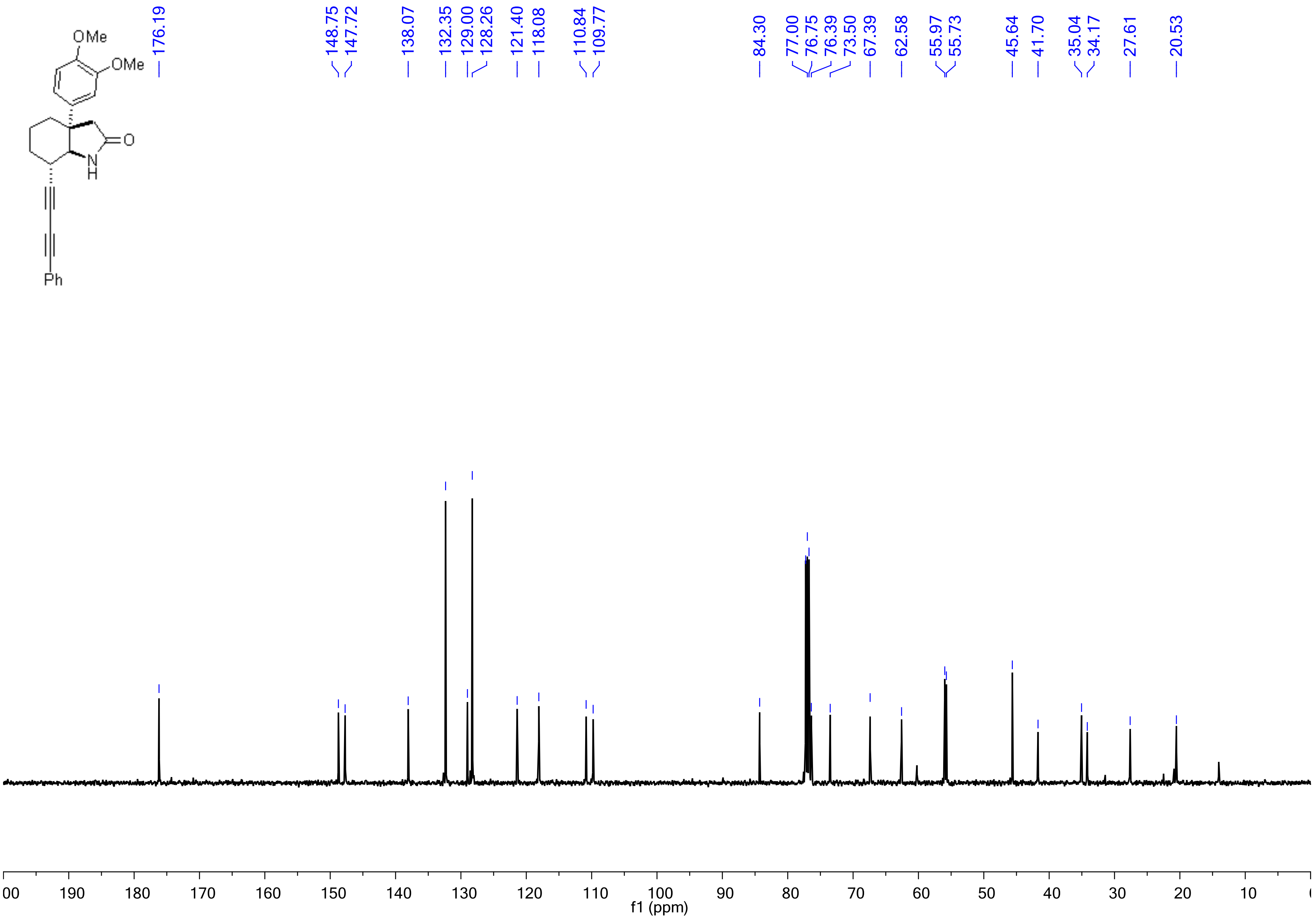
OMe
OMe
O
N
H
Ph
176.19
148.75
147.72
138.07
132.35
129.00
128.26
121.40
118.08
110.84
109.77
84.30
77.00
76.75
76.39
73.50
67.39
62.58
55.97
55.73
45.64
41.70
35.04
34.17
27.61
20.53
f1 (ppm)

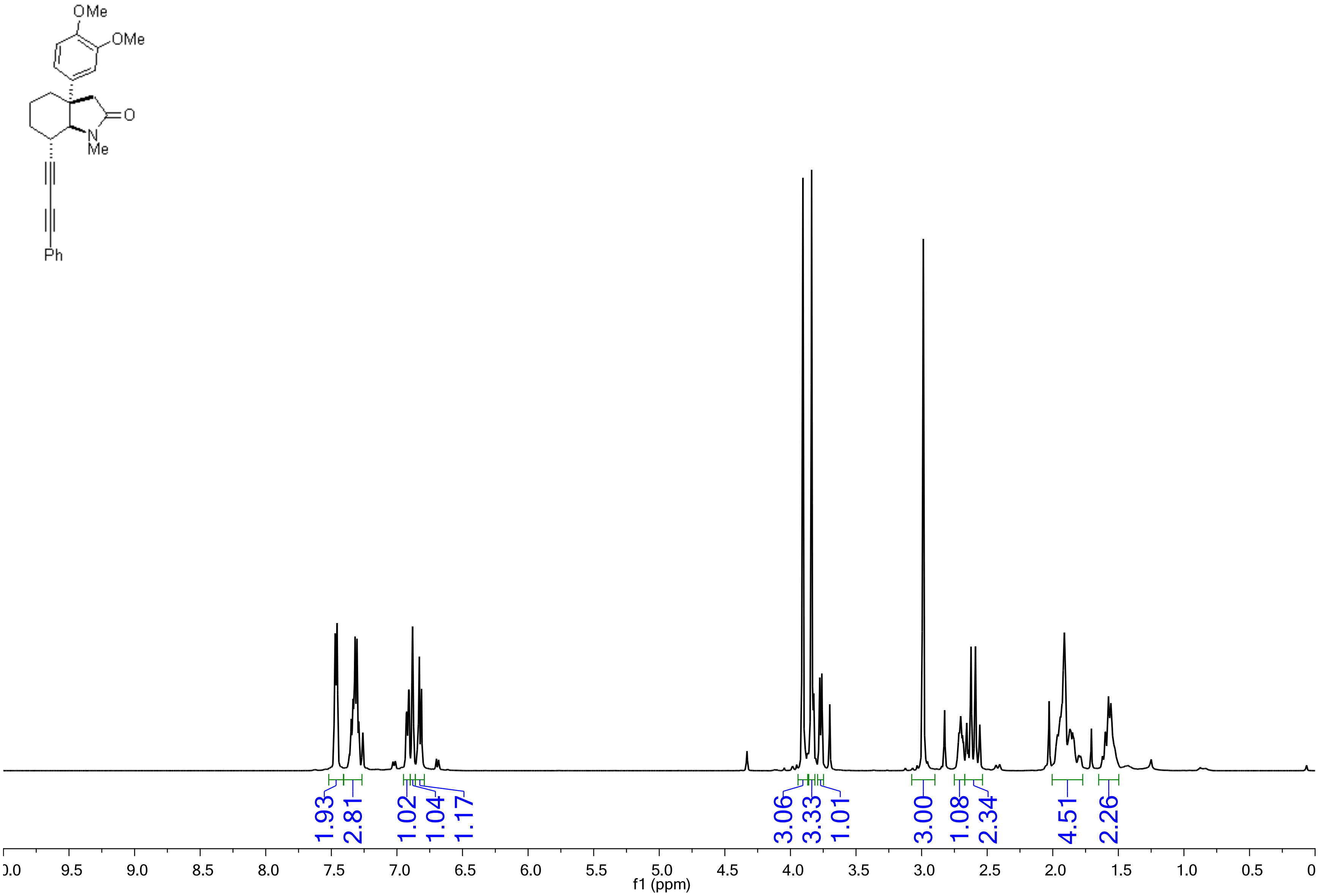
OMe
OMe
O
N
Me
Ph
1.93
2.81
1.02
1.04
1.17
3.06
3.33
1.01
3.00
1.08
2.34
4.51
2.26
10.0
9.5
9.0
8.5
8.0
7.5
7.0
6.5
6.0
5.5
5.0
4.5
4.0
3.5
3.0
2.5
2.0
1.5
1.0
0.5
0
f1 (ppm)

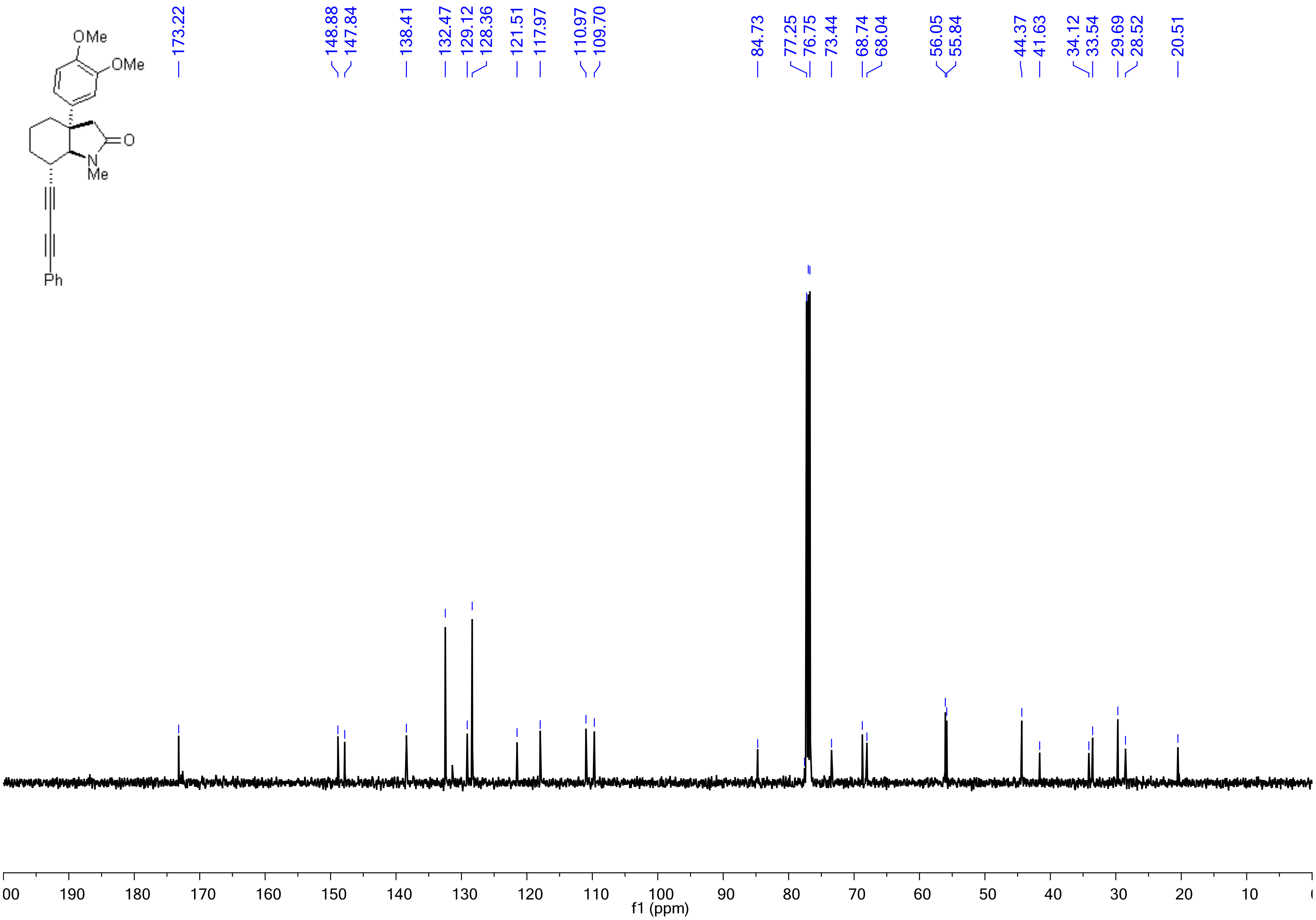

OMe
OMe
O
N
Me
Ph
173.22
148.88
147.84
138.41
132.47
129.12
128.36
121.51
117.97
110.97
109.70
84.73
77.25
76.75
73.44
68.74
68.04
56.05
55.84
44.37
41.63
34.12
33.54
29.69
28.52
20.51
00
190
180
170
160
150
140
130
120
110
100
90
80
70
60
50
40
30
20
10
f1 (ppm)

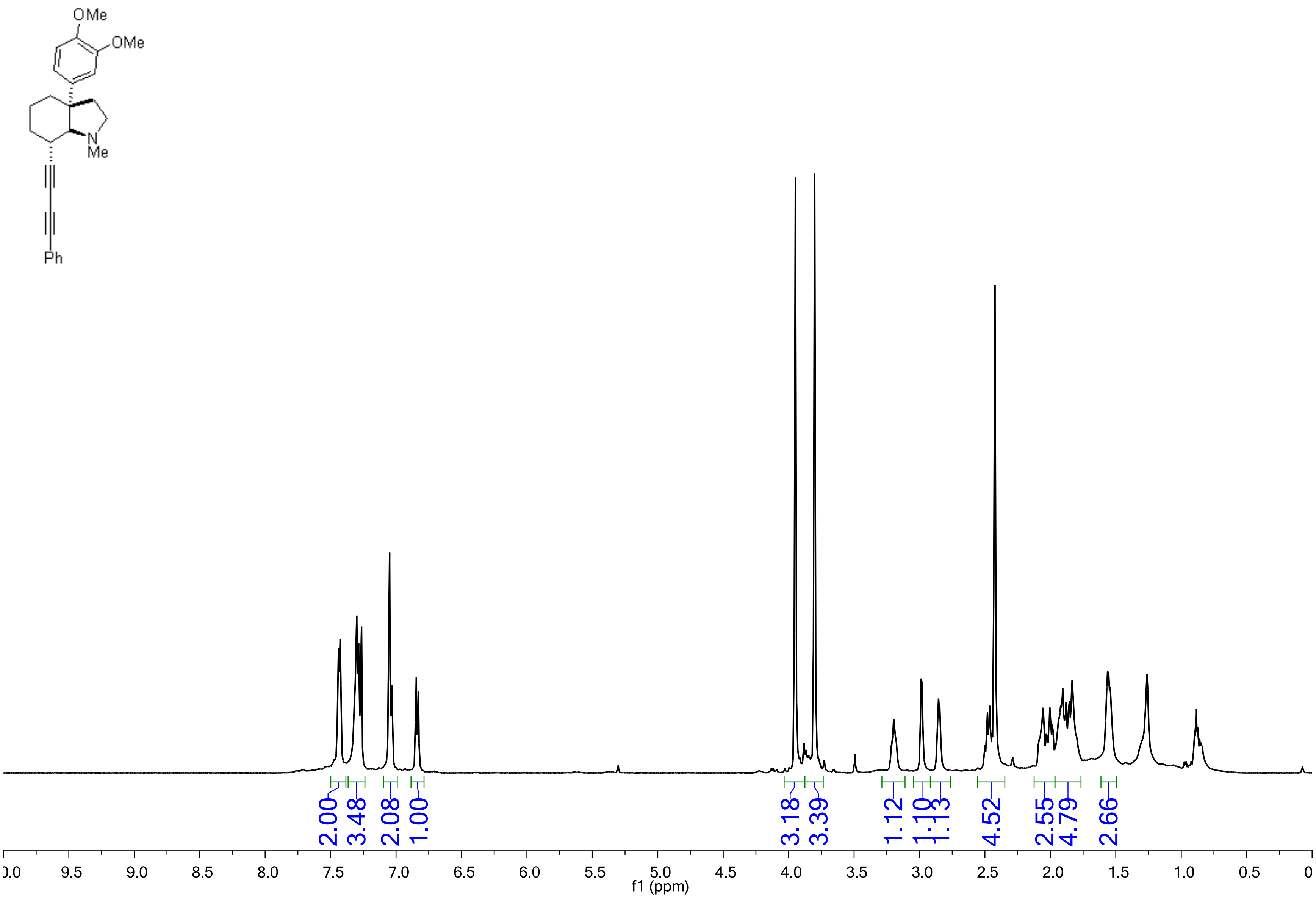

OMe
OMe
N
Me
Ph
2.00
3.48
2.08
1.00
3.18
3.39
1.12
1.10
1.13
4.52
2.55
4.79
2.66
f1 (ppm)

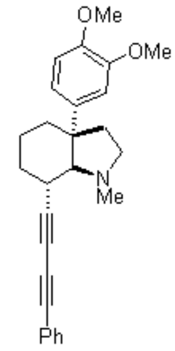
OMe
OMe
N
Me
Ph

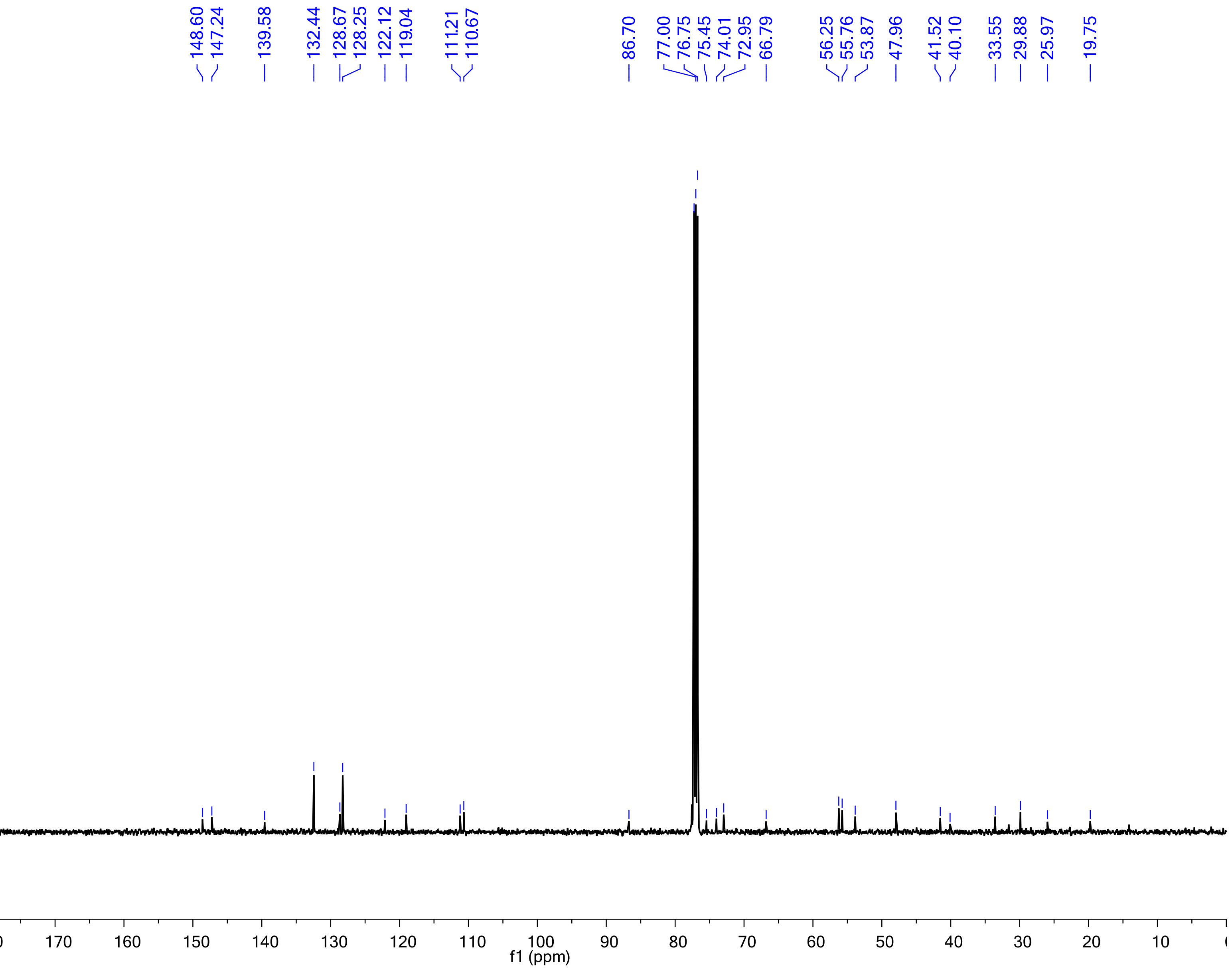
148.60
147.24
139.58
132.44
128.67
128.25
122.12
119.04
111.21
110.67
86.70
77.00
76.75
75.45
74.01
72.95
66.79
56.25
55.76
53.87
47.96
41.52
40.10
33.55
29.88
25.97
19.75
00
190
180
170
160
150
140
130
120
110
100
90
80
70
60
50
40
30
20
10
f1 (ppm)

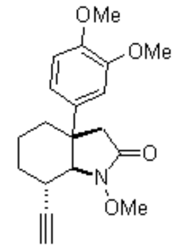
OMe
OMe
O
N
OMe

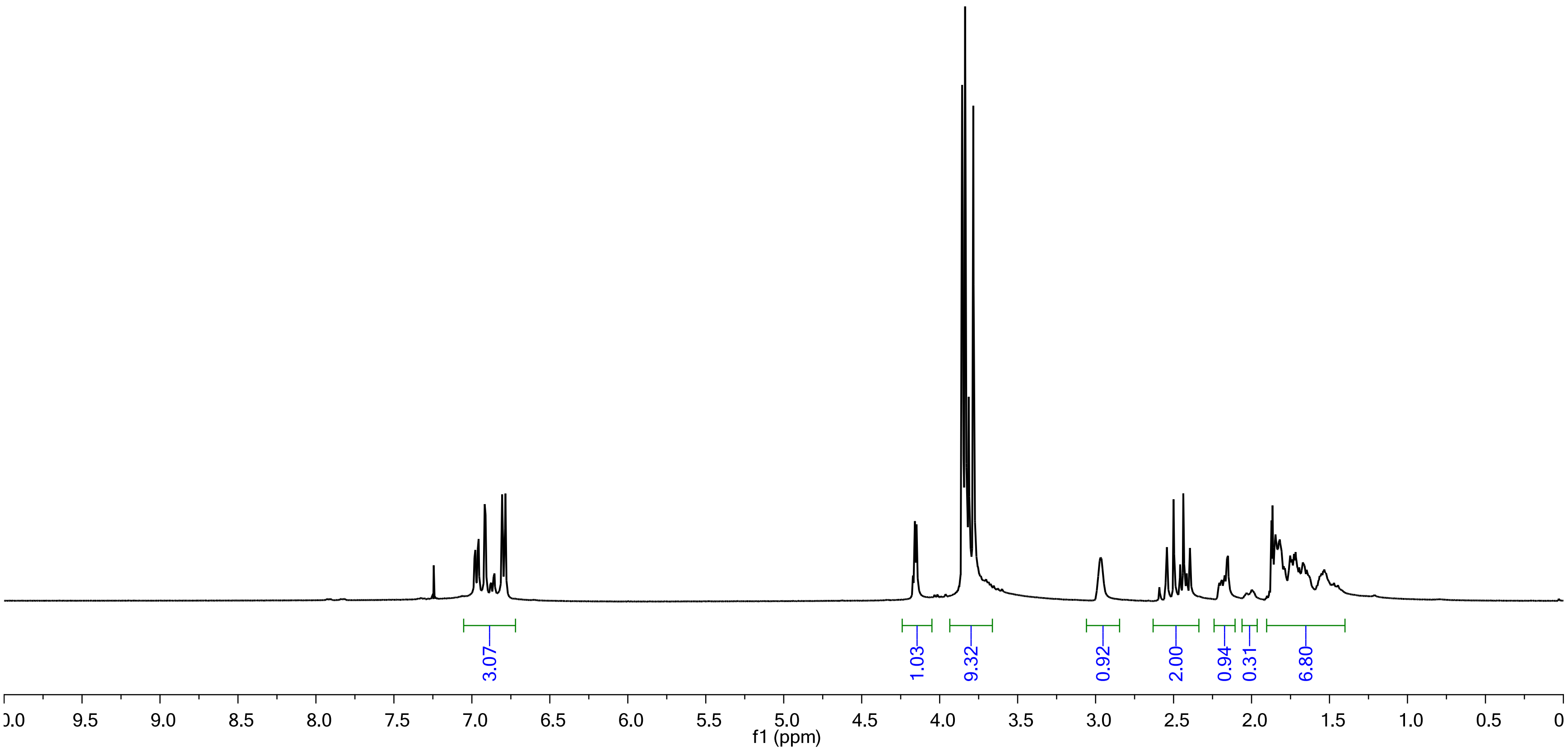
3.07
1.03
9.32
0.92
2.00
0.94
0.31
6.80
10.0
9.5
9.0
8.5
8.0
7.5
7.0
6.5
6.0
5.5
5.0
4.5
4.0
3.5
3.0
2.5
2.0
1.5
1.0
0.5
0
f1 (ppm)

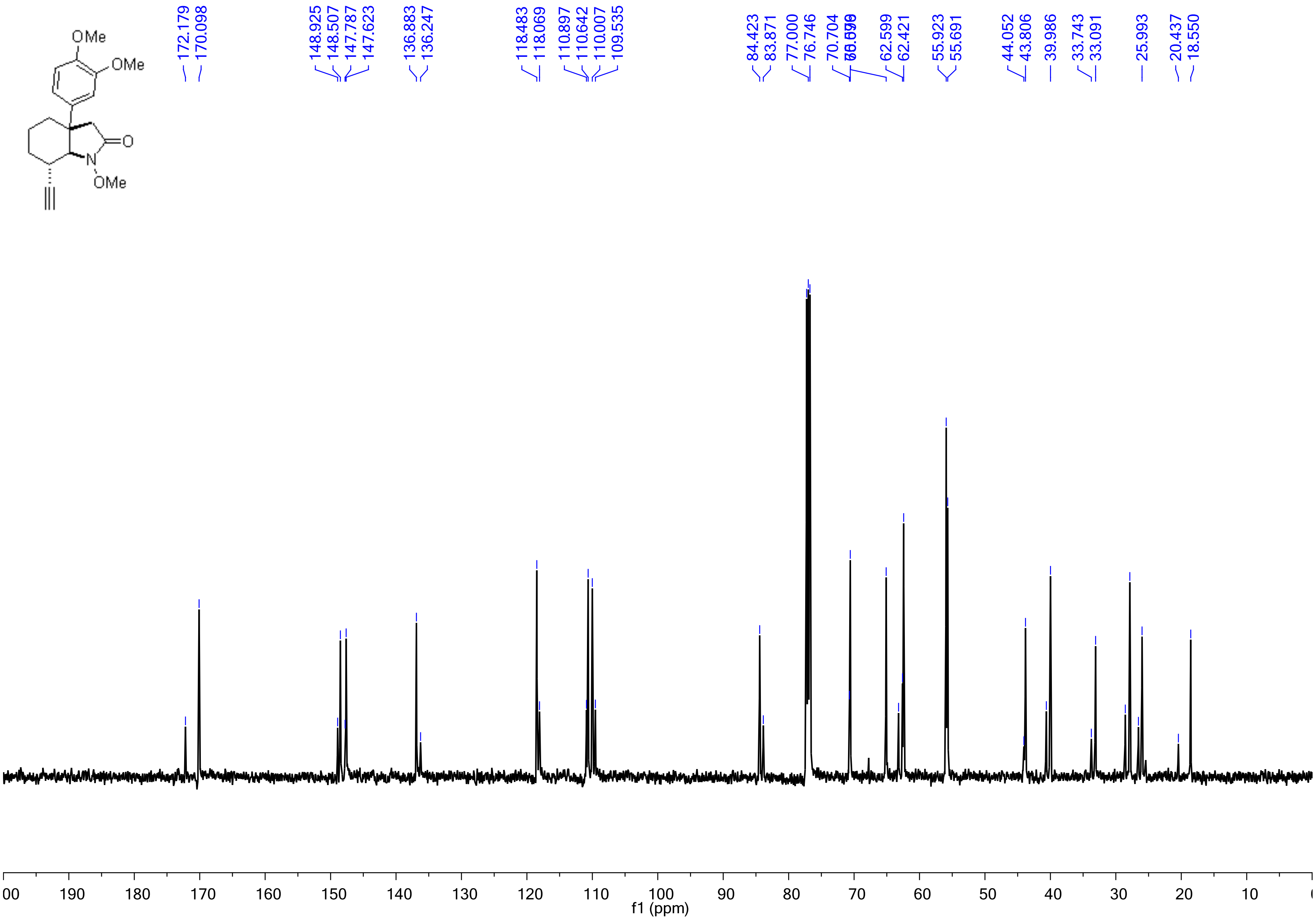
OMe
OMe
O
N
OMe
172.179
170.098
148.925
148.507
147.787
147.623
136.883
136.247
118.483
118.069
110.897
110.642
110.007
109.535
84.423
83.871
77.000
76.746
70.704
62.599
62.421
55.923
55.691
44.052
43.806
39.986
33.743
33.091
25.993
20.437
18.550
190
180
170
160
150
140
130
120
110
100
90
80
70
60
50
40
30
20
10
f1 (ppm)

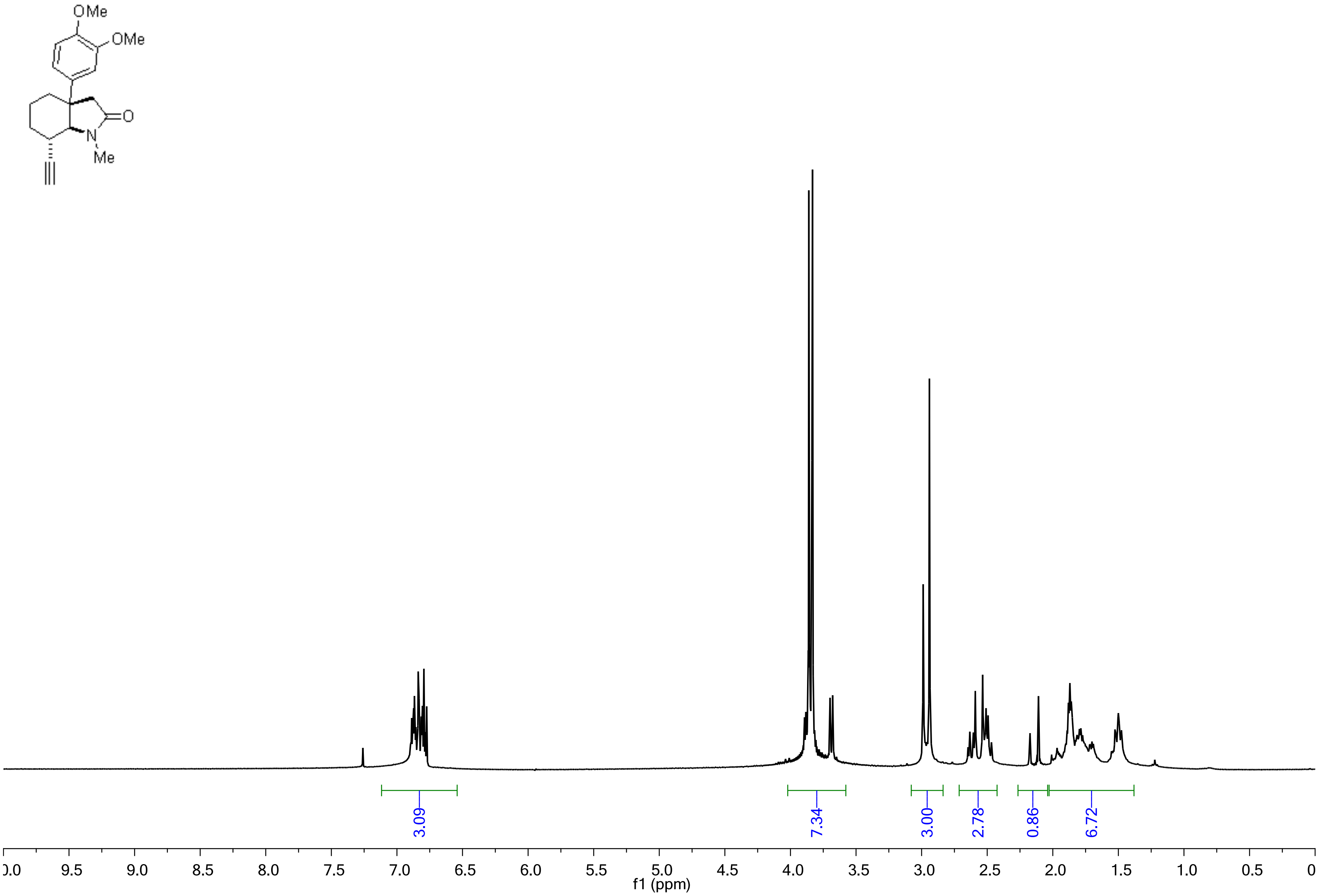
OMe
OMe
O
N
Me
3.09
7.34
3.00
2.78
0.86
6.72
10.0
9.5
9.0
8.5
8.0
7.5
7.0
6.5
6.0
5.5
5.0
4.5
4.0
3.5
3.0
2.5
2.0
1.5
1.0
0.5
0
f1 (ppm)

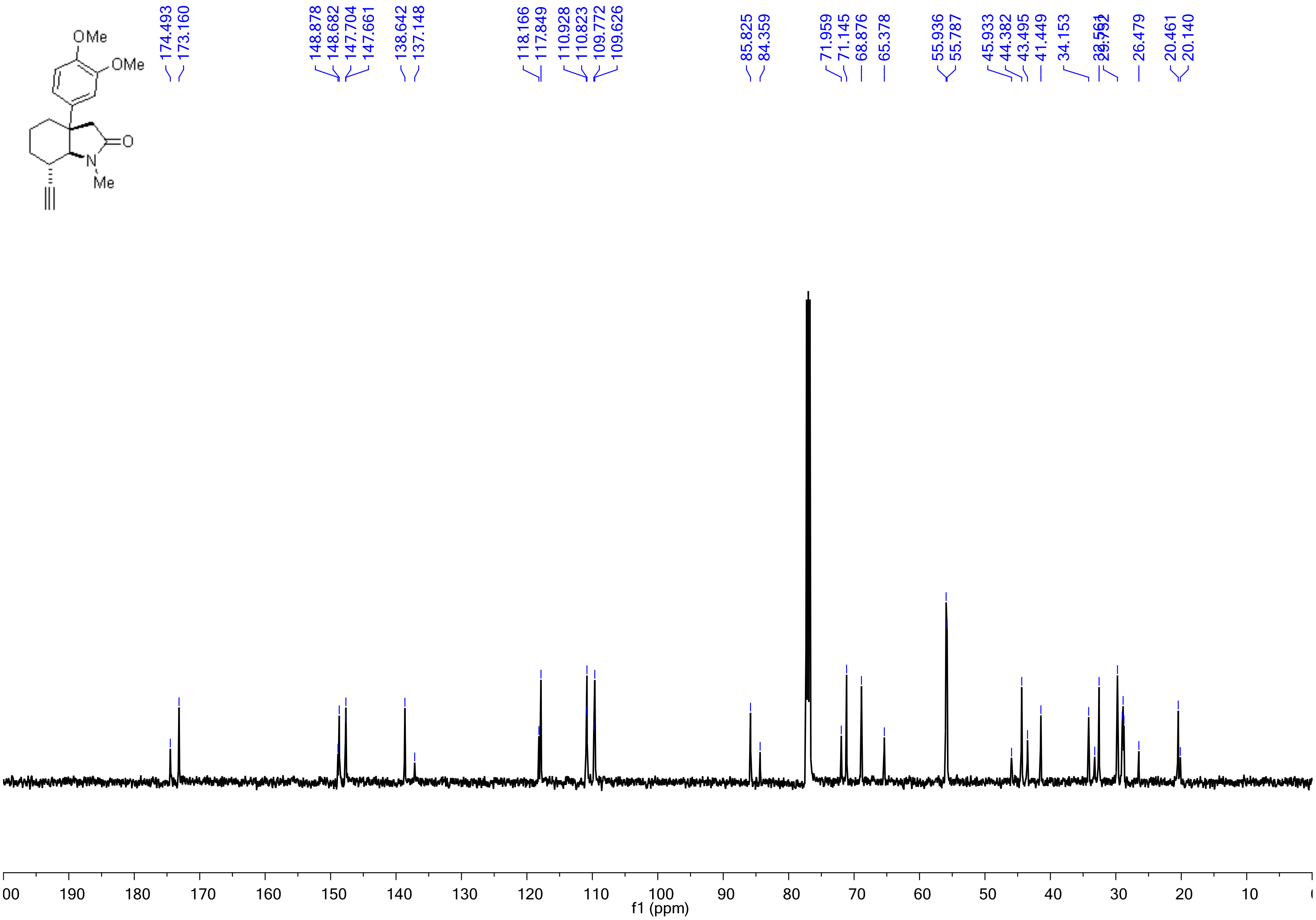
OMe
OMe
N
Me
O
174.493
173.160
148.878
148.682
147.704
147.661
138.642
137.148
118.166
117.849
110.928
110.823
109.772
109.626
85.825
84.359
71.959
71.145
68.876
65.378
55.936
55.787
45.933
44.382
43.495
41.449
34.153
26.479
20.461
20.140
190
180
170
160
150
140
130
120
110
100
90
80
70
60
50
40
30
20
10
f1 (ppm)

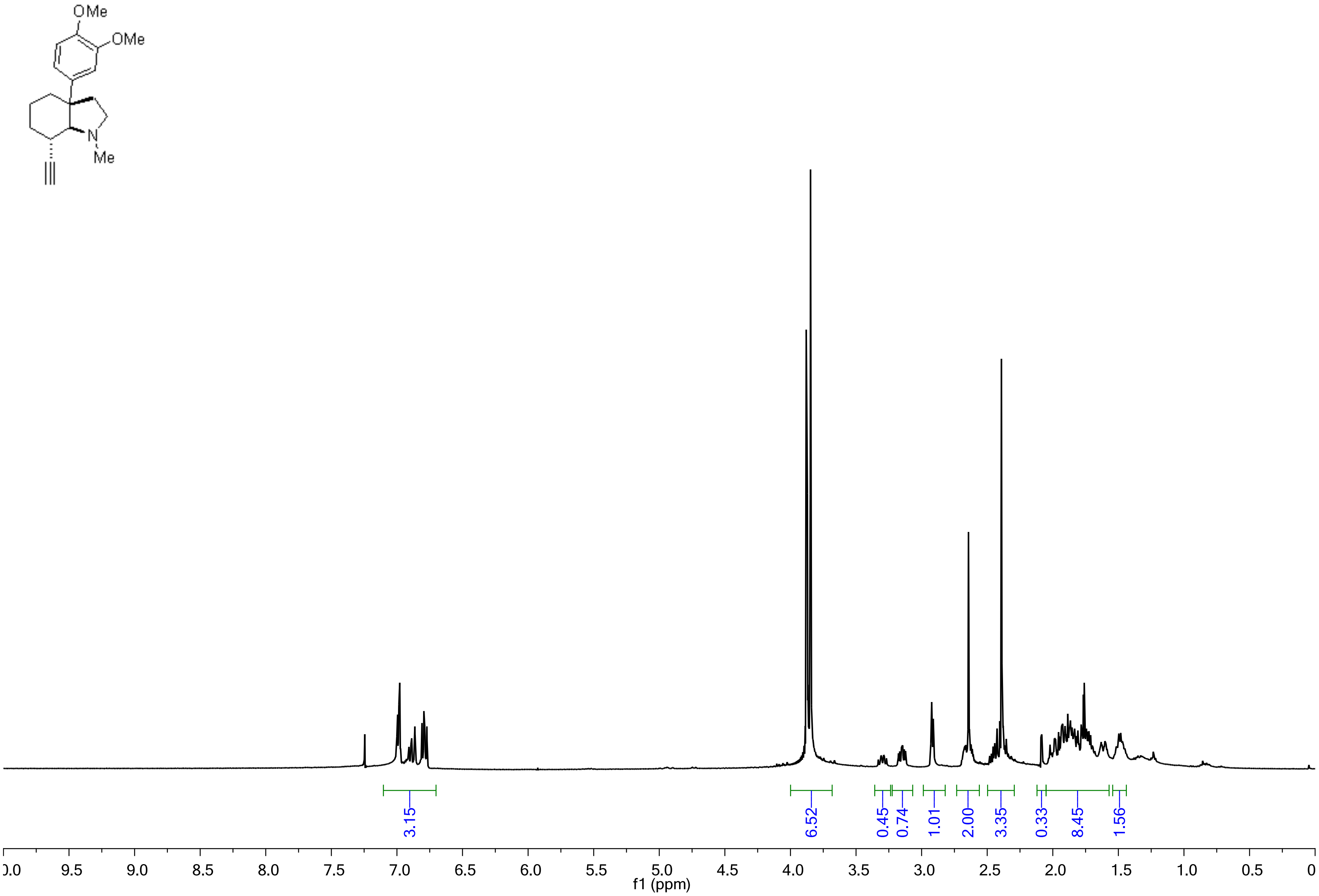
OMe
OMe
N
Me
3.15
6.52
0.45
0.74
1.01
2.00
3.35
0.33
8.45
1.56
9.5 9.0 8.5 8.0 7.5 7.0 6.5 6.0 5.5 5.0 4.5 4.0 3.5 3.0 2.5 2.0 1.5 1.0 0.5 0
f1 (ppm)

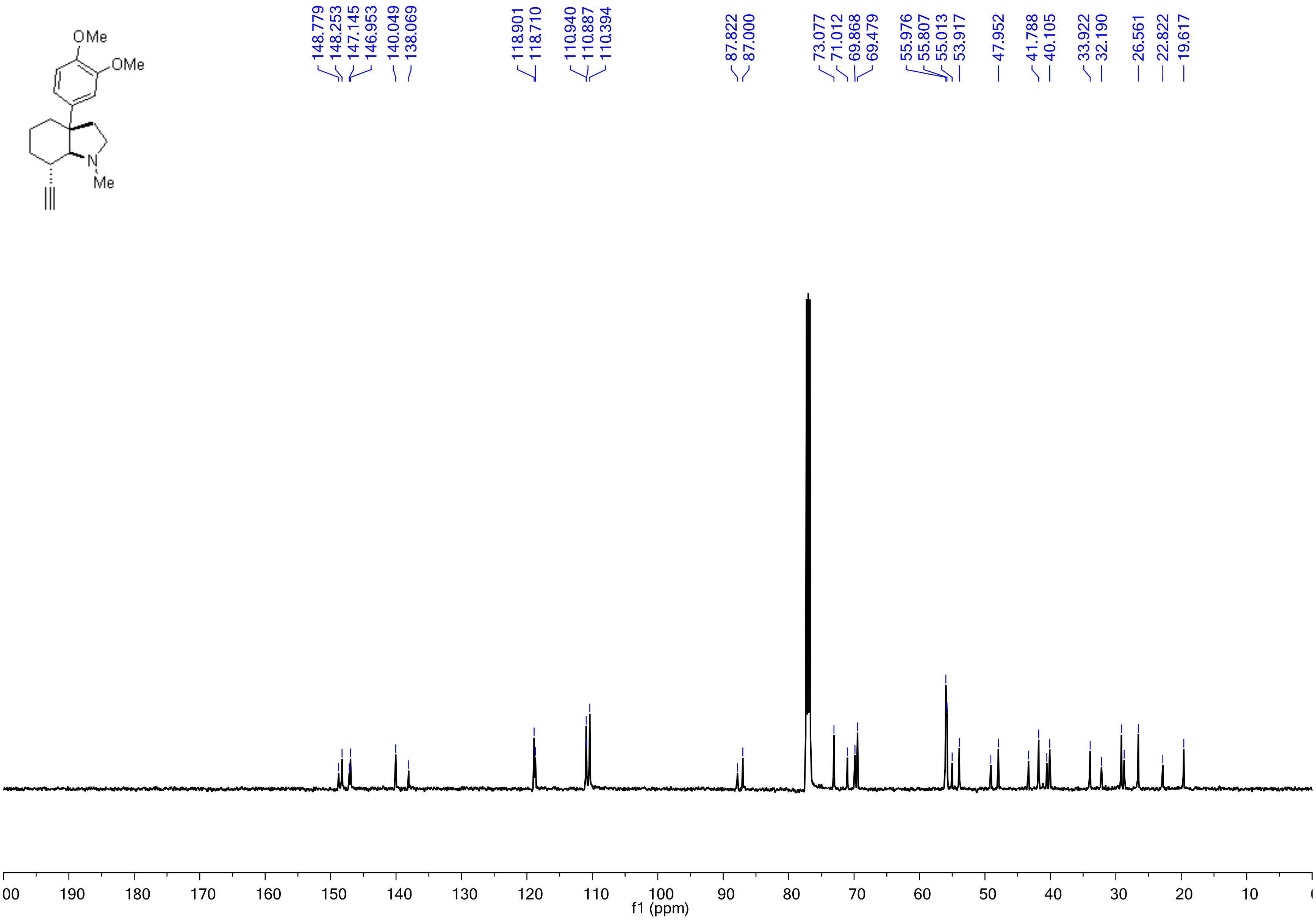
OMe
OMe
N
Me
148.779
148.253
147.145
146.953
140.049
138.069
118.901
118.710
110.940
110.887
110.394
87.822
87.000
73.077
71.012
69.868
69.479
55.976
55.807
55.013
53.917
47.952
41.788
40.105
33.922
32.190
26.561
22.822
19.617
00 190 180 170 160 150 140 130 120 110 100 90 80 70 60 50 40 30 20 10
f1 (ppm)

XI. X-Ray Data

1. Crystal Structure Report for rds667 (3pa-major)

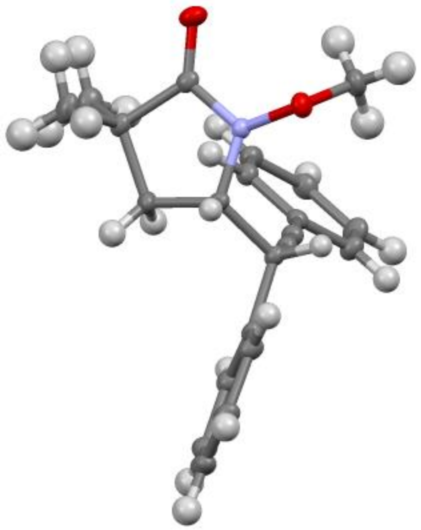

A colorless block-like specimen of $C_{22}H_{23}NO_2$, approximate dimensions 0.092 mm x 0.251 mm x 0.387 mm, was used for the X-ray crystallographic analysis. The X-ray intensity data were measured.
The total exposure time was 9.52 hours. The frames were integrated with the Bruker SAINT software package using a narrow-frame algorithm. The integration of the data using a triclinic unit cell yielded a total of 27055 reflections to a maximum θ angle of 29.75° (0.72 Å resolution), of which 5179 were independent (average redundancy 5.224, completeness = 99.6%, R_{int} = 3.37%, R_{sig} = 3.05%) and 3980 (76.85%) were greater than $2\sigma(F^2)$. The final cell constants of a = 9.3352(7) Å, b = 10.2282(9) Å, c = 11.6109(9) Å, α = 70.977(2)°, β = 69.277(3)°, γ = 63.872(2)°, volume = 911.65(13) $Å^3$, are based upon the refinement of the XYZ-centroids of 7324 reflections above 20 σ(I) with 4.528° < 2θ < 59.04°. Data were corrected for absorption effects using the Multi-Scan method (SADABS). The ratio of minimum to maximum apparent transmission was 0.966. The calculated minimum and maximum transmission coefficients (based on crystal size) are 0.9710 and 0.9930. The final anisotropic full-matrix least-squares refinement on F^2 with 229 variables converged at R1 = 4.22%, for the observed data and wR2 = 10.38% for all data. The goodness-of-fit was 1.037. The largest peak in the final difference electron density synthesis was 0.350 $e^-/Å^3$ and the largest hole was -0.205 $e^-/Å^3$ with an RMS deviation of 0.042 $e^-/Å^3$. On the basis of the final model, the calculated density was 1.215 g/cm^3 and F(000), 356 e^-.

Sample and crystal data for rds667.

**Identification code**	rds667	
**Chemical formula**	$C_{22}H_{23}NO_2$	
**Formula weight**	333.41 g/mol	
**Temperature**	121(2) K	
**Wavelength**	0.71073 Å	
**Crystal size**	0.092 x 0.251 x 0.387 mm	
**Crystal habit**	colorless block	
**Crystal system**	triclinic	
**Space group**	P -1	
**Unit cell dimensions**	a = 9.3352(7) Å	α = 70.977(2)°
	b = 10.2282(9) Å	β = 69.277(3)°
	c = 11.6109(9) Å	γ = 63.872(2)°

**Volume**	911.65(13) Å^3
**Z**	2
**Density (calculated)**	1.215 g/cm^3
**Absorption coefficient**	0.077 mm^{-1}
**F(000)**	356

Data collection and structure refinement for rds667.

**Theta range for data collection**	1.92 to 29.75°	
**Index ranges**	-13<=h<=12, -14<=k<=14, -16<=l<=16	
**Reflections collected**	27055	
**Independent reflections**	5179 [R(int) = 0.0337]	
**Coverage of independent reflections**	99.6%	
**Absorption correction**	Multi-Scan	
**Max. and min. transmission**	0.9930 and 0.9710	
**Refinement method**	Full-matrix least-squares on F^2	
**Refinement program**	SHELXL-2014/7 (Sheldrick, 2014)	
**Function minimized**	Σ w(F$_o^2$ - F$_c^2$)^2	
**Data / restraints / parameters**	5179 / 0 / 229	
**Goodness-of-fit on F^2**	1.037	
**Final R indices**	3980 data; I>2σ(I)	R1 = 0.0422, wR2 = 0.0944
	all data	R1 = 0.0627, wR2 = 0.1038
**Weighting scheme**	w=1/[σ^2(F$_o^2$)+(0.0421P)^2+0.2729P]<br>where P=(F$_o^2$+2F$_c^2$)/3	
**Largest diff. peak and hole**	0.350 and -0.205 eÅ^{-3}	
**R.M.S. deviation from mean**	0.042 eÅ^{-3}	

Atomic coordinates and equivalent isotropic atomic displacement parameters (Å^2) for rds667.

U(eq) is defined as one third of the trace of the orthogonalized U$_{ij}$ tensor.

	x/a	y/b	z/c	U(eq)
O1	0.98048(10)	0.17571(9)	0.63364(8)	0.02538(19)
C1	0.13770(13)	0.42874(12)	0.64168(10)	0.0175(2)
C2	0.05056(14)	0.39440(14)	0.78158(11)	0.0237(2)
O2	0.19244(10)	0.31650(9)	0.46822(7)	0.02135(18)
N1	0.13890(11)	0.31034(10)	0.59775(8)	0.01752(19)
C4	0.01951(13)	0.25426(12)	0.66679(10)	0.0178(2)
C9	0.41649(13)	0.28301(12)	0.67892(11)	0.0190(2)
C5	0.33354(15)	0.18515(14)	0.44035(12)	0.0281(3)
C6	0.76539(14)	0.40751(14)	0.79407(12)	0.0265(3)
C7	0.95659(16)	0.17517(14)	0.90093(12)	0.0281(3)
C8	0.31343(13)	0.42558(12)	0.61590(10)	0.0165(2)
C22	0.23052(14)	0.70194(13)	0.58258(11)	0.0222(2)
C10	0.49116(14)	0.16386(12)	0.73328(11)	0.0197(2)

	x/a	y/b	z/c	U(eq)
C11	0.57484(13)	0.01920(12)	0.80062(10)	0.0176(2)
C13	0.56142(14)	0.79443(13)	0.95075(11)	0.0226(2)
C12	0.48371(14)	0.93378(13)	0.88518(11)	0.0206(2)
C3	0.94621(13)	0.30762(12)	0.79041(10)	0.0188(2)
C21	0.20885(15)	0.82850(13)	0.61664(12)	0.0263(3)
C17	0.30087(13)	0.56119(12)	0.65251(10)	0.0172(2)
C16	0.74557(14)	0.96193(13)	0.78326(11)	0.0224(2)
C20	0.26002(15)	0.81632(14)	0.71915(13)	0.0278(3)
C18	0.35146(14)	0.54995(13)	0.75562(11)	0.0211(2)
C14	0.73095(15)	0.73813(13)	0.93319(11)	0.0234(2)
C15	0.82237(14)	0.82186(14)	0.84946(12)	0.0257(3)
C19	0.33205(15)	0.67735(15)	0.78803(12)	0.0269(3)

Bond lengths (Å) for rds667.

O1-C4	1.2186(14)	C1-N1	1.4539(14)
C1-C2	1.5389(16)	C1-C8	1.5472(14)
C1-H1	1.0	C2-C3	1.5424(16)
C2-H2A	0.99	C2-H2B	0.99
O2-N1	1.3963(12)	O2-C5	1.4380(14)
N1-C4	1.3582(14)	C4-C3	1.5208(16)
C9-C10	1.1963(16)	C9-C8	1.4678(15)
C5-H5A	0.98	C5-H5B	0.98
C5-H5C	0.98	C6-C3	1.5341(15)
C6-H6A	0.98	C6-H6B	0.98
C6-H6C	0.98	C7-C3	1.5266(16)
C7-H7A	0.98	C7-H7B	0.98
C7-H7C	0.98	C8-C17	1.5245(15)
C8-H8	1.0	C22-C21	1.3854(17)
C22-C17	1.3949(16)	C22-H22	0.95
C10-C11	1.4391(15)	C11-C16	1.3969(15)
C11-C12	1.3983(16)	C13-C12	1.3821(16)
C13-C14	1.3868(17)	C13-H13	0.95
C12-H12	0.95	C21-C20	1.3834(18)
C21-H21	0.95	C17-C18	1.3900(16)
C16-C15	1.3874(17)	C16-H16	0.95
C20-C19	1.3820(18)	C20-H20	0.95
C18-C19	1.3913(17)	C18-H18	0.95
C14-C15	1.3859(17)	C14-H14	0.95
C15-H15	0.95	C19-H19	0.95

Bond angles (°) for rds667.

N1-C1-C2	100.55(8)	N1-C1-C8	111.89(9)

C2-C1-C8	114.98(9)	N1-C1-H1	109.7
C2-C1-H1	109.7	C8-C1-H1	109.7
C1-C2-C3	106.35(9)	C1-C2-H2A	110.5
C3-C2-H2A	110.5	C1-C2-H2B	110.5
C3-C2-H2B	110.5	H2A-C2-H2B	108.6
N1-O2-C5	110.25(8)	C4-N1-O2	119.13(9)
C4-N1-C1	115.44(9)	O2-N1-C1	116.86(8)
O1-C4-N1	125.25(10)	O1-C4-C3	127.31(10)
N1-C4-C3	107.44(9)	C10-C9-C8	175.41(11)
O2-C5-H5A	109.5	O2-C5-H5B	109.5
H5A-C5-H5B	109.5	O2-C5-H5C	109.5
H5A-C5-H5C	109.5	H5B-C5-H5C	109.5
C3-C6-H6A	109.5	C3-C6-H6B	109.5
H6A-C6-H6B	109.5	C3-C6-H6C	109.5
H6A-C6-H6C	109.5	H6B-C6-H6C	109.5
C3-C7-H7A	109.5	C3-C7-H7B	109.5
H7A-C7-H7B	109.5	C3-C7-H7C	109.5
H7A-C7-H7C	109.5	H7B-C7-H7C	109.5
C9-C8-C17	114.23(9)	C9-C8-C1	110.53(9)
C17-C8-C1	108.76(8)	C9-C8-H8	107.7
C17-C8-H8	107.7	C1-C8-H8	107.7
C21-C22-C17	120.53(11)	C21-C22-H22	119.7
C17-C22-H22	119.7	C9-C10-C11	177.62(12)
C16-C11-C12	119.15(10)	C16-C11-C10	121.39(10)
C12-C11-C10	119.46(10)	C12-C13-C14	120.13(11)
C12-C13-H13	119.9	C14-C13-H13	119.9
C13-C12-C11	120.48(10)	C13-C12-H12	119.8
C11-C12-H12	119.8	C4-C3-C7	110.18(9)
C4-C3-C6	106.90(9)	C7-C3-C6	109.95(10)
C4-C3-C2	103.66(9)	C7-C3-C2	113.20(10)
C6-C3-C2	112.60(10)	C20-C21-C22	120.20(11)
C20-C21-H21	119.9	C22-C21-H21	119.9
C18-C17-C22	118.89(10)	C18-C17-C8	122.39(10)
C22-C17-C8	118.68(10)	C15-C16-C11	119.90(11)
C15-C16-H16	120.0	C11-C16-H16	120.0
C19-C20-C21	119.75(11)	C19-C20-H20	120.1
C21-C20-H20	120.1	C17-C18-C19	120.30(11)
C17-C18-H18	119.8	C19-C18-H18	119.8
C15-C14-C13	119.82(11)	C15-C14-H14	120.1
C13-C14-H14	120.1	C14-C15-C16	120.51(11)
C14-C15-H15	119.7	C16-C15-H15	119.7
C20-C19-C18	120.31(11)	C20-C19-H19	119.8
C18-C19-H19	119.8		

Torsion angles (°) for rds667.

N1-C1-C2-C3	-24.43(11)	C8-C1-C2-C3	-144.77(9)
C5-O2-N1-C4	-93.24(11)	C5-O2-N1-C1	120.30(10)
C2-C1-N1-C4	24.72(12)	C8-C1-N1-C4	147.24(9)
C2-C1-N1-O2	172.42(9)	C8-C1-N1-O2	-65.06(12)
O2-N1-C4-O1	18.78(16)	C1-N1-C4-O1	165.70(10)
O2-N1-C4-C3	-161.21(8)	C1-N1-C4-C3	-14.29(12)
N1-C1-C8-C9	-59.35(12)	C2-C1-C8-C9	54.52(12)
N1-C1-C8-C17	174.49(9)	C2-C1-C8-C17	-71.64(11)
C14-C13-C12-C11	0.05(17)	C16-C11-C12-C13	-0.03(16)
C10-C11-C12-C13	179.89(10)	O1-C4-C3-C7	55.52(15)
N1-C4-C3-C7	-124.49(10)	O1-C4-C3-C6	-63.92(14)
N1-C4-C3-C6	116.08(10)	O1-C4-C3-C2	176.93(11)
N1-C4-C3-C2	-3.07(11)	C1-C2-C3-C4	17.56(11)
C1-C2-C3-C7	136.92(10)	C1-C2-C3-C6	-97.60(11)
C17-C22-C21-C20	1.33(18)	C21-C22-C17-C18	-1.10(17)
C21-C22-C17-C8	176.60(10)	C9-C8-C17-C18	-13.45(14)
C1-C8-C17-C18	110.54(11)	C9-C8-C17-C22	168.93(10)
C1-C8-C17-C22	-67.08(12)	C12-C11-C16-C15	0.09(17)
C10-C11-C16-C15	-179.83(11)	C22-C21-C20-C19	-0.38(18)
C22-C17-C18-C19	-0.05(16)	C8-C17-C18-C19	-177.66(10)
C12-C13-C14-C15	-0.14(18)	C13-C14-C15-C16	0.20(18)
C11-C16-C15-C14	-0.18(18)	C21-C20-C19-C18	-0.78(18)
C17-C18-C19-C20	0.99(18)		

Anisotropic atomic displacement parameters (Å^2) for rds667.

The anisotropic atomic displacement factor exponent takes the form: $-2\pi^2[h^2 a^{*2} U_{11} + ... + 2 h k a^* b^* U_{12}]$

	U_{11}	U_{22}	U_{33}	U_{23}	U_{13}	U_{12}
O1	0.0285(4)	0.0251(4)	0.0292(5)	-0.0035(3)	-0.0080(4)	-0.0163(4)
C1	0.0147(5)	0.0162(5)	0.0223(5)	-0.0045(4)	-0.0042(4)	-0.0062(4)
C2	0.0197(5)	0.0310(6)	0.0234(6)	-0.0122(5)	0.0027(4)	-0.0134(5)
O2	0.0238(4)	0.0242(4)	0.0143(4)	-0.0032(3)	-0.0028(3)	-0.0094(3)
N1	0.0180(4)	0.0203(5)	0.0153(4)	-0.0042(3)	-0.0015(3)	-0.0095(4)
C4	0.0153(5)	0.0152(5)	0.0210(5)	0.0002(4)	-0.0068(4)	-0.0050(4)
C9	0.0162(5)	0.0204(5)	0.0207(5)	-0.0050(4)	-0.0026(4)	-0.0079(4)
C5	0.0266(6)	0.0280(6)	0.0264(6)	-0.0118(5)	0.0029(5)	-0.0099(5)
C6	0.0160(5)	0.0242(6)	0.0298(6)	-0.0006(5)	-0.0020(5)	-0.0055(5)
C7	0.0287(6)	0.0262(6)	0.0215(6)	0.0019(5)	-0.0063(5)	-0.0079(5)
C8	0.0152(5)	0.0168(5)	0.0175(5)	-0.0016(4)	-0.0033(4)	-0.0076(4)
C22	0.0236(5)	0.0212(6)	0.0234(6)	0.0002(4)	-0.0070(4)	-0.0121(5)
C10	0.0190(5)	0.0198(5)	0.0213(5)	-0.0059(4)	-0.0040(4)	-0.0075(4)
C11	0.0197(5)	0.0164(5)	0.0176(5)	-0.0057(4)	-0.0054(4)	-0.0053(4)

	U_{11}	U_{22}	U_{33}	U_{23}	U_{13}	U_{12}
C13	0.0254(6)	0.0218(6)	0.0207(6)	-0.0027(4)	-0.0036(4)	-0.0117(5)
C12	0.0173(5)	0.0226(6)	0.0221(5)	-0.0061(4)	-0.0042(4)	-0.0070(4)
C3	0.0156(5)	0.0186(5)	0.0198(5)	-0.0018(4)	-0.0028(4)	-0.0069(4)
C21	0.0251(6)	0.0189(6)	0.0341(7)	-0.0015(5)	-0.0058(5)	-0.0113(5)
C17	0.0136(5)	0.0185(5)	0.0200(5)	-0.0032(4)	-0.0017(4)	-0.0087(4)
C16	0.0194(5)	0.0216(6)	0.0257(6)	-0.0021(4)	-0.0043(4)	-0.0099(4)
C20	0.0250(6)	0.0249(6)	0.0379(7)	-0.0129(5)	-0.0013(5)	-0.0133(5)
C18	0.0182(5)	0.0232(6)	0.0221(6)	-0.0026(4)	-0.0049(4)	-0.0093(4)
C14	0.0258(6)	0.0183(5)	0.0231(6)	-0.0031(4)	-0.0081(5)	-0.0046(5)
C15	0.0172(5)	0.0245(6)	0.0314(6)	-0.0036(5)	-0.0070(5)	-0.0049(5)
C19	0.0248(6)	0.0345(7)	0.0280(6)	-0.0114(5)	-0.0051(5)	-0.0142(5)

Hydrogen atomic coordinates and isotropic atomic displacement parameters (Å^2) for rds667.

	x/a	y/b	z/c	U(eq)
H1	0.0681	0.5273	0.5999	0.021
H2A	-0.0207	0.4877	0.8128	0.028
H2B	0.1324	0.3338	0.8324	0.028
H5A	0.3725	0.1925	0.3491	0.042
H5B	0.3029	0.0975	0.4795	0.042
H5C	0.4214	0.1759	0.4734	0.042
H6A	-0.2921	0.3505	0.7881	0.04
H6B	-0.2403	0.4926	0.7232	0.04
H6C	-0.2870	0.4432	0.8732	0.04
H7A	-0.1041	0.1196	0.8975	0.042
H7B	-0.0917	0.2109	0.9800	0.042
H7C	0.0722	0.1100	0.8963	0.042
H8	0.3632	0.4346	0.5233	0.02
H22	0.1972	0.7111	0.5110	0.027
H13	0.4986	-0.2629	1.0080	0.027
H12	0.3675	-0.0282	0.8976	0.025
H21	0.1587	0.9239	0.5695	0.032
H16	0.8090	0.0188	0.7262	0.027
H20	0.2457	0.9032	0.7421	0.033
H18	0.3995	0.4548	0.8042	0.025
H14	0.7843	-0.3576	0.9784	0.028
H15	0.9385	-0.2170	0.8373	0.031
H19	0.3685	0.6688	0.8578	0.032

2. Crystal Structure Report for rds624 (17a-major)

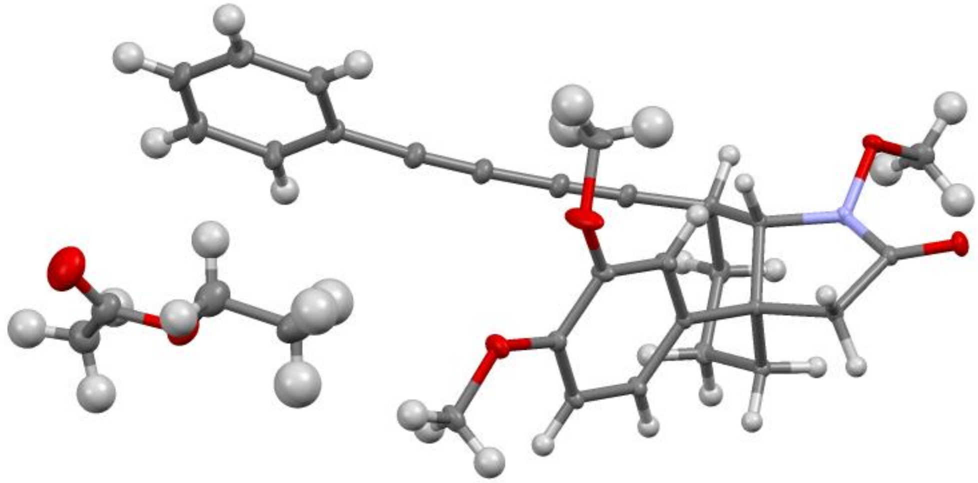

A colorless block-like specimen of $C_{31}H_{35}NO_6$, approximate dimensions 0.220 mm x 0.392 mm x 0.538 mm, was used for the X-ray crystallographic analysis. The X-ray intensity data were measured.
The total exposure time was 6.26 hours. The frames were integrated with the Bruker SAINT software package using a narrow-frame algorithm. The integration of the data using a monoclinic unit cell yielded a total of 94581 reflections to a maximum θ angle of 35.12° (0.62 Å resolution), of which 12175 were independent (average redundancy 7.768, completeness = 99.7%, R_{int} = 3.57%, R_{sig} = 2.44%) and 9610 (78.93%) were greater than $2\sigma(F^2)$. The final cell constants of a = 14.8729(4) Å, b = 11.2262(3) Å, c = 16.4910(5) Å, β = 92.9640(13)°, volume = 2749.76(13) $Å^3$, are based upon the refinement of the XYZ-centroids of 275 reflections above 20 σ(I) with 5.230° < 2θ < 60.61°. Data were corrected for absorption effects using the Multi-Scan method (SADABS). The ratio of minimum to maximum apparent transmission was 0.930. The calculated minimum and maximum transmission coefficients (based on crystal size) are 0.9550 and 0.9810.
The final anisotropic full-matrix least-squares refinement on F^2 with 348 variables converged at R1 = 5.19%, for the observed data and wR2 = 15.81% for all data. The goodness-of-fit was 1.042. The largest peak in the final difference electron density synthesis was 0.868 $e^-/Å^3$ and the largest hole was -0.891 $e^-/Å^3$ with an RMS deviation of 0.073 $e^-/Å^3$. On the basis of the final model, the calculated density was 1.250 g/cm^3 and F(000), 1104 e^-.

Sample and crystal data for rds624.

**Identification code**	rds624	
**Chemical formula**	$C_{31}H_{35}NO_6$	
**Formula weight**	517.60 g/mol	
**Temperature**	100(2) K	
**Wavelength**	0.71073 Å	
**Crystal size**	0.220 x 0.392 x 0.538 mm	
**Crystal habit**	colorless block	
**Crystal system**	monoclinic	
**Space group**	P 1 21/c 1	
**Unit cell dimensions**	a = 14.8729(4) Å	α = 90°
	b = 11.2262(3) Å	β = 92.9640(13)°
	c = 16.4910(5) Å	γ = 90°
**Volume**	2749.76(13) $Å^3$	
**Z**	4	

**Density (calculated)**	1.250 g/cm^3
**Absorption coefficient**	0.086 mm^{-1}
**F(000)**	1104

Data collection and structure refinement for rds624.

**Theta range for data collection**	2.20 to 35.12°	
**Index ranges**	-23<=h<=24, -18<=k<=18, -26<=l<=18	
**Reflections collected**	94581	
**Independent reflections**	12175 [R(int) = 0.0357]	
**Coverage of independent reflections**	99.7%	
**Absorption correction**	Multi-Scan	
**Max. and min. transmission**	0.9810 and 0.9550	
**Refinement method**	Full-matrix least-squares on F^2	
**Refinement program**	SHELXL-2014/7 (Sheldrick, 2014)	
**Function minimized**	Σ w(F$_o^2$ - F$_c^2$)^2	
**Data / restraints / parameters**	12175 / 0 / 348	
**Goodness-of-fit on F^2**	1.042	
**Δ/σ_{max}**	0.001	
**Final R indices**	9610 data; I>2σ(I)	R1 = 0.0519, wR2 = 0.1448
	all data	R1 = 0.0688, wR2 = 0.1581
**Weighting scheme**	w=1/[σ^2(F$_o^2$)+(0.0820P)^2+1.2208P]<br>where P=(F$_o^2$+2F$_c^2$)/3	
**Largest diff. peak and hole**	0.868 and -0.891 eÅ^{-3}	
**R.M.S. deviation from mean**	0.073 eÅ^{-3}	

Atomic coordinates and equivalent isotropic atomic displacement parameters (Å^2) for rds624.

U(eq) is defined as one third of the trace of the orthogonalized U_{ij} tensor.

	x/a	y/b	z/c	U(eq)
O1	0.63629(5)	0.94950(6)	0.48374(4)	0.01555(13)
O2	0.58490(5)	0.15223(7)	0.56066(4)	0.01534(13)
O3	0.22184(5)	0.17390(8)	0.12783(5)	0.01821(14)
O4	0.21076(5)	0.09559(11)	0.27560(5)	0.0308(2)
N1	0.58442(5)	0.14179(7)	0.47633(5)	0.01112(13)
C1	0.51613(6)	0.20878(7)	0.42852(5)	0.00997(14)
C2	0.53566(6)	0.16348(7)	0.34179(5)	0.00934(13)
C3	0.61989(6)	0.22692(8)	0.31234(6)	0.01155(14)
C4	0.61751(6)	0.36227(8)	0.32004(6)	0.01466(16)
C5	0.60958(6)	0.39706(8)	0.40878(6)	0.01515(16)
C6	0.52405(6)	0.34367(8)	0.44254(6)	0.01246(15)
C7	0.56191(6)	0.03179(8)	0.35888(5)	0.01206(14)
C8	0.59976(6)	0.03088(8)	0.44547(5)	0.01132(14)
C9	0.67374(7)	0.18205(12)	0.59154(7)	0.0227(2)

	x/a	y/b	z/c	U(eq)
C10	0.44249(6)	0.40179(8)	0.40771(6)	0.01489(16)
C11	0.37432(7)	0.44776(9)	0.38016(6)	0.01629(17)
C12	0.29596(7)	0.50131(9)	0.35179(7)	0.01743(17)
C13	0.22705(7)	0.55240(10)	0.33031(7)	0.01823(18)
C14	0.14626(6)	0.61805(9)	0.31118(6)	0.01617(17)
C15	0.06250(7)	0.56997(11)	0.32845(7)	0.02137(19)
C16	0.98466(7)	0.63644(12)	0.31396(8)	0.0256(2)
C17	0.98943(8)	0.75073(12)	0.28272(8)	0.0257(2)
C18	0.07231(8)	0.79901(11)	0.26486(8)	0.0248(2)
C19	0.15046(7)	0.73272(10)	0.27836(7)	0.02070(19)
C20	0.45325(6)	0.17291(7)	0.28312(5)	0.00970(13)
C21	0.45791(6)	0.21430(8)	0.20430(5)	0.01223(15)
C22	0.38205(6)	0.21668(8)	0.15047(6)	0.01307(15)
C23	0.29964(6)	0.17708(8)	0.17523(6)	0.01252(15)
C24	0.29367(6)	0.13581(9)	0.25521(6)	0.01478(16)
C25	0.36936(6)	0.13325(9)	0.30770(5)	0.01337(15)
C26	0.22784(7)	0.20642(10)	0.04456(6)	0.01979(19)
C27	0.18786(8)	0.11500(17)	0.35675(8)	0.0359(3)
O1S	0.99208(8)	0.81083(13)	0.05570(9)	0.0501(3)
O2S	0.12522(6)	0.71439(9)	0.06310(7)	0.0313(2)
C1S	0.07167(9)	0.81341(12)	0.05117(9)	0.0280(2)
C2S	0.12678(9)	0.92287(13)	0.04103(9)	0.0311(3)
C3S	0.07744(10)	0.60424(13)	0.07964(11)	0.0368(3)
C4S	0.14528(13)	0.50579(16)	0.08933(12)	0.0462(4)

Bond lengths (Å) for rds624.

O1-C8	1.2217(11)	O2-N1	1.3952(10)
O2-C9	1.4309(13)	O3-C23	1.3631(11)
O3-C26	1.4281(13)	O4-C24	1.3713(12)
O4-C27	1.4147(15)	N1-C8	1.3686(12)
N1-C1	1.4615(11)	C1-C6	1.5354(12)
C1-C2	1.5594(12)	C1-H1	1.0
C2-C20	1.5254(12)	C2-C3	1.5411(12)
C2-C7	1.5511(12)	C3-C4	1.5254(13)
C3-H3A	0.99	C3-H3B	0.99
C4-C5	1.5250(14)	C4-H4A	0.99
C4-H4B	0.99	C5-C6	1.5365(13)
C5-H5A	0.99	C5-H5B	0.99
C6-C10	1.4679(13)	C6-H6	1.0
C7-C8	1.5077(12)	C7-H7A	0.99
C7-H7B	0.99	C9-H9A	0.98
C9-H9B	0.98	C9-H9C	0.98

C10-C11	1.2054(13)	C11-C12	1.3719(14)
C12-C13	1.2113(14)	C13-C14	1.4309(14)
C14-C19	1.3990(15)	C14-C15	1.4004(14)
C15-C16	1.3878(16)	C15-H15	0.95
C16-C17	1.3857(19)	C16-H16	0.95
C17-C18	1.3920(18)	C17-H17	0.95
C18-C19	1.3884(15)	C18-H18	0.95
C19-H19	0.95	C20-C21	1.3856(12)
C20-C25	1.4041(12)	C21-C22	1.3993(12)
C21-H21	0.95	C22-C23	1.3850(13)
C22-H22	0.95	C23-C24	1.4051(13)
C24-C25	1.3844(13)	C25-H25	0.95
C26-H26A	0.98	C26-H26B	0.98
C26-H26C	0.98	C27-H27A	0.98
C27-H27B	0.98	C27-H27C	0.98
O1S-C1S	1.1904(18)	O2S-C1S	1.3756(16)
O2S-C3S	1.4590(18)	C1S-C2S	1.4913(19)
C2S-H2SA	0.98	C2S-H2SB	0.98
C2S-H2SC	0.98	C3S-C4S	1.500(2)
C3S-H3SA	0.99	C3S-H3SB	0.99
C4S-H4SA	0.98	C4S-H4SB	0.98
C4S-H4SC	0.98		

Bond angles (°) for rds624.

N1-O2-C9	109.36(7)	C23-O3-C26	116.83(8)
C24-O4-C27	116.41(9)	C8-N1-O2	117.05(7)
C8-N1-C1	113.08(7)	O2-N1-C1	117.51(7)
N1-C1-C6	112.37(7)	N1-C1-C2	99.77(6)
C6-C1-C2	116.31(7)	N1-C1-H1	109.3
C6-C1-H1	109.3	C2-C1-H1	109.3
C20-C2-C3	114.05(7)	C20-C2-C7	111.58(7)
C3-C2-C7	107.26(7)	C20-C2-C1	112.17(7)
C3-C2-C1	109.35(7)	C7-C2-C1	101.61(7)
C4-C3-C2	114.29(7)	C4-C3-H3A	108.7
C2-C3-H3A	108.7	C4-C3-H3B	108.7
C2-C3-H3B	108.7	H3A-C3-H3B	107.6
C5-C4-C3	109.77(8)	C5-C4-H4A	109.7
C3-C4-H4A	109.7	C5-C4-H4B	109.7
C3-C4-H4B	109.7	H4A-C4-H4B	108.2
C4-C5-C6	110.76(7)	C4-C5-H5A	109.5
C6-C5-H5A	109.5	C4-C5-H5B	109.5
C6-C5-H5B	109.5	H5A-C5-H5B	108.1
C10-C6-C1	108.91(7)	C10-C6-C5	111.57(8)

C1-C6-C5	112.87(7)	C10-C6-H6	107.8
C1-C6-H6	107.8	C5-C6-H6	107.8
C8-C7-C2	104.87(7)	C8-C7-H7A	110.8
C2-C7-H7A	110.8	C8-C7-H7B	110.8
C2-C7-H7B	110.8	H7A-C7-H7B	108.8
O1-C8-N1	124.50(8)	O1-C8-C7	128.95(8)
N1-C8-C7	106.55(7)	O2-C9-H9A	109.5
O2-C9-H9B	109.5	H9A-C9-H9B	109.5
O2-C9-H9C	109.5	H9A-C9-H9C	109.5
H9B-C9-H9C	109.5	C11-C10-C6	178.46(11)
C10-C11-C12	177.79(11)	C13-C12-C11	176.56(12)
C12-C13-C14	175.24(12)	C19-C14-C15	119.43(9)
C19-C14-C13	120.36(9)	C15-C14-C13	120.15(10)
C16-C15-C14	120.04(11)	C16-C15-H15	120.0
C14-C15-H15	120.0	C17-C16-C15	120.24(10)
C17-C16-H16	119.9	C15-C16-H16	119.9
C16-C17-C18	120.14(10)	C16-C17-H17	119.9
C18-C17-H17	119.9	C19-C18-C17	120.03(11)
C19-C18-H18	120.0	C17-C18-H18	120.0
C18-C19-C14	120.10(10)	C18-C19-H19	120.0
C14-C19-H19	120.0	C21-C20-C25	117.74(8)
C21-C20-C2	122.73(7)	C25-C20-C2	119.46(8)
C20-C21-C22	121.58(8)	C20-C21-H21	119.2
C22-C21-H21	119.2	C23-C22-C21	120.25(8)
C23-C22-H22	119.9	C21-C22-H22	119.9
O3-C23-C22	125.26(8)	O3-C23-C24	115.90(8)
C22-C23-C24	118.84(8)	O4-C24-C25	123.53(9)
O4-C24-C23	116.11(8)	C25-C24-C23	120.32(8)
C24-C25-C20	121.26(8)	C24-C25-H25	119.4
C20-C25-H25	119.4	O3-C26-H26A	109.5
O3-C26-H26B	109.5	H26A-C26-H26B	109.5
O3-C26-H26C	109.5	H26A-C26-H26C	109.5
H26B-C26-H26C	109.5	O4-C27-H27A	109.5
O4-C27-H27B	109.5	H27A-C27-H27B	109.5
O4-C27-H27C	109.5	H27A-C27-H27C	109.5
H27B-C27-H27C	109.5	C1S-O2S-C3S	115.32(11)
O1S-C1S-O2S	122.80(14)	O1S-C1S-C2S	125.54(14)
O2S-C1S-C2S	111.37(11)	C1S-C2S-H2SA	109.5
C1S-C2S-H2SB	109.5	H2SA-C2S-H2SB	109.5
C1S-C2S-H2SC	109.5	H2SA-C2S-H2SC	109.5
H2SB-C2S-H2SC	109.5	O2S-C3S-C4S	108.25(13)
O2S-C3S-H3SA	110.0	C4S-C3S-H3SA	110.0
O2S-C3S-H3SB	110.0	C4S-C3S-H3SB	110.0

H3SA-C3S-H3SB	108.4	C3S-C4S-H4SA	109.5
C3S-C4S-H4SB	109.5	H4SA-C4S-H4SB	109.5
C3S-C4S-H4SC	109.5	H4SA-C4S-H4SC	109.5
H4SB-C4S-H4SC	109.5		

Torsion angles (°) for rds624.

C9-O2-N1-C8	91.77(10)	C9-O2-N1-C1	-128.56(9)
C8-N1-C1-C6	-158.40(8)	O2-N1-C1-C6	60.40(10)
C8-N1-C1-C2	-34.54(9)	O2-N1-C1-C2	-175.74(7)
N1-C1-C2-C20	154.98(7)	C6-C1-C2-C20	-83.96(9)
N1-C1-C2-C3	-77.46(8)	C6-C1-C2-C3	43.61(9)
N1-C1-C2-C7	35.70(8)	C6-C1-C2-C7	156.76(7)
C20-C2-C3-C4	75.72(10)	C7-C2-C3-C4	-160.19(7)
C1-C2-C3-C4	-50.78(10)	C2-C3-C4-C5	60.09(10)
C3-C4-C5-C6	-58.85(10)	N1-C1-C6-C10	-167.35(8)
C2-C1-C6-C10	78.56(9)	N1-C1-C6-C5	68.16(10)
C2-C1-C6-C5	-45.94(10)	C4-C5-C6-C10	-70.62(10)
C4-C5-C6-C1	52.41(10)	C20-C2-C7-C8	-147.17(7)
C3-C2-C7-C8	87.25(8)	C1-C2-C7-C8	-27.46(8)
O2-N1-C8-O1	-20.22(13)	C1-N1-C8-O1	-161.61(9)
O2-N1-C8-C7	158.88(7)	C1-N1-C8-C7	17.48(10)
C2-C7-C8-O1	-173.10(9)	C2-C7-C8-N1	7.86(9)
C19-C14-C15-C16	-0.79(17)	C13-C14-C15-C16	176.47(11)
C14-C15-C16-C17	-0.29(19)	C15-C16-C17-C18	0.72(19)
C16-C17-C18-C19	-0.05(19)	C17-C18-C19-C14	-1.04(19)
C15-C14-C19-C18	1.46(17)	C13-C14-C19-C18	-175.80(11)
C3-C2-C20-C21	11.78(12)	C7-C2-C20-C21	-109.95(9)
C1-C2-C20-C21	136.80(8)	C3-C2-C20-C25	-171.28(8)
C7-C2-C20-C25	66.99(10)	C1-C2-C20-C25	-46.27(10)
C25-C20-C21-C22	-0.12(13)	C2-C20-C21-C22	176.87(8)
C20-C21-C22-C23	-0.05(14)	C26-O3-C23-C22	4.57(14)
C26-O3-C23-C24	-175.15(9)	C21-C22-C23-O3	-179.11(9)
C21-C22-C23-C24	0.60(14)	C27-O4-C24-C25	35.73(18)
C27-O4-C24-C23	-146.60(12)	O3-C23-C24-O4	1.00(14)
C22-C23-C24-O4	-178.74(10)	O3-C23-C24-C25	178.74(9)
C22-C23-C24-C25	-0.99(15)	O4-C24-C25-C20	178.42(10)
C23-C24-C25-C20	0.85(15)	C21-C20-C25-C24	-0.29(14)
C2-C20-C25-C24	-177.38(9)	C3S-O2S-C1S-O1S	1.6(2)
C3S-O2S-C1S-C2S	175.66(12)	C1S-O2S-C3S-C4S	178.88(13)

Anisotropic atomic displacement parameters (Å^2) for rds624.

The anisotropic atomic displacement factor exponent takes the form: $-2\pi^2[h^2 a^{*2} U_{11} + ... + 2 h k a^* b^* U_{12}]$

U_{11}	U_{22}	U_{33}	U_{23}	U_{13}	U_{12}

	U_{11}	U_{22}	U_{33}	U_{23}	U_{13}	U_{12}
O1	0.0182(3)	0.0123(3)	0.0158(3)	0.0042(2)	-0.0022(2)	0.0022(2)
O2	0.0146(3)	0.0230(3)	0.0083(3)	-0.0004(2)	-0.0003(2)	0.0009(2)
O3	0.0122(3)	0.0280(4)	0.0139(3)	0.0018(3)	-0.0042(2)	-0.0032(3)
O4	0.0133(3)	0.0638(7)	0.0155(3)	0.0010(4)	0.0015(3)	-0.0152(4)
N1	0.0137(3)	0.0114(3)	0.0081(3)	0.0002(2)	-0.0012(2)	0.0013(2)
C1	0.0096(3)	0.0096(3)	0.0106(3)	0.0003(3)	-0.0004(2)	0.0002(2)
C2	0.0096(3)	0.0087(3)	0.0097(3)	0.0009(2)	0.0002(2)	-0.0002(2)
C3	0.0095(3)	0.0123(3)	0.0129(3)	0.0020(3)	0.0009(3)	-0.0002(3)
C4	0.0135(4)	0.0122(3)	0.0183(4)	0.0038(3)	0.0010(3)	-0.0025(3)
C5	0.0138(4)	0.0106(3)	0.0209(4)	-0.0008(3)	-0.0010(3)	-0.0025(3)
C6	0.0119(3)	0.0103(3)	0.0151(4)	-0.0013(3)	-0.0011(3)	0.0018(3)
C7	0.0159(4)	0.0087(3)	0.0113(3)	0.0003(3)	-0.0016(3)	0.0006(3)
C8	0.0117(3)	0.0104(3)	0.0119(3)	0.0018(3)	0.0002(3)	-0.0004(3)
C9	0.0183(4)	0.0321(5)	0.0171(4)	-0.0070(4)	-0.0056(3)	-0.0002(4)
C10	0.0147(4)	0.0117(3)	0.0182(4)	-0.0014(3)	0.0005(3)	0.0020(3)
C11	0.0150(4)	0.0141(4)	0.0198(4)	-0.0002(3)	0.0008(3)	0.0023(3)
C12	0.0144(4)	0.0168(4)	0.0211(4)	0.0010(3)	0.0006(3)	0.0021(3)
C13	0.0136(4)	0.0187(4)	0.0222(4)	0.0001(3)	-0.0002(3)	0.0016(3)
C14	0.0111(3)	0.0188(4)	0.0183(4)	-0.0001(3)	-0.0015(3)	0.0019(3)
C15	0.0129(4)	0.0252(5)	0.0257(5)	0.0024(4)	-0.0012(3)	-0.0014(3)
C16	0.0110(4)	0.0388(6)	0.0269(5)	0.0012(5)	-0.0005(4)	0.0007(4)
C17	0.0160(4)	0.0344(6)	0.0264(5)	-0.0025(4)	-0.0035(4)	0.0101(4)
C18	0.0207(5)	0.0227(5)	0.0301(6)	0.0022(4)	-0.0056(4)	0.0061(4)
C19	0.0136(4)	0.0216(4)	0.0265(5)	0.0032(4)	-0.0027(3)	0.0013(3)
C20	0.0094(3)	0.0091(3)	0.0105(3)	0.0005(3)	-0.0002(2)	-0.0008(2)
C21	0.0113(3)	0.0133(3)	0.0119(3)	0.0030(3)	-0.0005(3)	-0.0022(3)
C22	0.0126(3)	0.0146(4)	0.0118(3)	0.0027(3)	-0.0018(3)	-0.0020(3)
C23	0.0109(3)	0.0142(4)	0.0122(3)	-0.0009(3)	-0.0019(3)	-0.0007(3)
C24	0.0101(3)	0.0218(4)	0.0124(4)	-0.0003(3)	0.0006(3)	-0.0036(3)
C25	0.0115(3)	0.0178(4)	0.0108(3)	0.0013(3)	0.0002(3)	-0.0035(3)
C26	0.0194(4)	0.0240(5)	0.0153(4)	0.0037(3)	-0.0061(3)	-0.0033(4)
C27	0.0145(4)	0.0742(11)	0.0196(5)	0.0081(6)	0.0054(4)	-0.0002(5)
O1S	0.0310(6)	0.0538(8)	0.0648(9)	-0.0054(7)	-0.0041(5)	0.0022(5)
O2S	0.0234(4)	0.0292(5)	0.0416(6)	-0.0029(4)	0.0031(4)	0.0001(3)
C1S	0.0229(5)	0.0282(6)	0.0326(6)	-0.0050(5)	-0.0014(4)	0.0005(4)
C2S	0.0294(6)	0.0305(6)	0.0329(6)	-0.0003(5)	-0.0015(5)	-0.0034(5)
C3S	0.0328(7)	0.0292(6)	0.0485(9)	-0.0061(6)	0.0041(6)	-0.0022(5)
C4S	0.0521(10)	0.0357(8)	0.0527(10)	0.0003(7)	0.0209(8)	0.0111(7)

Hydrogen atomic coordinates and isotropic atomic displacement parameters ($Å^2$) for rds624.

	x/a	y/b	z/c	U(eq)
H1	0.4549	0.1815	0.4428	0.012

	x/a	y/b	z/c	U(eq)
H3A	0.6735	0.1966	0.3439	0.014
H3B	0.6269	0.2060	0.2547	0.014
H4A	0.6732	0.3967	0.2994	0.018
H4B	0.5655	0.3943	0.2870	0.018
H5A	0.6077	0.4849	0.4135	0.018
H5B	0.6631	0.3681	0.4412	0.018
H6	0.5267	0.3579	0.5025	0.015
H7A	0.5085	-0.0207	0.3525	0.014
H7B	0.6076	0.0046	0.3214	0.014
H9A	0.6776	0.1720	0.6507	0.034
H9B	0.6869	0.2650	0.5780	0.034
H9C	0.7175	0.1295	0.5671	0.034
H15	0.0590	0.4918	0.3501	0.026
H16	-0.0721	0.6035	0.3255	0.031
H17	-0.0640	0.7962	0.2735	0.031
H18	0.0754	0.8774	0.2434	0.03
H19	0.2069	0.7653	0.2653	0.025
H21	0.5140	0.2417	0.1864	0.015
H22	0.3871	0.2456	0.0968	0.016
H25	0.3644	0.1041	0.3614	0.016
H26A	0.2509	0.2880	0.0412	0.03
H26B	0.1680	0.2019	0.0169	0.03
H26C	0.2688	0.1517	0.0185	0.03
H27A	0.2256	0.0647	0.3932	0.054
H27B	0.1244	0.0949	0.3623	0.054
H27C	0.1978	0.1989	0.3709	0.054
H2SA	0.1695	0.9094	-0.0013	0.047
H2SB	0.1599	0.9415	0.0924	0.047
H2SC	0.0871	0.9897	0.0254	0.047
H3SA	0.0335	0.5860	0.0342	0.044
H3SB	0.0443	0.6127	0.1299	0.044
H4SA	0.1814	0.5028	0.0414	0.069
H4SB	0.1140	0.4297	0.0952	0.069
H4SC	0.1847	0.5205	0.1378	0.069

3. Crystal Structure Report for rds625 (17a-minor)

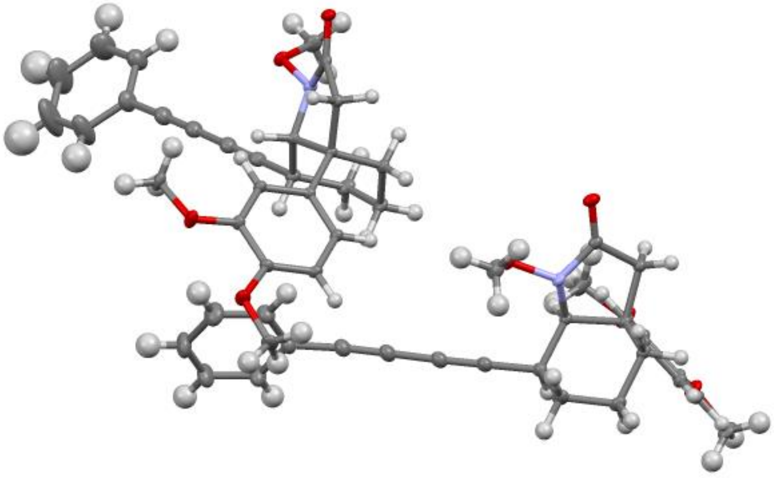

A colorless plate-like specimen of $C_{58}H_{62}N_2O_{10}$, approximate dimensions 0.098 mm x 0.225 mm x 0.352 mm, was used for the X-ray crystallographic analysis. The X-ray intensity data were measured.
The total exposure time was 28.09 hours. The frames were integrated with the Bruker SAINT software package using a narrow-frame algorithm. The integration of the data using a triclinic unit cell yielded a total of 98492 reflections to a maximum θ angle of 30.59° (0.70 Å resolution), of which 15311 were independent (average redundancy 6.433, completeness = 99.6%, R_{int} = 4.16%, R_{sig} = 3.23%) and 11862 (77.47%) were greater than $2\sigma(F^2)$. The final cell constants of a = 10.3428(7) Å, b = 16.0861(11) Å, c = 17.3663(12) Å, α = 65.705(3)°, β = 73.172(4)°, γ = 89.520(4)°, volume = 2500.3(3) Å^3, are based upon the refinement of the XYZ-centroids of 9798 reflections above 20 σ(I) with 4.656° < 2θ < 60.92°. Data were corrected for absorption effects using the Multi-Scan method (SADABS). The ratio of minimum to maximum apparent transmission was 0.925. The calculated minimum and maximum transmission coefficients (based on crystal size) are 0.9700 and 0.9920.
The final anisotropic full-matrix least-squares refinement on F^2 with 696 variables converged at R1 = 6.16%, for the observed data and wR2 = 17.71% for all data. The goodness-of-fit was 1.098. The largest peak in the final difference electron density synthesis was 0.664 $e^-/Å^3$ and the largest hole was -0.485 $e^-/Å^3$ with an RMS deviation of 0.068 $e^-/Å^3$. On the basis of the final model, the calculated density was 1.258 g/cm^3 and F(000), 1008 e^-.

Sample and crystal data for rds625.

**Identification code**	rds625	
**Chemical formula**	$C_{58}H_{62}N_2O_{10}$	
**Formula weight**	947.09 g/mol	
**Temperature**	100(2) K	
**Wavelength**	0.71073 Å	
**Crystal size**	0.098 x 0.225 x 0.352 mm	
**Crystal habit**	colorless plate	
**Crystal system**	triclinic	
**Space group**	P -1	
**Unit cell dimensions**	a = 10.3428(7) Å	α = 65.705(3)°
	b = 16.0861(11) Å	β = 73.172(4)°
	c = 17.3663(12) Å	γ = 89.520(4)°
**Volume**	2500.3(3) Å^3	
**Z**	2	
**Density (calculated)**	1.258 g/cm^3	

**Absorption coefficient**	0.086 mm^{-1}
**F(000)**	1008

Data collection and structure refinement for rds625.

**Theta range for data collection**	1.48 to 30.59°	
**Index ranges**	-14<=h<=14, -22<=k<=22, -24<=l<=24	
**Reflections collected**	98492	
**Independent reflections**	15311 [R(int) = 0.0416]	
**Coverage of independent reflections**	99.6%	
**Absorption correction**	Multi-Scan	
**Max. and min. transmission**	0.9920 and 0.9700	
**Refinement method**	Full-matrix least-squares on F^2	
**Refinement program**	SHELXL-2014/7 (Sheldrick, 2014)	
**Function minimized**	Σ w(F_o^2 - F_c^2)^2	
**Data / restraints / parameters**	15311 / 70 / 696	
**Goodness-of-fit on F^2**	1.098	
**Final R indices**	11862 data; I>2σ(I)	R1 = 0.0616, wR2 = 0.1663
	all data	R1 = 0.0813, wR2 = 0.1771
**Weighting scheme**	w=1/[$\sigma^2(F_o^2)+(0.0750P)^2+2.1946P$]<br>where P=($F_o^2+2F_c^2$)/3	
**Largest diff. peak and hole**	0.664 and -0.485 eÅ^{-3}	
**R.M.S. deviation from mean**	0.068 eÅ^{-3}	

Atomic coordinates and equivalent isotropic atomic displacement parameters (Å^2) for rds625.

U(eq) is defined as one third of the trace of the orthogonalized U_{ij} tensor.

	x/a	y/b	z/c	U(eq)
O1	0.14864(12)	0.06430(8)	0.42312(8)	0.0166(2)
O2	0.18174(12)	0.96240(8)	0.58399(8)	0.0163(2)
O3	0.42852(12)	0.17598(8)	0.75151(9)	0.0209(3)
O4	0.49008(12)	0.03146(8)	0.72538(9)	0.0222(3)
N1	0.12870(13)	0.04657(9)	0.56441(9)	0.0133(2)
C1	0.02133(15)	0.05484(10)	0.63677(10)	0.0122(3)
C2	0.98180(15)	0.15110(10)	0.58432(10)	0.0116(3)
C3	0.09017(16)	0.22744(11)	0.56759(11)	0.0146(3)
C4	0.12166(17)	0.21867(11)	0.65108(12)	0.0177(3)
C5	0.17219(18)	0.12635(12)	0.69346(12)	0.0200(3)
C6	0.06491(17)	0.04753(11)	0.71651(11)	0.0160(3)
C7	0.99631(16)	0.14902(11)	0.49363(10)	0.0137(3)
C8	0.09958(15)	0.08374(10)	0.48562(10)	0.0133(3)
C9	0.32547(17)	0.97835(13)	0.56474(13)	0.0222(3)
C10	0.10492(18)	0.95641(12)	0.76063(11)	0.0187(3)
C11	0.13623(19)	0.88119(13)	0.79622(12)	0.0210(3)
C12	0.17008(19)	0.79456(13)	0.83722(12)	0.0225(3)

	x/a	y/b	z/c	U(eq)
C13	0.1981(2)	0.71842(13)	0.87401(12)	0.0252(4)
C14	0.2327(2)	0.62871(14)	0.91930(14)	0.0296(4)
C15	0.3534(2)	0.60117(15)	0.88168(15)	0.0332(5)
C16	0.3878(3)	0.51524(18)	0.9273(2)	0.0506(7)
C17	0.3050(4)	0.4582(2)	0.0119(2)	0.0712(11)
C18	0.1865(4)	0.4852(2)	0.0506(2)	0.0784(13)
C19	0.1479(3)	0.56981(19)	0.00403(19)	0.0547(8)
C20	0.83673(15)	0.16131(10)	0.63015(10)	0.0117(3)
C21	0.80139(16)	0.23843(10)	0.64463(10)	0.0136(3)
C22	0.66569(16)	0.24558(11)	0.68547(11)	0.0147(3)
C23	0.56334(16)	0.17574(11)	0.71218(11)	0.0147(3)
C24	0.59711(16)	0.09691(11)	0.69786(11)	0.0152(3)
C25	0.73097(16)	0.09077(10)	0.65703(11)	0.0140(3)
C26	0.39058(19)	0.25366(13)	0.76911(13)	0.0247(4)
C27	0.52330(18)	0.94497(12)	0.72624(14)	0.0227(4)
O5	0.42083(13)	0.56417(8)	0.42335(8)	0.0178(2)
O6	0.23903(12)	0.46253(8)	0.58630(8)	0.0171(2)
O7	0.83718(13)	0.67723(8)	0.74236(9)	0.0205(3)
O8	0.80234(14)	0.53297(9)	0.71591(10)	0.0238(3)
N2	0.30920(14)	0.54707(9)	0.56511(9)	0.0142(3)
C28	0.35035(16)	0.55643(10)	0.63579(10)	0.0130(3)
C29	0.43821(15)	0.65262(10)	0.58146(10)	0.0120(3)
C30	0.34395(16)	0.72896(11)	0.56610(11)	0.0149(3)
C31	0.23435(17)	0.72050(11)	0.65068(12)	0.0180(3)
C32	0.14474(17)	0.62788(12)	0.69534(13)	0.0208(3)
C33	0.23346(17)	0.54970(11)	0.71681(11)	0.0168(3)
C34	0.50756(16)	0.64992(11)	0.49077(10)	0.0141(3)
C35	0.41180(16)	0.58408(11)	0.48509(11)	0.0140(3)
C36	0.11107(18)	0.47772(13)	0.57021(14)	0.0249(4)
C37	0.15291(18)	0.45850(12)	0.76421(12)	0.0194(3)
C38	0.08650(18)	0.38422(13)	0.80206(12)	0.0213(3)
C39	0.00992(19)	0.29901(13)	0.84309(12)	0.0221(3)
C40	0.94234(19)	0.22529(13)	0.87917(12)	0.0233(4)
C41	0.85467(19)	0.13969(12)	0.92560(12)	0.0229(3)
C42	0.7840(2)	0.10997(14)	0.88204(13)	0.0270(4)
C43	0.6934(3)	0.03020(16)	0.92894(16)	0.0369(5)
C44	0.6712(3)	0.98003(16)	0.01993(16)	0.0388(5)
C45	0.7403(3)	0.00882(15)	0.06357(14)	0.0358(5)
C46	0.8322(2)	0.08807(15)	0.01703(13)	0.0323(5)
C47	0.54145(15)	0.66300(10)	0.62544(10)	0.0123(3)
C48	0.56089(16)	0.73937(11)	0.64099(11)	0.0145(3)
C49	0.65848(16)	0.74647(11)	0.68026(11)	0.0152(3)

	x/a	y/b	z/c	U(eq)
C50	0.73832(16)	0.67698(11)	0.70478(11)	0.0150(3)
C51	0.72032(17)	0.59893(11)	0.68948(11)	0.0159(3)
C52	0.62380(16)	0.59300(11)	0.65022(11)	0.0148(3)
C53	0.8518(2)	0.75214(13)	0.76472(13)	0.0241(4)
C54	0.7683(2)	0.44653(12)	0.71754(15)	0.0259(4)
O1S	0.2715(4)	0.1303(3)	0.1306(3)	0.0438(9)
O2S	0.3973(3)	0.2364(3)	0.9972(3)	0.0476(9)
C1S	0.2821(5)	0.1859(4)	0.0539(4)	0.0410(11)
C2S	0.1676(5)	0.2103(9)	0.0161(4)	0.071(2)
C3S	0.5165(4)	0.2230(3)	0.0259(3)	0.0388(10)
C4S	0.6279(5)	0.2914(3)	0.9531(3)	0.0479(12)
O1T	0.3320(12)	0.1724(9)	0.0932(7)	0.079(3)
O2T	0.4189(7)	0.2598(7)	0.9491(7)	0.062(2)
C1T	0.3194(15)	0.2242(12)	0.0203(9)	0.070(4)
C2T	0.1817(19)	0.237(3)	0.0064(18)	0.168(16)
C3T	0.5526(8)	0.2354(7)	0.9574(6)	0.052(2)
C4T	0.6016(18)	0.2886(14)	0.9986(16)	0.120(8)

Bond lengths (Å) for rds625.

O1-C8	1.2211(19)	O2-N1	1.4023(16)
O2-C9	1.427(2)	O3-C23	1.3644(19)
O3-C26	1.430(2)	O4-C24	1.3644(19)
O4-C27	1.425(2)	N1-C8	1.372(2)
N1-C1	1.469(2)	C1-C6	1.537(2)
C1-C2	1.562(2)	C1-H1	1.0
C2-C20	1.524(2)	C2-C3	1.546(2)
C2-C7	1.552(2)	C3-C4	1.527(2)
C3-H3A	0.99	C3-H3B	0.99
C4-C5	1.526(2)	C4-H4A	0.99
C4-H4B	0.99	C5-C6	1.532(2)
C5-H5A	0.99	C5-H5B	0.99
C6-C10	1.468(2)	C6-H6	1.0
C7-C8	1.509(2)	C7-H7A	0.99
C7-H7B	0.99	C9-H9A	0.98
C9-H9B	0.98	C9-H9C	0.98
C10-C11	1.202(3)	C11-C12	1.375(3)
C12-C13	1.204(3)	C13-C14	1.431(3)
C14-C15	1.393(3)	C14-C19	1.397(3)
C15-C16	1.385(3)	C15-H15	0.95
C16-C17	1.382(4)	C16-H16	0.95
C17-C18	1.378(5)	C17-H17	0.95
C18-C19	1.392(4)	C18-H18	0.95

C19-H19	0.95	C20-C21	1.390(2)
C20-C25	1.411(2)	C21-C22	1.405(2)
C21-H21	0.95	C22-C23	1.380(2)
C22-H22	0.95	C23-C24	1.412(2)
C24-C25	1.384(2)	C25-H25	0.95
C26-H26A	0.98	C26-H26B	0.98
C26-H26C	0.98	C27-H27A	0.98
C27-H27B	0.98	C27-H27C	0.98
O5-C35	1.2186(19)	O6-N2	1.4005(17)
O6-C36	1.429(2)	O7-C50	1.3637(19)
O7-C53	1.431(2)	O8-C51	1.3659(19)
O8-C54	1.424(2)	N2-C35	1.374(2)
N2-C28	1.472(2)	C28-C33	1.531(2)
C28-C29	1.561(2)	C28-H28	1.0
C29-C47	1.527(2)	C29-C30	1.546(2)
C29-C34	1.549(2)	C30-C31	1.528(2)
C30-H30A	0.99	C30-H30B	0.99
C31-C32	1.528(2)	C31-H31A	0.99
C31-H31B	0.99	C32-C33	1.535(2)
C32-H32A	0.99	C32-H32B	0.99
C33-C37	1.468(2)	C33-H33	1.0
C34-C35	1.508(2)	C34-H34A	0.99
C34-H34B	0.99	C36-H36A	0.98
C36-H36B	0.98	C36-H36C	0.98
C37-C38	1.199(3)	C38-C39	1.379(3)
C39-C40	1.199(3)	C40-C41	1.432(3)
C41-C42	1.399(3)	C41-C46	1.403(3)
C42-C43	1.381(3)	C42-H42	0.95
C43-C44	1.394(3)	C43-H43	0.95
C44-C45	1.381(3)	C44-H44	0.95
C45-C46	1.382(3)	C45-H45	0.95
C46-H46	0.95	C47-C48	1.390(2)
C47-C52	1.406(2)	C48-C49	1.401(2)
C48-H48	0.95	C49-C50	1.382(2)
C49-H49	0.95	C50-C51	1.410(2)
C51-C52	1.385(2)	C52-H52	0.95
C53-H53A	0.98	C53-H53B	0.98
C53-H53C	0.98	C54-H54A	0.98
C54-H54B	0.98	C54-H54C	0.98
O1S-C1S	1.234(6)	O2S-C1S	1.314(6)
O2S-C3S	1.442(5)	C1S-C2S	1.490(5)
C2S-H2SA	0.98	C2S-H2SB	0.98
C2S-H2SC	0.98	C3S-C4S	1.473(6)

C3S-H3SA	0.99	C3S-H3SB	0.99
C4S-H4SA	0.98	C4S-H4SB	0.98
C4S-H4SC	0.98	O1T-C1T	1.240(13)
O2T-C1T	1.267(13)	O2T-C3T	1.466(10)
C1T-C2T	1.511(15)	C2T-H2TA	0.98
C2T-H2TB	0.98	C2T-H2TC	0.98
C3T-C4T	1.492(12)	C3T-H3TA	0.99
C3T-H3TB	0.99	C4T-H4TA	0.98
C4T-H4TB	0.98	C4T-H4TC	0.98

<u>Bond angles (°) for rds625.</u>

N1-O2-C9	109.08(12)	C23-O3-C26	117.36(13)
C24-O4-C27	116.38(13)	C8-N1-O2	115.72(12)
C8-N1-C1	111.75(12)	O2-N1-C1	117.15(12)
N1-C1-C6	115.16(13)	N1-C1-C2	99.93(12)
C6-C1-C2	114.05(12)	N1-C1-H1	109.1
C6-C1-H1	109.1	C2-C1-H1	109.1
C20-C2-C3	113.99(12)	C20-C2-C7	111.89(12)
C3-C2-C7	107.70(12)	C20-C2-C1	111.43(12)
C3-C2-C1	109.65(12)	C7-C2-C1	101.41(11)
C4-C3-C2	114.13(13)	C4-C3-H3A	108.7
C2-C3-H3A	108.7	C4-C3-H3B	108.7
C2-C3-H3B	108.7	H3A-C3-H3B	107.6
C5-C4-C3	110.13(13)	C5-C4-H4A	109.6
C3-C4-H4A	109.6	C5-C4-H4B	109.6
C3-C4-H4B	109.6	H4A-C4-H4B	108.1
C4-C5-C6	110.04(13)	C4-C5-H5A	109.7
C6-C5-H5A	109.7	C4-C5-H5B	109.7
C6-C5-H5B	109.7	H5A-C5-H5B	108.2
C10-C6-C5	112.91(14)	C10-C6-C1	110.97(13)
C5-C6-C1	113.39(14)	C10-C6-H6	106.3
C5-C6-H6	106.3	C1-C6-H6	106.3
C8-C7-C2	104.89(12)	C8-C7-H7A	110.8
C2-C7-H7A	110.8	C8-C7-H7B	110.8
C2-C7-H7B	110.8	H7A-C7-H7B	108.8
O1-C8-N1	124.59(15)	O1-C8-C7	128.40(15)
N1-C8-C7	107.00(13)	O2-C9-H9A	109.5
O2-C9-H9B	109.5	H9A-C9-H9B	109.5
O2-C9-H9C	109.5	H9A-C9-H9C	109.5
H9B-C9-H9C	109.5	C11-C10-C6	178.87(18)
C10-C11-C12	179.1(2)	C13-C12-C11	179.1(2)
C12-C13-C14	178.4(2)	C15-C14-C19	119.5(2)
C15-C14-C13	120.67(19)	C19-C14-C13	119.7(2)

C16-C15-C14	120.2(2)	C16-C15-H15	119.9
C14-C15-H15	119.9	C17-C16-C15	119.9(2)
C17-C16-H16	120.0	C15-C16-H16	120.0
C18-C17-C16	120.5(2)	C18-C17-H17	119.7
C16-C17-H17	119.7	C17-C18-C19	120.1(3)
C17-C18-H18	119.9	C19-C18-H18	119.9
C18-C19-C14	119.7(2)	C18-C19-H19	120.2
C14-C19-H19	120.2	C21-C20-C25	117.53(14)
C21-C20-C2	123.40(13)	C25-C20-C2	119.04(13)
C20-C21-C22	121.34(14)	C20-C21-H21	119.3
C22-C21-H21	119.3	C23-C22-C21	120.56(14)
C23-C22-H22	119.7	C21-C22-H22	119.7
O3-C23-C22	125.37(14)	O3-C23-C24	115.66(14)
C22-C23-C24	118.96(14)	O4-C24-C25	124.59(14)
O4-C24-C23	115.35(14)	C25-C24-C23	120.05(14)
C24-C25-C20	121.54(14)	C24-C25-H25	119.2
C20-C25-H25	119.2	O3-C26-H26A	109.5
O3-C26-H26B	109.5	H26A-C26-H26B	109.5
O3-C26-H26C	109.5	H26A-C26-H26C	109.5
H26B-C26-H26C	109.5	O4-C27-H27A	109.5
O4-C27-H27B	109.5	H27A-C27-H27B	109.5
O4-C27-H27C	109.5	H27A-C27-H27C	109.5
H27B-C27-H27C	109.5	N2-O6-C36	108.95(12)
C50-O7-C53	117.14(13)	C51-O8-C54	116.50(13)
C35-N2-O6	115.70(12)	C35-N2-C28	111.48(12)
O6-N2-C28	117.26(12)	N2-C28-C33	115.51(13)
N2-C28-C29	99.98(12)	C33-C28-C29	114.00(12)
N2-C28-H28	109.0	C33-C28-H28	109.0
C29-C28-H28	109.0	C47-C29-C30	114.01(12)
C47-C29-C34	112.21(12)	C30-C29-C34	107.67(12)
C47-C29-C28	110.99(12)	C30-C29-C28	109.66(12)
C34-C29-C28	101.54(12)	C31-C30-C29	114.13(13)
C31-C30-H30A	108.7	C29-C30-H30A	108.7
C31-C30-H30B	108.7	C29-C30-H30B	108.7
H30A-C30-H30B	107.6	C30-C31-C32	110.42(13)
C30-C31-H31A	109.6	C32-C31-H31A	109.6
C30-C31-H31B	109.6	C32-C31-H31B	109.6
H31A-C31-H31B	108.1	C31-C32-C33	109.60(14)
C31-C32-H32A	109.8	C33-C32-H32A	109.8
C31-C32-H32B	109.8	C33-C32-H32B	109.8
H32A-C32-H32B	108.2	C37-C33-C28	111.58(14)
C37-C33-C32	112.52(14)	C28-C33-C32	113.52(14)
C37-C33-H33	106.2	C28-C33-H33	106.2

C32-C33-H33	106.2	C35-C34-C29	105.00(12)
C35-C34-H34A	110.7	C29-C34-H34A	110.7
C35-C34-H34B	110.7	C29-C34-H34B	110.7
H34A-C34-H34B	108.8	O5-C35-N2	124.55(15)
O5-C35-C34	128.33(15)	N2-C35-C34	107.11(13)
O6-C36-H36A	109.5	O6-C36-H36B	109.5
H36A-C36-H36B	109.5	O6-C36-H36C	109.5
H36A-C36-H36C	109.5	H36B-C36-H36C	109.5
C38-C37-C33	179.19(19)	C37-C38-C39	178.2(2)
C40-C39-C38	179.4(2)	C39-C40-C41	176.1(2)
C42-C41-C46	119.51(17)	C42-C41-C40	120.61(17)
C46-C41-C40	119.75(18)	C43-C42-C41	119.99(18)
C43-C42-H42	120.0	C41-C42-H42	120.0
C42-C43-C44	119.9(2)	C42-C43-H43	120.1
C44-C43-H43	120.1	C45-C44-C43	120.6(2)
C45-C44-H44	119.7	C43-C44-H44	119.7
C44-C45-C46	120.0(2)	C44-C45-H45	120.0
C46-C45-H45	120.0	C45-C46-C41	120.07(19)
C45-C46-H46	120.0	C41-C46-H46	120.0
C48-C47-C52	117.60(14)	C48-C47-C29	123.45(13)
C52-C47-C29	118.93(13)	C47-C48-C49	121.40(14)
C47-C48-H48	119.3	C49-C48-H48	119.3
C50-C49-C48	120.47(14)	C50-C49-H49	119.8
C48-C49-H49	119.8	O7-C50-C49	125.37(14)
O7-C50-C51	115.60(14)	C49-C50-C51	119.03(14)
O8-C51-C52	124.46(14)	O8-C51-C50	115.65(14)
C52-C51-C50	119.89(14)	C51-C52-C47	121.61(14)
C51-C52-H52	119.2	C47-C52-H52	119.2
O7-C53-H53A	109.5	O7-C53-H53B	109.5
H53A-C53-H53B	109.5	O7-C53-H53C	109.5
H53A-C53-H53C	109.5	H53B-C53-H53C	109.5
O8-C54-H54A	109.5	O8-C54-H54B	109.5
H54A-C54-H54B	109.5	O8-C54-H54C	109.5
H54A-C54-H54C	109.5	H54B-C54-H54C	109.5
C1S-O2S-C3S	117.8(4)	O1S-C1S-O2S	122.9(4)
O1S-C1S-C2S	125.5(5)	O2S-C1S-C2S	111.5(5)
C1S-C2S-H2SA	109.5	C1S-C2S-H2SB	109.5
H2SA-C2S-H2SB	109.5	C1S-C2S-H2SC	109.5
H2SA-C2S-H2SC	109.5	H2SB-C2S-H2SC	109.5
O2S-C3S-C4S	106.8(4)	O2S-C3S-H3SA	110.4
C4S-C3S-H3SA	110.4	O2S-C3S-H3SB	110.4
C4S-C3S-H3SB	110.4	H3SA-C3S-H3SB	108.6
C3S-C4S-H4SA	109.5	C3S-C4S-H4SB	109.5

H4SA-C4S-H4SB	109.5	C3S-C4S-H4SC	109.5
H4SA-C4S-H4SC	109.5	H4SB-C4S-H4SC	109.5
C1T-O2T-C3T	115.7(10)	O1T-C1T-O2T	123.2(12)
O1T-C1T-C2T	122.0(16)	O2T-C1T-C2T	114.0(14)
C1T-C2T-H2TA	109.5	C1T-C2T-H2TB	109.5
H2TA-C2T-H2TB	109.5	C1T-C2T-H2TC	109.5
H2TA-C2T-H2TC	109.5	H2TB-C2T-H2TC	109.5
O2T-C3T-C4T	109.2(9)	O2T-C3T-H3TA	109.8
C4T-C3T-H3TA	109.8	O2T-C3T-H3TB	109.8
C4T-C3T-H3TB	109.8	H3TA-C3T-H3TB	108.3
C3T-C4T-H4TA	109.5	C3T-C4T-H4TB	109.5
H4TA-C4T-H4TB	109.5	C3T-C4T-H4TC	109.5
H4TA-C4T-H4TC	109.5	H4TB-C4T-H4TC	109.5

Torsion angles (°) for rds625.

C9-O2-N1-C8	101.72(16)	C9-O2-N1-C1	-123.10(15)
C8-N1-C1-C6	-159.34(13)	O2-N1-C1-C6	63.80(17)
C8-N1-C1-C2	-36.70(15)	O2-N1-C1-C2	-173.57(11)
N1-C1-C2-C20	155.69(12)	C6-C1-C2-C20	-80.90(16)
N1-C1-C2-C3	-77.17(14)	C6-C1-C2-C3	46.25(17)
N1-C1-C2-C7	36.50(13)	C6-C1-C2-C7	159.91(13)
C20-C2-C3-C4	74.21(17)	C7-C2-C3-C4	-161.02(13)
C1-C2-C3-C4	-51.49(17)	C2-C3-C4-C5	58.78(18)
C3-C4-C5-C6	-58.20(18)	C4-C5-C6-C10	-177.89(14)
C4-C5-C6-C1	54.79(19)	N1-C1-C6-C10	-63.35(18)
C2-C1-C6-C10	-178.06(13)	N1-C1-C6-C5	64.97(17)
C2-C1-C6-C5	-49.74(18)	C20-C2-C7-C8	-145.36(12)
C3-C2-C7-C8	88.62(14)	C1-C2-C7-C8	-26.49(14)
O2-N1-C8-O1	-21.0(2)	C1-N1-C8-O1	-158.49(14)
O2-N1-C8-C7	157.81(12)	C1-N1-C8-C7	20.29(16)
C2-C7-C8-O1	-175.77(15)	C2-C7-C8-N1	5.52(16)
C19-C14-C15-C16	1.3(4)	C13-C14-C15-C16	178.1(2)
C14-C15-C16-C17	-2.8(5)	C15-C16-C17-C18	1.6(6)
C16-C17-C18-C19	1.1(7)	C17-C18-C19-C14	-2.5(6)
C15-C14-C19-C18	1.4(5)	C13-C14-C19-C18	-175.4(3)
C3-C2-C20-C21	8.6(2)	C7-C2-C20-C21	-113.91(16)
C1-C2-C20-C21	133.34(15)	C3-C2-C20-C25	-173.60(14)
C7-C2-C20-C25	63.90(18)	C1-C2-C20-C25	-48.84(18)
C25-C20-C21-C22	0.7(2)	C2-C20-C21-C22	178.59(14)
C20-C21-C22-C23	-0.2(2)	C26-O3-C23-C22	-2.1(2)
C26-O3-C23-C24	178.53(15)	C21-C22-C23-O3	-179.32(15)
C21-C22-C23-C24	0.0(2)	C27-O4-C24-C25	11.8(2)
C27-O4-C24-C23	-169.34(16)	O3-C23-C24-O4	0.0(2)

C22-C23-C24-O4	-179.42(15)	O3-C23-C24-C25	178.94(15)
C22-C23-C24-C25	-0.5(2)	O4-C24-C25-C20	179.92(15)
C23-C24-C25-C20	1.1(2)	C21-C20-C25-C24	-1.2(2)
C2-C20-C25-C24	-179.13(15)	C36-O6-N2-C35	-102.43(16)
C36-O6-N2-C28	122.73(15)	C35-N2-C28-C33	159.28(13)
O6-N2-C28-C33	-64.08(17)	C35-N2-C28-C29	36.50(15)
O6-N2-C28-C29	173.13(12)	N2-C28-C29-C47	-155.79(12)
C33-C28-C29-C47	80.36(16)	N2-C28-C29-C30	77.34(14)
C33-C28-C29-C30	-46.51(17)	N2-C28-C29-C34	-36.35(14)
C33-C28-C29-C34	-160.20(13)	C47-C29-C30-C31	-74.01(17)
C34-C29-C30-C31	160.81(13)	C28-C29-C30-C31	51.13(17)
C29-C30-C31-C32	-58.39(18)	C30-C31-C32-C33	57.97(18)
N2-C28-C33-C37	63.88(18)	C29-C28-C33-C37	178.88(13)
N2-C28-C33-C32	-64.54(18)	C29-C28-C33-C32	50.46(18)
C31-C32-C33-C37	176.95(15)	C31-C32-C33-C28	-55.12(19)
C47-C29-C34-C35	144.92(13)	C30-C29-C34-C35	-88.83(14)
C28-C29-C34-C35	26.35(15)	O6-N2-C35-O5	21.3(2)
C28-N2-C35-O5	158.65(15)	O6-N2-C35-C34	-157.60(12)
C28-N2-C35-C34	-20.24(17)	C29-C34-C35-O5	175.78(15)
C29-C34-C35-N2	-5.38(16)	C46-C41-C42-C43	-0.3(3)
C40-C41-C42-C43	-176.0(2)	C41-C42-C43-C44	0.8(4)
C42-C43-C44-C45	-0.8(4)	C43-C44-C45-C46	0.1(4)
C44-C45-C46-C41	0.5(4)	C42-C41-C46-C45	-0.4(3)
C40-C41-C46-C45	175.4(2)	C30-C29-C47-C48	-7.3(2)
C34-C29-C47-C48	115.48(16)	C28-C29-C47-C48	-131.69(15)
C30-C29-C47-C52	174.10(14)	C34-C29-C47-C52	-63.17(18)
C28-C29-C47-C52	49.66(18)	C52-C47-C48-C49	-0.3(2)
C29-C47-C48-C49	-178.94(15)	C47-C48-C49-C50	-0.1(2)
C53-O7-C50-C49	5.3(2)	C53-O7-C50-C51	-175.64(15)
C48-C49-C50-O7	179.14(16)	C48-C49-C50-C51	0.2(2)
C54-O8-C51-C52	-11.8(3)	C54-O8-C51-C50	167.97(16)
O7-C50-C51-O8	1.3(2)	C49-C50-C51-O8	-179.66(15)
O7-C50-C51-C52	-178.96(15)	C49-C50-C51-C52	0.1(2)
O8-C51-C52-C47	179.27(16)	C50-C51-C52-C47	-0.5(3)
C48-C47-C52-C51	0.6(2)	C29-C47-C52-C51	179.29(15)
C3S-O2S-C1S-O1S	2.3(7)	C3S-O2S-C1S-C2S	177.6(5)
C1S-O2S-C3S-C4S	-176.3(4)	C3T-O2T-C1T-O1T	1.(2)
C3T-O2T-C1T-C2T	-170.(2)	C1T-O2T-C3T-C4T	-76.8(16)

Anisotropic atomic displacement parameters (Å^2) for rds625.

The anisotropic atomic displacement factor exponent takes the form: $-2\pi^2[h^2 a^{*2} U_{11} + ... + 2 h k a^* b^* U_{12}]$

	U_{11}	U_{22}	U_{33}	U_{23}	U_{13}	U_{12}
O1	0.0180(5)	0.0170(5)	0.0174(5)	-0.0113(5)	-0.0034(4)	0.0013(4)

	U_{11}	U_{22}	U_{33}	U_{23}	U_{13}	U_{12}
O2	0.0145(5)	0.0139(5)	0.0240(6)	-0.0103(5)	-0.0077(5)	0.0061(4)
O3	0.0137(5)	0.0178(6)	0.0291(7)	-0.0120(5)	-0.0008(5)	0.0039(4)
O4	0.0128(5)	0.0155(5)	0.0374(7)	-0.0140(5)	-0.0025(5)	0.0004(4)
N1	0.0130(6)	0.0133(6)	0.0163(6)	-0.0086(5)	-0.0052(5)	0.0049(5)
C1	0.0112(6)	0.0134(6)	0.0145(7)	-0.0080(5)	-0.0046(5)	0.0029(5)
C2	0.0109(6)	0.0120(6)	0.0140(7)	-0.0073(5)	-0.0040(5)	0.0026(5)
C3	0.0119(6)	0.0141(7)	0.0192(7)	-0.0091(6)	-0.0038(6)	0.0002(5)
C4	0.0172(7)	0.0175(7)	0.0248(8)	-0.0129(6)	-0.0099(6)	0.0018(6)
C5	0.0203(8)	0.0221(8)	0.0273(9)	-0.0154(7)	-0.0141(7)	0.0053(6)
C6	0.0180(7)	0.0174(7)	0.0161(7)	-0.0092(6)	-0.0075(6)	0.0040(6)
C7	0.0147(7)	0.0141(7)	0.0136(7)	-0.0069(6)	-0.0051(5)	0.0030(5)
C8	0.0120(6)	0.0129(6)	0.0160(7)	-0.0074(6)	-0.0039(5)	-0.0009(5)
C9	0.0134(7)	0.0249(8)	0.0332(10)	-0.0159(8)	-0.0090(7)	0.0077(6)
C10	0.0203(8)	0.0230(8)	0.0159(7)	-0.0096(6)	-0.0083(6)	0.0045(6)
C11	0.0231(8)	0.0236(8)	0.0173(8)	-0.0086(7)	-0.0082(6)	0.0026(7)
C12	0.0254(9)	0.0241(8)	0.0180(8)	-0.0083(7)	-0.0078(7)	0.0029(7)
C13	0.0285(9)	0.0241(9)	0.0201(8)	-0.0064(7)	-0.0081(7)	0.0046(7)
C14	0.0324(10)	0.0220(9)	0.0259(9)	-0.0025(7)	-0.0085(8)	0.0064(7)
C15	0.0351(11)	0.0306(10)	0.0263(10)	-0.0064(8)	-0.0074(8)	0.0092(8)
C16	0.0468(15)	0.0380(13)	0.0524(16)	-0.0091(12)	-0.0106(12)	0.0227(11)
C17	0.063(2)	0.0363(14)	0.070(2)	0.0104(14)	-0.0085(17)	0.0222(14)
C18	0.0591(19)	0.0468(17)	0.0598(19)	0.0241(15)	0.0096(16)	0.0149(14)
C19	0.0394(13)	0.0399(13)	0.0451(15)	0.0067(11)	0.0055(11)	0.0145(11)
C20	0.0111(6)	0.0123(6)	0.0126(6)	-0.0058(5)	-0.0041(5)	0.0022(5)
C21	0.0138(7)	0.0125(6)	0.0162(7)	-0.0074(6)	-0.0053(6)	0.0031(5)
C22	0.0159(7)	0.0136(7)	0.0171(7)	-0.0089(6)	-0.0055(6)	0.0046(5)
C23	0.0126(7)	0.0157(7)	0.0151(7)	-0.0069(6)	-0.0028(6)	0.0043(5)
C24	0.0124(7)	0.0138(7)	0.0190(7)	-0.0070(6)	-0.0042(6)	0.0014(5)
C25	0.0129(7)	0.0129(7)	0.0181(7)	-0.0086(6)	-0.0043(6)	0.0022(5)
C26	0.0197(8)	0.0218(8)	0.0288(9)	-0.0135(7)	0.0018(7)	0.0056(6)
C27	0.0192(8)	0.0148(7)	0.0346(10)	-0.0123(7)	-0.0065(7)	0.0005(6)
O5	0.0228(6)	0.0174(5)	0.0193(6)	-0.0119(5)	-0.0095(5)	0.0071(4)
O6	0.0153(5)	0.0134(5)	0.0262(6)	-0.0109(5)	-0.0080(5)	0.0000(4)
O7	0.0234(6)	0.0186(6)	0.0299(7)	-0.0145(5)	-0.0171(5)	0.0048(5)
O8	0.0262(6)	0.0174(6)	0.0427(8)	-0.0179(6)	-0.0245(6)	0.0112(5)
N2	0.0148(6)	0.0135(6)	0.0178(6)	-0.0089(5)	-0.0069(5)	0.0003(5)
C28	0.0132(6)	0.0130(6)	0.0152(7)	-0.0076(6)	-0.0056(5)	0.0020(5)
C29	0.0125(6)	0.0110(6)	0.0145(7)	-0.0068(5)	-0.0053(5)	0.0030(5)
C30	0.0167(7)	0.0132(7)	0.0192(7)	-0.0088(6)	-0.0092(6)	0.0054(5)
C31	0.0155(7)	0.0180(7)	0.0257(8)	-0.0136(7)	-0.0077(6)	0.0067(6)
C32	0.0144(7)	0.0217(8)	0.0271(9)	-0.0134(7)	-0.0032(6)	0.0048(6)
C33	0.0152(7)	0.0175(7)	0.0176(7)	-0.0085(6)	-0.0035(6)	0.0017(6)

	U_{11}	U_{22}	U_{33}	U_{23}	U_{13}	U_{12}
C34	0.0149(7)	0.0142(7)	0.0144(7)	-0.0070(6)	-0.0048(6)	0.0017(5)
C35	0.0155(7)	0.0128(6)	0.0168(7)	-0.0076(6)	-0.0076(6)	0.0049(5)
C36	0.0168(8)	0.0271(9)	0.0378(10)	-0.0174(8)	-0.0132(7)	0.0020(7)
C37	0.0180(7)	0.0211(8)	0.0183(8)	-0.0092(6)	-0.0031(6)	0.0021(6)
C38	0.0193(8)	0.0236(8)	0.0188(8)	-0.0081(7)	-0.0040(6)	0.0029(6)
C39	0.0210(8)	0.0229(8)	0.0183(8)	-0.0062(7)	-0.0041(7)	0.0030(6)
C40	0.0231(8)	0.0242(8)	0.0192(8)	-0.0070(7)	-0.0051(7)	0.0018(7)
C41	0.0239(8)	0.0205(8)	0.0193(8)	-0.0050(7)	-0.0050(7)	-0.0004(7)
C42	0.0315(10)	0.0276(9)	0.0193(8)	-0.0071(7)	-0.0084(7)	0.0005(8)
C43	0.0413(12)	0.0357(11)	0.0358(11)	-0.0137(9)	-0.0173(10)	-0.0058(9)
C44	0.0426(13)	0.0273(10)	0.0342(11)	-0.0030(9)	-0.0092(10)	-0.0134(9)
C45	0.0456(13)	0.0292(10)	0.0212(9)	-0.0004(8)	-0.0095(9)	-0.0087(9)
C46	0.0426(12)	0.0288(10)	0.0206(9)	-0.0040(8)	-0.0123(8)	-0.0077(9)
C47	0.0114(6)	0.0123(6)	0.0139(7)	-0.0065(5)	-0.0036(5)	0.0012(5)
C48	0.0141(7)	0.0123(6)	0.0185(7)	-0.0077(6)	-0.0056(6)	0.0029(5)
C49	0.0169(7)	0.0131(7)	0.0181(7)	-0.0090(6)	-0.0055(6)	0.0015(5)
C50	0.0143(7)	0.0158(7)	0.0174(7)	-0.0083(6)	-0.0068(6)	0.0005(5)
C51	0.0162(7)	0.0144(7)	0.0214(8)	-0.0092(6)	-0.0097(6)	0.0041(5)
C52	0.0151(7)	0.0137(7)	0.0206(7)	-0.0106(6)	-0.0079(6)	0.0039(5)
C53	0.0344(10)	0.0209(8)	0.0283(9)	-0.0152(7)	-0.0192(8)	0.0044(7)
C54	0.0287(9)	0.0165(8)	0.0433(11)	-0.0161(8)	-0.0219(9)	0.0097(7)
O1S	0.0486(19)	0.060(2)	0.0409(18)	-0.0353(16)	-0.0199(15)	0.0186(15)
O2S	0.0277(15)	0.077(2)	0.0345(18)	-0.0199(17)	-0.0117(14)	0.0167(15)
C1S	0.035(2)	0.074(3)	0.040(2)	-0.043(2)	-0.0215(18)	0.029(2)
C2S	0.027(2)	0.162(7)	0.036(2)	-0.051(3)	-0.0165(18)	0.037(3)
C3S	0.0371(18)	0.050(2)	0.040(2)	-0.0252(17)	-0.0199(15)	0.0162(16)
C4S	0.040(2)	0.047(2)	0.055(3)	-0.020(2)	-0.015(2)	0.0140(18)
O1T	0.085(7)	0.104(7)	0.057(5)	-0.045(5)	-0.019(5)	0.041(6)
O2T	0.049(4)	0.092(6)	0.056(5)	-0.041(4)	-0.020(3)	0.015(3)
C1T	0.066(6)	0.093(9)	0.056(5)	-0.040(5)	-0.012(5)	0.020(6)
C2T	0.072(9)	0.28(4)	0.122(16)	-0.056(17)	-0.036(9)	0.035(11)
C3T	0.043(4)	0.066(5)	0.057(6)	-0.033(4)	-0.019(4)	0.009(4)
C4T	0.099(11)	0.166(16)	0.22(2)	-0.159(18)	-0.111(14)	0.075(11)

Hydrogen atomic coordinates and isotropic atomic displacement parameters (Å^2) for rds625.

	x/a	y/b	z/c	U(eq)
H1	-0.0576	0.0068	0.6581	0.015
H3A	0.1754	0.2263	0.5237	0.018
H3B	0.0582	0.2878	0.5411	0.018
H4A	0.1921	0.2693	0.6353	0.021
H4B	0.0385	0.2236	0.6942	0.021
H5A	0.1906	0.1207	0.7481	0.024

	x/a	y/b	z/c	U(eq)
H5B	0.2582	0.1229	0.6516	0.024
H6	-0.0180	0.0527	0.7606	0.019
H7A	-0.0920	0.1266	0.4929	0.016
H7B	0.0287	0.2111	0.4442	0.016
H9A	0.3671	0.0196	0.5016	0.033
H9B	0.3632	-0.0802	0.5782	0.033
H9C	0.3448	0.0067	0.6012	0.033
H15	0.4124	-0.3586	0.8246	0.04
H16	0.4682	-0.5045	0.9004	0.061
H17	0.3301	-0.6001	1.0436	0.085
H18	0.1310	-0.5540	1.1092	0.094
H19	0.0644	-0.4126	1.0297	0.066
H21	-0.1296	0.2873	0.6265	0.016
H22	-0.3558	0.2989	0.6948	0.018
H25	-0.2479	0.0378	0.6469	0.017
H26A	-0.5827	0.3096	0.7131	0.037
H26B	-0.5633	0.2589	0.8092	0.037
H26C	-0.7082	0.2457	0.7971	0.037
H27A	-0.4332	-0.0473	0.6651	0.034
H27B	-0.5600	-0.0987	0.7533	0.034
H27C	-0.4141	-0.0786	0.7609	0.034
H28	0.4106	0.5087	0.6561	0.016
H30A	0.4004	0.7894	0.5386	0.018
H30B	0.2990	0.7276	0.5234	0.018
H31A	0.1774	0.7709	0.6356	0.022
H31B	0.2780	0.7262	0.6924	0.022
H32A	0.0966	0.6236	0.6552	0.025
H32B	0.0756	0.6225	0.7508	0.025
H33	0.2769	0.5563	0.7588	0.02
H34A	0.5200	0.7118	0.4412	0.017
H34B	0.5976	0.6277	0.4885	0.017
H36A	0.1266	0.5176	0.5071	0.037
H36B	0.0584	0.5072	0.6064	0.037
H36C	0.0605	0.4187	0.5861	0.037
H42	-0.2016	0.1447	0.8202	0.032
H43	-0.3537	0.0095	0.8993	0.044
H44	-0.3920	-0.0744	1.0522	0.047
H45	-0.2754	-0.0258	1.1256	0.043
H46	-0.1197	0.1076	1.0470	0.039
H48	0.5068	0.7878	0.6246	0.017
H49	0.6699	0.7994	0.6901	0.018
H52	0.6130	0.5403	0.6398	0.018

	x/a	y/b	z/c	U(eq)
H53A	0.8780	0.8099	0.7104	0.036
H53B	0.9222	0.7429	0.7939	0.036
H53C	0.7650	0.7549	0.8052	0.036
H54A	0.7820	0.4540	0.6567	0.039
H54B	0.6727	0.4235	0.7532	0.039
H54C	0.8267	0.4026	0.7439	0.039
H2SA	0.1662	0.1772	-0.0202	0.106
H2SB	0.1799	0.2767	-0.0211	0.106
H2SC	0.0812	0.1931	0.0646	0.106
H3SA	0.5399	0.1599	0.0382	0.047
H3SB	0.4999	0.2320	0.0809	0.047
H4SA	0.6417	0.2826	-0.1012	0.072
H4SB	0.7118	0.2836	-0.0306	0.072
H4SC	0.6043	0.3534	-0.0574	0.072
H2TA	0.1206	0.1790	0.0431	0.252
H2TB	0.1917	0.2549	-0.0564	0.252
H2TC	0.1434	0.2850	0.0235	0.252
H3TA	0.6181	0.2497	-0.1021	0.063
H3TB	0.5456	0.1686	-0.0051	0.063
H4TA	0.6004	0.3544	-0.0358	0.18
H4TB	0.6948	0.2772	-0.0011	0.18
H4TC	0.5419	0.2693	0.0599	0.18
